# Enantioselective NHC-Catalyzed Annulation via Umpolung for the Construction of Hydroxylamine Architectures with Variable Ring Sizes

**DOI:** 10.1021/acs.orglett.5c02576

**Published:** 2025-08-18

**Authors:** Izabela Barańska, Monika Radosińska, Liliana Dobrzańska, Krzysztof Dzieszkowski, Zbigniew Rafiński

**Affiliations:** † Nicolaus Copernicus University in Toruń, Faculty of Chemistry, 7 Gagarin Street, 87-100 Toruń, Poland; ‡ Jagiellonian University, Faculty of Chemistry, Gronostajowa 2, 30-387 Kraków, Poland

## Abstract

An efficient and highly enantioselective organocatalytic annulation for the synthesis of chiral hydroxylamine-containing heterocycles is described. The *N*-heterocyclic carbene (NHC)-catalyzed reaction successfully engages traditionally inert keto-oxime ethers as electrophiles in an intramolecular cyclization with in situ generated Breslow intermediates. This protocol overcomes the inherent challenges of this substrate class, such as low C=N bond electrophilicity and potential N–O bond cleavage, to deliver diverse five- and six-membered N–O frameworks in excellent yields (up to 99%) and enantioselectivities (up to >99% ee). This work provides a powerful, metal-free strategy to access valuable chiral building blocks and significantly expands the reactivity scope of keto-oximes in asymmetric catalysis.

## Graphical abstract

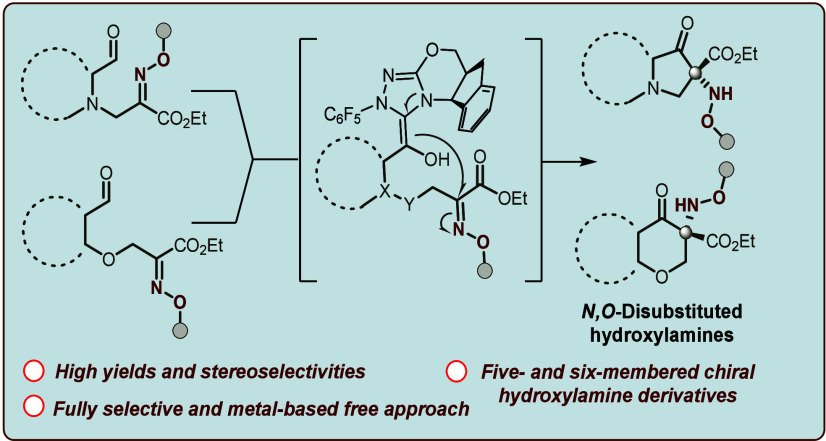

Chiral hydroxylamines, defined by their characteristic N–O bond, are pivotal structural motifs in bioactive molecules, including the agrochemical Clomazone and pharmaceuticals such as Zileuton and Spiropidion (Figure 1A).^1^ This N–O bond endows these compounds with unique pharmacokinetic properties, such as enhanced metabolic stability and improved target binding affinity.^2^ Despite their importance, the enantioselective synthesis of hydroxylamines remains considerably less developed than that of their amine counterparts, primarily due to intrinsic challenges in oxime chemistry.^3^

**Figure 1:**
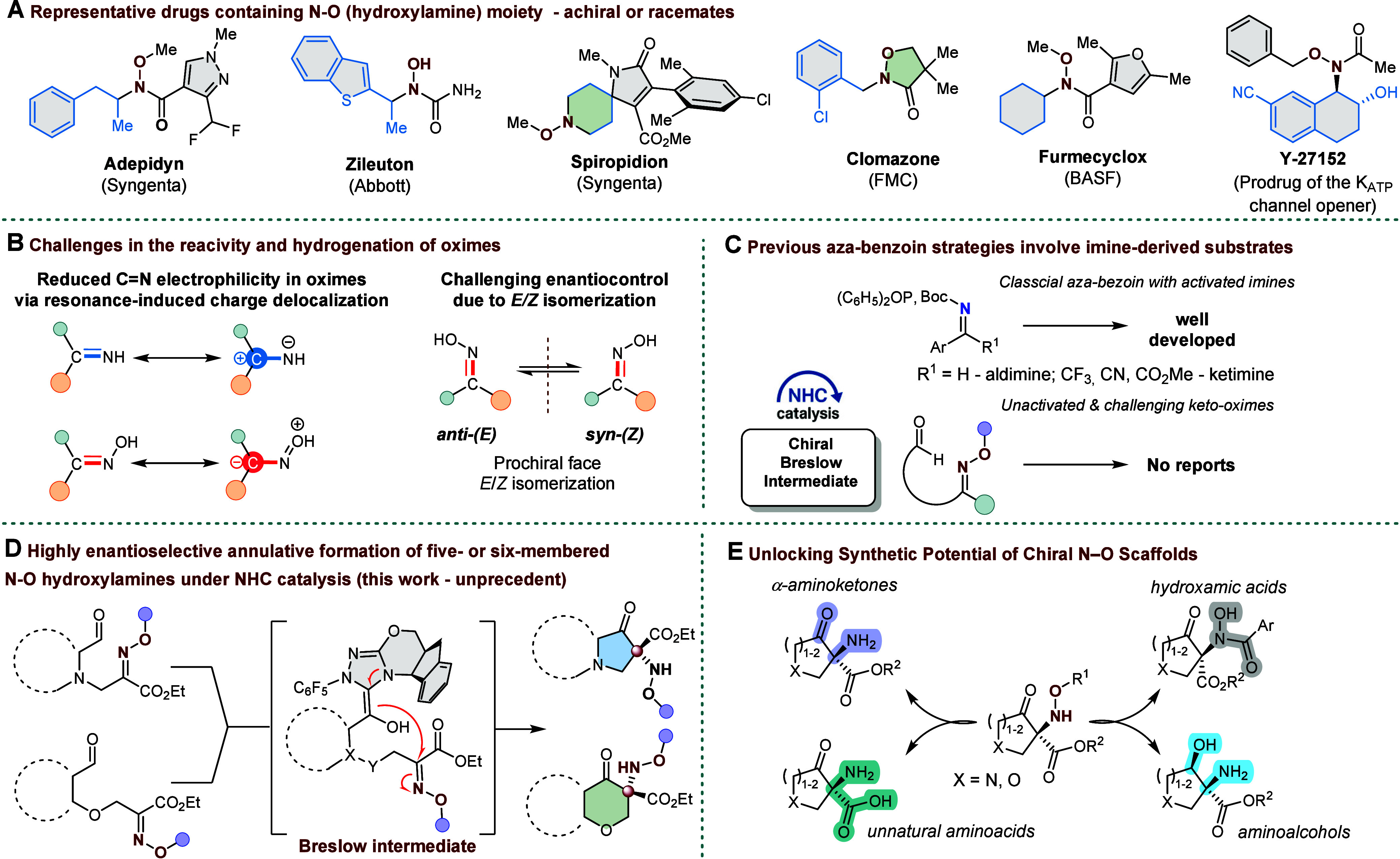
Importance of the functional *N–O* (hydroxylamino) group, synthesis challenges and strategies, and NHC-catalyzed annulation for *N*-alkoxyamine synthesis.

Oximes, which feature a C=N–O linkage, present significant obstacles to stereoselective transformations. In particular, resonance delocalization reduces the C=N bond’s electrophilicity, thereby diminishing its reactivity toward nucleophilic attack (Figure 1B). Furthermore, the low bond dissociation energy of the N–O bond (∼57 kcal/mol) predisposes it to cleavage under reductive conditions, while E/Z isomerization complicates stereochemical control.^4^ While asymmetric hydrogenation of imines is a well-advanced field, the analogous transformation for oximes has lagged behind, often plagued by over-reduction.^5^ Stoichiometric chiral reducing agents offer some stereocontrol but suffer from high cost, low atom economy, and poor scalability.^6^ One of the most direct approaches involves transition-metal-catalyzed hydrogenation. Two landmark studies by Cramer^7^ and Zhang^8^ have recently broadened this field, demonstrating highly enantioselective hydrogenations of oximes with sophisticated Iridium and Nickel catalysts that prevent N–O bond cleavage. Notably, the applicability of these pioneering methods to more structurally complex or electronically diverse substrates continues to evolve. This underscores the need for innovative solutions, such as organocatalysis, to overcome the limitations associated with high hydrogen pressures or specialized metal catalysts.

Organocatalysis, particularly using *N*-heterocyclic carbenes (NHCs), offers a compelling alternative. NHCs have proven exceptionally versatile in polarity-reversal (Umpolung) reactions of aldehydes, and *aza*-benzoin reactions have emerged as a robust strategy for stereocontrolled C–N bond formation.^9,10^ This field has been extensively developed by Rovis, You, Ye, Chi, and others for reactions with activated ketimines.^11^ It is crucial to note, however, that these established methods almost exclusively rely on activated imine electrophiles (e.g., *N*-Boc, *N*-phosphinoyl imines). Consequently, the significantly more challenging keto-oxime ethers—which pose unique difficulties such as diminished electrophilicity and E/Z isomerism—have remained an unexplored class of substrates for this powerful strategy.

To date, no NHC-catalyzed enantioselective intramolecular *aza*-benzoin-type reactions with keto-oxime ethers have been described. While the formation of Breslow intermediates is widely known, their strategic deployment to promote selective cyclization onto substrates traditionally considered inert is unprecedented (Figure 1C). In response to these challenges, we now present the first organocatalytic strategy for constructing chiral N–O disubstituted *O*-alkylhydroxylamines via an annulative formation of five- and six-membered frameworks under NHC catalysis. This approach provides a direct organocatalytic solution to the challenge of C=N bond functionalization in otherwise inert oximes (Figure 1D). Crucially, the resulting hydroxylamine framework is highly functionalized, enabling straightforward synthetic access to medicinally relevant hydroxamic acids, α-amino ketones, and non-natural amino acids (Figure 1E).

At the outset, the O-Bn oxime ester derived from indole-2-carbaldehyde **1a** was selected as the model substrate to investigate our proposed intramolecular umpolung strategy (Table 1). The initial screening focused on identifying the optimal *N*-heterocyclic carbene (NHC) catalyst. When the carbene derived from precatalyst **A** was employed with K_3_PO_4_ as the base, the desired pyrrolo­[1,2-*a*]­indole **2a** was formed in 22% yield and 66% ee (entry 1). Replacing the pyroglutamic acid moiety with phenylglycine (preNHC **B**) significantly improved the outcome to 61% yield and 90% ee (entry 2).

**Table 1:**
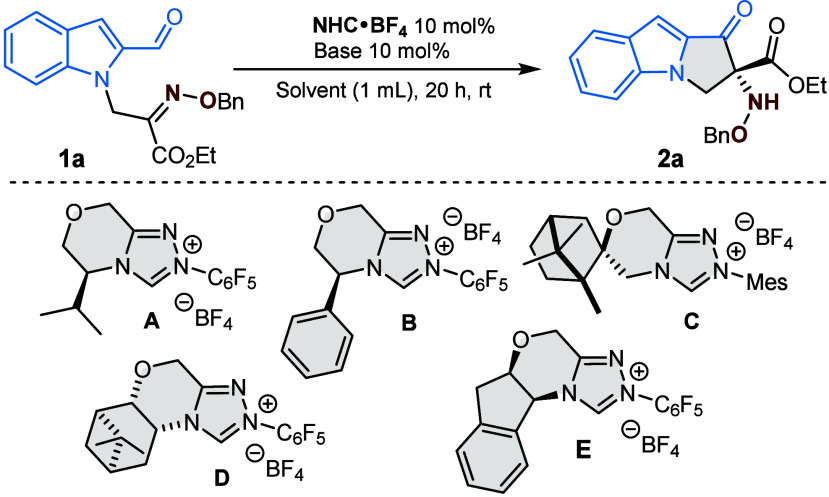
Optimization of the Reaction Conditions^a^

entry	NHC·HBF_4_	solvent	base	yield^b^ (%)	ee^c^
1	A	THF	K_3_PO_4_	22	66
2	B	THF	K_3_PO_4_	61	90
3	C	THF	K_3_PO_4_	75	12
4	D	THF	K_3_PO_4_	5	
5	E	THF	K_3_PO_4_	99	96

a The reaction conditions were carried out using **1a** (0.10 mmol), NHC·HBF_4_ catalyst **A**–**E** (10 mol %), base (10 mol %) and solvent (1.0 mL) at room temperature for 24 h.

b The ^1^H NMR yield of a crude product was determined with the aid of 1,1,2,2-tetrachloroethane as an internal standard.

c The HPLC analysis on a chiral stationary phase was used for determining er.

While the spirocamphor-based preNHC **C** gave a good yield (75%), the enantioselectivity was poor (entry 3), and the pinene-derived precatalyst **D** was found to be completely inactive (entry 4). To our delight, the aminoindanol-derived preNHC **E** proved to be superior, providing the target hydroxylamine **2a** with excellent yield and enantioselectivity (99% yield, 96% ee, entry 5). Further screening of various solvents and bases confirmed these conditions were optimal (for the full optimization data, see Table S1 in the Supporting Information). Consequently, the optimal reaction conditions were established as precatalyst **E** (10 mol %), K_3_PO_4_ as the base, and THF as the solvent at room temperature for 24 h.

A series of indole-2-carbaldehyde derivatives bearing electron-donating, neutral, or electron-withdrawing substituents at the 5-position underwent smooth intramolecular cyclization, affording tricyclic pyrrolo­[1,2-*a*]­indole derivatives **2a**–**2e** bearing a chiral *N*-benzyloxyamine unit in excellent yields and with enantioselectivities exceeding 94% ee in all cases (Scheme 1). Notably, compound **2a** was obtained in 90% yield and 96% ee on a 1.0 mmol scale, demonstrating the scalability and practicality of this method. Furthermore, substituents at the 3- and 6-positions of the indole ring were similarly well tolerated, producing the corresponding *N*-benzyloxyamine-containing pyrrolo­[1,2-*a*]­indoles **2f**–**2i** in good to excellent yields and ≥ 96% ee.

**Scheme 1:**
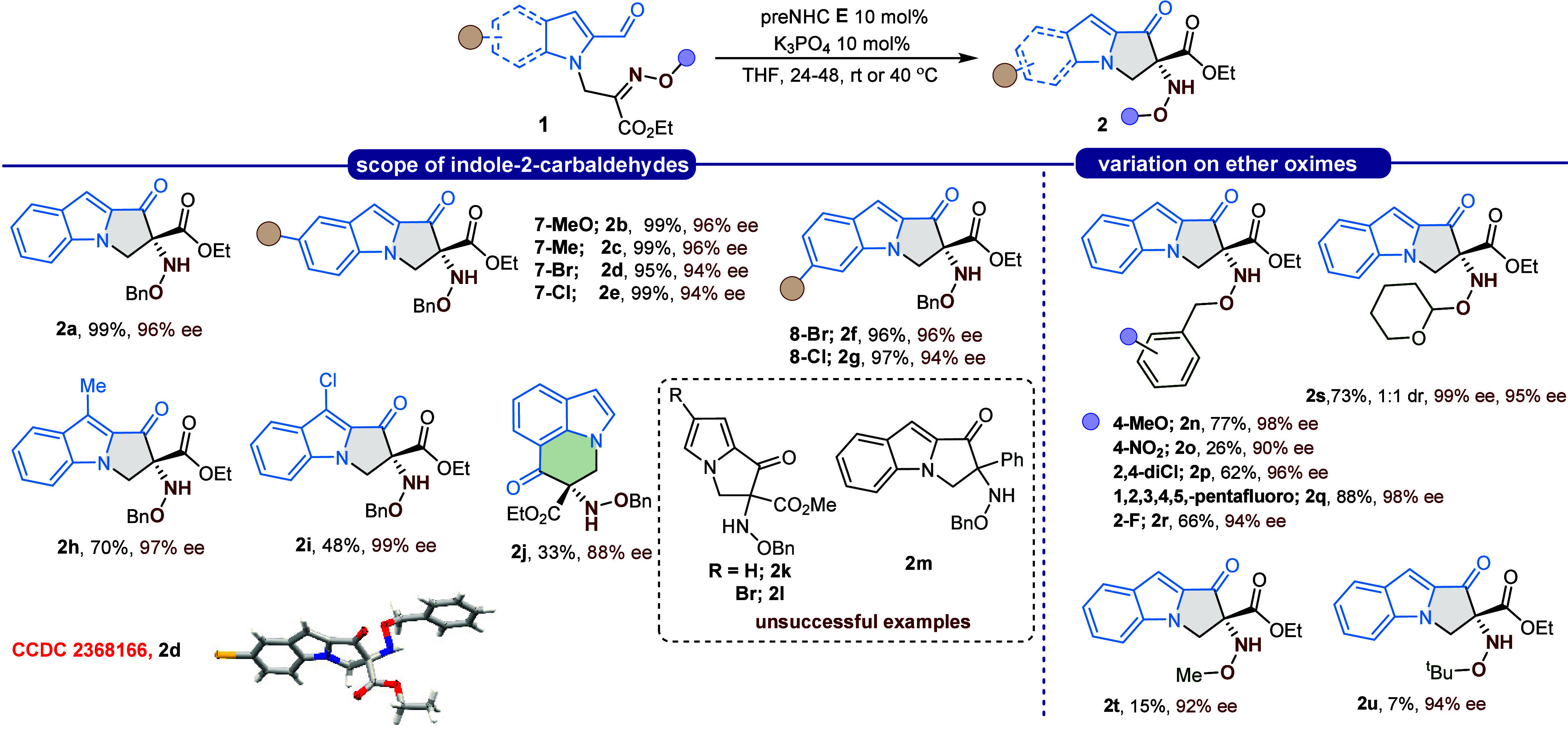
Scope of Indole Substitution and Ether Oxime Substrates^a^

a Reaction conditions: **1** (0.1 mmol), NHC·HBF_4_ **E** (10 mol %), K_3_PO_4_ (10 mol %) in THF (1.0 mL) at rt or 40 °C, and 24–48 h. Given are yields of the column chromatography-purified products. *ee* values determined by HPLC on chiral stationary phase.

The structure and absolute stereochemistry of the hydroxylamine moiety in **2d** were confirmed by SCXRD analysis. Interestingly, an indole-7-carbaldehyde derivative afforded a six-membered-ring *N*-OBn amine, identified as pyrrolo­[3,2,1-ij]­quinolinone **2j**, in moderate yield and high enantioselectivity. In contrast, pyrrole-based substrates **2k** and **2l** proved entirely unreactive, affording only recovered starting material. This stark difference in reactivity likely arises from the electronic properties of the heterocyclic core. We hypothesize that the enhanced π-donating character of pyrrole leads to excessive stabilization of the Breslow intermediate, diminishing its nucleophilicity to a point where the subsequent intramolecular attack on the weakly electrophilic oxime is no longer kinetically feasible. Moreover, the electronic nature of the substituent adjacent to the oxime carbon was found to be indispensable for reactivity. When the ester group was replaced by a phenyl group, which lacks comparable electron-withdrawing capabilities, the desired product **2m** was not detected. This underscores the crucial role of the ester in activating the C=N bond. This activation is likely 2-fold: (i) a strong inductive and mesomeric effect, which significantly enhances the electrophilicity of the oxime carbon, and (ii) the ability of the ester carbonyl to stabilize the developing negative charge in the transition state of the nucleophilic attack. The phenyl group offers no such stabilizing interaction, rendering the nonactivated keto-oxime moiety completely inert toward the NHC-generated nucleophile.

The substrate scope was also evaluated with respect to various *O*-alkyl-substituted oximes (Scheme 1). Notably, a range of electron-donating and electron-withdrawing substituents on the *O*-benzyl moiety proved highly efficient in this NHC-catalyzed enantioselective hydroxylamine formation. In general, *O*-benzyl derivatives afforded high stereoselectivities (over 90% ee). However, the *p*-nitro-substituted *O*-benzyl compound showed reduced reactivity, furnishing the target hydroxylamine ether in only 26% yield. Replacing the benzyl group with either a methyl or *tert*-butyl substituent caused a drastic decrease in yield while maintaining excellent enantioselectivity. Furthermore, the use of a THP-protected oxime led to the desired protected hydroxylamine **2s** as a 1:1 mixture of diastereoisomers, each displaying very high enantiomeric excess (>99% and 95% ee, respectively). The observed trend in yields for different *O*-alkyl groups is counterintuitive from a purely steric perspective. The dramatic drop in reactivity for both methyl and *tert*-butyl groups, compared to the efficient benzyl and THP-protected analogues, suggests that productive noncovalent interactions play a crucial role in stabilizing the key cyclization transition state. We hypothesize that the aromatic ring of the *O*-benzyl group engages in stabilizing π-stacking or C–H···π interactions with the catalyst or substrate framework. Similarly, the Lewis basic oxygen atoms of the THP group may act as hydrogen bond acceptors for the Breslow intermediate’s hydroxyl group. In contrast, the noninteracting aliphatic methyl and *tert*-butyl groups offer no such stabilization, leading to a higher activation barrier and consequently, lower yields.

To further extend the applicability of the reaction beyond indole-2-carbaldehyde-based α-oxime esters, we investigated a system designed to construct chiral hydroxylamine ethers with six-membered ring closure. Such an approach significantly expands the methodology by enabling the synthesis of ring systems of different sizes. In this vein, salicylaldehyde was selected as a model substrate to explore the formation of six. As shown in Scheme 2, a wide array of salicylaldehyde-derived *O*-benzyl oxime esters were tested under the optimized reaction conditions, furnishing chromanone-type products in very high yield and with excellent enantioselectivities. The influence of substituent electronic properties and positioning on the aromatic ring was examined in detail. Substrates bearing electron-donating, neutral, or electron-withdrawing groups at the 4-position (**4a**–**4e**) all displayed outstanding reactivity, enabling the formation of six-membered chromanone scaffolds bearing an *N*-benzyloxyamine moiety in consistently high yield and >99% ee. Likewise, the 5-methoxy- and 5-methyl-substituted salicylaldehyde-derived oxime esters afforded products **4f** and **4g**, respectively, in excellent yields (99%). While **4g** showed 99% ee, the enantiomeric excess of **4f** could not be determined, despite screening multiple chiral stationary phases by HPLC, likely due to coelution or insufficient resolution under the tested conditions. Similar trends were observed for electron-deficient substituents (**4h**, **4i**), which provided nearly quantitative yields and exceptional optical purity. Moreover, we investigated the effect of 6-position substituents on the annulation process, given the potential influence of *ortho*-oriented activating or deactivating groups. Both electron-withdrawing (**4k**, **4l**) and electron-donating (**4j**) functionalities were well tolerated, affording the corresponding *N*-benzyloxyamine-derived chromanones in excellent yields and enantioselectivities in all cases. Substitution at the 3-position (**4m**) similarly furnished the desired chromanone-type hydroxylamine with comparably high performance. Furthermore, a naphthaldehyde-derived α-oxime ester (**3n**) readily underwent cyclization to form the tricyclic naphthoannulated pyranone **4n** in a highly stereoselective manner, albeit in somewhat lower yield. Overall, these findings demonstrate that the reaction is not significantly influenced by either the position or electronic nature of substituents on the aromatic ring, consistently providing excellent yields and stereocontrol. Finally, the *O*-THP-protected α-oxime ester was also compatible under these conditions, providing the *O*-THP hydroxylamine product **4o** in quantitative yield as a 1:1 mixture of diastereomers—both of which exhibited 99% ee (Scheme 2). Notably, mild acidic conditions readily cleave the *O*-THP protecting group, furnishing the free hydroxylamine without erosion of enantioselectivity. This deprotection step underscores the synthetic utility of our protocol, as the liberated hydroxylamine can be further transformed, for instance, into hydroxamic acids or other valuable derivatives. (Scheme 3).

**Scheme 2:**
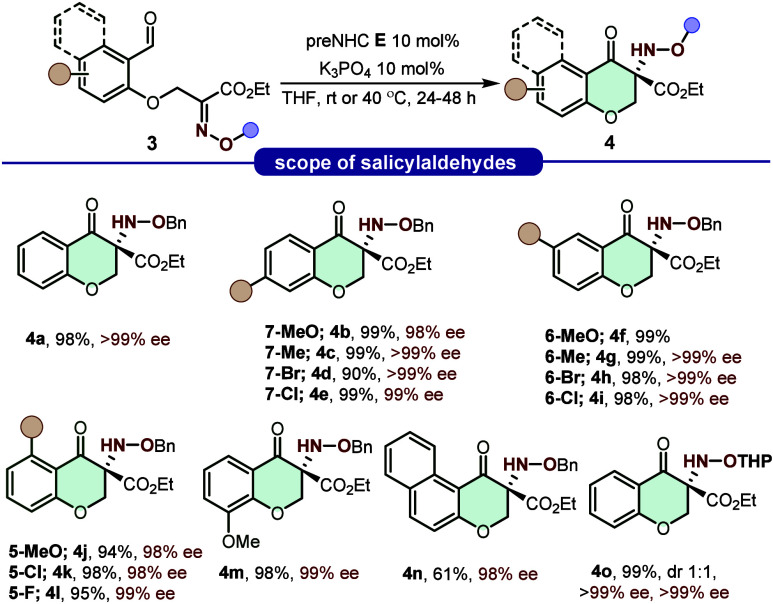
Scope of Salicylaldehyde-Derived Ether Oximes^a^

a Reaction conditions: **1** (0.1 mmol), NHC·HBF_4_ **E** (10 mol %), K_3_PO_4_ (10 mol %) in THF (1.0 mL) at rt or 40 °C, and 24–48 h. Given are yields of the column chromatography-purified products. *ee* values determined by HPLC on chiral stationary phase.

**Scheme 3:**
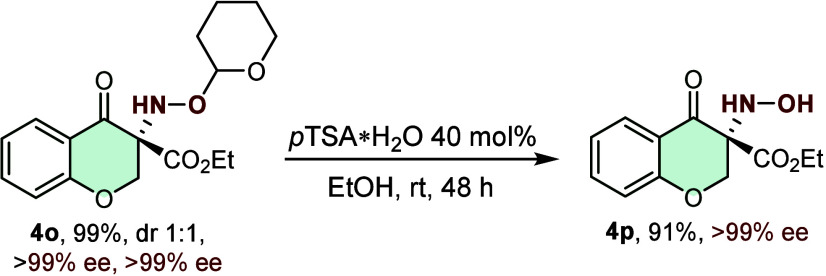
Deprotection of *O*-THP for Hydroxylamine Moiety Formation

In summary, we have developed a highly efficient and enantioselective NHC-catalyzed intramolecular annulation of α-oxime esters. The reaction proved highly versatile, tolerating a broad range of substituents on both indole- and salicylaldehyde-derived substrates, affording access to valuable five- and six-membered *N*-alkoxyamines. This protocol is characterized by its operational simplicity, scalability, and consistently excellent yields and enantioselectivities (often >99% ee). Beyond its clear synthetic utility, this work provides several key mechanistic insights into the reactivity of this challenging substrate class. We have demonstrated the indispensable role of an adjacent ester group in activating the otherwise inert keto-oxime C=N bond and uncovered the high sensitivity of the reaction to the electronics of the Breslow intermediate. Significantly, we have also shown that the reaction’s success is dictated not by simple sterics but by productive noncovalent interactions that stabilize the key cyclization transition state. This work thus represents a valuable contribution to the field of NHC-catalyzed polarity-reversal reactions, enabling the assembly of diverse chiral N–O architectures. We believe this deeper understanding will inspire further exploration of electronically challenging substrates in organocatalysis.

## Supplementary Material



## Data Availability

The data underlying this study are available in the published article and its Supporting Information.

## Notes

Details on experimental procedures, characterization, crystal structure (**2d**), NMR spectra and HPLC data of all spectra of new chiral five- and six-membered *N*-alkoxyamine derivatives (PDF)
