# Supplementary material for: Enantioselective NHC-Catalyzed Annulation via Umpolung for the Construction of Hydroxylamine Architectures with Variable Ring Sizes
Source: Org Lett. 2025 Aug 18;27(34):9364–9. doi: 10.1021/acs.orglett.5c02576 (PMC12400417; doi:10.1021/acs.orglett.5c02576)
Supplement: Supplementary file 1 [file ol5c02576_si_001.pdf]

Supporting Information

# Enantioselective NHC-Catalyzed Annulation via Umpolung for the Construction of Hydroxylamine Architectures with Variable Ring Sizes

Izabela Barańska, Monika Radosińska, Liliana Dobrzańska, Krzysztof Dzieszkowski, Zbigniew Rafiński*

Nicolaus Copernicus University in Torun
Faculty of Chemistry
7 Gagarin Street, 87-100 Torun, Poland
*Correspondence: payudo@chem.umk.pl

## List of contents

1. General Methods……………………………………………………………..S2
2. Optimization Studies…………………………………………………………S3
3. Synthetic Procedures, Stereochemical Model and Proposed Mechanism …...………S4
4. X-Ray Crystallography data…………………………………………………...S41
5. NMR Spectra…………………………………………………………………..S43
6. HPLC Chromatograms………………………………………………………….S113
7. References……………………………………………………………………….S147

# 1. General Methods

Presented reactions were carried out in dry glassware under an inert atmosphere of argon. All reagents were purchased from Sigma Aldrich and Fluorochem and were used without further purification. Selected reactions were monitored by using thin-layer chromatography (TLC), which was visualized under a UV lamp (254 nm). All solvents were purchased from Honeywell. Anhydrous solvents were prepared using an INERT PureSolv Solvent Purification System. Purification of selected products was performed by column chromatography using CombiFlash Rf+ Lumen system with UV-VIS and ELSD detectors.

NMR spectra were recorded on Bruker AMX 400 [400 MHz ($^1$H)] and Bruker AMX 700 [700 MHz ($^1$H)] spectrometers using $CDCl_3$ or DMSO-$d_6$ as solvents and were reported in ppm relative to $CHCl_3$ residual peak (δ 7.24) or DMSO-$d_5$ residual peak (2.50 ppm) for $^1$H NMR and relative to the central $CDCl_3$ (δ 77.23) or DMSO-$d_6$ (39.5 ppm) resonance for $^{13}$C NMR. Coupling constants (*J*) are given in Hz.

Infrared spectra were measured on an Alpha FT-IR spectrometer from Bruker with ATR module. Mass spectra were recorded on an Agilent 6530 Q-TOF LC/MS system coupled with a 1290 Infinity II liquid chromatograph. The melting points of obtained products were measured on a Stuart SMP50 Melting Point automatic apparatus. The specific rotation of products was measure with Bellingham + Stanley ADP430 Polarimeter. The enantiomeric excess of chiral products was determined using HPLC Agilent Technologies 1200 Series and chiral stationary phases: Phenomenex Lux Cellulose-1 (3 μm) and Phenomenex Lux Amylose-1 (3 μm).

The triazolium salts were obtained according to the following procedures: **A** [1], **B** [2], **C**, **D** [3], **E** [4].

## 2. Optimization studies[a]

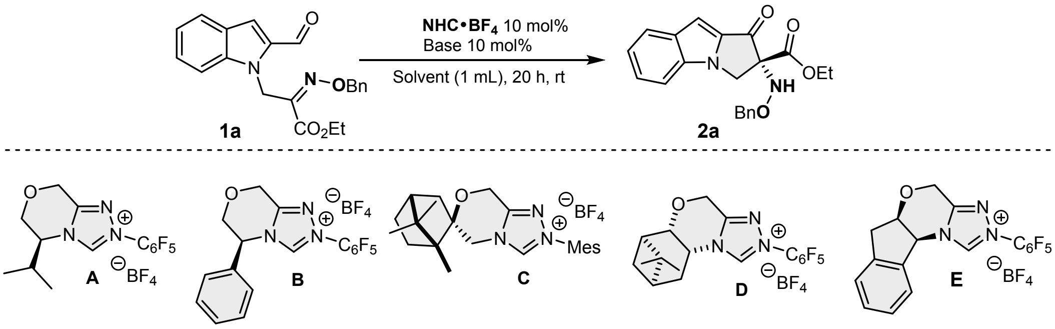


| Entry | NHC•HBF$_4$ | Base | Solvent | Yield (%)[b] | Ee of 2a (%)[c] |
|---|---|---|---|---|---|
| 1 | **A** | $K_3PO_4$ | THF | 22 | 66 |
| 2 | **B** | $K_3PO_4$ | THF | 61 | 90 |
| 3 | **C** | $K_3PO_4$ | THF | 75 | 12 |
| 4 | **D** | $K_3PO_4$ | THF | 5 | --- |
| 5 | **E** | $K_3PO_4$ | THF | 99 | 96 |
| 6 | **E** | $K_3PO_4$ | Toluene | 74 | 96 |
| 7 | **E** | $K_3PO_4$ | DCM | 99 | 92 |
| 8 | **E** | $K_3PO_4$ | $C_6H_5F$ | 99 | 92 |
| 9 | **E** | $K_3PO_4$ | $Et_2O$ | 85 | 96 |
| 10 | **E** | $K_3PO_4$ | CPME | 71 | 97 |
| 11 | **E** | $K_3PO_4$ | *m*-Xylene | 89 | 98 |
| 12[d] | **E** | $K_3PO_4$ | THF | 78 | 98 |
| 13 | **E** | $Cs_3CO_3$ | THF | 89 | 98 |
| 14 | **E** | DIPEA | THF | 53 | 97 |
| 15 | **E** | DABCO | THF | --- | --- |
| 16 | **E** | DBU | THF | 11 | 68 |

[a]The reaction conditions were carried out using: **1a** (0.10 mmol), NHC•HBF$_4$ catalyst **A-E** (10 mol%), base (10 mol%) and solvent (1.0 mL) at room temperature for 24 h. [b]The 1H NMR yield of a crude product was determined with the aid of 1,1,2,2-tetrachloroethane as an internal standard. [c]The HPLC analysis on a chiral stationary phase was used for determining er. [d]5 mol% of NHC•HBF$_4$ catalyst **E** was used.

## 3. Synthetic procedures

### 3.1.Indole-2-carboxylic acids reduction procedure (GP 1):

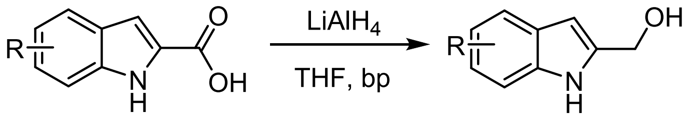


**5**

The dry THF (100 mL) and $LiAlH_4$ (124 mmol; 2.0 eq) were introduced to the two-neck flask equipped with a reflux condenser and inert gas inlet. After the hydride was dissolved, the mixture was cooled to 0°C, and the indole-2-carboxylic acid (62 mmol; 1.0 eq) was added portion-wise. The reaction was carried out for 24 h at 66°C using oil bath. The mixture was then cooled to 0°C following the slow addition of water and 15% NaOH solution. Subsequently, THF was evaporated, and the water phase was extracted with $Et_2O$. The combined organic extracts were washed with brine and dried with anhydrous $MgSO_4$. After filtering off the $MgSO_4$, the solvents were evaporated and the crude was purified by flash column chromatography (MeOH:DCM 1:99%) to give the pure compound **5**.

### 3.2.2-(Hydroxymethyl)indoles oxidation procedure (GP 2):

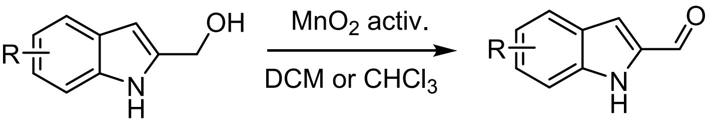


**5** **6**

To a solution of 2-(hydroxymethyl)indole (**5**) (42.6 mmol; 1.0 eq) in chloroform (160 mL), active manganese (IV) oxide (170.3 mmol; 4.0 eq) was added. The resulting suspension was heated to reflux in an oil bath for 24 h. After cooling to room temperature, the mixture was filtered through a pad of Celite. The filtrate was concentrated, and the residue was purified by flash column chromatography to afford the final aldehyde (**6**).

## 3.3.General Procedures for the synthesis of oxime ethers (GP 3)

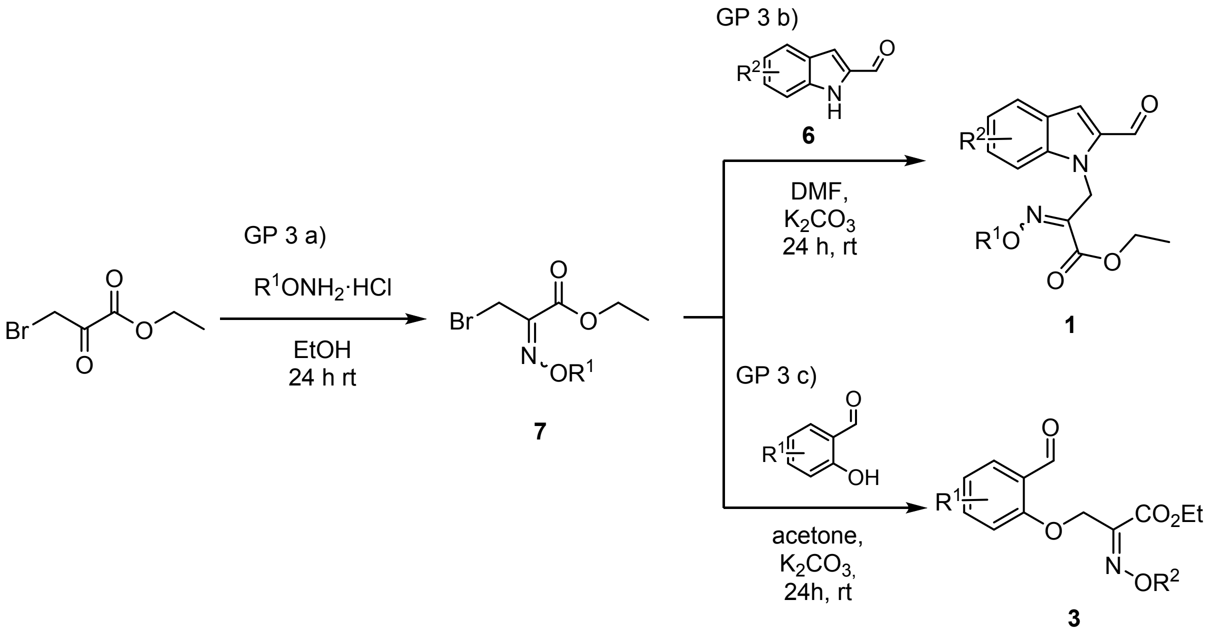


**GP 3 a)** The hydroxylamine or hydroxylamine hydrochloride (15.4 mmol; 1.5 eq) was introduced to the solution of ethyl bromopyruvate in ethanol (31 mL). The reaction was carried out for 24 h at room temperature. Next, the ethanol was evaporated, and the residue was dissolved in $Et_2O$ and water. After the separation of the phases, the water phase was extracted with $Et_2O$. The combined organic extracts were washed with brine and dried with anhydrous $MgSO_4$. Subsequent filtering of $MgSO_4$ and evaporation of the solvent allowed obtaining the final product (**7**) as a mixture of E/Z isomers. The oxime ether (**7**) was used in the next step without further purification.

*All oxime ethers were obtained according to the procedure described above, but methanol was used as a solvent for the synthesis of **2t** and **2u**.*

**GP 3 b)** To the solution of aldehyde (**6**) in DMF (3 mL) $K_2CO_3$ (3.11 mmol; 3.0 eq) and oxime ether obtained in the previous step (**7**) (1.14 mmol, 1.0 eq) dissolved in 3 mL of DMF were added. The reaction was further conducted overnight at room temperature. Then, water was added and the mixture was extracted with ethyl acetate. The combined organic extracts were washed with brine and dried with $MgSO_4$. After filtration of the $MgSO_4$, the crude was purified by flash column chromatography (hexane/EtOAc gradient 0 → 20% EtOAc) to give the final pure product **1**.

**GP 3 c)** To the solution of aldehyde (**6**) (1.14 mmol; 1.0 eq) in acetone (1 mL) $K_2CO_3$ (1.14 mmol; 1.0 eq) and oxime ether obtained in the previous step (**7**) (0.20 g, 1.14 mmol, 1.0 eq) dissolved in 1 mL of acetone (1.14 mmol, 1.0 eq) were added. The reaction was further conducted overnight at room temperature. Then, acetone was evaporated, and the residue was dissolved in water and ethyl acetate.

The phases were separated and the water phase was extracted with ethyl acetate. The combined organic extracts were washed with brine and dried with $MgSO_4$. After filtration off the $MgSO_4$, the crude was purified by flash column chromatography (hexane/EtOAc gradient 0 → 20% EtOAc) to give the final pure product **3**.

### 3.4. Enantioselective NHC-Catalyzed Annulative Synthesis of hydroxylamines (GP 4):

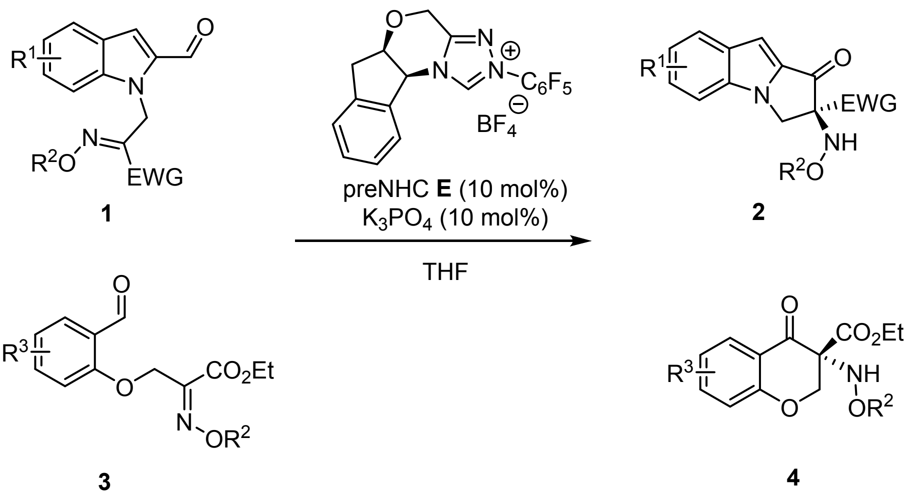


The flask was charged with precatalyst **E** (0.013 mmol; 0.1 eq), $K_3PO_4$ (0.013 mmol; 0.1 eq), and dry THF (1.3 mL). Then, the substrate **1** or **3** (0.13 mmol; 1.0 eq) was introduced in one portion. The progress of the reaction was monitored using the TLC method (the reaction temperature and time are given below with a description of the analytical data for a specific compound). The solvent was evaporated, and the residue was dissolved in $Et_2O$ and filtered through a syringe filter 0.45 µm. After evaporation of the $Et_2O$, the pure product **2** or **4** was obtained. If necessary, the product was additionally purified using flash column chromatography. In this case, information about this is provided below with the yield of the specific product.

### 3.5. THP-Deprotection Procedure (GP 5)

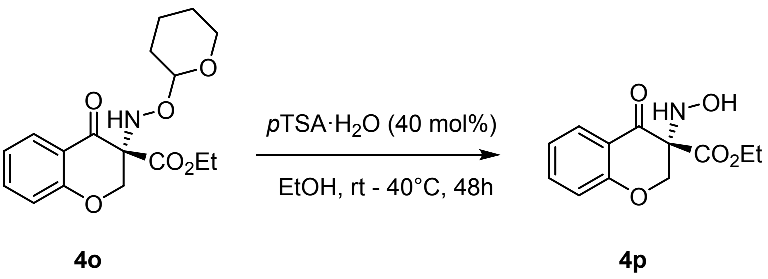


**4o** **4p**

To a solution of the protected alkoxylamine (**4o**) (200 mg, 0.60 mmol, 1.0 eq) in ethanol (5.6 mL) was added p-toluenesulfonic acid monohydrate (22.7 mg, 0.12 mmol, 0.2 eq). The mixture was stirred at room temperature for 24 h. Subsequently, an additional portion of the acid (22.7 mg, 0.12 mmol, 0.2 eq) was added, and the reaction mixture was heated to 40 °C in an oil bath for another 24 h. The solvent was removed under reduced pressure, and the crude residue was purified by flash column chromatography (eluting with a gradient of 0% to 20% EtOAc in hexane) to afford compound **4p** as a pure solid (137 mg, 91% yield).

### 3.6. Synthesis of compound 2a (1.0 mmol scale)

A flask was charged with precatalyst **E** (46.7 mg, 0.10 mmol, 0.1 eq), $K_3PO_4$ (21.2 mg, 0.10 mmol, 0.1 eq), and substrate **1a** (364.4 mg, 1.0 mmol, 1.0 eq). Dry THF (10 mL) was then added, and the resulting mixture was stirred at room temperature for 24 h. After completion, the solvent was removed under reduced pressure, and the crude residue was purified by flash column chromatography (eluting with a gradient of 0% to 20% EtOAc in hexane) to afford compound **2a** (328 mg, 90% yield) with an enantiomeric excess of 96% ee.

### 3.7. Procedure for the synthesis of 1j (GP 6)

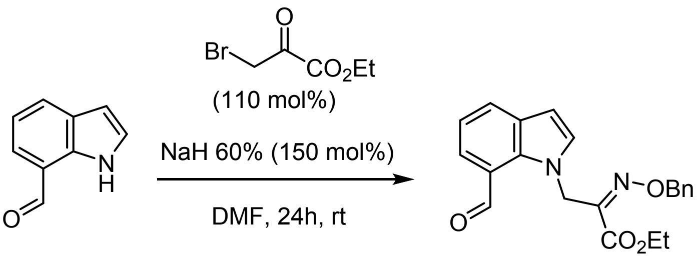


**1j**

The indolo-7-carbaldehyde (0.3 g; 2.07 mmol; 1.0 eq) was introduced into the flask and dissolved in 3 mL of DMF. Then, NaH 60% (3.1 mmol; 1.5 eq) was added and mixing was continued for 10 minutes at room temperature. Next, ethyl bromopyruvate (2.28 mmol; 1.1 eq) dissolved in DMF (3 mL) was dropped into the mixture. The reaction was carried out for 24 hours at room temperature. Then, water and ethyl acetate were added. The phases were separated and the water phase was extracted with ethyl acetate. The combined organic extracts were washed with brine and dried with anhydrous $MgSO_4$. After filtering off the $MgSO_4$, the crude was purified by flash column chromatography to give pure **1j** (0.16 g; 0.43 mmol; yield: 21%).

## 3.8. Mechanistic Discussion and Origin of Enantioselectivity

The catalytic cycle commences with the deprotonation of the triazolium salt **E** to generate the free *N*-heterocyclic carbene. The carbene then attacks the aldehyde substrate, forming a tetrahedral intermediate **I**. Subsequent proton transfer yields intermediate **II**, which upon deprotonation at the former aldehydic carbon, affords the key resonance-stabilized Breslow intermediate (**III**), achieving the desired polarity reversal. The nucleophilic Breslow intermediate then undergoes an intramolecular addition to the C=N bond of the oxime ether, forming intermediate **IV**. Finally, collapse of this intermediate regenerates the NHC catalyst and releases the final product **2a**, closing the catalytic cycle.

To rationalize the observed stereochemical outcome, a plausible transition-state model is proposed, consistent with previous studies on aminoindanol-derived NHC catalysts and our X-ray crystallographic data for product (*R*)-**2d**. The Breslow intermediate is known to exist predominantly as the (*E*)-isomer, which minimizes the number of competing diastereomeric transition states. In the favored transition state **TS-A**, the substrate adopts a conformation that allows for a stabilizing intramolecular hydrogen bond between the hydroxyl group of the Breslow intermediate and the ester carbonyl oxygen. This pre-organizes the molecule for cyclization. The chiral environment of the catalyst then directs the nucleophilic attack onto the *Si*-face of the oxime. This arrangement is sterically favored, as the alternative approach (**TS-B**), leading to the (*S*)-enantiomer, would induce significant steric repulsion between the bulky *O*-benzyl group of the oxime and the pentafluorophenyl ($C_6F_5$) moiety of the catalyst scaffold. Consequently, the lower-energy pathway through **TS-A** predominates, yielding the (*R*)-configured product with high enantioselectivity.

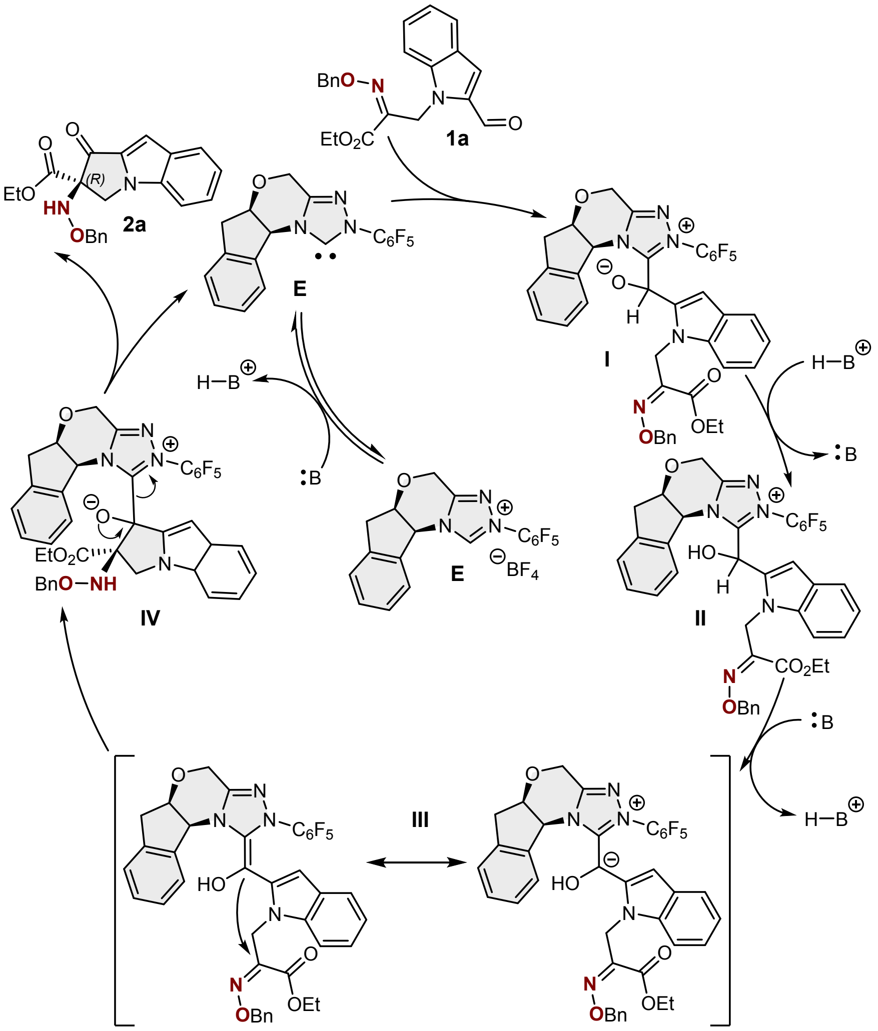
1a
BnO
N
EtO2C
I
H–B⊕
:B
II
CO2Et
OBn
III
IV
BnO–NH
EtO2C
E
BF4
H–B⊕
:B
2a
(R)
HN
OBn
EtO
C6F5

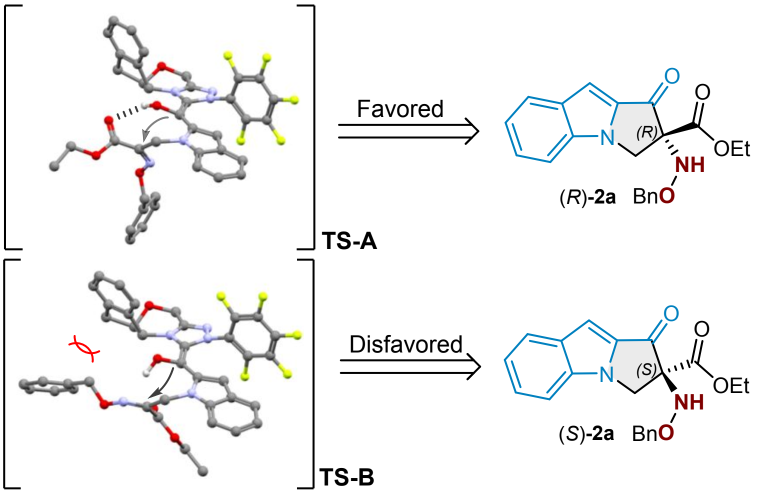
TS-A
Favored
(R)
OEt
NH
(R)-2a BnO
TS-B
Disfavored
(S)
OEt
NH
(S)-2a BnO

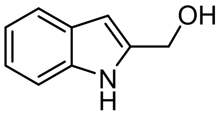


*(1H-indol-2-yl)methanol* (**5a**). The synthesis was carried out according to **GP 1**. A scale of 62 mmol, orange solid, 6.03 g (41 mmol), isolated yield of 61%. **$^1$H NMR** (400 MHz, $CDCl_3$) δ 8.37 (br. s, 1H), 7.58 (d, *J* = 7.9 Hz, 1H), 7.33 (d, *J* = 8.2 Hz, 1H), 7.20 - 7.16 (m, 1H), 7.13 - 7.09 (m, 2H), 6.40 (s, 1H), 4.80 (s, 2H). The above analysis results correspond to the literature data. [5]

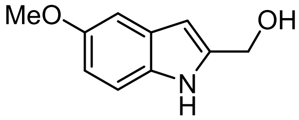


*(5-methoxy-1H-indol-2-yl)methanol* (**5b**). The synthesis was carried out according to according to **GP 1**. A scale of 10.5 mmol, yellow solid, 1.41 g (7.95 mmol), isolated yield of 76%. **$^1$H NMR** (400 MHz, $CDCl_3$) δ 8.22 (br. s, 1H), 7.23 (d, *J* = 8.8 Hz, 1H), 7.04 (d, *J* = 2.4 Hz, 1H), 6.85 (dd, *J* = 8.8, 2.6 Hz, 1H), 6.34 (dd, *J* = 2.1, 0.9 Hz, 1H), 4.80 (s, 2H), 3.84 (s, 3H). The above analysis results correspond to the literature data. [6]

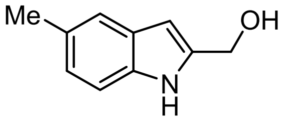


*(5-methyl-1H-indol-2-yl)methanol* (**5c**). The synthesis was carried out according to according to **GP 1**. A scale of 11.04 mmol, yellow solid, 1.60 g (9.93 mmol), isolated yield of 87%. **$^1$H NMR** (400 MHz, $CDCl_3$) δ 8.24 (br. s, 1H), 7.37 (d, *J* = 0.9 Hz, 1H), 7.22 (d, *J* = 8.5 Hz, 1H), 7.03 - 7.00 (m, 1H), 6.32 (d, *J* = 1.2 Hz, 1H), 4.79 (s, 2H), 2.44 (s, 3H). The above analysis results correspond to the literature data. [5]

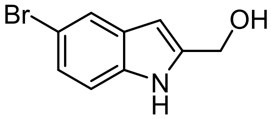


*(5-bromo-1H-indol-2-yl)methanol* (**5d**). The synthesis was carried out according to **GP 1**. A scale of 8.33 mmol, yellow solid, 0.69 g (3.05 mmol), isolated yield of 37%. **$^1$H NMR** (400 MHz, $CDCl_3$) δ 8.40 (br. s, 1H), 7.70 - 7.69 (m, 1H), 7.27 - 7.21 (m, 2H), 6.34 (d, *J* = 1.5 Hz, 1H), 4.84 (s, 2H). The above analysis results correspond to the literature data. [7]

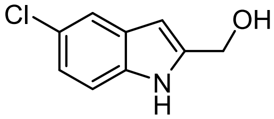


*(5-chloro-1H-indol-2-yl)methanol* (**5e**). The synthesis was carried out according to **GP 1**. A scale of 10.22 mmol, beige solid, 0.70 g (3.85 mmol), isolated yield of 38%. **$^1$H NMR** (700 MHz, DMSO-$d_6$) δ 11.21 (s, 1H), 7.49 (d, *J* = 2.2 Hz, 1H), 7.33 (d, *J* = 8.6 Hz, 1H), 7.02 (dd, *J* = 8.2, 2.2 Hz, 1H), 6.27 (dd, *J* = 2.2, 0.9 Hz, 1H), 5.34 (t, *J* = 5.6 Hz, 1H), 4.60 (d, *J* = 5.2 Hz, 2H). The above analysis results correspond to the literature data. [8]

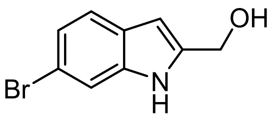 *(6-bromo-1H-indol-2-yl)methanol* (**5f**). The synthesis was carried out according to **GP 1**. A scale of 8.33 mmol, yellow solid, 1.49 g (6.59 mmol), isolated yield of 79%. **$^1$H NMR** (400 MHz, $CDCl_3$) δ 8.35 (s, 1H), 7.51 - 7.50 (m, 1H), 7.43 (d, *J* = 8.5 Hz, 1H), 7.20 (dd, *J* = 8.5, 1.8 Hz, 1H), 6.38 - 6.37 (m, 1H), 4.83 (d, *J* = 0.6 Hz, 2H). The above analysis results correspond to the literature data. [9]

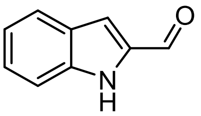 *1H-indole-2-carbaldehyde* (**6a**). The synthesis was carried out according to **GP 2**. A scale of 42.57 mmol, yellow solid, 4.47 g (30.79 mmol), isolated yield of 72%. **$^1$H NMR** (400 MHz, $CDCl_3$) δ 9.86 (s, 1H), 9.14 (br. s, 1H), 7.76 (dq, *J* = 8.2, 0.9, 1H), 7.47 (dq, *J* = 8.5, 0.8 Hz, 1H), 7.41 (ddd, *J* = 8.5, 6.8, 1.2 Hz, 1H), 7.29 (dd, *J* = 2.1, 0.9 Hz, 1H), 7.19 (ddd, *J* = 7.9, 6.8, 1.2 Hz, 1H). The above analysis results correspond to the literature data. [5]

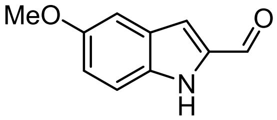 *5-methoxy-1H-indole-2-carbaldehyde* (**6b**). The synthesis was carried out according to **GP 2**. A scale of 6.77 mmol, yellow solid, 0.82 g (4.68 mmol), isolated yield of 69%. **$^1$H NMR** (400 MHz, $CDCl_3$) δ 9.81 (s, 1H), 9.01 (br. s, 1H), 7.36 - 7.34 (m, 1H), 7.19 (dd, *J* = 2.1, 0.9 Hz, 1H), 7.11 (d, *J* = 2.6 Hz, 1H), 7.09 - 7.06 (m, 1H), 3.86 (s, 3H). The above analysis results correspond to the literature data. [6]

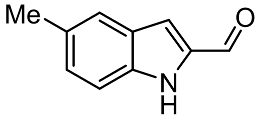 *5-methyl-1H-indole-2-carbaldehyde* (**6c**). The synthesis was carried out according to **GP 2**. A scale of 9.93 mmol, pink solid, 0.82 g (5.09 mmol), isolated yield of 52%. **$^1$H NMR** (400 MHz, $CDCl_3$) δ 9.84 (s, 1H), 9.00 (s, 1H), 7.52 - 7.51 (m, 1H), 7.36 (d, *J* = 8.4 Hz, 1H), 7.23 (dd, *J* = 8.4, 1.6 Hz, 1H), 7.18 (dd, *J* = 2.0, 1.0 Hz, 1H), 2.45 (s, 3H). The above analysis results correspond to the literature data. [5]

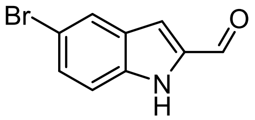 *5-bromo-1H-indole-2-carbaldehyde* (**6d**). The synthesis was carried out according to **GP 2**. A scale of 3.05 mmol, beige solid, 0.21 g (0.94 mmol), isolated yield of 31%. $^1$H NMR (400 MHz, $CDCl_3$) δ 9.85 (s, 1H), 8.94 (br. s, 1H), 7.90 (m, 1H), 7.48 (dd, *J* = 8.8, 1.8 Hz, 1H), 7.33 (d, *J* = 8.8 Hz, 1H), 7.20 (dd, *J* = 2.1, 0.9 Hz, 1H). The above analysis results correspond to the literature data. [10]

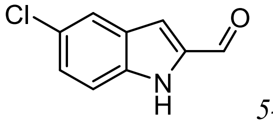 *5-chloro-1H-indole-2-carbaldehyde* (**6e**). The synthesis was carried out according to **GP 2**. A scale of 3.85 mmol, beige solid, 0.10 g (0.56 mmol), isolated yield of 15%. $^1$H NMR (400 MHz, $CDCl_3$) δ 9.85 (s, 1H), 9.11 (br. s, 1H), 7.73 (m, 1H), 7.39 (d, $J$ = 8.8 Hz, 1H), 7.36 - 7.33 (m, 1H), 7.21 (dd, $J$ = 2.1, 0.9 Hz, 1H). The above analysis results correspond to the literature data. [5]

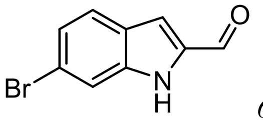 *6-bromo-1H-indole-2-carbaldehyde* (**6f**). The synthesis was carried out according to **GP 2**. Solvent: $CH_3CN$, A scale of 3.30 mmol, orange solid, 0.35 g (1.56 mmol), isolated yield of 47%. $^1$H NMR (400 MHz, $CDCl_3$) δ 9.86 (s, 1H), 9.14 (br. s, 1H), 7.64 (s, 1H), 7.63 - 7 .60 (m, 1H), 7.29 (dd, $J$ = 8.5, 1.6, 1H), 7.25 (m, 1H). The above analysis results correspond to the literature data. [9]

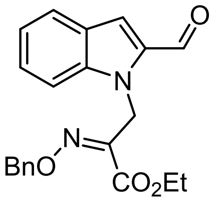 *ethyl (Z)-2-((benzyloxy)imino)-3-(2-formyl-1H-indol-1-yl)propanoate* (**1a**). The synthesis was carried out according to **GP 3 b)**. A scale of 8.03 mmol, yellow solid, 2.32 g (6.37 mmol), isolated yield of 79%. ; **$^1$H NMR** (400 MHz, $CDCl_3$) δ 9.76 (s, 1H), 7.68 (dt, $J$ = 8.0, 1.0 Hz, 1H), 7.35 - 7.33 (m, 3H), 7.26 - 7.23 (m, 4H), 7.18 (d, $J$ = 0.6 Hz, 1H), 7.14 (ddd, $J$ = 7.9, 6.2, 2.1 Hz, 1H), 5.78 (s, 2H), 5.27 (s, 2H), 4.15 (q, $J$ = 7.0 Hz, 2H), 1.15 (t, $J$ = 7.2 Hz, 3H). **$^{13}$C{$^1$H} NMR** (75.5 MHz, $CDCl_3$) δ 182.6, 162.3, 148.1, 140.4, 135.8, 128.8, 128.5, 127.1, 126.3, 123.4, 121.3, 118.1, 110.7, 78.5, 61.9, 39.2, 13.9. **IR $\nu_{max}$**: 3018, 2981, 2821, 2733, 1715, 1674, 1614, 1518, 1464, 1415, 1371, 1321, 1256, 1238, 1169, 1145, 1127, 1111, 1024, 1004, 980, 926, 847, 812, 798, 750, 735, 697 cm$^{-1}$. **mp** 58.5 - 60.9 °C. **HRMS** (ESI Q-TOF) m/z: [(M+H)$^+$] calcd. for $C_{21}H_{21}N_2O_4$ 365.1501 found: 365.1496.

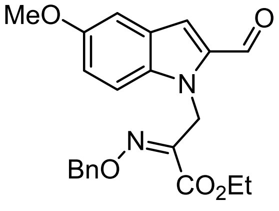 *ethyl (Z)-2-((benzyloxy)imino)-3-(2-formyl-5-methoxy-1H-indol-1-yl)propanoate* (**1b**). The synthesis was carried out according to **GP 3 b)**. A scale of 1.14 mmol, white solid, 0.18 g (0.46 mmol), isolated yield of 41%. ; **$^1$H NMR** (400 MHz, $CDCl_3$) δ 9.72 (s, 1H), 7.35 - 7.33 (m, 3H), 7.26 - 7.23 (m, 2H), 7.12 - 7.10 (m, 1H), 7.09 (d, $J$ = 0.9 Hz, 1H), 7.04 (d, $J$ = 2.4 Hz, 1H), 6.90 (dd, $J$ = 9.1, 2.6 Hz, 1H), 5.73 (s, 2H), 5.26 (s, 2H), 4.15 (q, $J$ = 7.0 H, 2H), 3.84 (s, 3H), 1.16 (t, $J$ = 7.0 Hz, 3H). **$^{13}$C{$^1$H} NMR** (75.5 MHz, $CDCl_3$) δ 182.4, 162.3, 155.0, 148.1, 136.0, 136.0, 135.8, 128.8, 128.5, 126.6, 118.9, 117.2, 111.7, 102.8, 78.5, 61.9, 55.7, 39.2, 13.9. **IR $\nu_{max}$**: 3054, 2977, 2952,

2836, 1747, 1663, 1593, 1518, 1477, 1451, 1339, 1295, 1254, 1241, 1210, 1176, 1164, 1146, 1022, 985, 928, 844, 804, 759, 745, 723, 701, 690, 602, 527 $cm^{-1}$. **mp** 93.5 - 94.1 °C. **HRMS** (ESI Q-TOF) m/z: $[(M+H)^+]$ calcd. for $C_{22}H_{23}N_2O_5$ 395.1607 found: 395.1603

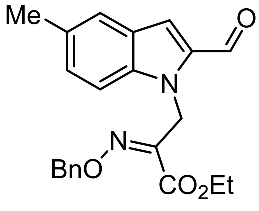


*ethyl (Z)-2-((benzyloxy)imino)-3-(2-formyl-5-methyl-1H-indol-1-yl)propanoate* (**1c**). The synthesis was carried out according to **GP 3 b)**. A scale of 1.88 mmol, yellow solid, 0.45 g (1.19 mmol), isolated yield of 63%. ; **$^1$H NMR** (400 MHz, $CDCl_3$) δ 9.72 (s, 1H), 7.45 - 7.44 (m, 1H), 7.35 - 7.32 (m, 3H), 7.24 - 7.22 (m, 2H), 7.13 - 7.06 (m, 3H), 5.74 (s, 2H), 5.25 (s, 2H), 4.15 (q, $J$ = 7.0 Hz, 2H), 2.41 (s, 3H), 1.16 (t, $J$ = 7.0 Hz, 3H). **$^{13}$C{$^1$H} NMR** (75.5 MHz, $CDCl_3$) δ 182.5, 162.3, 148.2, 139.0, 135.8, 130.6, 129.2, 128.8, 128.5, 128.5, 126.6, 122.5, 117.6, 110.4, 78.5, 61.9, 39.2, 21.4, 14.0. **IR $v_{max}$**: 3015, 2981, 2938, 2909, 2856, 2838, 1718, 1675, 1524, 1477, 1413, 1369, 1322, 1258, 1241, 1166, 1147, 1117, 979, 851, 802, 754, 722, 695, 603 $cm^{-1}$. **mp** 72.0 - 72.7 °C. **HRMS** (ESI Q-TOF) m/z: $[(M+H)^+]$ calcd. for $C_{22}H_{23}N_2O_4$ 379.1658 found: 379.1655.

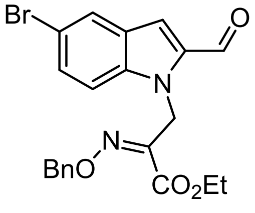


*ethyl (Z)-2-((benzyloxy)imino)-3-(5-bromo-2-formyl-1H-indol-1-yl)propanoate* (**1d**). The synthesis was carried out according to **GP 3 b)**. A scale of 0.94 mmol, white solid, 0.30 g (0.67 mmol), isolated yield of 71%. ; **$^1$H NMR** (400 MHz, $CDCl_3$) δ 9.74 (s, 1H), 7.81 - 7.80 (m, 1H), 7.37 - 7.31 (m, 3H), 7.26 - 7.24 (m, 1H), 7.19 - 7.17 (m, 2H), 7.10 - 7.08 (m, 2H), 5.70 (s, 2H), 5.22 (s, 2H), 4.20 (q, $J$ = 7.0 Hz, 2H), 1.21 (t, $J$ = 7.2 Hz, 3H). **$^{13}$C{$^1$H} NMR** (101 MHz, $CDCl_3$) δ 182.5, 162.3, 147.5, 138.9, 136.4, 135.4, 129.8, 128.9, 128.6, 128.5, 127.7, 122.5, 116.6, 114.5, 112.3, 78.7, 62.0, 39.3, 14.0. **IR $v_{max}$**: 3107, 2982, 2947, 2849, 1723, 1679, 1515, 1452, 1415, 1340, 1321, 1252, 1231, 1166, 1135, 1121, 1027, 1006, 866, 850, 821, 801, 755, 698, 655, 609 $cm^{-1}$. **mp** 91.8 - 93.8 °C. **HRMS** (ESI Q-TOF) m/z: $[(M+H)^+]$ calcd. for $C_{21}H_{20}BrN_2O_4$ 443.0601 found: 443.0607.

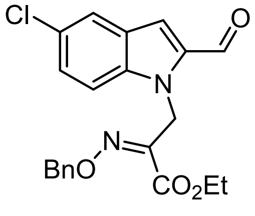


*ethyl (Z)-2-((benzyloxy)imino)-3-(5-chloro-2-formyl-1H-indol-1-yl)propanoate* (**1e**). The synthesis was carried out according to **GP 3 b)**. A scale of 0.56 mmol, white solid, 0.13 g (0.33 mmol), isolated yield of 58%. ; **$^1$H NMR** (400 MHz, $CDCl_3$) δ 9.74 (s, 1H), 7.64 - 7.63 (m, 1H), 7.37 - 7.31 (m, 3H), 7.19 (dd, $J$ = 7.6, 2.0 Hz, 2H), 7.13 (d, $J$ = 1.5 Hz, 2H), 7.11 (s, 1H), 5.71 (s, 2H),

5.22 (s, 2H), 4.20 (q, *J* = 7.0 Hz, 2H), 1.21 (t, *J* = 7.2 Hz, 3H). **$^{13}$C{$^{1}$H} NMR** (101 MHz, $CDCl_3$) δ 182.5, 162.3, 147.5, 138.6, 136.5, 135.5, 128.9, 128.6, 128.5, 127.4, 127.0, 127.0, 122.3, 116.8, 112.0, 78.6, 62.0, 39.3, 13.9. **IR $\nu_{max}$**: 3115, 2981, 2945, 2856, 1721, 1678, 1520, 1465, 1419, 1342, 1321, 1253, 1234, 1144, 998, 979, 928, 848, 803, 756, 698, 608, 596, 521 $cm^{-1}$. **mp** 88.0 - 89.4 °C. **HRMS** (ESI Q-TOF) m/z: [$(M+Na)^+$] calcd. for $C_{21}H_{19}ClN_2O_4Na$ 421.0931 found: 421.0929.

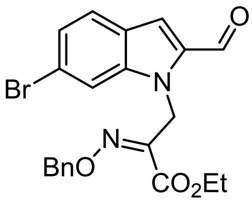

*ethyl (Z)-2-((benzyloxy)imino)-3-(6-bromo-2-formyl-1H-indol-1-yl)propanoate* (**1f**). The synthesis was carried out according to **GP 3 b)**. A scale of 0.89 mmol, white solid, 0.30 g (0.68 mmol), isolated yield of 78%. ; **$^{1}$H NMR** (400 MHz, $CDCl_3$) δ 9.74 (s, 1H), 7.54 (dd, *J* = 8.5, 0.6 Hz, 1H), 7.52 (dd, *J* = 0.9, 0.9 Hz, 1H), 7.35 - 7.33 (m, 3H), 7.26 - 7.24 (m, 3H), 7.14 (d, *J* = 0.9, 1H), 5.69 (s, 2H), 5.26 (s, 2H), 4.21 (q, *J* = 7.0 Hz, 2H), 1.22 (t, *J* = 7.0 Hz, 3H). **$^{13}$C{$^{1}$H} NMR** (75.5 MHz, $CDCl_3$) δ 182.4, 162.3, 147.4, 140.9, 136.2, 135.3, 128.9, 128.6, 128.6, 125.0, 125.0, 124.5, 121.1, 117.7, 113.9, 78.7, 62.1, 39.2, 14.0. **IR $\nu_{max}$**: 2984, 2904, 2823, 1712, 1668, 1608, 1514, 1459, 1377, 1333, 1303, 1236, 1167, 1134, 1027, 976, 911, 854, 818, 806, 759, 741, 697, 609, 591 $cm^{-1}$. **mp** 105.8 - 106.8 °C. **HRMS** (ESI Q-TOF) m/z: [$(M+H)^+$] calcd. for $C_{21}H_{20}BrN_2O_4$ 443.0606 found: 443.0603.

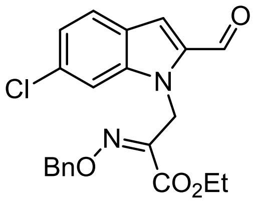

*ethyl (Z)-2-((benzyloxy)imino)-3-(6-chloro-2-formyl-1H-indol-1-yl)propanoate* (**1g**). The synthesis was carried out according to **GP 3 b)**. A scale of 0.92 mmol, yellow solid, 0.28 g (0.69 mmol), isolated yield of 84%. ; **$^{1}$H NMR** (400 MHz, $CDCl_3$) δ 9.73 (s, 1H), 7.59 (d, *J* = 8.2 Hz, 1H), 7.35 - 7.33 (m, 4H), 7.25 - 7.24 (m, 2H), 7.14 (d, *J* = 0.9 Hz, 1H), 7.11 (dd, *J* = 8.5, 1.8 Hz, 1H), 5.70 (s, 2H), 5.26 (s, 2H), 4.21 (q, *J* = 7.0 Hz, 2H), 1.21 (t, *J* = 7.2 Hz, 3H). **$^{13}$C{$^{1}$H} NMR** (75.5 MHz, $CDCl_3$) δ 182.4, 162.3, 147.4, 140.7, 136.3, 135.3, 133.1, 128.9, 128.6, 128.6, 124.7, 124.3, 122.4, 117.7, 110.7, 78.7, 62.1, 39.2, 14.0. **IR $\nu_{max}$**: 3034, 2986, 2904, 2824, 1711, 1668, 1606, 1516, 1461, 1377, 1321, 1245, 1166, 1134, 1061, 1028, 977, 919, 852, 811, 759, 696, 609, 597, 545, 496, 447, 438 $cm^{-1}$. **mp** 106.7 - 108.8 °C. **HRMS** (ESI Q-TOF) m/z: [$(M+Na)^+$] calcd. for $C_{21}H_{19}ClN_2O_4Na$ 421.0931 found: 421.0930.

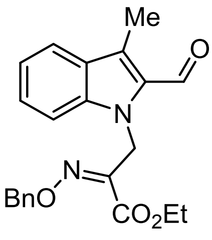
*ethyl (Z)-2-((benzyloxy)imino)-3-(2-formyl-3-methyl-1H-indol-1-yl)propanoate* (**1h**). The synthesis was carried out according to **GP 3 b)**. A scale of 1.38 mmol, yellow solid, 0.29 g (0.77 mmol), isolated yield of 56%. ; **$^{1}$H NMR** (400 MHz, $CDCl_3$) δ 10.00 (s, 1H), 7.65 - 7.63 (m, 1H), 7.37 (s, 1H), 7.35 - 7.33 (m, 2H), 7.29 - 7.25 (m, 1H), 7.23 - 7.19 (m, 3H), 7.14 - 7.10 (m, 1H), 5.73 (s, 2H), 5.23 (s, 2H), 4.17 (q, *J* = 7.0 Hz, 2H), 2.56 (s, 3H), 1.16 (t, *J* = 7.2 Hz, 3H). **$^{13}$C{$^{1}$H} NMR** (75.5 MHz, $CDCl_3$) δ 181.3, 162.5, 148.5, 139.4, 135.8, 131.1, 128.8, 128.5, 128.4, 127.5, 127.1, 127.0, 121.2, 120.5, 110.5, 78.4, 61.9, 39.3, 13.9, 8.5. **IR $\nu_{max}$**: 3011, 2919, 2867, 1720, 1662, 1613, 1533, 1465, 1397, 1371, 1342, 1322, 1260, 1234, 1185, 1166, 1143, 1023, 1001, 979, 927, 876, 852, 822, 759, 740, 695, 606, 517 $cm^{-1}$. **mp** 69.8 - 72.4 °C. **HRMS** (ESI Q-TOF) m/z: [$(M+H)^+$] calcd. for $C_{22}H_{23}N_2O_4$ 379.1658 found: 379.1654.

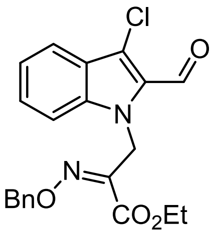
*ethyl (Z)-2-((benzyloxy)imino)-3-(3-chloro-2-formyl-1H-indol-1-yl)propanoate* (**1i**). The synthesis was carried out according to **GP 3 b)**. A scale of 0.84 mmol, yellow solid, 0.24 g (0.60 mmol), isolated yield of 65%. ; **$^{1}$H NMR** (400 MHz, $CDCl_3$) δ 9.97 (s, 1H), 7.69 (dt, *J* = 8.2, 1.0 Hz, 1H), 7.36 - 7.32 (m, 3H), 7.29 (ddd, *J* = 8.5, 6.8, 1.2 Hz, 1H), 7.23 - 7.17 (m, 4H), 5.73 (s, 2H), 5.22 (s, 2H), 4.20 (q, *J* = 7.0 Hz, 2H), 1.20 (t, *J* = 7.0 Hz, 3H). **$^{13}$C{$^{1}$H} NMR** (75.5 MHz, $CDCl_3$) δ 181.0, 162.3, 147.6, 138.3, 135.5, 129.1, 128.8, 128.6, 128.3, 124.3, 121.8, 120.5, 120.1, 110.9, 78.7, 62.1, 39.3, 14.0. **IR $\nu_{max}$**: 3014, 2981, 1719, 1666, 1612, 1509, 1462, 1412, 1372, 1322, 1256, 1229, 1169, 1149, 979, 928, 852, 823, 741, 697, 606, 517, 498 $cm^{-1}$. **mp** 72.8 - 73.2 °C. **HRMS** (ESI Q-TOF) m/z: [$(M+Na)^+$] calcd. for $C_{21}H_{19}ClN_2O_4Na$ 421.0931 found: 421.0928.

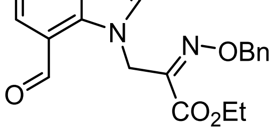
*ethyl (Z)-2-((benzyloxy)imino)-3-(7-formyl-1H-indol-1-yl)propanoate* (**1j**). The synthesis was carried out according to **GP 6**. A scale of 2.07 mmol, yellow oil, 0.16 g (0.43 mmol), isolated yield of 21%. **$^{1}$H NMR** (700 MHz, $CDCl_3$) δ 9.83 (s, 1H), 7.85 (dd, *J* = 7.7, 1.3 Hz, 1H), 7.56 (dd, *J* = 7.3, 0.9 Hz, 1H), 7.37 - 7.34 (m, 4H), 7.24 - 7.22 (m, 3H), 7.12 (d, *J* = 3.0 Hz, 1H), 6.61 (d, *J* = 3.0 Hz, 1H), 5.96 (s, 2H), 5.24 (s, 2H), 4.16 (q, *J* = 7.2 Hz, 2H), 1.14 (t, *J* = 7.1 Hz, 3H). **$^{13}$C{$^{1}$H} NMR** (101 MHz, $CDCl_3$) δ 192.1, 162.7, 148.9, 135.7, 133.9, 132.6, 131.1, 130.7, 128.7, 128.5, 128.4, 128.1,

123.3, 119.0, 104.1, 78.3, 62.0, 44.2, 13.8. **IR $v_{max}$**: 3032, 2981, 2938, 2874, 2740, 1716, 1682, 1599, 1573, 1523, 1453, 1371, 1310, 1264, 1222, 1180, 1137, 1107, 1004, 857, 795, 726, 695, 602, 479, 460 $cm^{-1}$. **HRMS** (ESI Q-TOF) m/z: $[(M+H)^+]$ calcd. for $C_{21}H_{21}N_2O_4$ 365.1501 found: 365.1497.

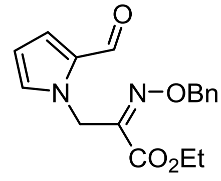

*ethyl (Z)-2-((benzyloxy)imino)-3-(2-formyl-1H-pyrrol-1-yl)propanoate* (**1k**). The synthesis was carried out according to **GP 3 b)**. A scale of 1.36 mmol, white oil, 0.28 g (0.89 mmol), isolated yield of 65%. **$^1$H NMR** (400 MHz, $CDCl_3$) δ 9.48 (d, *J* = 0.9 Hz, 1H), 7.37 - 7.33 (m, 3H), 7.29 - 7.27 (m, 2H), 6.88 - 6.87 (m, 2H), 6.18 (dd, *J* = 3.8, 2.6 Hz, 1H), 5.46 (s, 2H), 5.29 (s, 2H), 4.28 (q, *J* = 7.1 Hz, 2H), 1.29 (t, *J* = 7.2 Hz, 3H). **$^{13}$C{$^1$H} NMR** (101 MHz, $CDCl_3$) δ 179.3, 162.5, 147.7, 135.8, 131.8, 131.6, 128.5, 128.5, 124.1, 110.0, 78.4, 62.2, 42.1, 14.0. **IR $v_{max}$**: 3032, 2981, 2936, 2807, 1718, 1658, 1531, 1478, 1404, 1370, 1301, 1243, 1218, 1176, 1133, 1078, 1008, 954, 876, 857, 809, 763, 742, 697, 607 $cm^{-1}$. **HRMS** (ESI Q-TOF) m/z: $[(M+Na)^+]$ calcd. for $C_{17}H_{18}N_2O_4Na$ 337.1165 found: 337.1164.

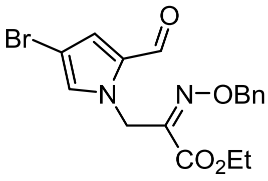

*ethyl (Z)-2-((benzyloxy)imino)-3-(4-bromo-2-formyl-1H-pyrrol-1-yl)propanoate* (**1l**). The synthesis was carried out according to **GP 3 b)**. A scale of 1.15 mmol, white oil, 0.34 g (0.85 mmol), isolated yield of 75%. **$^1$H NMR** (400 MHz, $CDCl_3$) δ 9.40 (d, *J* = 0.6 Hz, 1H), 7.38 - 7.34 (m, 3H), 7.28 - 7.26 (m, 2H), 6.83 (s, 2H), 5.40 (s, 2H), 5.28 (s, 2H), 4.31 (q, *J* = 7.1 Hz, 2H), 1.32 (t, *J* = 7.2 Hz, 3H). **$^{13}$C{$^1$H} NMR** (101 MHz, $CDCl_3$) δ 178.8, 162.4, 147.0, 135.5, 131.8, 130.9, 128.6, 124.6, 97.3, 78.7, 62.3, 42.1, 14.1. **IR $v_{max}$**: 3122, 2978, 2965, 2943, 2933, 1720, 1658, 1474, 1387, 1329, 1295, 1242, 1179, 1167, 1121, 1094, 1022, 1002, 960, 947, 932, 918, 889, 847, 822, 806, 772, 755, 722, 698, 594 $cm^{-1}$. **HRMS** (ESI Q-TOF) m/z: $[(M+Na)^+]$ calcd. for $C_{17}H_{17}BrN_2O_4Na$ 415.0270 found: 415.0269.

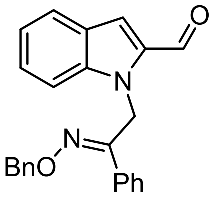

*(E)-1-(2-((benzyloxy)imino)-2-phenylethyl)-1H-indole-2-carbaldehyde* (**1m**). The synthesis was carried out according to **GP 3 b)**. A scale of 1.03 mmol, yellow solid, 0.28 g (0.77 mmol), isolated yield of 74%. **$^1$H NMR** (400 MHz, $CDCl_3$) δ 9.75 (s, 1H), 7.60 - 7.57 (m, 1H), 7.53 - 7.51 (m, 2H), 7.46 - 7.42 (m, 2H), 7.41 - 7.39 (m, 1H), 7.23 - 7.19 (m, 4H), 7.17 - 7.11 (m, 2H), 7.10 - 7.08 (m, 3H), 6.09 (s, 2H), 5.37 (s, 2H). **$^{13}$C{$^1$H} NMR** (101 MHz, $CDCl_3$) δ 183.1, 156.1, 140.6, 137.5, 135.2,

133.3, 129.1, 128.8, 128.6, 128.2, 128.0, 127.4, 127.2, 126.3, 123.3, 121.3, 118.9, 110.9, 77.1, 40.5. **IR** $\mathbf{v_{max}}$**:** 2981, 2938, 1686, 1657, 1578, 1461, 1448, 1223, 1114, 993, 978, 845, 827, 811, 752, 739, 729, 685, 618, 590, 562, 487, 439 $cm^{-1}$. **HRMS** (ESI Q-TOF) m/z: $[(M+H)^+]$ calcd. for $C_{24}H_{21}N_2O_2$ 369.1603 found: 369.1599.

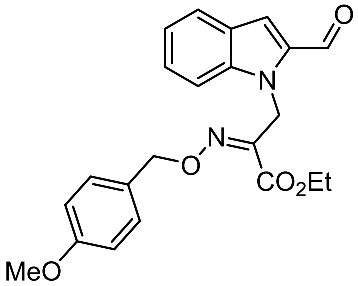

*ethyl (Z)-3-(2-formyl-1H-indol-1-yl)-2-(((4-methoxybenzyl)oxy)imino)propanoate* (**1n**). The synthesis was carried out according to **GP 3 b)**. A scale of 0.55 mmol, white solid, 0.12 g (0.30 mmol), isolated yield of 53%. ; $^{1}$**H NMR** (400 MHz, $CDCl_3$) δ 9.75 (s, 1H), 7.69 - 7.66 (m, 1H), 7.24 (dd, *J* = 6.5, 1.2 Hz, 1H), 7.23 - 7.19 (m, 2H), 7.18 - 7.17 (m, 2H), 7.14 (ddd, *J* = 7.9, 6.5, 1.5 Hz, 1H), 6.88 - 6.85 (m, 2H), 5.75 (s, 2H), 5.19 (s, 2H), 4.15 (q, *J* = 7.0 Hz, 2H), 3.83 (s, 3H), 1.15 (t, J = 7.0 Hz, 3H). $^{13}$**C{$^{1}$H} NMR** (75.5 MHz, $CDCl_3$) δ 182.6, 162.3, 159.9, 147.8, 140.4, 135.8, 130.6, 127.1, 126.3, 123.3, 121.2, 118.0, 113.9, 110.7, 78.3, 61.9, 55.3, 39.1, 13.9. **IR** $\mathbf{v_{max}}$: 2984, 2949, 2932, 2835, 1715, 1677, 1611, 1511, 1464, 1321, 1253, 1168, 1147, 1128, 995, 983, 849, 816, 794, 751, 695, 607, 589, 490, 443 $cm^{-1}$. **mp** 75.7 - 77.7 °C. **HRMS** (ESI Q-TOF) m/z: $[(M+Na)^+]$ calcd. for $C_{22}H_{22}N_2O_5Na$ 417.1427 found: 417.1421.

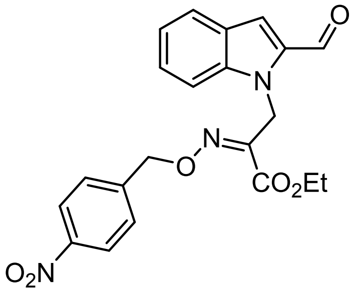

*ethyl (Z)-3-(2-formyl-1H-indol-1-yl)-2-(((4-nitrobenzyl)oxy)imino)propanoate* (**1o**). The synthesis was carried out according to **GP 3 b)**. A scale of 0.29 mmol, white solid, 0.024 g (0.0059 mmol), isolated yield of 20%. ; $^{1}$**H NMR** (700 MHz, $CDCl_3$) δ 9.82 (s, 1H), 8.13 - 8.11 (m, 2H), 7.74 - 7.72 (m, 1H), 7.34 - 7.30 (m, 2H), 7.25 (d, *J* = 0.9 Hz, 1H), 7.23 - 7.21 (m, 2H), 7.19 (ddd, *J* = 8.1, 6.3, 1.5 Hz, 1H), 5.76 (s, 2H), 5.29 (s, 2H), 4.22 (q, *J* = 7.3 Hz, 2H), 1.20 (t, *J* = 7.1 Hz, 3H). $^{13}$**C{$^{1}$H} NMR** (176 MHz, $CDCl_3$) δ 182.5, 162.2, 149.1, 147.7, 143.1, 140.5, 135.6, 128.6, 127.2, 126.3, 123.6, 123.5, 121.4, 118.2, 110.5, 76.6, 62.2, 39.8, 13.9. **IR** $\mathbf{v_{max}}$**:** 2983, 2804, 1718, 1673, 1615, 1606, 1517, 1464, 1344, 1320, 1257, 1244, 1162, 1145, 1123, 1112, 990, 964, 851, 799, 749, 739, 707, 689 $cm^{-1}$. **mp:** 115.4 - 118.1°C. **HRMS** (ESI Q-TOF) m/z: $[(M+Na)^+]$ calcd. for $C_{21}H_{19}N_3O_6Na$ 432.1171 found: 432.1169.

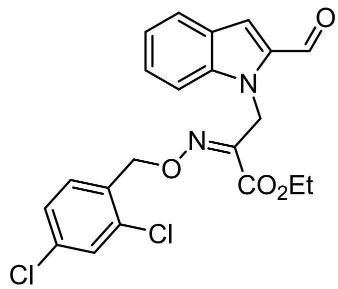

*ethyl (Z)-2-(((2,4-dichlorobenzyl)oxy)imino)-3-(2-formyl-1H-indol-1-yl)propanoate* (**1p**). The synthesis was carried out according to **GP 3 b)**. A scale of 0.56 mmol, white solid, 0.17 g (0.38 mmol), isolated yield of 69%. ; **$^1$H NMR** (700 MHz, $CDCl_3$) δ 9.81 (s, 1H), 7.71 - 7.69 (m, 1H), 7.37 (d, $J$ = 1.7 Hz, 1H), 7.31 - 7.28 (m, 2H), 7.23 (s, 1H), 7.17 (ddd, $J$ = 7.7, 6.0, 2.2 Hz, 1H), 7.15 (dd, $J$ = 8.2, 2.2 Hz, 1H), 7.03 (d, $J$ = 8.6 Hz, 1H), 5.76 (s, 2H), 5.31 (s, 2H), 4.20 (q, $J$ = 7.3 Hz, 2H), 1.20 (t, $J$ = 7.1 Hz, 3H). **$^{13}$C{$^1$H} NMR** (176 MHz, $CDCl_3$) δ 182.5, 182.5, 162.2, 148.7, 140.4, 135.6, 134.9, 134.6, 132.2, 131.2, 129.4, 127.1, 126.3, 123.4, 121.3, 118.2, 110.6, 74.6, 62.1, 39.5, 13.9. **IR $v_{max}$**: 3185, 3072, 2973, 2958, 2853, 1719, 1660, 1614, 1588, 1464, 1340, 1304, 1253, 1232, 1171, 1126, 1101, 1056, 984, 958, 849, 821, 798, 750, 733, 587, 494, 480, 438 cm$^{-1}$. **mp** 81.8 - 84.3 °C. **HRMS** (ESI Q-TOF) m/z: [(M+Na)$^+$] calcd. for $C_{21}H_{18}Cl_2N_2O_4Na$ 455.0542 found: 455.0539.

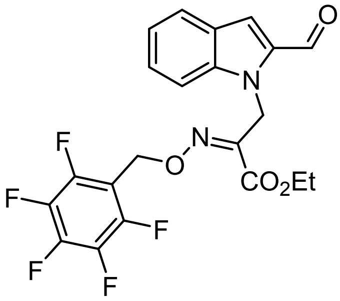

*ethyl (Z)-3-(2-formyl-1H-indol-1-yl)-2-(((perfluorophenyl)methoxy)imino)propanoate* (**1q**). The synthesis was carried out according to **GP 3 b)**. A scale of 0.23 mmol, yellow oil, 0.066 g (0.15 mmol), isolated yield of 63%. ; **$^1$H NMR** (400 MHz, $CDCl_3$) δ 9.80 (s, 1H), 7.65 (d, $J$ = 8.2 Hz, 1H), 7.25 - 7.24 (m, 1H), 7.22 (s, 1H), 7.13 - 7.09 (m, 2H), 5.61 (s, 2H), 5.21 - 5.20 (m, 2H), 4.25 (q, $J$ = 7.0 Hz, 2H), 1.24 (t, $J$ = 7.2 Hz, 3H). **$^{13}$C{$^1$H} NMR** (101 MHz, $CDCl_3$) δ 182.4, 162.2, 149.2, 140.4, 135.4, 126.7, 126.2, 123.3, 121.1, 118.1, 110.4, 64.1, 62.2, 39.7, 13.9 (The description does not include signals from C-F carbon atoms in the $^{13}$C spectrum due to the multiplicity and low intensity). **IR $v_{max}$:** 2963, 2917, 2849, 1723, 1668, 1522, 1504, 1462, 1306, 1126, 1059, 1014, 943, 844, 804, 752, 736, 672 cm$^{-1}$. **HRMS** (ESI Q-TOF) m/z: [(M+H)$^+$] calcd. for $C_{21}H_{16}F_5N_2O_4$ 455.1025 found: 455.1030.

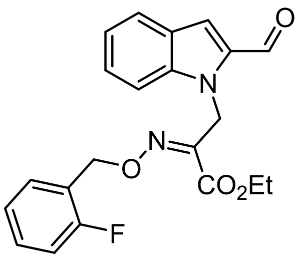


*ethyl (Z)-2-(((2-fluorobenzyl)oxy)imino)-3-(2-formyl-1H-indol-1-yl)propanoate* (**1r**). The synthesis was carried out according to **GP 3 b)**. A scale of 1.14 mmol, yellow solid, 0.22 g (0.57 mmol), isolated yield of 50%. ; **$^1$H NMR** (400 MHz, $CDCl_3$) δ 9.80 (s, 1H), 7.71 - 7.69 (m, 1H), 7.39 - 7.33 (m, 1H), 7.30 - 7.28 (m, 2H), 7.26 - 7.22 (m, 1H), 7.22 (s, 1H), 7.18 - 7.14 (m, 1H), 7.14 - 7.11 (m, 1H), 7.10 - 7.09 (m, 1H), 5.79 (s, 2H), 5.36 (s, 2H), 4.18 (q, *J* = 7.0 Hz, 2H), 1.18 (t, *J* = 7.2 Hz, 3H). **$^{13}$C{$^1$H} NMR** (101 MHz, $CDCl_3$) δ 182.6, 162.2, 161.1 (d, *J* = 248.9 Hz), 148.2, 140.4, 135.7, 131.2 (d, *J* = 3.1 Hz), 130.5 (d, *J* = 8.5 Hz), 127.1, 126.3, 124.1 (d, *J* = 3.1 Hz), 123.3, 123.0 (d, *J* = 14.6 Hz), 121.3, 118.1, 115.5 (d, *J* = 20.8 Hz), 110.6, 71.9 (d, *J* = 3.9 Hz), 61.9, 39.1, 13.9. **IR $\nu_{max}$**: 3018, 2981, 2964, 2814, 2726, 1720, 1673, 1615, 1492, 1463, 1372, 1321, 1254, 1233, 1167, 1146, 1125, 1112, 1004, 982, 870, 844, 798, 751, 735, 694, 616, 490, 440 $cm^{-1}$. **mp** 74.1 - 75.5 °C. **HRMS** (ESI Q-TOF) m/z: [$(M+H)^+$] calcd. for $C_{21}H_{20}FN_2O_4$ 383.1407 found: 383.1404.

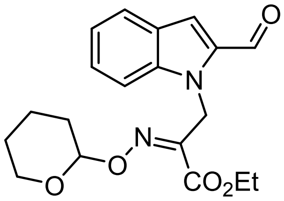


*ethyl (Z)-3-(2-formyl-1H-indol-1-yl)-2-(((tetrahydro-2H-pyran-2-yl)oxy)imino)propanoate* (**1s**). The synthesis was carried out according to **GP 3 b)**. A scale of 1.55 mmol, colorless oil, 0.082 g (0.28 mmol), isolated yield of 15%. ; **$^1$H NMR** (400 MHz, $CDCl_3$) δ 9.90 (s, 1H), 7.74 (dt, *J* = 7.9, 0.9 Hz, 1H), 7.44 - 7.42 (m, 2H), 7.30 (d, *J* = 0.6 Hz, 1H), 7.20 (ddd, *J* = 8.1, 5.7, 2.1 Hz, 1H), 5.85 (d, *J* = 16.4 Hz, 1H), 5.78 (d, *J* = 16.4 Hz, 1H), 5.41 (m, 1H), 4.21 (m, 2H), 3.62 (dd, *J* = 8.4, 3.2 Hz, 2H), 1.70 (m, 1H), 1.62 (m, 2H), 1.50 (m, 3H), 1.18 (t, *J* = 6.8 Hz, 3H). **$^{13}$C{$^1$H} NMR** (176 MHz, $CDCl_3$) δ 182.5, 162.4, 148.9, 140.5, 135.6, 127.3, 126.4, 123.4, 121.4, 118.1, 110.7, 102.4, 62.6, 62.0, 39.8, 28.2, 24.8, 18.8, 13.9. **IR $\nu_{max}$**: 2944, 2871, 1718, 1666, 1463, 1372, 1353, 1314, 1255, 1205, 1172, 1115, 1063, 1039, 1018, 997, 948, 899, 845, 818, 800, 753, 737, 438 $cm^{-1}$. **HRMS** (ESI Q-TOF) m/z: [$(M+Na)^+$] calcd. for $C_{19}H_{22}N_2O_5Na$ 381.1427 found: 381.1426.

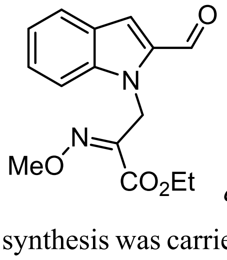


*ethyl (Z)-3-(2-formyl-1H-indol-1-yl)-2-(methoxyimino)propanoate* (**1t**). The synthesis was carried out according to **GP 3 b)**. A scale of 1.01 mmol, colorless oil, 0.080 g (0.28 mmol), isolated yield of 27%. ; **$^1$H NMR** (400 MHz, $CDCl_3$) δ 9.91 (s, 1H), 7.74 (dt, *J* = 8.2, 0.9 Hz, 1H), 7.42

(ddd, $J$ = 8.5, 6.8, 1.2 Hz, 1H), 7.36 (dq, $J$ = 8.5, 0.9 Hz, 1H), 7.29 (d, $J$ = 0.9 Hz, 1H), 7.20 (ddd, $J$ = 8.1, 6.9, 1.2 Hz, 1H), 5.79 (s, 2H), 4.14 (q, $J$ = 7.04 Hz, 2H), 4.11 (s, 3H), 1.13 (t, $J$ = 7.2 Hz, 3H). **$^{13}C\{^1H\}$ NMR** (101 MHz, $CDCl_3$) δ 182.6, 162.1, 147.9, 140.5, 135.8, 127.2, 126.4, 123.4, 121.3, 118.1, 110.6, 63.7, 61.9, 38.9, 13.8. **IR $v_{max}$**: 2982, 2940, 2821, 1721, 1665, 1613, 1522, 1461, 1371, 1351, 1313, 1252, 1234, 1169, 1127, 1039, 1017, 997, 929, 843, 802, 751, 734, $cm^{-1}$. **HRMS** (ESI Q-TOF) m/z: $[(M+H)^+]$ calcd. for $C_{15}H_{17}N_2O_4$ 289.1188 found: 289.1184.

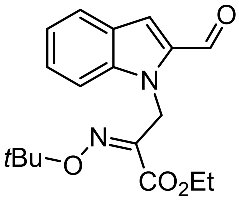


*ethyl (Z)-2-(tert-butoxyimino)-3-(2-formyl-1H-indol-1-yl)propanoate* (**1u**). The synthesis was carried out according to **GP 3 b)**. A scale of 0.68 mmol, white solid, 0.091 g (0.28 mmol), isolated yield of 41%. ; **$^1H$ NMR** (700 MHz, $CDCl_3$) δ 9.90 (s, 1H), 7.74 - 7.72 (m, 1H), 7.40 - 7.39 (m, 2H), 7.27 (s, 1H), 7.19 (ddd, $J$ = 8.1, 5.1, 2.8 Hz, 1H), 5.77 (s, 2H), 4.16 (q, $J$ = 7.1 Hz, 2H), 1.25 (s, 9H), 1.18 (t, $J$ = 7.1 Hz, 3H). **$^{13}C\{^1H\}$ NMR** (176 MHz, $CDCl_3$) δ 182.6, 162.9, 146.1, 140.5, 136.0, 126.9, 126.3, 123.3, 121.2, 117.8, 110.9, 82.1, 61.4, 39.1, 27.3, 13.9, **IR $v_{max}$:** 2974, 2801, 2733, 1714, 1667, 1467, 1370, 1325, 1258, 1173, 1158, 1129, 1112, 1002, 979, 912, 846, 820, 802, 753, 739, 663, 609, 588, 492, 443 $cm^{-1}$. **mp:** 60.9 - 62.9°C. **HRMS** (ESI Q-TOF) m/z: $[(M+H)^+]$ calcd. for $C_{18}H_{23}N_2O_4$ 331.1658 found: 331.1656.

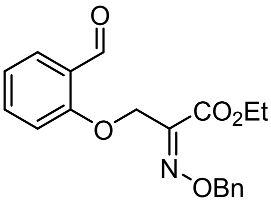


*ethyl (Z)-2-((benzyloxy)imino)-3-(2-formylphenoxy)propanoate* (**3a**). The synthesis was carried out according to **GP 3 c)**. A scale of 0.82 mmol, white solid, 0.12 g (0.36 mmol), isolated yield of 44%. **$^1H$ NMR** (700 MHz, $CDCl_3$) δ 10.39 (d, $J$ = 1.1 Hz, 1H), 7.84 (dd, $J$ = 7.7, 1.9 Hz, 1H), 7.51 - 7.48 (m, 1H), 7.40 - 7.38 (m, 5H), 7.08 - 7.06 (m, 1H), 7.03 (d, $J$ = 8.5 Hz, 1H), 5.39 (s, 2H), 5.10 (s, 2H), 4.37 (q, $J$ = 7.1 Hz, 2H), 1.36 (t, $J$ = 6.9 Hz, 3H). **$^{13}C\{^1H\}$ NMR** (101 MHz, $CDCl_3$) δ 189.4, 162.3, 160.5, 147.4, 135.80, 135.76, 128.63, 128.57, 128.3, 125.4, 121.6, 113.1, 78.7, 62.2, 59.3, 14.1. **IR $v_{max}$**: 2994, 2979, 2938, 2868, 2769, 1706, 1685, 1599, 1483, 1455, 1391, 1326, 1284, 1239, 1169, 1147, 1100, 997, 890, 843, 817, 755, 733, 695, 662 $cm^{-1}$. **mp:** 77.0 - 79.8°C. **HRMS** (ESI Q-TOF) m/z: $[(M+Na)^+]$ calcd. for $C_{19}H_{19}NO_5Na$ 364.1161 found: 364.1159.

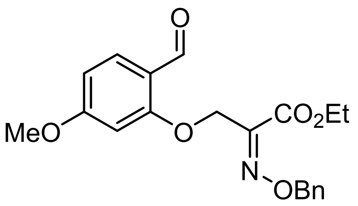

*ethyl (Z)-2-((benzyloxy)imino)-3-(2-formyl-5-methoxyphenoxy)propanoate* (**3b**). The synthesis was carried out according to **GP 3 c)**. A scale of 0.99 mmol, white solid, 0.19 g (0.51 mmol), isolated yield of 51%. $^{1}$**H NMR** (400 MHz, $CDCl_3$) δ 10.20 (d, *J* = 0.9 Hz, 1H), 7.80 (d, *J* = 8.8 Hz, 1H), 7.37 – 7.36 (m, 5H), 6.56 (ddd, *J* = 8.8, 2.2, 0.9 Hz, 1H), 6.51 (d, *J* = 2.1 Hz, 1H), 5.36 (s, 2H), 5.05 (s, 2H), 4.35 (q, *J* = 7.0 Hz, 2H), 3.81 (s, 3H), 1.34 (t, *J* = 7.2 Hz, 3H). $^{13}$**C{$^{1}$H} NMR** (101 MHz, $CDCl_3$) δ 188.0, 166.0, 162.3, 147.3, 135.7, 130.2, 128.62, 128.56, 119.4, 107.1, 99.0, 78.7, 62.3, 59.2, 55.6, 14.1. **mp:** 68.4 - 70.0°C. **IR $\nu_{max}$**: 2979, 2939, 2858, 2772, 1710, 1677, 1602, 1499, 1388, 1328, 1312, 1289, 1262, 1242, 1197, 1154, 1110, 995, 934, 862, 833, 783, 739, 692, 629, 595, 569 cm$^{-1}$. **HRMS** (ESI Q-TOF) m/z: [$(M+Na)^+$] calcd. for $C_{20}H_{21}NO_6Na$ 394.1267 found: 394.1264.

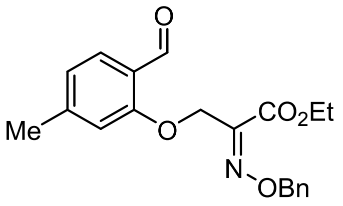

*ethyl (Z)-2-((benzyloxy)imino)-3-(2-formyl-5-methylphenoxy)propanoate* (**3c**). The synthesis was carried out according to **GP 3 c)**. A scale of 1.14 mmol, white solid, 0.20 g (0.55 mmol), isolated yield of 48%. $^{1}$**H NMR** (400 MHz, $CDCl_3$) δ 10.29 (d, *J* = 0.9 Hz, 1H), 7.71 (d, *J* = 7.6 Hz, 1H), 7.37 – 7.36 (m, 5H), 6.85 (d, *J* = 7.9 Hz, 1H), 6.81 (s, 1H), 5.36 (s, 2H), 5.06 (s, 2H), 4.35 (q, *J* = 7.0 Hz, 2H), 2.33 (s, 3H), 1.34 (t, *J* = 7.0 Hz, 3H). $^{13}$**C{$^{1}$H} NMR** (101 MHz, $CDCl_3$) δ 189.1, 162.3, 160.6, 147.5, 147.3, 135.8, 128.6, 128.5, 128.2, 123.2, 122.6, 113.6, 78.7, 62.2, 59.3, 22.2, 14.1. **mp:** 83.1 - 84.7°C. **IR $\nu_{max}$**: 2975, 2937, 2860, 2768, 1709, 1682, 1605, 1455, 1412, 1389, 1329, 1258, 1239, 1206, 1148, 1109, 1038, 1017, 995, 944, 932, 883, 861, 844, 811, 783, 753, 737, 695 cm$^{-1}$. **HRMS** (ESI Q-TOF) m/z: [$(M+H)^+$] calcd. for $C_{20}H_{22}NO_5$ 356.1498 found: 356.1493.

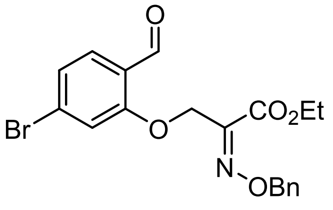

*ethyl (Z)-2-((benzyloxy)imino)-3-(5-bromo-2-formylphenoxy)propanoate* (**3d**). The synthesis was carried out according to **GP 3 c)**. A scale of 0.75 mmol, white solid, 0.14 g (0.34 mmol), isolated yield of 45%. $^{1}$**H NMR** (400 MHz, $CDCl_3$) δ 10.27 (d, *J* = 0.9 Hz, 1H), 7.67 (d, *J* = 8.2 Hz, 1H), 7.38 - 7.37 (m, 5H), 7.23 (d, *J* = 1.8 Hz, 1H), 7.20 (ddd, *J* = 8.3, 1.5, 0.7 Hz, 1H), 5.37 (s, 2H), 5.06 (s, 2H), 4.35 (q, *J* = 7.0 Hz, 2H), 1.35 (t, *J* = 7.2 Hz, 3H). $^{13}$**C{$^{1}$H} NMR** (101 MHz, $CDCl_3$) δ 188.4, 162.1, 160.6, 146.7, 135.5, 130.3, 129.5, 128.71, 128.66, 125.1, 124.3, 116.7, 78.9, 62.4, 59.5, 14.1. **mp:** 74.2 - 75.2°C. **IR $\nu_{max}$**: 2975, 2938, 2865, 2767, 1685, 1588, 1475, 1408, 1385, 1327, 1235,

1150, 986, 897, 860, 835, 807, 774, 753, 733, 695, 673, 637, 595 $cm^{-1}$. **HRMS** (ESI Q-TOF) m/z: $[(M+H)^+]$ calcd. for $C_{19}H_{19}BrNO_5$ 420.0446 found: 420.0440.

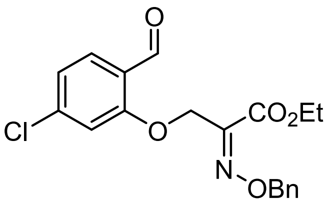

*ethyl (Z)-2-((benzyloxy)imino)-3-(5-chloro-2-formylphenoxy)propanoate* (**3e**). The synthesis was carried out according to **GP 3 c)**. A scale of 1.14 mmol, white solid, 0.13 g (0.35 mmol), isolated yield of 31%. **$^1$H NMR** (400 MHz, $CDCl_3$) δ 10.27 (d, *J* = 0.9 Hz, 1H), 7.75 (d, *J* = 8.2 Hz, 1H), 7.37 (s, 5H), 7.06 (d, *J* = 1.8 Hz, 1H), 7.05 - 7.02 (m, 1H), 5.27 (s, 2H), 5.06 (s, 2H), 4.36 (q, *J* = 7.2 Hz, 2H), 1.35 (t, *J* = 7.0 Hz, 3H). **$^{13}$C{$^1$H} NMR** (101 MHz, $CDCl_3$) δ 188.2, 162.1, 160.8, 146.7, 141.8, 135.5, 129.4, 128.7, 128.7, 123.9, 122.2, 113.7, 78.9, 62.4, 59.4, 14.1. **mp:** 88.7 - 89.4°C. **IR $v_{max}$**: 2976, 2938, 2866, 1686, 1594, 1478, 1410, 1386, 1329, 1238, 1151, 1105, 1088, 994, 911, 877, 861, 834, 811, 777, 754, 734, 696, 683, 640, 597 $cm^{-1}$. **HRMS** (ESI Q-TOF) m/z: $[(M+Na)^+]$ calcd. for $C_{19}H_{18}ClNO_5Na$ 398.0772 found: 398.0770.

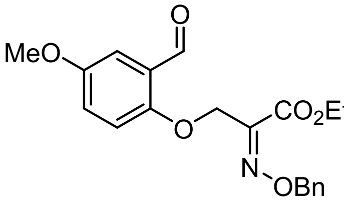

*ethyl (Z)-2-((benzyloxy)imino)-3-(2-formyl-4-methoxyphenoxy)propanoate* (**3f**). The synthesis was carried out according to **GP 3 c)**. A scale of 0.99 mmol, white solid, 0.12 g (0.32 mmol), isolated yield of 32%. **$^1$H NMR** (400 MHz, $CDCl_3$) δ 10.32 (s, 1H), 7.39 - 7.34 (m, 5H), 7.29 (d, *J* = 3.2 Hz, 1H), 7.03 (dd, *J* = 9.1, 3.2 Hz, 1H), 6.97 (d, *J* = 9.1 Hz, 1H), 5.34 (s, 2H), 5.03 (s, 2H), 4.34 (q, *J* = 7.0 Hz, 2H), 3.79 (s, 3H), 1.34 (t, *J* = 7.0 Hz, 3H). **$^{13}$C{$^1$H} NMR** (176 MHz, $CDCl_3$) δ 189.3, 162.3, 155.3, 154.4, 147.6, 135.8, 128.63, 128.60, 126.0, 123.3, 115.6, 110.1, 78.7, 62.2, 60.2, 55.8, 14.1. **mp:** 59.8 - 62.5°C. **IR $v_{max}$**: 3005, 2978, 2942, 2873, 2835, 1682, 1495, 1452, 1427, 1400, 1329, 1312, 1280, 1261, 1241, 1212, 1191, 1148, 996, 938, 884, 859, 827, 807, 772, 751, 734, 714, 694, $cm^{-1}$. **HRMS** (ESI Q-TOF) m/z: $[(M+H)^+]$ calcd. for $C_{20}H_{22}NO_6$ 372.1447 found: 372.1444.

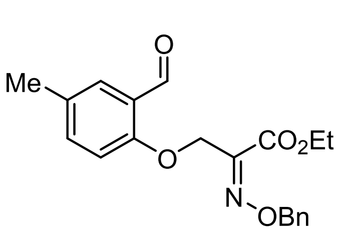

*ethyl (Z)-2-((benzyloxy)imino)-3-(2-formyl-4-methylphenoxy)propanoate* (**3g**). The synthesis was carried out according to **GP 3 c)**. A scale of 1.10 mmol, white solid, 0.21 g (0.59 mmol), isolated yield of 54%. **$^1$H NMR** (400 MHz, $CDCl_3$) δ 10.33 (s, 1H), 7.60 (d, *J* = 2.1 Hz, 1H), 7.37 - 7.35 (m, 5H), 7.27 - 7.25 (m, 1H), 6.90 (d, *J* = 8.2 Hz, 1H), 5.34 (s, 2H), 5.04 (s, 2H), 4.33 (q, *J*

= 7.0 Hz, 2H), 2.29 (s, 3H), 1.32 (t, *J* = 7.0 Hz, 3H). **$^{13}$C{$^{1}$H} NMR** (101 MHz, $CDCl_3$) δ 189.7, 162.3, 158.7, 147.6, 136.4, 135.8, 131.1, 128.61, 128.59, 128.56, 128.2, 125.2, 113.2, 78.7, 62.2, 59.6, 20.3, 14.1. **mp:** 88.5 - 89.8 °C. **IR $\nu_{max}$**: 2998, 2940, 2870, 1683, 1493, 1453, 1326, 1287, 1232, 1152, 1115, 1041, 995, 941, 884, 861, 812, 771, 752, 730, 693 $cm^{-1}$. **HRMS** (ESI Q-TOF) m/z: [$(M+H)^+$] calcd. for $C_{20}H_{22}NO_5$ 356.1498 found: 356.1492.

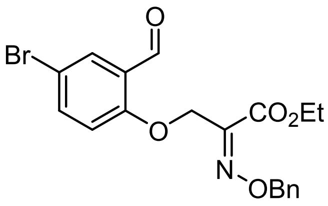


*ethyl (Z)-2-((benzyloxy)imino)-3-(4-bromo-2-formylphenoxy)propanoate* (**3h**). The synthesis was carried out according to **GP 3 c)**. A scale of 0.75 mmol, white solid, 0.071 g (0.17 mmol), isolated yield of 22%. **$^{1}$H NMR** (400 MHz, $CDCl_3$) δ 10.25 (s, 1H), 7.89 (d, *J* = 2.6 Hz, 1H), 7.52 (dd, *J* = 8.8, 2.6 Hz, 1H), 7.39 - 7.33 (m, 5H), 6.91 (d, *J* = 9.1 Hz, 1H), 5.35 (s, 2H), 5.05 (s, 2H), 4.35 (q, J = 7.0 Hz, 2H), 1.34 (t, *J* = 7.0 Hz, 3H). **$^{13}$C{$^{1}$H} NMR** (101 MHz, $CDCl_3$) δ 188.0, 162.2, 159.4, 146.9, 138.1, 135.6, 130.9, 128.75, 128.68, 128.66, 126.7, 115.2, 114.5, 78.9, 62.3, 59.6, 14.1. **mp:** 101.8 - 102.6°C. **IR $\nu_{max}$**: 3100, 3048, 3032, 2979, 2938, 2862, 1722, 1681, 1590, 1478, 1451, 1388, 1329, 1277, 1238, 1180, 1146, 992, 880, 818, 766, 752, 730, 694 $cm^{-1}$. **HRMS** (ESI Q-TOF) m/z: [$(M+Na)^+$] calcd. for $C_{19}H_{18}BrNO_5Na$ 442.0266 found: 442.0265.

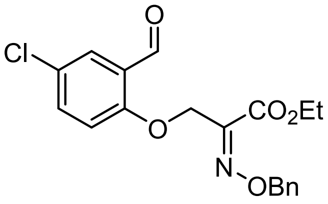


*ethyl (Z)-2-((benzyloxy)imino)-3-(4-chloro-2-formylphenoxy)propanoate* (**3i**). The synthesis was carried out according to **GP 3 c)**. A scale of 0.96 mmol, white solid, 0.12 g (0.33 mmol), isolated yield of 34%. **$^{1}$H NMR** (400 MHz, $CDCl_3$) δ 10.26 (d, *J* = 0.9 Hz, 1H), 7.75 (d, *J* = 2.9 Hz, 1H), 7.39 - 7.33 (m, 6H), 6.96 (d, *J* = 8.8 Hz, 1H), 5.35 (s, 2H), 5.05 (s, 2H), 4.34 (q, *J* = 7.2 Hz, 2H), 1.34 (t, *J* = 7.0 Hz, 3H). **$^{13}$C{$^{1}$H} NMR** (101 MHz, $CDCl_3$) δ 188.2, 162.2, 158.9, 146.9, 135.6, 135.2, 128.75, 128.68, 128.66, 127.9, 127.3, 126.3, 114.9, 78.9, 62.3, 59.5, 14.1. **mp:** 98.7 - 100.4°C. **IR $\nu_{max}$**: 3102, 3068, 2980, 2939, 2864, 1721, 1682, 1596, 1482, 1452, 1390, 1327, 1245, 1232, 1182, 1146, 1127, 993, 885, 820, 752, 730, 694 $cm^{-1}$. **HRMS** (ESI Q-TOF) m/z: [$(M+Na)^+$] calcd. for $C_{19}H_{18}ClNO_5Na$ 398.0766 found: 398.0769.

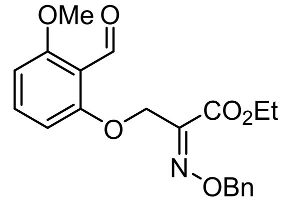


*ethyl (Z)-2-((benzyloxy)imino)-3-(2-formyl-3-methoxyphenoxy)propanoate* (**3j**). The synthesis was carried out according to **GP 3 c)**. A scale of 0.99 mmol, white solid, 0.21 g (0.56

mmol), isolated yield of 56%. **$^1$H NMR** (400 MHz, $CDCl_3$) δ 10.40 (s, 1H), 7.37 - 7.34 (m, 6H), 6.57 (dd, *J* = 8.5, 4.4 Hz, 2H), 5.34 (s, 2H), 5.04 (s, 2H), 4.33 (q, *J* = 7.0 Hz, 2H), 3.88 (s, 3H), 1.33 (t, *J* = 7.0 Hz, 3H). **$^{13}$C{$^1$H} NMR** (101 MHz, $CDCl_3$) 188.9, 162.2, 161.3, 147.7, 135.9, 135.6, 128.59, 128.55, 128.53, 115.1, 105.2, 104.9, 78.6, 62.2, 59.8, 56.1, 14.1. **mp:** 72.8 - 73.8°C. **IR $\nu_{max}$**: 3004, 2969, 2942, 2883, 2840, 1692, 1593, 1474, 1405, 1384, 1324, 1305, 1251, 1231, 1166, 1109, 986, 882, 827, 772, 753, 733, 711, 693 $cm^{-1}$. **HRMS** (ESI Q-TOF) m/z: [$(M+H)^+$] calcd. for $C_{20}H_{22}NO_6$ 372.1447 found: 372.1443.

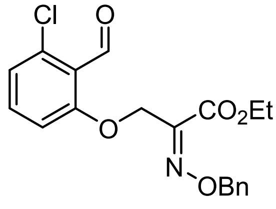


*ethyl (Z)-2-((benzyloxy)imino)-3-(3-chloro-2-formylphenoxy)propanoate* (**3k**). The synthesis was carried out according to **GP 3 c)**. A scale of 0.96 mmol, white solid, 0.10 g (0.27 mmol), isolated yield of 28%. **$^1$H NMR** (400 MHz, $CDCl_3$) δ 10.37 (s, 1H), 7.37 - 7.35 (m, 5H), 7.29 (t, *J* = 8.2 Hz, 1H), 7.04 (d, *J* = 7.6 Hz, 1H), 6.91 (dd, *J* = 8.5, 0.6 Hz, 1H), 5.34 (s, 2H), 5.05 (s, 2H), 4.34 (q, *J* = 7.1 Hz, 2H), 1.34 (t, *J* = 7.2 Hz, 3H). **$^{13}$C{$^1$H} NMR** (101 MHz, $CDCl_3$) δ 188.6, 162.2, 161.0, 147.1, 135.7, 135.5, 134.2, 128.6, 127.9, 124.2, 123.2, 111.9, 78.8, 62.3, 59.9, 14.1. **mp:** 93.5 - 97.3°C. **IR $\nu_{max}$**: 3033, 2989, 2941, 2882, 1696, 1590, 1446, 1384, 1325, 1267, 1232, 1164, 1153, 1136, 1086, 993, 953, 905, 848, 781, 752, 728, 694, 667 $cm^{-1}$. **HRMS** (ESI Q-TOF) m/z: [$(M+Na)^+$] calcd. for $C_{19}H_{18}ClNO_5Na$ 398.0772 found: 398.0770.

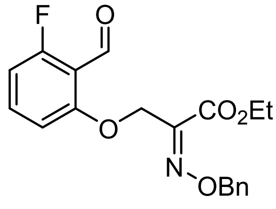


*ethyl (Z)-2-((benzyloxy)imino)-3-(3-fluoro-2-formylphenoxy)propanoate* (**3l**). The synthesis was carried out according to **GP 3 c)**. A scale of 1.07 mmol, white solid, 0.12 g (0.33 mmol), isolated yield of 30%. **$^1$H NMR** (400 MHz, $CDCl_3$) δ 10.30 (m, 1H), 7.38 - 7.34 (m, 6H), 6.78 (d, *J* = 8.5 Hz, 1H), 6.73 (dd, *J* = 10.0, 8.,7 Hz, 1H), 5.35 (s, 2H), 5.06 (s, 2H), 4.35 (q, *J* = 7.0 Hz, 2H), 1.34 (t, *J* = 7.0 Hz, 3H). **$^{13}$C{$^1$H} NMR** (101 MHz, $CDCl_3$) δ 187.0, 162.6 (d, *J* = 263.6 Hz), 162.2, 161.0 (d, *J* = 5.4 Hz), 147.0, 135.8, 135.7, 128.7, 128.65, 128.62, 114.8 (d, *J* = 10.0 Hz), 109.7 (d, *J* = 20.8 Hz), 108.6 (d, *J* = 3.9 Hz), 78.8, 62.3, 59.9, 14.1. **mp:** 75.1 - 78.8°C. **IR $\nu_{max}$**: 2995, 2941, 2882, 1695, 1610, 1470, 1384, 1326, 1232, 1164, 1078, 991, 866, 829, 784, 753,733, 711, 694, 673 $cm^{-1}$. **HRMS** (ESI Q-TOF) m/z: [$(M+Na)^+$] calcd. for $C_{19}H_{18}FNO_5Na$ 382.1067 found: 382.1066.

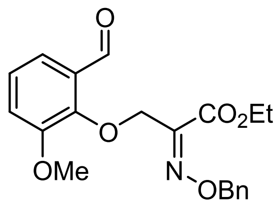


*ethyl (Z)-2-((benzyloxy)imino)-3-((1-formylnaphthalen-2-yl)oxy)propanoate* (**3m**). The synthesis was carried out according to **GP 3 c)**. A scale of 0.87 mmol, white solid, 0.12 g (0.30 mmol), isolated yield of 35%. **$^{1}$H NMR** (400 MHz, $CDCl_3$) δ 10.31 (d, *J* = 0.9 Hz, 1H), 7.37 (dd, *J* = 7.6, 1.8 Hz, 1H), 7.35 - 7.32 (m, 3H), 7.28 - 7.26 (m, 2H), 7.12 - 7.08 (m, 1H), 7.06 (dd, *J* = 8.2, 1.8 Hz, 1H), 5.25 (s, 2H), 5.12 (s, 2H), 4.33 (q, *J* = 7.0 Hz, 2H), 3.78 (s, 3H), 1.33 (t, *J* = 7.2 Hz, 3H). **$^{13}$C{$^{1}$H} NMR** (101 MHz, $CDCl_3$) δ 190.1, 162.7, 152.7, 151.0, 148.0, 135.8, 129.9, 128.5, 128.5, 128.4, 124.4, 119.0, 117.9, 78.4, 63.4, 62.1, 55.9, 14.1. **mp:** 56.1 - 57.6°C. **IR $\nu_{max}$**: 3035, 3021, 2986, 2939, 2888, 2837, 1685, 1583, 1479, 1455, 1437, 1375, 1325, 1251, 1215, 1147, 1066, 1010, 967, 909, 877, 824, 785, 755, 715, 694, 658 cm$^{-1}$. **HRMS** (ESI Q-TOF) m/z: [(M+H)$^{+}$] calcd. for $C_{20}H_{22}NO_6$ 372.1447 found: 372.1444.

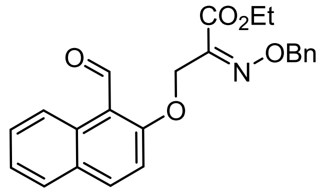


*ethyl (Z)-2-((benzyloxy)imino)-3-(2-formyl-6-methoxyphenoxy)propanoate* (**3n**). The synthesis was carried out according to **GP 3 c)**. A scale of 0.99 mmol, yellow solid, 0.20 g (0.55 mmol), isolated yield of 55%. **$^{1}$H NMR** (400 MHz, $CDCl_3$) δ 10.76 (s, 1H), 9.24 (dd, *J* = 8.8, 0.9 Hz, 1H), 7.96 (d, *J* = 9.1 Hz, 1H), 7.76 (d, *J* = 7.6 Hz, 1H), 7.62 (ddd, *J* = 8.7, 6.9, 1.5 Hz, 1H), 7.43 (ddd, *J* = 8.1, 6.9, 1.2 Hz, 2H), 7.34 (s, 5H), 7.29 (d, *J* = 9.1 Hz, 1H), 5.36 (s, 2H), 5.20 (s, 2H), 4.35 (q, *J* = 7.0 Hz, 2H), 1.34 (t, *J* = 7.0 Hz, 3H). **$^{13}$C{$^{1}$H} NMR** (101 MHz, $CDCl_3$) δ 191.9, 162.7, 162.3, 147.2, 137.3, 135.7, 131.4, 129.8, 129.1, 128.6, 128.2, 125.1, 117.9, 114.1, 78.8, 62.3, 60.3, 14.1. **mp:** 92.2 - 93.8°C. **IR $\nu_{max}$**: 2996, 2965, 2944, 2933, 2884, 1707, 1673, 1590, 1510, 1449, 1436, 1344, 1326, 1244, 1232, 1213, 1168, 1145, 988, 883, 859, 810, 748, 693, 662 cm$^{-1}$. **HRMS** (ESI Q-TOF) m/z: [(M+H)$^{+}$] calcd. for $C_{23}H_{22}NO_5$ 392.1498 found: 392.1496.

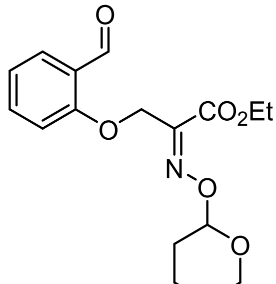


*ethyl (Z)-3-(2-formylphenoxy)-2-(((tetrahydro-2H-pyran-2-yl)oxy)imino)propanoate* (**3o**). The synthesis was carried out according to **GP 3 c)**. A scale of 5 mmol, yellow oil, 0.46 g (1.37 mmol), isolated yield of 27%. **$^{1}$H NMR** (400 MHz, $CDCl_3$) δ 10.41 (d, *J* = 0.9 Hz, 1H), 7.83 (dd, *J* = 7.8, 1.6 Hz, 1H), 7.55 (dd, *J* = 7.3, 2.1 Hz, 1H), 7.10 - 7.05 (m, 2H), 5.50 (t, *J* =

3.7 Hz, 1H), 5.13 (s, 2H), 4.39 - 4.30 (m, 2H), 3.85 - 3.79 (m, 1H), 3.69 - 3.64 (m, 1H), 1.84 - 1.57 (m, 6H), 1.33 (t, *J* = 7.0 Hz, 3H). **$^{13}$C{$^1$H} NMR** (101 MHz, $CDCl_3$) δ 189.4, 162.4, 160.7, 148.5, 135.8, 128.3, 125.5, 121.7, 113.1, 102.6, 63.1, 62.4, 59.6, 28.4, 24.8, 19.2, 14.1. **IR $\nu_{max}$**: 2945, 2870, 1718, 1687, 1598, 1482, 1456, 1394, 1372, 1329, 1285, 1232, 1205, 1187, 1149, 1128, 1117, 1102, 1040, 1016, 946, 895, 871, 819, 759 cm$^{-1}$. **HRMS** (ESI Q-TOF) m/z: [(M+H)$^+$] calcd. for $C_{17}H_{22}NO_6$ 336.1442 found: 336.1447.

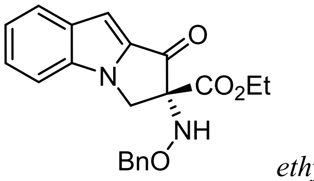


*ethyl (R)-2-((benzyloxy)amino)-1-oxo-2,3-dihydro-1H-pyrrolo[1,2-a]indole-2-carboxylate* (**2a**). The synthesis was carried out according to **GP 4**. Reaction conditions: 24 h, rt. A scale of 0.05 mmol, orange oil, 18.20 mg (0.049 mmol), isolated yield of 99%. **$^1$H NMR** (700 MHz, $CDCl_3$) δ 7.81 - 7.80 (m, 1H), 7.46 - 7.41 (m, 2H), 7.33 - 7.31 (m, 3H), 7.28 - 7.26 (m, 2H), 7.26 - 7.23 (m, 1H), 7.15 (d, *J* = 0.9 Hz, 1H), 6.88 (s, 1H), 5.00 (d, *J* = 11.2 Hz, 1H), 4.74 (dd, *J* = 15.9, 12.5 Hz, 2H), 4.31 (d, *J* = 11.2 Hz, 1H), 4.26 (q, *J* = 7.2 Hz, 2H), 1.26 (t, *J* = 7.1 Hz, 3H). **$^{13}$C{$^1$H} NMR** (101 MHz, $CDCl_3$) δ 185.6, 166.7, 136.9, 135.8, 132.8, 132.1, 128.7, 128.6, 128.5, 128.2, 126.0, 124.4, 121.9, 110.8, 101.9, 78.4, 62.8, 48.2, 14.0. **IR $\nu_{max}$**: 3168, 3062, 3010, 2982, 2928, 2907, 2874, 1712, 1642, 1534, 1454, 1368, 1329, 1269, 1165, 1135, 1095, 1008, 807, 738, 697, 469, 434 cm$^{-1}$. **HPLC** (Phenomenex Lux Amylose-1, 3μm, *n*-hexane:*i*-PrOH 90:10, 1 mL/min, det. UV 254 nm): Rt= 16.5 min (enantiomer *R*), 18.8 min (enantiomer *S*); *ee* 96%, $[\alpha]_D{}^{20}$ = -26.85 (c 1.0, $CHCl_3$). **HRMS** (ESI Q-TOF) m/z: [(M+H)$^+$] calcd. for $C_{21}H_{21}N_2O_4$ 365.1501 found: 365.1497.

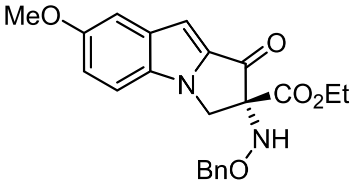


*ethyl (R)-2-((benzyloxy)amino)-7-methoxy-1-oxo-2,3-dihydro-1H-pyrrolo[1,2-a]indole-2-carboxylate* (**2b**). The synthesis was carried out according to **GP 4**. Reaction conditions: 24h, rt. A scale of 0.1 mmol, orange oil, 39.15 mg (0.099 mmol), isolated yield of 99%. **$^1$H NMR** (400 MHz, $CDCl_3$) δ 7.30 - 7.28 (m, 3H), 7.27 - 7.24 (m, 3H), 7.12 - 7.09 (m, 2H), 7.02 (d, *J* = 0.9 Hz, 1H), 6.87 (s, 1H), 4.94 (d, *J* = 11.2 Hz, 1H), 4.72 (d, *J* = 1.2 Hz, 2H), 4.24 (q, *J* = 7.1 Hz, 2H), 4.24 (d, *J* = 11.2 Hz, 1H), 3.87 (s, 3H), 1.24 (t, *J* = 7.0 Hz, 3H). **$^{13}$C{$^1$H} NMR** (101 MHz, $CDCl_3$) δ 184.9, 166.8, 155.5, 137.0, 133.1, 132.6, 131.7, 128.6, 128.5, 128.1, 119.0, 111.8, 102.9, 101.1, 78.5, 62.8, 55.6, 48.3, 25.3, 14.0. **IR $\nu_{max}$**: 2963, 2935, 2837, 1709, 1625, 1532, 1454, 1394, 1366, 1321, 1292, 1264, 1240, 1211, 1175, 1025, 968, 936, 844, 802, 735, 698, 652, 604, 476, 434 cm$^{-1}$. **HPLC** (Phenomenex Lux Amylose-1, 3μm, *n*-hexane:*i*-PrOH 90:10, 1 mL/min, det. UV 254 nm): Rt= 23.6 min (enantiomer *R*), 33.6 min (enantiomer *S*); *ee* 96%, $[\alpha]_D{}^{20}$ = -31.16 (c 1.0, $CHCl_3$). **HRMS** (ESI Q-TOF) m/z: [(M+H)$^+$] calcd. for $C_{22}H_{23}N_2O_5$ 395.1607 found: 395.1605.

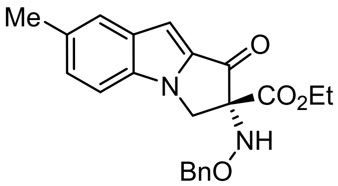

*ethyl (R)-2-((benzyloxy)amino)-7-methyl-1-oxo-2,3-dihydro-1H-pyrrolo[1,2-a]indole-2-carboxylate* (**2c**). The synthesis was carried out according to **GP 4**. Reaction conditions: 24 h, rt, then 24 h 40°C. A scale of 0.1 mmol, orange oil, 37.38 mg (0.099 mmol), isolated yield of 99%. **$^{1}$H NMR** (700 MHz, $CDCl_3$) δ 7.56 (s, 1H), 7.32 - 7.30 (m, 4H), 7.27 - 7.26 (m, 3H), 7.04 (s, 1H), 6.86 (s, 1H), 4.97 (d, *J* = 11.2 Hz, 1H), 4.73 (d, *J* = 3.9 Hz, 2H), 4.25 (q, *J* = 7.0 Hz, 2H), 2.49 (s, 3H), 1.25 (t, *J* = 7.1 Hz, 3H). **$^{13}$C{$^{1}$H} NMR** (101 MHz, $CDCl_3$) δ 185.4, 166.8, 137.0, 134.4, 132.9, 132.4, 131.4, 128.6, 128.5, 128.3, 128.1, 123.3, 110.5, 101.2, 78.5, 62.8, 48.2, 21.6, 14.0. **IR $\nu_{max}$**: 2918, 2860, 1742, 1711, 1536, 1386, 1320, 1295, 1256, 1200, 1178, 1124, 1108, 1070, 1011, 967, 853, 792, 734, 697, 644 $cm^{-1}$. **HPLC** (Phenomenex Lux Amylose-1, 3μm, *n*-hexane:*i*-PrOH 90:10, 1 mL/min, det. UV 254 nm): Rt= 17.5 min (enantiomer *R*), 26.8 min (enantiomer *S*); *ee* 96%, $[\alpha]_D{}^{20}$ = -35.65 (c 1.0, $CHCl_3$). **HRMS** (ESI Q-TOF) m/z: [$(M+H)^+$] calcd. for $C_{22}H_{23}N_2O_4$ 379.1658 found: 379.1656.

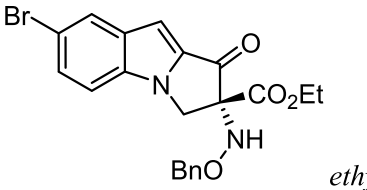

*ethyl (R)-2-((benzyloxy)amino)-7-bromo-1-oxo-2,3-dihydro-1H-pyrrolo[1,2-a]indole-2-carboxylate* (**2d**). The synthesis was carried out according to **GP 4**. Reaction conditions: 3 h 40°C. A scale of 0.1 mmol, orange oil, 42.31 mg (0.095 mmol), isolated yield of 95%. **$^{1}$H NMR** (400 MHz, $CDCl_3$) δ 7.92 (d, *J* = 1.5 Hz, 1H), 7.48 (dd, *J* = 8.8, 1.8 Hz, 1H), 7.29 (dd, *J* = 5.0, 2.1 Hz, 2H), 7.25 (dd, *J* = 8.2, 1.3 Hz, 2H), 7.23 - 7.22 (m, 1H), 7.03 (d, *J* = 0.9 Hz, 1H), 6.85 (s, 1H), 4.94 (d, *J* = 11.2 Hz, 1H), 4.70 (s, 2H), 4.24 (q, *J* = 7.0 Hz, 2H), 4.21 (d, *J* = 11.2 Hz, 1H), 1.24 (t, *J* = 7.0 Hz, 3H). **$^{13}$C{$^{1}$H} NMR** (101 MHz, $CDCl_3$) δ 185.6, 166.4, 136.9, 134.1, 133.7, 133.3, 129.1, 128.7, 128.6, 128.5, 128.2, 126.6, 115.1, 112.3, 101.0, 78.4, 63.0, 48.4, 14.0. **IR $\nu_{max}$**: 3063, 2958, 2925, 2870, 1714, 1531, 1454, 1381, 1319, 1263, 1215, 1194, 1141, 1107, 1068, 1044, 1011, 967, 898, 866, 792, 733, 697, 637, 451, 429 $cm^{-1}$. **HPLC** (Phenomenex Lux Amylose-1, 3μm, *n*-hexane:*i*-PrOH 90:10, 1 mL/min, det. UV 254 nm): Rt= 19.8 min (enantiomer *R*), 28.3 min (enantiomer *S*); *ee* 94%, $[\alpha]_D{}^{20}$ = -23.78 (c 1.0, $CHCl_3$). **HRMS** (ESI Q-TOF) m/z: [$(M+Na)^+$] calcd. for $C_{21}H_{19}BrN_2O_4Na$ 465.0426 found: 465.0425.

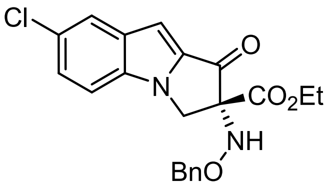

*ethyl (R)-2-((benzyloxy)amino)-7-chloro-1-oxo-2,3-dihydro-1H-pyrrolo[1,2-a]indole-2-carboxylate* (**2e**). The synthesis was carried out according to **GP 4**. Reaction conditions: 24 h 40°C. A scale of 0.1 mmol, orange oil, 39.44 mg (0.099 mmol), isolated yield of 99%.

**$^1$H NMR** (400 MHz, $CDCl_3$) δ 7.75 (d, *J* = 1.5 Hz, 1H), 7.36 (dd, *J* = 9.1, 1.9 Hz, 1H), 7.31 - 7.28 (m, 4H), 7.24 - 7.22 (m, 2H), 7.04 (d, *J* = 0.9 Hz, 1H), 6.85 (s, 1H), 4.94 (d, *J* = 11.2 Hz, 1H), 4.70 (s, 2H), 4.24 (q, *J* = 7.1 Hz, 2H), 4.22 (d, *J* = 11.2 Hz, 1H), 1.24 (t, *J* = 7.0 Hz, 3H). **$^{13}C\{^1H\}$ NMR** (101 MHz, $CDCl_3$) δ 185.6, 166.4, 136.9, 134.0, 133.8, 132.7, 128.6, 128.5, 128.2, 127.6, 127.0, 126.7, 123.3, 112.0, 101.1, 78.4, 63.0, 48.4, 14.0. **IR $v_{max}$**: 3252, 3032, 2981, 2928, 1716, 1533, 1454, 1340, 1320, 1264, 1214, 1158, 1141, 1107, 1058, 1011, 994, 970, 906, 867, 795, 729, 697, 641, 430 $cm^{-1}$. **HPLC** (Phenomenex Lux Amylose-1, 3µm, *n*-hexane:*i*-PrOH 90:10, 1 mL/min, det. UV 254 nm): Rt= 18.9 min (enantiomer *R*), 24.7 min (enantiomer *S*); *ee* 94%, $[\alpha]_D{}^{20}$ = -26.31 (c 1.0, $CHCl_3$). **HRMS** (ESI Q-TOF) m/z: [$(M+Na)^+$] calcd. for $C_{21}H_{19}ClN_2O_4Na$ 421.0931 found: 421.0929.

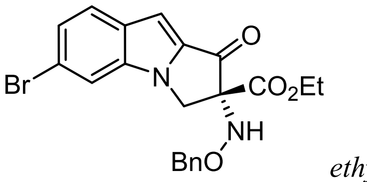


*ethyl (R)-2-((benzyloxy)amino)-6-bromo-1-oxo-2,3-dihydro-1H-pyrrolo[1,2-a]indole-2-carboxylate* (**2f**). The synthesis was carried out according to **GP 4**. Reaction conditions: 24 h, rt. A scale of 0.1 mmol, orange oil, 42.60 mg (0.096 mmol), isolated yield of 96%. **$^1$H NMR** (400 MHz, $CDCl_3$) δ 7.66 (dd, *J* = 8.8, 0.6 Hz, 1H), 7.57 - 7.57 (m, 1H), 7.35 - 7.31 (m, 4H), 7.26 - 7.24 (m, 2H), 7.10 (d, *J* = 1.2 Hz, 1H), 4.93 (d, *J* = 11.2 Hz, 1H), 4.72 (s, 2H), 4.26 (q, *J* = 7.0 Hz, 2H), 4.23 (d, *J* = 11.2 Hz, 1H), 1.26 (t, *J* = 7.0 Hz, 3H). **$^{13}C\{^1H\}$ NMR** (101 MHz, $CDCl_3$) δ 185.5, 166.4, 136.8, 136.1, 133.3, 130.7, 128.7, 128.6, 128.5, 128.3, 125.5, 125.5, 120.0, 113.9, 102.0, 78.4, 63.0, 48.3, 14.0. **IR $v_{max}$**: 2978, 2962, 2922, 2873, 2857, 1714, 1607, 1530, 1468, 1391, 1340, 1320, 1290, 1257, 1216, 1151, 1107, 1070, 1041, 977, 819, 733, 697, 640, 620, 588, 429 $cm^{-1}$. **HPLC** (Phenomenex Lux Cellulose-1, 3µm, *n*-hexane:*i*-PrOH 95:5, 0.8 mL/min, det. UV 254 nm): Rt= 15.4 min (enantiomer *R*), 17.1 min (enantiomer *S*); *ee* 96%, $[\alpha]_D{}^{20}$ = -29.94 (c 1.0, $CHCl_3$). **HRMS** (ESI Q-TOF) m/z [$(M+Na)^+$] calc. for $C_{21}H_{19}BrN_2O_4Na$ 465.0426; found: 465.0423.

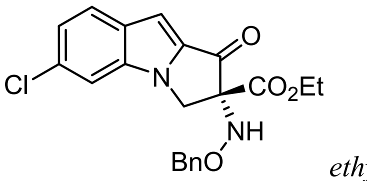


*ethyl (R)-2-((benzyloxy)amino)-6-chloro-1-oxo-2,3-dihydro-1H-pyrrolo[1,2-a]indole-2-carboxylate* (**2g**). The synthesis was carried out according to **GP 4**. Reaction conditions: 24 h, rt. A scale of 0.1 mmol, orange oil, 38.74 mg (0.097 mmol), isolated yield of 97%. **$^1$H NMR** (400 MHz, $CDCl_3$) δ 7.69 (dd, *J* = 8.8, 0.6 Hz, 1H), 7.37 - 7.37 (m, 1H), 7.32 - 7.26 (m, 3H), 7.25 - 7.22 (m, 2H), 7.18 (dd, *J* = 8.8, 1.8 Hz, 1H), 7.08 (d, *J* = 0.9 Hz, 1H), 6.86 (s, 1H), 4.91 (d, *J* = 11.2 Hz, 1H), 4.70 (s, 2H), 4.24 (q, *J* = 7.0 Hz, 2H), 4.21 (d, *J* = 11.4 Hz, 1H), 1.24 (t, *J* = 7.0 Hz, 3H). **$^{13}C\{^1H\}$ NMR** (101 MHz, $CDCl_3$) δ 185.3, 166.4, 136.8, 135.7, 133.5, 132.2, 130.4, 128.7, 128.5, 128.3, 125.3, 123.0, 110.7, 102.0, 78.4, 77.1, 63.0, 48.3, 14.0. **IR $v_{max}$**: 3032, 2982, 2938, 2808, 1717, 1660, 1478, 1454, 1405, 1369, 1301, 1243, 1218, 1176, 1134, 1077, 1009, 954, 764, 741, 697, 608 $cm^{-1}$. **HPLC** (Phenomenex Lux Amylose-1, 3µm, *n*-hexane:*i*-PrOH 95:5, 0.8 mL/min, det. UV 254 nm): Rt= 28.9

min (enantiomer *R*), 30.1 min (enantiomer *S*); *ee* 94%, $[\alpha]_D^{20}$ = -36.43 (c 1.0, $CHCl_3$). **HRMS** (ESI Q-TOF) m/z $[(M+Na)^+]$ calc. for $C_{21}H_{19}ClN_2O_4Na$ 421.0931; found: 421.0927.

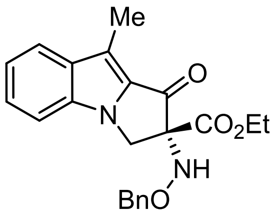

*ethyl (R)-2-((benzyloxy)amino)-9-methyl-1-oxo-2,3-dihydro-1H-pyrrolo[1,2-a]indole-2-carboxylate carboxylate* (**2h**). The synthesis was carried out according to **GP 4**. Reaction conditions: 24 h, rt, then 24 h 40°C. A scale of 0.1 mmol, orange oil, 26.00 mg (0.07 mmol), isolated yield of 70% after flash column chromatography (*n*-hexane:EtOAc gradient 0→10% EtOAc). **$^1$H NMR** (400 MHz, $CDCl_3$) δ 7.75 (dt, *J* = 8.2, 1.0 Hz, 1H), 7.43 (ddd, *J* = 8.5, 6.8, 1.2 Hz, 1H), 7.35 (dt, *J* = 8.5, 0.9 Hz, 2H), 7.33 - 7.30 (m, 5H), 7.21 (ddd, *J* = 8.2, 6.9, 1.0 Hz, 1H), 4.94 (d, *J* = 10.9 Hz, 1H), 4.74 (d, *J* = 1.8 Hz, 2H), 4.30 - 4.22 (m, 3H), 2.60 (s, 3H), 1.27 (t, *J* = 7.0 Hz, 3H). **$^{13}$C{$^1$H} NMR** (101 MHz, $CDCl_3$) δ 185.5, 167.0, 137.0, 135.7, 132.3, 129.9, 128.6, 128.5, 128.1, 126.2, 122.4, 120.8, 115.6, 110.8, 78.8, 62.7, 47.8, 29.7, 14.0, 9.11. **IR $v_{max}$**: 3254, 2979, 2958, 2919, 2869, 1742, 1706, 1564, 1379, 1367, 1336, 1314, 1263, 1246, 1209, 1186, 1152, 1014, 935, 915, 738, 697, 434 $cm^{-1}$. **HPLC** (Phenomenex Lux Amylose-1, 3μm, *n*-hexane:*i*-PrOH 90:10, 1 mL/min, det. UV 254 nm): Rt= 13.9 min (enantiomer *S*), 14.9 min (enantiomer *R*); *ee* 97%, $[\alpha]_D^{20}$ = -21.86 (c 1.0, $CHCl_3$). **HRMS** (ESI Q-TOF) m/z $[(M+H)^+]$ calc. for $C_{22}H_{23}N_2O_4$ 379.1658; found: 379.1655.

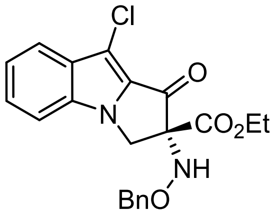

*ethyl (R)-2-((benzyloxy)amino)-9-chloro-1-oxo-2,3-dihydro-1H-pyrrolo[1,2-a]indole-2-carboxylate* (**2i**). The synthesis was carried out according to **GP 4**. Reaction conditions: 24 h, rt. A scale of 0.1 mmol, orange oil, 19.2 mg (0.048 mmol), isolated yield of 48% after flash colum chromatography chromatography (*n*-hexane:EtOAc gradient 0→10% EtOAc). **$^1$H NMR** (400 MHz, $CDCl_3$) δ 7.78 (dt, *J* = 8.2, 1.0 Hz, 1H), 7.45 (ddd, *J* = 8.5, 7.0, 1.2 Hz, 1H), 7.34 (dt, *J* = 8.5, 1.0 Hz, 1H), 7.31 - 7.29 (m, 3H), 7.29 - 7.27 (m, 2H), 7.26 - 7.24 (m, 1H), 4.92 (d, *J* = 11.2 Hz, 1H), 4.70 (s, 2H), 4.29 - 4.22 (m, 2H), 4.20 (d, *J* = 10.9 Hz, 1H), 1.25 (t, *J* = 7.2 Hz, 3H). **$^{13}$C{$^1$H} NMR** (176 MHz, $CDCl_3$) δ 183.7, 166.3, 136.9, 134.9, 129.8, 128.7, 128.5, 128.2, 128.0, 127.0, 122.3, 121.5, 111.1, 105.9, 78.7, 63.0, 48.3, 14.0. **IR $v_{max}$**: 3061, 3030, 2981, 2926, 2872, 1744, 1714, 1537, 1468, 1454, 1390, 1377, 1349, 1329, 1315, 1261, 1238, 1199, 1165, 1126, 1012, 950, 738, 697 $cm^{-1}$. **HPLC** (Phenomenex Lux Amylose-1, 3μm, *n*-hexane:*i*-PrOH 90:10, 1 mL/min, det. UV 254 nm): Rt= 14.3 min (enantiomer *S*), 15.1 min (enantiomer *R*); *ee* 99%, $[\alpha]_D^{20}$ = -38.27 (c 1.0, $CHCl_3$). **HRMS** (ESI Q-TOF) m/z $[(M+Na)^+]$ calc. for $C_{21}H_{19}ClN_2O_4Na$ 421.0931; found: 421.0930.

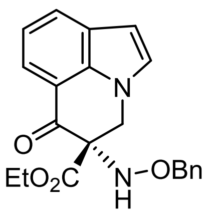

*ethyl (R)-5-((benzyloxy)amino)-6-oxo-5,6-dihydro-4H-pyrrolo[3,2,1-ij]quinoline-5-carboxylate* (**2j**). The synthesis was carried out according to **GP 4**. Reaction conditions: 24 h, rt, then 24 h 40°C. A scale of 0.041 mmol, yellow oil, 5.00 mg (0.014 mmol), isolated yield of 33% after flash column chromatography (*n*-hexane:EtOAc gradient 0→10% EtOAc). **$^1$H NMR** (400 MHz, $CDCl_3$) δ 7.86 (dd, *J* = 7.9, 1.0 Hz, 1H), 7.72 - 7.70 (m, 1H), 7.36 - 7.31 (m, 5H), 7.21 - 7.16 (m, 2H), 6.60 (d, *J* = 2.9 Hz, 1H), 5.00 (d, *J* = 12.6 Hz, 1H), 4.71 (dd, *J* = 15.6, 11.7 Hz, 2H), 4.44 (d, *J* = 12.6 Hz, 1H), 4.21 - 4.13 (m, 1H), 4.13 - 4.05 (m, 1H), 1.12 (t, *J* = 7.2 Hz, 3H). **$^{13}$C{$^1$H} NMR** (176 MHz, $CDCl_3$) δ 185.9, 167.1, 138.3, 137.0, 128.7, 128.4, 128.3, 128.1, 127.7, 120.3, 119.8, 115.5, 102.8, 77.3, 72.1, 62.4, 48.5, 29.7, 13.9. **IR $\nu_{max}$**: 3063, 3031, 2980, 2924, 2871, 1736, 1684, 1589, 1509, 1469, 1453, 1347, 1230, 1199, 1169, 1094, 1017, 907, 895, 858, 797, 730, 697, 607, 560 cm$^{-1}$. **HPLC** (Phenomenex Lux Amylose-1, 3µm, *n*-hexane:*i*-PrOH 90:10, 1 mL/min, det. UV 254 nm): Rt= 13.0 min (enantiomer *R*), 14.0 min (enantiomer *S*); *ee* 88%, $[\alpha]_D^{20}$ = -54.28 (c 1.0, $CHCl_3$). **HRMS** (ESI Q-TOF) m/z $[(M+H)^+]$ calc. for $C_{21}H_{19}ClN_2O_4Na$ 421.0931; found: 421.0930.

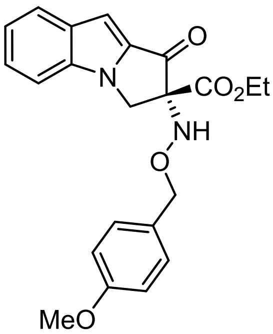

*ethyl (R)-2-(((4-methoxybenzyl)oxy)amino)-1-oxo-2,3-dihydro-1H-pyrrolo[1,2-a]indole-2-carboxylate* (**2n**). The synthesis was carried out according to **GP 4**. Reaction conditions: 24 h 40°C. A scale of 0.1 mmol, orange oil, 30.20 mg (0.077 mmol), isolated yield of 77% after flash column chromatography (*n*-hexane:EtOAc gradient 0→10% EtOAc). **$^1$H NMR** (400 MHz, $CDCl_3$) δ 7.81 - 7.79 (m, 1H), 7.44 (ddd, *J* = 8.5, 6.5, 1.2 Hz, 1H), 7.39 (dd, *J* = 8.5, 2.1, 0.9 Hz, 1H), 7.24 (ddd, *J* = 8.2, 6.8, 1.2 Hz, 1H), 7.19 (d, *J* = 8.8 Hz, 2H), 7.14 (d, *J* = 0.9 Hz, 1H), 6.83 - 6.81 (m, 2H), 4.96 (d, *J* = 11.2 Hz, 1H), 4.66 (s, 2H), 4.25 (m, 4H), 3.80 (s, 3H), 1.25 (t, *J* = 7.0 Hz, 3H). **$^{13}$C{$^1$H} NMR** (101 MHz, $CDCl_3$) δ 185.6, 166.8, 159.6, 135.8, 132.9, 132.1, 130.3, 129.1, 125.9, 124.4, 121.8, 113.8, 110.8, 101.8, 78.4, 76.6, 62.8, 55.2, 48.2, 14.0. **IR $\nu_{max}$**: 2980, 2958, 2932, 2837, 1744, 1710, 1611, 1535, 1512, 1466, 1349, 1315, 1245, 1218, 1165,1136, 1071, 1030, 1011, 968, 845, 821, 788, 744, 637, 565, 507, 434 cm$^{-1}$. **HPLC** (Phenomenex Lux Amylose-1, 3µm, *n*-hexane:*i*-PrOH 90:10, 1 mL/min, det. UV 254 nm): Rt= 21.3 min (enantiomer *R*), 27.2 min (enantiomer *S*); *ee* 98%, $[\alpha]_D^{20}$ = -26.53 (c 1.0, $CHCl_3$). **HRMS** (ESI Q-TOF) m/z $[(M+H)^+]$ calc. for $C_{22}H_{23}N_2O_5$ 395.1607; found: 395.1611.

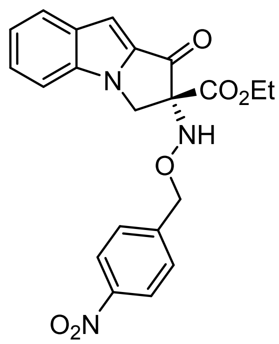


*ethyl (R)-2-(((4-nitrobenzyl)oxy)amino)-1-oxo-2,3-dihydro-1H-pyrrolo[1,2-a]indole-2-carboxylate* (**2o**). The synthesis was carried out according to **GP 4**. Reaction conditions: 24 h rt, then 24 h 40°C. A scale of 0.046 mmol, white solid, 5.00 mg (0.012 mmol), isolated yield of 26% after flash column chromatography (*n*-hexane:EtOAc gradient 0→10% EtOAc). **$^1$H NMR** (700 MHz, $CDCl_3$) δ 8.16 - 8.15 (m, 2H), 7.82 - 7.81 (m, 1H), 7.45 (ddd, *J* = 8.2, 6.5, 1.0 Hz, 1H), 7.42 - 7.41 (m, 3H), 7.26 (ddd, *J* = 8.2, 6.9, 0.9 Hz, 1H), 7.17 (d, *J* = 0.9 Hz, 1H), 6.96 (s, 1H), 5.06 (d, *J* = 11.2 Hz, 1H), 4.84 (d, *J* = 5.2 Hz, 2H), 4.38 (d, *J* = 10.8 Hz, 1H), 4.28 (q, *J* = 7.2 Hz, 2H), 1.26 (t, *J* = 7.1 Hz, 3H). **$^{13}C\{^1H\}$ NMR** (176 MHz, $CDCl_3$) δ 185.1, 166.4, 147.8, 144.1, 135.8, 132.7, 132.1, 128.7, 126.3, 124.5, 123.7, 122.1, 110.7, 102.2, 78.4, 75.8, 63.0, 48.2, 14.0. **mp**: 115.4 - 118.1°C. **IR $v_{max}$**: 2925, 2854, 1725, 1702, 1537, 1515, 1346, 1315, 1259, 1229, 1197, 1165, 1108, 1050, 1017, 975, 859, 849, 822, 808, 742, 732, 692, 638 $cm^{-1}$. **HPLC** (Phenomenex Lux Amylose-1, 3µm, *n*-hexane:*i*-PrOH 70:30, 1 mL/min, det. UV 254 nm): Rt= 21.3 min (enantiomer *R*), 27.1 min (enantiomer *S*); *ee* 90%, $[\alpha]_D{}^{20}$ = -35.17 (c 1.0, $CHCl_3$). **HRMS** (ESI Q-TOF) m/z $[(M+H)^+]$ calc. for $C_{21}H_{20}N_3O_6$ 410.1352; found: 410.1349.

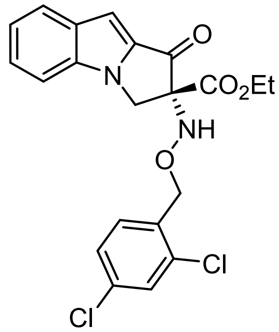


*ethyl (R)-2-(((2,4-dichlorobenzyl)oxy)amino)-1-oxo-2,3-dihydro-1H-pyrrolo[1,2-a]indole-2-carboxylate* (**2p**). The synthesis was carried out according to **GP 4**. Reaction conditions: 24 h rt, then 24 h 40°C. A scale of 0.1 mmol, yellow oil, 27.00 mg (0.062 mmol), isolated yield of 62% after flash column chromatography (*n*-hexane:EtOAc gradient 0→10% EtOAc). **$^1$H NMR** (400 MHz, $CDCl_3$) δ 7.81 (dt, *J* = 8.2, 0.9 Hz, 1H), 7.46 - 7.44 (m, 2H), 7.34 (d, *J* = 2.1 Hz, 1H), 7.27 - 7.22 (m, 2H), 7.16 (dd, *J* = 8.2, 2.1 Hz, 1H), 7.16 (s, 1H), 5.07 (d, *J* = 11.2 Hz, 1H), 4.83 (s, 2H), 4.42 (d, *J* = 11.4 Hz, 1H), 4.28 (q, *J* = 7.1 Hz, 2H), 1.27 (t, *J* = 7.0 Hz, 3H). **$^{13}C\{^1H\}$ NMR** (101 MHz, $CDCl_3$) δ 185.4, 166.5, 135.8, 134.6, 134.5, 133.0, 132.7, 132.1, 131.1, 129.4, 127.1, 126.2, 124.5, 122.0, 110.8, 102.1, 78.4, 73.7, 63.0, 48.2, 14.0. **IR $v_{max}$**: 2979, 2927, 1743, 1711, 1535, 1471, 1379, 1349, 1315, 1261, 1217, 1197, 1165, 1135, 1104, 1012, 969, 847, 815, 787, 737, 639, 434 $cm^{-1}$. **HPLC** (Phenomenex Lux Amylose-1, 3µm, *n*-hexane:*i*-PrOH 90:10, 1 mL/min, det. UV 254 nm): Rt= 18.9 min (enantiomer

*R*), 25.1 min (enantiomer *S*); *ee* 96%, $[\alpha]_D^{20}$ = -29.81 (c 1.0, $CHCl_3$). **HRMS** (ESI Q-TOF) m/z $[(M+Na)^+]$ calc. for $C_{21}H_{18}Cl_2N_2O_4Na$ 455.0542; found: 455.0540.

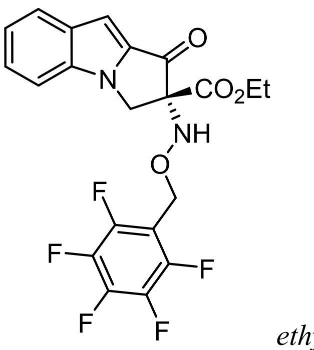


*ethyl (R)-1-oxo-2-(((perfluorophenyl)methoxy)amino)-2,3-dihydro-1H-pyrrolo[1,2-a]indole-2-carboxylate* (**2q**). The synthesis was carried out according to **GP 4**. Reaction conditions: 24 h rt. A scale of 0.1 mmol, white solid, 40.00 mg (0.088 mmol), isolated yield of 88% after flash column chromatography (*n*-hexane:EtOAc gradient 0→10% EtOAc). **$^1$H NMR** (400 MHz, $CDCl_3$) δ 7.81 (dt, *J* = 8.3, 1.0 Hz, 1H), 7.48 - 7.47 (m, 2H), 7.26 (ddd, *J* = 7.9, 5.6, 2.6 Hz, 1H), 7.15 (s, 1H), 6.99 (s, 1H), 5.10 (d, *J* = 11.2 Hz, 1H), 4.85 (t, *J* = 1.6 Hz, 2H), 4.44 (d, *J* = 11.2 Hz, 1H), 4.27 (q, *J* = 7.0 Hz, 2H), 1.26 (t, *J* = 7.2 Hz, 3H). **$^{13}$C{$^1$H} NMR** (101 MHz, $CDCl_3$) δ 184.6, 166.4, 135.8, 132.5, 132.1, 126.3, 124.5, 122.0, 110.7, 102.3, 78.4, 63.6, 63.0, 47.9, 14.0 (The description does not include signals from C-F carbon atoms in the $^{13}$C spectrum due to the multiplicity and low intensity). **IR $v_{max}$**: 2953, 2921, 1736, 1716, 1521, 1500, 1265, 1215, 1168, 1123, 1110, 1073, 1041, 1015, 935, 813, 746 cm$^{-1}$. **HPLC** (Phenomenex Lux Amylose-1, 3μm, *n*-hexane:*i*-PrOH 70:30, 1 mL/min, det. UV 254 nm): Rt= 7.9 min (enantiomer *R*), 26.2 min (enantiomer *S*); *ee* 98%, $[\alpha]_D^{20}$ = 31.72 (c 1.0, $CHCl_3$). **HRMS** (ESI Q-TOF) m/z $[(M+H)^+]$ calc. for $C_{21}H_{16}F_5N_2O_4$ 455.1030 found: 455.1025.

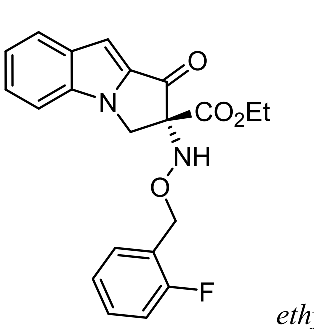


*ethyl (R)-2-(((2-fluorobenzyl)oxy)amino)-1-oxo-2,3-dihydro-1H-pyrrolo[1,2-a]indole-2-carboxylate* (**2r**). The synthesis was carried out according to **GP 4**. Reaction conditions: 24 h rt, then 24 h 40°C. A scale of 0.1 mmol, yellow oil, 25.13 mg (0.066 mmol), isolated yield of 66% after flash column chromatography (*n*-hexane:EtOAc gradient 0→10% EtOAc). **$^1$H NMR** (400 MHz, $CDCl_3$) δ 7.78 (dt, *J* = 8.3 Hz, 1H), 7.42 (dd, *J* = 4.4, 0.9 Hz, 2H), 7.30 - 7.25 (m, 2H), 7.22 (dt, *J* = 8.1, 4.0 Hz, 1H), 7.12 (s, 1H), 7.05 (td, *J* = 7.5, 1.2 Hz, 1H), 7.00 (ddd, *J* = 9.8, 8.7, 1.2 Hz, 1H), 6.98 (bs, 1H), 5.03 (d, *J* = 11.4 Hz, 1H), 4.80 (s, 2H), 4.39 (d, *J* = 11.2 Hz, 1H), 4.24 (q, *J* = 7.1 Hz, 2H), 1.24 (t, *J* = 7.0 Hz, 3H). **$^{13}$C{$^1$H} NMR** (101 MHz, $CDCl_3$) δ 185.4, 166.6, 161.1 (d, *J* = 248.2 Hz), 135.8, 132.8, 132.1, 131.0 (d, *J* = 3.9 Hz), 130.1 (d, *J* = 8.5 Hz), 126.0, 124.4, 124.0 (d, *J* = 3.9 Hz), 123.8 (d, *J* = 14.6 Hz), 121.9, 115.4 (d, *J* = 21.6 Hz), 110.8, 102.0, 78.5, 70.7 (d, *J* = 3.1 Hz), 62.9, 48.1, 14.0. **IR $v_{max}$**:

3254, 2970, 2930, 1743, 1710, 1535, 1490, 1392, 1378, 1349, 1315, 1262, 1226, 1195, 1165, 1135, 1108, 1012, 967, 744, 637, 433 cm$^{-1}$. **HPLC** (Phenomenex Lux Amylose-1, 3µm, *n*-hexane:*i*-PrOH 90:10, 1 mL/min, det. UV 254 nm): Rt= 16.5 min (enantiomer *R*), 23.7 min (enantiomer *S*); *ee* 94%, $[\alpha]_D^{20}$ = -33.19 (c 1.0, $CHCl_3$). **HRMS** (ESI Q-TOF) m/z [$(M+H)^+$] calc. for $C_{21}H_{20}FN_2O_4$ 383.1407 found: 383.1402.

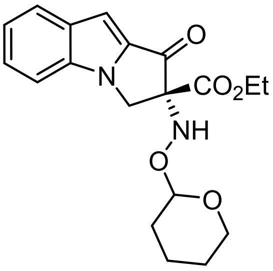


*ethyl (2R)-1-oxo-2-(((tetrahydro-2H-pyran-2-yl)oxy)amino)-2,3-dihydro-1H-pyrrolo[1,2-a]indole-2-carboxylate* (**2s**). The synthesis was carried out according to **GP 4**. Reaction conditions: 24 h 40°C. A scale of 0.13 mmol, diastereoisomers were separated and isolated as an orange oils, 34.09 mg (0.095 mmol) isolated yield for both diastereoisomers (*dr* 1:1) : 73% after flash column chromatography (*n*-hexane:EtOAc gradient 0→10% EtOAc). Analytical data for one diastereoisomer: **$^1$H NMR** (400 MHz, $CDCl_3$) δ 7.79 (d, *J* = 8.2 Hz, 1H), 7.49 (dd, *J* = 8.5, 0.6 Hz, 1H), 7.42 (ddd, *J* = 8.2, 7.0, 0.9 Hz, 1H), 7.22 (ddd, *J* = 7.9, 7.0, 0.9 Hz, 1H), 7.13 (s, 1H), 7.08 (s, 1H), 5.10 (d, *J* = 11.2 Hz, 1H), 4.85 - 4.84 (m, 1H), 4.63 (d, *J* = 10.9 Hz, 1H), 4.25 (qd, *J* = 7.1, 2.3 Hz, 2H), 3.92 - 3.87 (m, 1H), 3.62 - 3.58 (m, 1H), 1.60 - 1.49 (m, 4H), 1.44 - 1.40 (m, 2H), 1.25 (t, *J* = 7.2 Hz, 3H). **$^{13}$C{$^1$H} NMR** (101 MHz, $CDCl_3$) δ 186.4, 166.2, 135.8, 132.9, 132.0, 125.9, 124.4, 121.8, 110.9, 101.6, 101.3, 78.3, 62.9, 62.7, 48.3, 28.7, 25.1, 19.4, 14.0. **IR $\nu_{max}$**: 2941, 2868, 2852, 1745, 1711, 1534, 1349, 1316, 1261, 1201, 1164, 1135, 1106, 1077, 1063, 1036, 1018, 980, 964, 939, 908, 894, 869, 807, 785, 745, 636, 433 cm$^{-1}$. **HPLC** diastereoisomer 1: (Phenomenex Lux Amylose-1, 3µm, *n*-hexane:*i*-PrOH 95:5, 0.5 mL/min, det. 254 nm): Rt= 20.0 min, 31.5 min; *ee* >99%. diastereoisomer 2: (Phenomenex Lux Amylose-1, 3µm, *n*-hexane:*i*-PrOH 95:5, 0.5 mL/min, det. UV 254 nm): Rt= 21.3 min, 27.9 min; *ee* 95%, $[\alpha]_D^{20}$ = -16.11 (c 1.0, $CHCl_3$). **HRMS** (ESI Q-TOF) m/z [$(M+H)^+$] calc. for $C_{19}H_{23}N_2O_5$ 359.1607 found: 359.1601.

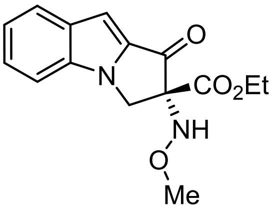


*ethyl (R)-2-(methoxyamino)-1-oxo-2,3-dihydro-1H-pyrrolo[1,2-a]indole-2-carboxylate* (**2t**). The synthesis was carried out according to **GP 4**. Reaction conditions: 24 h rt, then 24 h 40°C. A scale of 0.11 mmol, orange oil, 4.5 mg (0.016 mmol), isolated yield of 15% after flash column chromatography (*n*-hexane:EtOAc gradient 0→10% EtOAc). **$^1$H NMR** (400 MHz, $CDCl_3$) δ 7.79 (dt, *J* = 8.2, 0.9 Hz, 1H), 7.50 - 7.48 (m, 1H), 7.43 (ddd, *J* = 8.5, 6.8, 1.2 Hz, 1H), 7.24 - 7.20 (m, 1H), 7.13 (d, *J* = 0.9 Hz, 1H), 6.87 (s, 1H), 5.09 (d, *J* = 10.9 Hz, 1H), 4.51 (d, *J* = 11.2, 1H), 4.25 (qd, *J* = 7.1, 2.1,

2H), 3.56 (s, 3H), 1.25 (t, *J* = 7.0 Hz, 3H). **$^{13}C\{^1H\}$ NMR** (101 MHz, $CDCl_3$) δ 185.7, 166.6, 135.9, 132.8, 132.1, 126.1, 124.5, 121.9, 110.8, 101.9, 78.5, 63.1, 62.9, 48.0, 14.0. **IR $v_{max}$**: 3248, 2938, 1743, 1710, 1535, 1378, 1349, 1315, 1263, 1199, 1165, 1135, 1106, 1074, 1044, 1015, 962, 851, 806, 787, 743, 638, 504, 434 $cm^{-1}$. **HPLC** (Phenomenex Lux Amylose-1, 3μm, *n*-hexane:*i*-PrOH 90:10, 1 mL/min, det. UV 254 nm): Rt= 12.1 min (enantiomer *R*), 15.1 min (enantiomer *S*); *ee* 92%, $[\alpha]_D{}^{20}$ = -19.66 (c 1.0, $CHCl_3$). **HRMS** (ESI Q-TOF) m/z $[(M+H)^+]$ calc. for $C_{15}H_{17}N_2O_4$ 289.1188 found: 289.1186.

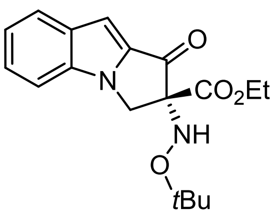


*ethyl (R)-2-(tert-butoxyamino)-1-oxo-2,3-dihydro-1H-pyrrolo[1,2-a]indole-2-carboxylate* (**2u**). The synthesis was carried out according to **GP 4**. Reaction conditions: 24 h rt, then 24 h 40°C. A scale of 0.13 mmol, orange oil, 3.00 mg (0.0091 mmol), isolated yield of 7% after flash column chromatography (*n*-hexane:EtOAc gradient 0→10% EtOAc). **$^1H$ NMR** (400 MHz, $CDCl_3$) δ 7.79 (dt, *J* = 8.3, 1.0 Hz, 1H), 7.49 (dq, *J* = 8.5, 0.9 Hz, 1H), 7.43 (ddd, *J* = 8.5, 6.8, 1.2 Hz, 1H), 7.22 (ddd, *J* = 7.9, 7.1, 1.2 Hz, 1H), 7.11 (d, *J* = 0.9 Hz, 1H), 5.06 (d, *J* = 11.2 Hz, 1H), 4.53 (d, *J* = 10.9 Hz, 1H), 4.26 (qd, *J* = 7.1, 2.5 Hz, 2H), 1.25 (t, *J* = 7.0 Hz, 3H), 1.16 (s, 9H). **$^{13}C\{^1H\}$ NMR** (101 MHz, $CDCl_3$) δ 186.1, 167.1, 135.8, 133.0, 132.1, 125.9, 124.4, 121.8, 110.8, 101.6, 78.1, 77.5, 62.7, 47.9, 26.7, 14.0. **IR $v_{max}$**: 2975, 2924, 1741, 1702, 1535, 1439, 1311, 1257, 1227, 1185, 1167, 1147, 1133, 1113, 1101, 967, 897, 802, 752, 733, 698, 638 $cm^{-1}$. **HPLC** (Phenomenex Lux Amylose-1, 3μm, *n*-hexane:*i*-PrOH 90:10, 1 mL/min, det. UV 254 nm): Rt= 6.8 min (enantiomer *R*), 8.9 min (enantiomer *S*); *ee* 94%, $[\alpha]_D{}^{20}$ = -23.87 (c 1.0, $CHCl_3$). **HRMS** (ESI Q-TOF) m/z $[(M+H)^+]$ calc. for $C_{18}H_{23}N_2O_4$ 331.1658 found: 331.1652.

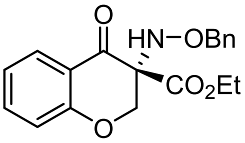


*ethyl (S)-3-((benzyloxy)amino)-4-oxochromane-3-carboxylate* (**4a**). The synthesis was carried out according to **GP 4**. Reaction conditions: 24 h 40°C. A scale of 0.059 mmol, orange oil, 19.6 mg (0.057 mmol), isolated yield of 98%. **$^1H$ NMR** (700 MHz, $CDCl_3$) δ 7.90 (dd, *J* = 8.0, 1.6 Hz, 1H), 7.54 (ddd, *J* = 8.5, 6.9, 1.6 Hz, 1H), 7.37 - 7.32 (m, 5H), 7.07 - 7.04 (m, 1H), 7.03 (d, *J* = 8.0 Hz, 1H), 6.71 (s, 1H), 4.88 (d, *J* = 11.7 Hz, 1H), 4.80 (d, *J* = 11.7 Hz, 1H), 4.76 (d, *J* = 11.7 Hz, 1H), 4.65 (d, *J* = 11.7 Hz, 1H), 4.32 - 4.24 (m, 2H), 1.27 (t, *J* = 7.2 Hz, 3H). **$^{13}C\{^1H\}$ NMR** (176 MHz, $CDCl_3$) δ 185.4, 166.9, 161.5, 137.0, 136.7, 128.7, 128.4, 128.1, 127.9, 121.7, 119.3, 118.0, 77.4, 70.5, 69.1, 62.4, 14.0. **IR $v_{max}$**: 3266, 3032, 2982, 2934, 2873, 1738, 1689, 1605, 1478, 1464, 1307, 1215, 1138, 1096, 1018, 950, 934, 913, 861, 744, 697, 601 $cm^{-1}$. **HPLC** (Phenomenex Lux Amylose-1, 3μm, *n*-hexane:*i*-PrOH 90:10, 1 mL/min, det. UV 254 nm): Rt= 12.4 min (enantiomer *S*), 14.8 min (enantiomer *R*); *ee*

>99%, $[\alpha]_D^{20}$ = -58.45 (c 1.0, $CHCl_3$). **HRMS** (ESI Q-TOF) m/z $[(M+H)^+]$ calc. for $C_{19}H_{20}NO_5$ 342.1341 found: 342.1336.

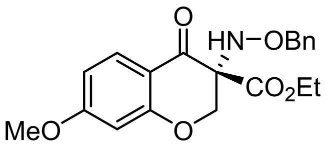

*ethyl (S)-3-((benzyloxy)amino)-7-methoxy-4-oxochromane-3-carboxylate* (**4b**). The synthesis was carried out according to **GP 4**. Reaction conditions: 24 h 40°C. A scale of 0.08 mmol, orange oil, 29.50 mg (0.079 mmol), isolated yield of 99%. **$^1$H NMR** (700 MHz, $CDCl_3$) δ 7.82 (d, *J* = 9.1 Hz, 1H), 7.37 - 7.32 (m, 5H), 6.75 (s, 1H), 6.61 (dd, *J* = 8.8, 2.4 Hz, 1H), 6.45 (d, *J* = 2.1 Hz, 1H), 4.87 (d, *J* = 12.3 Hz, 1H), 4.79 (d, *J* = 11.7 Hz, 1H), 4.74 (d, *J* = 11.7 Hz, 1H), 4.59 (d, *J* = 11.7 Hz, 1H), 4.32 - 4.23 (m, 2H), 3.86 (s, 3H), 1.27 (t, *J* = 7.2 Hz, 3H). **$^{13}$C{$^1$H} NMR** (176 MHz, $CDCl_3$) δ 183.8, 167.0, 166.7, 163.6, 137.1, 129.6, 128.7, 128.4, 128.0, 113.0, 110.7, 100.6, 77.3, 70.1, 69.4, 62.3, 55.7, 14.0. **IR $\nu_{max}$**: 2980, 2933, 2845, 1736, 1680, 1604, 1434, 1255, 1231, 1199, 1163, 1092, 1024, 992, 952, 914, 838, 746, 697, 600, 555, 470 $cm^{-1}$. **HPLC** (Phenomenex Lux Amylose-1, 3µm, *n*-hexane:*i*-PrOH 90:10, 1 mL/min, det. UV 254 nm): Rt= 21.1 min (enantiomer *R*), 23.2 min (enantiomer *S*); *ee* 98%, $[\alpha]_D^{20}$ = -62.14 (c 1.0, $CHCl_3$). **HRMS** (ESI Q-TOF) m/z $[(M+H)^+]$ calc. for $C_{20}H_{22}NO_6$ 372.1447 found: 372.1445.

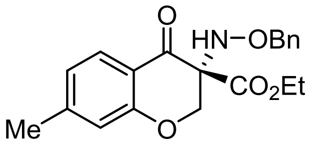

*ethyl (S)-3-((benzyloxy)amino)-7-methyl-4-oxochromane-3-carboxylate* (**4c**). The synthesis was carried out according to **GP 4**. Reaction conditions: 24 h 40°C. A scale of 0.1 mmol, orange oil, 35.20 mg (0.099 mmol), isolated yield of 99%. **$^1$H NMR** (400 MHz, $CDCl_3$) δ 7.74 (d, *J* = 7.9 Hz, 1H), 7.33 - 7.29 (m, 5H), 6.84 (ddd, *J* = 8.2, 1.5, 0.6 Hz, 1H), 6.80 (s, 1H), 6.68 (s, 1H), 4.83 (d, *J* = 11.7 Hz, 1H), 4.77 (d, *J* = 11.7 Hz, 1H), 4.71 (d, *J* = 11.7 Hz, 1H), 4.57 (d, *J* = 11.7 Hz, 1H), 4.29 - 4.19 (m, 2H), 2.36 (s, 3H), 1.24 (t, *J* = 7.2 Hz, 3H). **$^{13}$C{$^1$H} NMR** (101 MHz, $CDCl_3$) δ 185.0, 167.0, 161.5, 148.5, 137.1, 128.7, 128.4, 128.0, 127.8, 123.2, 117.9, 117.0, 70.4, 69.1, 62.3, 22.0, 14.0. **IR $\nu_{max}$**: 2990, 2917, 2875, 1739, 1680, 1620, 1456, 1295, 1269, 1225, 1199, 1162, 1105, 1026, 985, 952, 905, 863, 827, 808, 771, 744, 698, 630, 549, 528 $cm^{-1}$. **HPLC** (Phenomenex Lux Amylose-1, 3µm, *n*-hexane:*i*-PrOH 90:10, 1 mL/min, det. UV 254 nm): Rt= 13.4 min (enantiomer *R*), 13.9 min (enantiomer *S*); *ee* >99%, $[\alpha]_D^{20}$ = -64.68 (c 1.0, $CHCl_3$). **HRMS** (ESI Q-TOF) m/z $[(M+H)^+]$ calc. for $C_{20}H_{22}NO_5$ 356.1498 found: 356.1493.

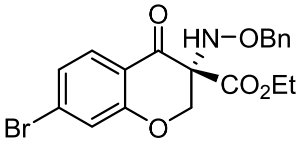

*ethyl (S)-3-((benzyloxy)amino)-7-bromo-4-oxochromane-3-carboxylate* (**4d**). The synthesis was carried out according to **GP 4**. Reaction conditions: 24 h 40°C. A scale of 0.087

mmol, orange oil, 33.00 mg (0.079 mmol), isolated yield of 90%. **$^1$H NMR** (700 MHz, $CDCl_3$) δ 7.74 (d, *J* = 8.5 Hz, 1H), 7.37 - 7.32 (m, 5H), 7.24 (d, *J* = 1.6 Hz, 1H), 7.19 (dd, *J* = 8.5, 1.6 Hz, 1H), 6.68 (s, 1H), 4.86 (d, *J* = 12.3 Hz, 1H), 4.78 (d, *J* = 11.7 Hz, 1H), 4.74 (d, *J* = 11.7 Hz, 1H), 4.64 (d, *J* = 12.3 Hz, 1H), 4.32 - 4.24 (m, 2H), 1.27 (t, *J* = 7.2 Hz, 3H). **$^{13}$C{$^1$H} NMR** (176 MHz, $CDCl_3$) δ 184.7, 166.6, 161.6, 136.9, 131.4, 129.0, 128.7, 128.4, 128.1, 125.5, 121.2, 118.2, 77.4, 70.3, 69.3, 62.5, 14.0. **IR $\nu_{max}$**: 3031, 2981, 2963, 2930, 1737, 1692, 1593, 1454, 1419, 1369, 12681201, 1097, 1063, 1023, 994, 948, 933, 856, 823, 743, 697 cm$^{-1}$. **HPLC** (Phenomenex Lux Amylose-1, 3μm, *n*-hexane:*i*-PrOH 90:10, 1 mL/min, det. UV 254 nm): Rt= 10.8 min (enantiomer *R*), 12.5 min (enantiomer *S*); *ee* >99%, $[\alpha]_D^{20}$ = -53.17 (c 1.0, $CHCl_3$). **HRMS** (ESI Q-TOF) m/z $[(M+Na)^+]$ calc. for $C_{19}H_{18}BrNO_5Na$ 442.0266 found: 442.0264.

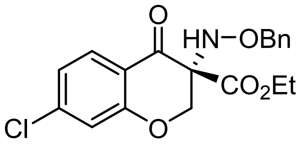


*ethyl (S)-3-((benzyloxy)amino)-7-chloro-4-oxochromane-3-carboxylate* (**4e**). The synthesis was carried out according to **GP 4**. Reaction conditions: 24 h 40°C. A scale of 0.1 mmol, orange oil, 37.00 mg (0.099 mmol), isolated yield of 99%. **$^1$H NMR** (400 MHz, $CDCl_3$) δ 7.81 (d, *J* = 8.5 Hz, 1H), 7.34 - 7.30 (m, 5H), 7.03 (d, *J* = 1.5 Hz, 1H), 7.00 (dd, *J* = 8.5, 1.9 Hz, 1H), 6.65 (s, 1H), 4.84 (d, *J* = 12.0 Hz, 1H), 4.76 (d, *J* = 11.7 Hz, 1H), 4.70 (d, *J* = 11.7 Hz, 1H), 4.62 (d, *J* = 12.0 Hz, 1H), 4.29 - 4.21 (m, 2H), 1.24 (t, *J* = 7.2 Hz, 3H). **$^{13}$C{$^1$H} NMR** (101 MHz, $CDCl_3$) δ 184.5, 166.6, 161.7, 142.8, 136.9, 129.1, 128.7, 128.4, 128.1, 122.6, 118.1, 117.9, 70.3, 69.3, 62.5, 14.0. **IR $\nu_{max}$**: 3032, 2983, 2933, 1737, 1693, 1599, 1567, 1454, 1422, 1369, 1322, 1269, 1204, 1072, 1025, 912, 880, 858, 825, 745, 697, 599, 540 cm$^{-1}$. **HPLC** (Phenomenex Lux Amylose-1, 3μm, *n*-hexane:*i*-PrOH 90:10, 1 mL/min, det. UV 254 nm): Rt= 10.3 min (enantiomer *R*), 11.5 min (enantiomer *S*); *ee* 99%, $[\alpha]_D^{20}$ = -57.43 (c 1.0, $CHCl_3$). **HRMS** (ESI Q-TOF) m/z $[(M+Na)^+]$ calc. for $C_{19}H_{18}ClNO_5Na$ 398.0772 found: 398.0770.

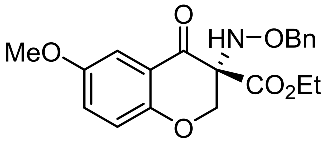


*ethyl (S)-3-((benzyloxy)amino)-6-methoxy-4-oxochromane-3-carboxylate* (**4f**). The synthesis was carried out according to **GP 4**. Reaction conditions: 24 h 40°C. A scale of 0.069 mmol, orange oil, 25.20 mg (0.068 mmol), isolated yield of 99%. **$^1$H NMR** (400 MHz, $CDCl_3$) δ 7.36 - 7.29 (m, 5H), 7.26 (s, 1H), 7.13 (dd, *J* = 8.8, 3.1 Hz, 1H), 6.94 (d, *J* = 9.1 Hz, 1H), 6.68 (s, 1H), 4.81 (d, *J* = 12.0 Hz, 1H), 4.78 (d, *J* = 11.7 Hz, 1H), 4.72 (d, *J* = 11.7 Hz, 1H), 4.58 (d, *J* = 12.0 Hz, 1H), 4.30 - 4.21 (m, 2H), 3.78 (s, 3H), 1.25 (t, *J* = 7.0 Hz, 3H). **$^{13}$C{$^1$H} NMR** (101 MHz, $CDCl_3$) δ 185.5, 166.9, 156.3, 154.3, 137.0, 128.7, 128.4, 128.1, 126.2, 119.3, 119.1, 107.8, 70.4, 69.3, 62.4, 55.8, 14.0. **IR $\nu_{max}$**: 2981, 2936, 2872, 1738, 1686, 1490, 1428, 1367, 1281, 1234, 1198, 1134, 1104, 1022, 975, 909, 867, 827, 741, 697, 625 cm$^{-1}$. **HPLC**: Despite the use of various chiral phases, a satisfactory separation

of enantiomers by HPLC was not achieved. **HRMS** (ESI Q-TOF) m/z [(M+H)$^+$] calc. for $C_{20}H_{21}NO_6$ 372.1447 found: 372.1445.

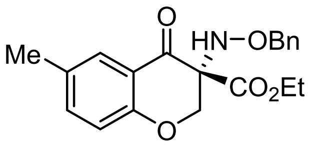


*ethyl (S)-3-((benzyloxy)amino)-6-methyl-4-oxochromane-3-carboxylate* (**4g**). The synthesis was carried out according to **GP 4**. Reaction conditions: 24 h 40°C. A scale of 0.1 mmol, orange oil, 35.14 mg (0.099 mmol), isolated yield of 99%. **$^1$H NMR** (400 MHz, $CDCl_3$) δ 7.65 (d, *J* = 1.5 Hz, 1H), 7.33 - 7.31 (m, 5H), 6.91 (d, *J* = 8.5 Hz, 1H), 6.68 (s, 1H), 4.83 (d, *J* = 11.7 Hz, 1H), 4.78 (d, *J* = 11.7 Hz, 1H), 4.72 (d, *J* = 11.7 Hz, 1H), 4.59 (d, *J* = 12.0 Hz, 1H), 4.30 - 4.20 (m, 2H), 2.30 (s, 3H), 1.24 (t, *J* = 7.2 Hz, 3H). **$^{13}$C{$^1$H} NMR** (101 MHz, $CDCl_3$) δ 185.6, 166.9, 159.6, 137.9, 137.0, 131.2, 128.7, 128.4, 128.0, 127.3, 118.9, 117.8, 77.4, 70.5, 69.1, 62.3, 20.4, 14.0. **IR $\nu_{max}$**: 2991, 2915, 2874, 1738, 1680, 1620, 1494, 1413, 1298, 1250, 1215, 1139, 1105, 1083, 1056, 1043, 1025, 985, 908, 870, 827, 744, 698, 622, 548, 520, 479 cm$^{-1}$. **HPLC** (Phenomenex Lux Amylose-1, 3µm, *n*-hexane:*i*-PrOH 90:10, 1 mL/min, det. UV 254 nm): Rt= 11.7 min (enantiomer *S*), 12.4 min (enantiomer *R*); *ee* >99%, $[\alpha]_D^{20}$ = -60.73 (c 1.0, $CHCl_3$). **HRMS** (ESI Q-TOF) m/z [(M+H)$^+$] calc. for $C_{20}H_{22}NO_5$ 356.1498 found: 356.1494.

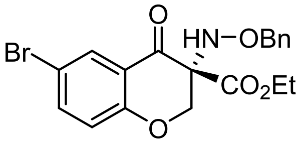


*ethyl (S)-3-((benzyloxy)amino)-6-bromo-4-oxochromane-3-carboxylate* (**4h**). The synthesis was carried out according to **GP 4**. Reaction conditions: 24 h 40°C. A scale of 0.055 mmol, orange oil, 22.60 mg (0.054 mmol), isolated yield of 98%. **$^1$H NMR** (700 MHz, $CDCl_3$) δ 7.99 (d, *J* = 2.1 Hz, 1H), 7.61 (dd, *J* = 8.5, 2.7 Hz, 1H), 7.37 - 7.32 (m, 5H), 6.94 (d, *J* = 9.1 Hz, 1H), 6.66 (s, 1H), 4.85 (d, *J* = 11.7 Hz, 1H), 4.78 (d, *J* = 11.7 Hz, 1H), 4.73 (d, *J* = 11.7 Hz, 1H), 4.64 (d, *J* = 11.7 Hz, 1H), 4.32 - 4.23 (m, 2H), 1.27 (t, *J* = 7.2 Hz, 3H). **$^{13}$C{$^1$H} NMR** (176 MHz, $CDCl_3$) δ 184.4, 166.5, 160.3, 139.3, 136.9, 130.2, 128.7, 128.4, 128.1, 120.6, 120.0, 114.4, 77.4, 70.2, 69.2, 62.6, 14.0. **IR $\nu_{max}$**: 3031, 2982, 2929, 1738, 1694, 1599, 1472, 1415 , 1272, 1230, 1202, 1141, 1097, 1021, 952, 860, 823, 745, 697, 610, 595 cm$^{-1}$. **HPLC** (Phenomenex Lux Amylose-1, 3µm, *n*-hexane:*i*-PrOH 90:10, 1 mL/min, det. UV 254 nm): Rt= 11.0 min (enantiomer *S*), 11.8 min (enantiomer *R*); *ee* >99%, $[\alpha]_D^{20}$ = -63.89 (c 1.0, $CHCl_3$). **HRMS** (ESI Q-TOF) m/z [(M+Na)$^+$] calc. for $C_{19}H_{18}BrNO_5Na$ 442.0266 found: 442.0266.

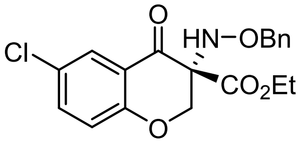


*ethyl (S)-3-((benzyloxy)amino)-6-chloro-4-oxochromane-3-carboxylate* (**4i**). The synthesis was carried out according to **GP 4**. Reaction conditions: 24 h 40°C. A scale of 0.082

mmol, orange oil, 30.15 mg (0.080 mmol), isolated yield of 98%. **$^1$H NMR** (700 MHz, $CDCl_3$) δ 7.84 (d, *J* = 2.7 Hz, 1H), 7.47 (dd, *J* = 9.1, 2.7 Hz, 1H), 7.37 - 7.33 (m, 5H), 7.00 (d, *J* = 8.5 Hz, 1H), 6.67 (s, 1H), 4.85 (d, *J* = 12.3 Hz, 1H), 4.79 (d, *J* = 11.7 Hz, 1H), 4.74 (d, *J* = 11.7 Hz, 1H), 4.64 (d, *J* = 12.3 Hz, 1H), 4.31 - 4.25 (m, 2H), 1.27 (t, *J* = 7.21 Hz, 3H). **$^{13}$C{$^1$H} NMR** (176 MHz, $CDCl_3$) δ 184.5, 166.5, 159.9, 136.9, 136.5, 128.7, 128.4, 128.1, 127.3, 127.0, 120.1, 119.7, 77.4, 70.2, 69.3, 62.6, 14.0. **IR $v_{max}$**: 3031, 2982, 2931, 2874, 1738, 1694, 1605, 1475, 1419, 1273, 1227, 1202, 1141, 1101, 1082, 1022, 993, 957, 900, 825, 746, 698, 644, 617, 528 cm-1. **HPLC** (Phenomenex Lux Amylose-1, 3µm, *n*-hexane:*i*-PrOH 90:10, 1 mL/min, det. UV 254 nm): Rt= 10.5 min (enantiomer *S*), 11.9 min (enantiomer *R*); *ee* >99%, $[\alpha]_D$ $^{20}$ = -65.32 (c 1.0, $CHCl_3$). **HRMS** (ESI Q-TOF) m/z [(M+Na)$^+$] calc. for $C_{19}H_{18}ClNO_5Na$ 398.0772 found: 398.0769.

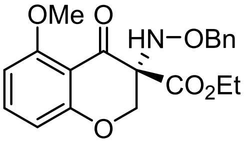


*ethyl (S)-3-((benzyloxy)amino)-5-methoxy-4-oxochromane-3-carboxylate* (**4j**). The synthesis was carried out according to **GP 4**. Reaction conditions: 24 h 40°C. A scale of 0.071 mmol, orange oil, 25.00 mg (0.067 mmol), isolated yield of 94%. **$^1$H NMR** (700 MHz, $CDCl_3$) δ 7.43 (t, *J* = 8.5 Hz, 1H), 7.35 - 7.31 (m, 5H), 6.72 (s, 1H), 6.62 - 6.61 (m, 1H), 6.52 (d, *J* = 7.5 Hz, 1H), 4.83 (d, *J* = 11.7 Hz, 1H), 4.79 (d, *J* = 11.7 Hz, 1H), 4.75 (d, *J* = 11.7 Hz, 1H), 4.61 (d, *J* = 12.3 Hz, 1H), 4.31 - 4.22 (m, 2H), 3.91 (s, 3H), 1.27 (t, *J* = 7.2 Hz, 3H). **$^{13}$C{$^1$H} NMR** (176 MHz, $CDCl_3$) δ 183.7, 167.1, 163.0, 161.5, 137.1, 136.8, 128.7, 128.3, 128.0, 109.9, 109.7, 103.9, 77.3, 70.9, 68.2, 62.2, 56.2, 14.0. **IR $v_{max}$**: 2980, 2935, 2843, 1736, 1684, 1601, 1474, 1435, 1331, 1284, 1252, 1129, 1073, 1024, 992, 955, 905, 859, 798, 741, 697, 605, 517 cm$^{-1}$. **HPLC** (Phenomenex Lux Amylose-1, 3µm, *n*-hexane:*i*-PrOH 80:20, 1 mL/min, det. UV 254 nm): Rt= 17.0 min (enantiomer *R*), 20.5 min (enantiomer *S*); *ee* 98%, $[\alpha]_D$ $^{20}$ = -61.68 (c 1.0, $CHCl_3$). **HRMS** (ESI Q-TOF) m/z [(M+H)$^+$] calc. for $C_{20}H_{22}NO_6$ 372.1447 found: 372.1442.

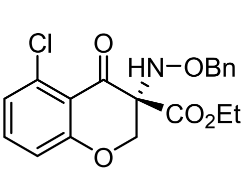


*ethyl (S)-3-((benzyloxy)amino)-5-chloro-4-oxochromane-3-carboxylate* (**4k**). The synthesis was carried out according to **GP 4**. Reaction conditions: 24 h 40°C. A scale of 0.075 mmol, orange oil, 27.5 mg (0.073 mmol), isolated yield of 98%. **$^1$H NMR** (400 MHz, $CDCl_3$) 7.38 - 7.30 (m, 6H), 7.05 (dd, *J* = 7.9, 1.2 Hz, 1H), 6.93 (dd, *J* = 8.5, 1.2 Hz, 1H), 6.66 (s, 1H), 4.83 (d, *J* = 12.0 Hz, 1H), 4.77 (d, *J* = 11.7 Hz, 1H), 4.72 (d, *J* = 11.7 Hz, 1H), 4.62 (d, *J* = 12.0 Hz, 1H), 4.30 - 4.22 (m, 2H), 1.25 (t, *J* = 7.2 Hz, 3H). **$^{13}$C{$^1$H} NMR** (101 MHz, $CDCl_3$) δ 183.8, 166.7, 162.8, 136.9, 135.4, 135.1, 128.7, 128.4, 128.1, 124.9, 116.9, 116.7, 70.7, 68.4, 62.4, 14.0. **IR $v_{max}$**: 3262, 2982, 2928, 1738, 1695, 1594, 1513, 1464, 1445, 1367, 1314, 1260, 1209, 1102, 1072, 1025, 992, 949, 915, 881, 795, 740, 697, 631, 606, 539, 463 cm$^{-1}$. **HPLC** (Phenomenex Lux Amylose-1, 3µm, *n*-hexane:*i*-PrOH 90:10, 1 mL/min, det. UV 254 nm): Rt= 13.5 min (enantiomer *S*), 18.1 min (enantiomer *R*); *ee* 98%, $[\alpha]_D$ $^{20}$ =

-56.48 (c 1.0, $CHCl_3$). **HRMS** (ESI Q-TOF) m/z [$(M+H)^+$] calc. for $C_{19}H_{19}ClNO_5$ 376.0952 found: 376.0949.

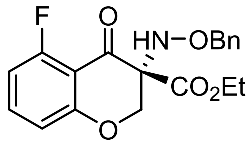

*ethyl (S)-3-((benzyloxy)amino)-5-fluoro-4-oxochromane-3-carboxylate* (**4l**). The synthesis was carried out according to **GP 4**. Reaction conditions: 24 h 40°C. A scale of 0.088 mmol, orange oil, 30.0 mg (0.083 mmol), isolated yield of 95%. **$^1$H NMR** (700 MHz, $CDCl_3$) 7.47 (td, $J$ = 8.5, 5.9 Hz, 1H), 7.37 - 7.32 (m, 5H), 6.84 (d, $J$ = 8.5 Hz, 1H), 6.74 - 6.72 (m, 1H), 6.70 (s, 1H), 4.85 (d, $J$ = 12.3 Hz, 1H), 4.79 (d, $J$ = 11.7 Hz, 1H), 4.75 (d, $J$ = 11.7 Hz, 1H), 4.65 (d, $J$ = 11.7 Hz, 1H), 4.33 - 4.24 (m, 2H), 1.28 (t, $J$ = 7.21 Hz, 3H). **$^{13}$C{$^1$H} NMR** (176 MHz, $CDCl_3$) δ 183.0, 166.6, 162.20 (d, $J$ = 267.2 Hz), 162.16 (d, $J$ = 2.7 Hz), 136.9, 136.7 (d, $J$ = 11.7 Hz), 128.7, 128.4, 128.1, 113.6 (d, $J$ = 3.9 Hz), 109.7 (d, $J$ = 9.4 Hz), 109.1 (d, $J$ = 20.8 Hz), 77.4, 70.7, 68.8, 62.5, 14.0. **IR $v_{max}$**: 3269, 3031, 2984, 2932, 2874, 1738, 1696, 1619, 1473, 1325, 1250, 1219, 1102, 1070, 995, 913, 860, 801,740, 698 $cm^{-1}$. **HPLC** (Phenomenex Lux Amylose-1, 3μm, *n*-hexan:*i*-PrOH 90:10, 1 mL/min, det. UV 254 nm): Rt= 13.0 min (enantiomer *S*), 17.2 min (enantiomer *R*); *ee* 99%, $[\alpha]_D{}^{20}$ = -49.88 (c 1.0, $CHCl_3$). **HRMS** (ESI Q-TOF) m/z [$(M+H)^+$] calc. for $C_{19}H_{19}FNO_5$ 360.1247 found: 360.1243.

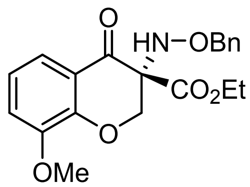

*ethyl (S)-3-((benzyloxy)amino)-8-methoxy-4-oxochromane-3-carboxylate* (**4m**). The synthesis was carried out according to **GP 4**. Reaction conditions: 24 h 40°C. A scale of 0.1 mmol, orange oil, 36.50 mg (0.098 mmol), isolated yield of 98%. **$^1$H NMR** (400 MHz, $CDCl_3$) δ 7.47 (dd, $J$ = 7.9, 1.5 Hz, 1H), 7.32 - 7.30 (m, 5H), 7.08 (dd, $J$ = 7.9, 1.5 Hz, 1H), 6.97 (t, $J$ = 7.9 Hz, 1H), 6.66 (s, 1H), 4.92 (d, $J$ = 12.0 Hz, 1H), 4.79 (d, $J$ = 11.7 Hz, 1H), 4.73 (d, $J$ = 11.7 Hz, 1H), 4.71 (d, $J$ = 12.0 Hz, 1H), 4.30 - 4.21 (m, 2H), 3.91 (s, 3H), 1.24 (t, $J$ = 7.2 Hz, 3H). **$^{13}$C{$^1$H} NMR** (101 MHz, $CDCl_3$) δ 185.5, 166.8, 151.6, 148.8, 137.0, 128.6, 128.3, 128.0, 121.3, 120.0, 118.9, 117.4, 77.4, 70.4, 69.6, 62.4, 56.3, 14.0. **IR $v_{max}$**: 2918, 2849, 1737, 1688, 1605, 1584, 1490, 1440, 1367, 1260, 1213, 1189, 1175, 1103, 1069, 1024, 992, 950, 805, 735, 698 $cm^{-1}$. **HPLC** (Phenomenex Lux Cellulose-1, 3μm, *n*-hexane:*i*-PrOH 95:5, 0.8 mL/min, det. UV 254 nm): Rt= 20.5 min (enantiomer *S*), 21.8 min (enantiomer *R*); *ee* 99%, $[\alpha]_D{}^{20}$ = -55.29 (c 1.0, $CHCl_3$). **HRMS** (ESI Q-TOF) m/z [$(M+H)^+$] calc. for $C_{20}H_{22}NO_6$ 372.1447 found: 372.1446.

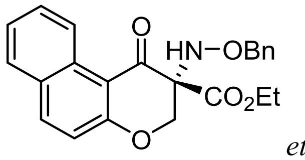


*ethyl (S)-2-((benzyloxy)amino)-1-oxo-2,3-dihydro-1H-benzo[f]chromene-2-carboxylate* (**4n**). The synthesis was carried out according to **GP 4**. Reaction conditions: 24 h 40°C. A scale of 0.1 mmol, orange oil, 24.00 mg (0.061 mmol), isolated yield of 61% after flash column chromatography (*n*-hexane:EtOAc gradient 0→10% EtOAc). **$^1$H NMR** (400 MHz, $CDCl_3$) δ 9.31 (dd, *J* = 8.8, 1.0 Hz, 1H), 7.95 (d, *J* = 8.8 Hz, 1H), 7.77 - 7.74 (m, 1H), 7.63 (ddd, *J* = 8.5, 7.0, 1.5 Hz, 1H), 7.44 (ddd, *J* = 8.2, 6.8, 1.2 Hz, 1H), 7.35 - 7.29 (m, 5H), 7.12 (d, *J* = 8.8 Hz, 1H), 6.82 (s, 1H), 4.98 (d, *J* = 11.7 Hz, 1H), 4.80 (d, *J* = 11.7 Hz, 1H), 4.75 (d, *J* = 11.7 Hz, 1H), 4.71 (d, *J* = 12.0 Hz, 1H), 4.34 - 4.20 (m, 2H), 1.25 (t, *J* = 7.0 Hz, 3H). **$^{13}$C{$^1$H} NMR** (101 MHz, $CDCl_3$) δ 185.9, 167.2, 163.8, 138.4, 137.1, 131.6, 130.0, 129.2, 128.7, 128.6, 128.4, 128.1, 125.6, 125.2, 118.6, 110.8, 70.6, 68.8, 62.3, 14.0. **IR $\nu_{max}$**: 3031, 2982, 2929, 1737, 1667, 1618, 1597, 1568, 1511, 1470, 1435, 1366, 1238, 1206, 1158, 1135, 1108, 1064, 1027, 987, 892, 860, 825, 741, 697, 613 $cm^{-1}$. **HPLC** (Phenomenex Lux Amylose-1, 3µm, *n*-hexane:*i*-PrOH 90:10, 1 mL/min, det. UV 254 nm): Rt= 16.3 min (enantiomer *S*), 18.3 min (enantiomer *R*); *ee* 98%, $[\alpha]_D{}^{20}$ = -44.67 (c 1.0, $CHCl_3$). **HRMS** (ESI Q-TOF) m/z [$(M+H)^+$] calc. for $C_{23}H_{22}NO_5$ 392.1498 found: 392.1493.

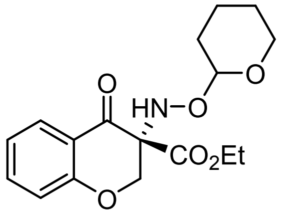


*ethyl (3S)-4-oxo-3-(((tetrahydro-2H-pyran-2-yl)oxy)amino)chromane-3-carboxylate* (**4o**). The synthesis was carried out according to **GP 4**. Reaction conditions: 24 h 40°C. A scale of 0.2 mmol, orange oil, 66.50 mg (0.198 mmol), isolated yield of 99% for both diastereoisomers. *dr* 1:1. Analytical data for the mixture of diastereoisomers: **$^1$H NMR** (400 MHz, $CDCl_3$) δ 7.90 (dd, *J* = 7.9, 1.6 Hz, 1H), 7.86 (dd, *J* = 7.9, 1.8 Hz, 1H), 7.53 - 7.48 (m, 2H), 7.06 - 6.99 (m, 4H), 6.98 (s, 1H), 6.78 (s, 1H), 5.02 (d, *J* = 11.7 Hz, 1H), 4.92 (d, *J* = 12.0 Hz, 1H), 4.90 - 4.88 (m, 1H), 4.83 - 4.81 (m, 1H), 4.66 (d, *J* = 12.0 Hz, 1H), 4.63 (d, *J* = 11.7 Hz, 1H), 4.32 - 4.22 (m, 4H), 3.97 - 3.87 (m, 2H), 3.61 - 3.56 (m, 2H), 1.72 - 1.46 (m, 12H), 1.29 (t, *J* = 7.0 Hz, 3H), 1.24 (t, *J* = 7.2 Hz, 3H). **$^{13}$C{$^1$H} NMR** (101 MHz, $CDCl_3$) δ 186.2, 184.9, 166.9, 166.2, 161.6, 161.4, 136.7, 136.6, 127.9, 127.7, 121.7, 119.6, 119.1, 118.1, 118.0, 101.9, 101.6, 70.3, 70.0, 69.7, 69.0, 62.9, 62.39, 62.37, 62.28, 28.8, 28.6, 25.20, 25.15, 19.66, 19.51, 14.1, 14.0. **IR $\nu_{max}$**: 2942, 2869, 1737, 1691, 1605, 1478, 1307, 1204, 1137, 1106, 1074, 1038, 1018, 967, 906, 870, 840, 758, 732, 522 $cm^{-1}$. **HPLC** diastereoisomer 1: (Phenomenex Lux Amylose-1, 3µm, *n*-hexane:*i*-PrOH 90:10, 1 mL/min, det. 254 nm): Rt= 14.5 min, 18.0 min; *ee* >99%. diastereoisomer 2: (Phenomenex Lux Amylose-1, 3µm, *n*-hexane:*i*-PrOH 90:10, 1 mL/min, det. UV 254 nm): Rt= 15.7 min, 25.9 min; *ee* >99%, $[\alpha]_D{}^{20}$ = -21.25 (c 1.0, $CHCl_3$). **HRMS** (ESI Q-TOF) m/z [$(M+H)^+$] calc. for $C_{17}H_{22}NO_6$ 336.1447 found: 336.1444.

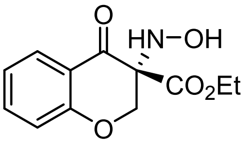


*ethyl (S)-3-(hydroxyamino)-4-oxochromane-3-carboxylate* (**4p**). The synthesis was carried out according to **GP 5**. A scale of 0.6 mmol, white oil, 0.137 g (0.55 mmol), isolated yield of 91%. **$^1$H NMR** (400 MHz, $CDCl_3$) δ 7.90 (dd, *J* = 7.9, 1.5 Hz, 1H), 7.53 (ddd, *J* = 8.5, 7.3, 1.8 Hz, 1H), 7.06 (ddd, *J* = 7.9, 7.0, 0.9 Hz, 1H), 7.01 (dd, *J* = 8.5, 0.8 Hz, 1H), 4.88 (d, *J* = 11.7 Hz, 1H), 4.71 (d, *J* = 12.0 Hz, 1H), 4.33 - 4.25 (m, 2H), 1.26 (t, *J* = 7.0 Hz, 3H). **$^{13}$C{$^1$H} NMR** (101 MHz, $CDCl_3$) δ 186.1, 167.4, 161.3, 136.8, 127.9, 122.0, 119.4, 118.0, 70.9, 69.1, 62.5, 14.0. **IR $v_{max}$**: 3451, 3276, 2983, 2938, 1736, 1688, 1604, 1464, 1327, 1307, 1216, 1138, 1097, 1017, 953, 928, 857, 757, 621, 522 $cm^{-1}$. **HPLC** (Phenomenex Lux Amylose-1, 3µm, *n*-hexane:i-PrOH 70:30, 1.2 mL/min, det. 254 nm): Rt= 6.7 min (enantiomer *R*), 13.7 min (enantiomer *S*); *ee* >99 %, $[\alpha]_D^{20}$ = -66.38 (c 1.0, $CHCl_3$). **HRMS** (ESI Q-TOF) m/z [$(M+Na)^+$] calc. for $C_{12}H_{13}NO_5Na$ 274.0692 found: 274.0690.

## 4. X-Ray Crystallography data

*Sample preparation:* A single crystal of **2d** was obtained by slow evaporation from EtOAc/hex solution.

**Structure determination**

Single-crystal X-ray diffraction data for **2d** were collected on an XtaLAB Synergy-S Dualflex diffractometer equipped with monochromated CuKα radiation (λ = 1.54184 Å). The crystal was coated with Paratone-N oil and mounted on a loop. Data collection was carried out at 100(2) K to minimize solvent loss, possible structural disorder and thermal motion effects. Data frames were processed (unit cell determination, intensity data integration, correction for Lorentz and polarisation effects, and empirical absorption correction) by using the corresponding diffractometer's software package.[11] The structure was solved by using direct method with SHELXS-2019/3 [12] and refined by using full-matrix least-squares method based on $F^2$ by using SHELXL-2019/3. [13] The programs Mercury [14] and POV-Ray [15] were both used to prepare molecular graphics. All non-hydrogen atoms were refined anisotropically. All C-H hydrogen atoms were positioned geometrically with C-H = 0.95 Å (aromatic), 0.98 (methyl), 0.99 Å (methylene) and refined as riding, with Uiso(H) = 1.2 Ueq (C) or 1.5 Ueq (C) for methyl groups. Atom H13 of the N-H group was located from a diference Fourier map and refined freely. The ethyl moiety from the ester group was found to be disordered and modelled into two positions with occupancies of 81 and 19%, respectively.

A summary of the data collection and structure refinement parameters is provided in **Table 1**.

**Table 1** Crystal data and details of the refinement parameters for **2d**

| Compound reference | **2d** |
|---|---|
| Chemical formula | $C_{21}H_{19}BrN_2O_4$ |
| Formula Mass | 443.29 |
| Crystal system | Monoclinic |
| *a*/Å | 8.60390(10) |
| *b*/Å | 10.67560(10) |
| *c*/Å | 10.84340(10) |
| *α*/° | 90 |

| | |
|---|---|
| $\beta$/° | 106.7690(10) |
| $\gamma$/° | 90 |
| Unit cell volume/Å$^3$ | 953.632(17) |
| Temperature/K | 100(2) |
| Space group | $P$21 |
| No. of formula units per unit cell, $Z$ | 2 |
| Radiation type | CuKα |
| Absorption coefficient, $\mu$/mm$^{-1}$ | 3.201 |
| No. of reflections measured | 11973 |
| No. of independent reflections | 3626 |
| $R_{int}$ | 0.0148 |
| Final $R_1{}^a$ values ($I > 2\sigma(I)$) | 0.0175 |
| Final $wR_2{}^b(F^2)$ values ($I > 2\sigma(I)$) | 0.0450 |
| Final $R_1{}^a$ values (all data) | 0.0175 |
| Final $wR_2{}^b(F^2)$ values (all data) | 0.0450 |
| Goodness of fit on $F^2$ | 1.080 |
| Flack parameter | -0.007(6) |

$^aR_1 = \sum\|F_o| - |F_c\|/\sum|F_o|$.

$^b wR_2 = \{\sum[w(F_o{}^2 - F_c{}^2)^2]/\sum[w(F_o{}^2)^2]\}^{1/2}$.

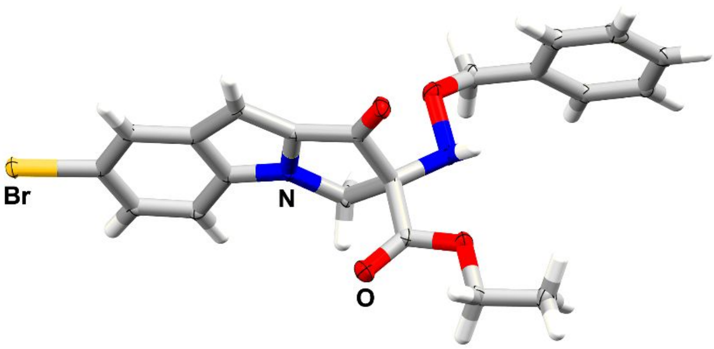


**Figure S1**. Molecular structure of **2d**, with the atomic displacement plot shown at 50% probability; disorder on the ester side chain omitted for clarity.

## 5. NMR Spectra

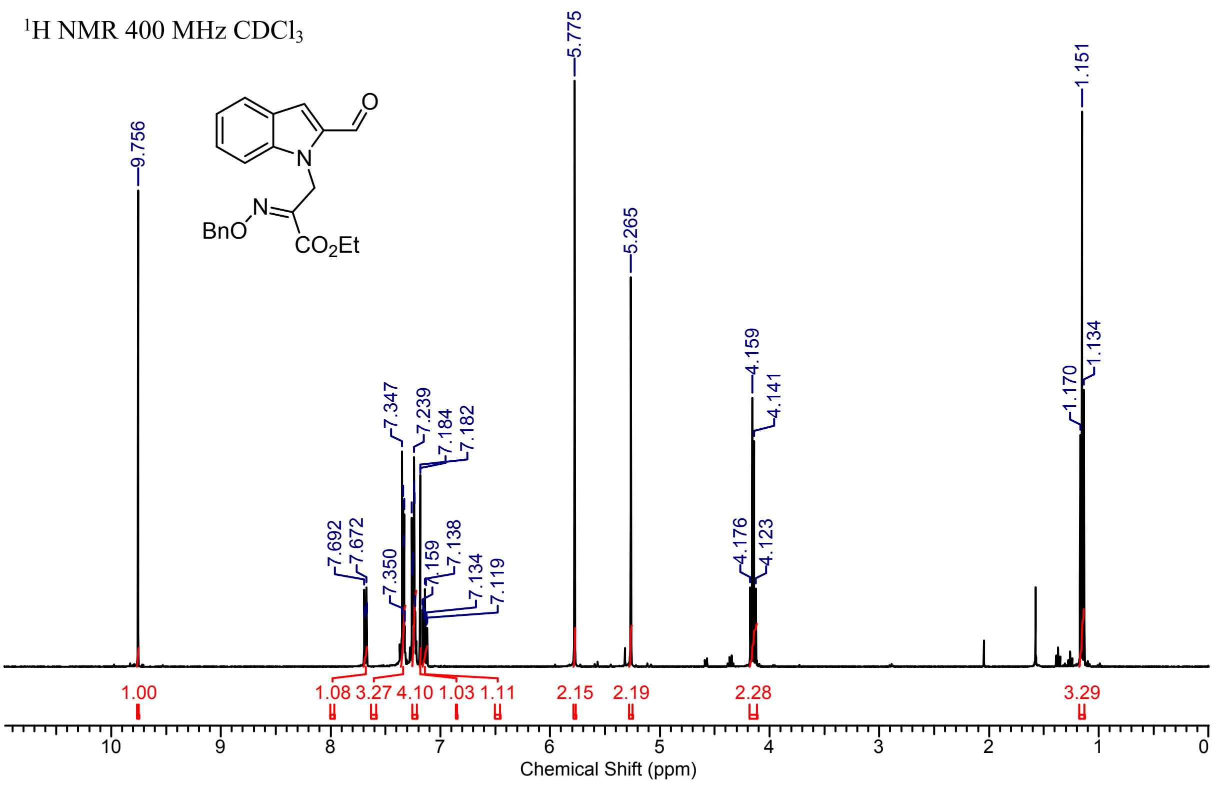


**Figure S2**. $^1$H spectrum of compound **1a**.

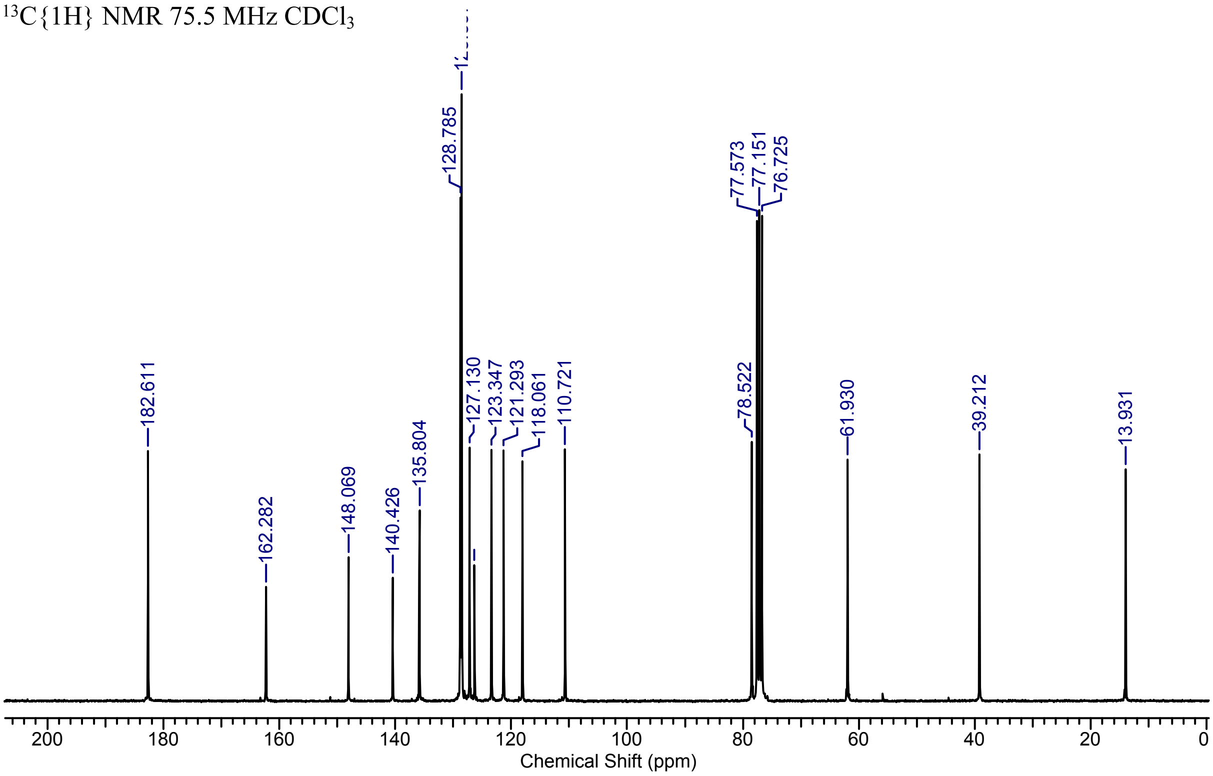


**Figure S3**. $^{13}$C spectrum of compound **1a**

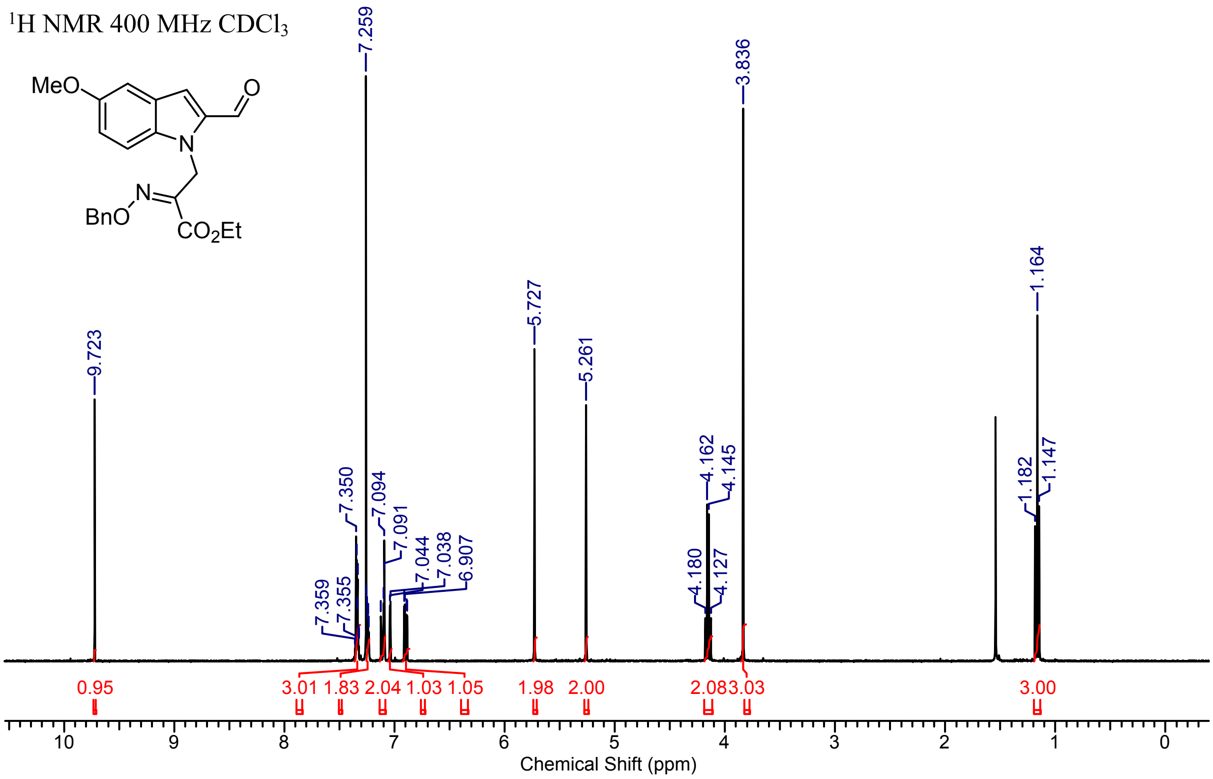


**Figure S4**. $^{1}$H spectrum of compound **1b**.

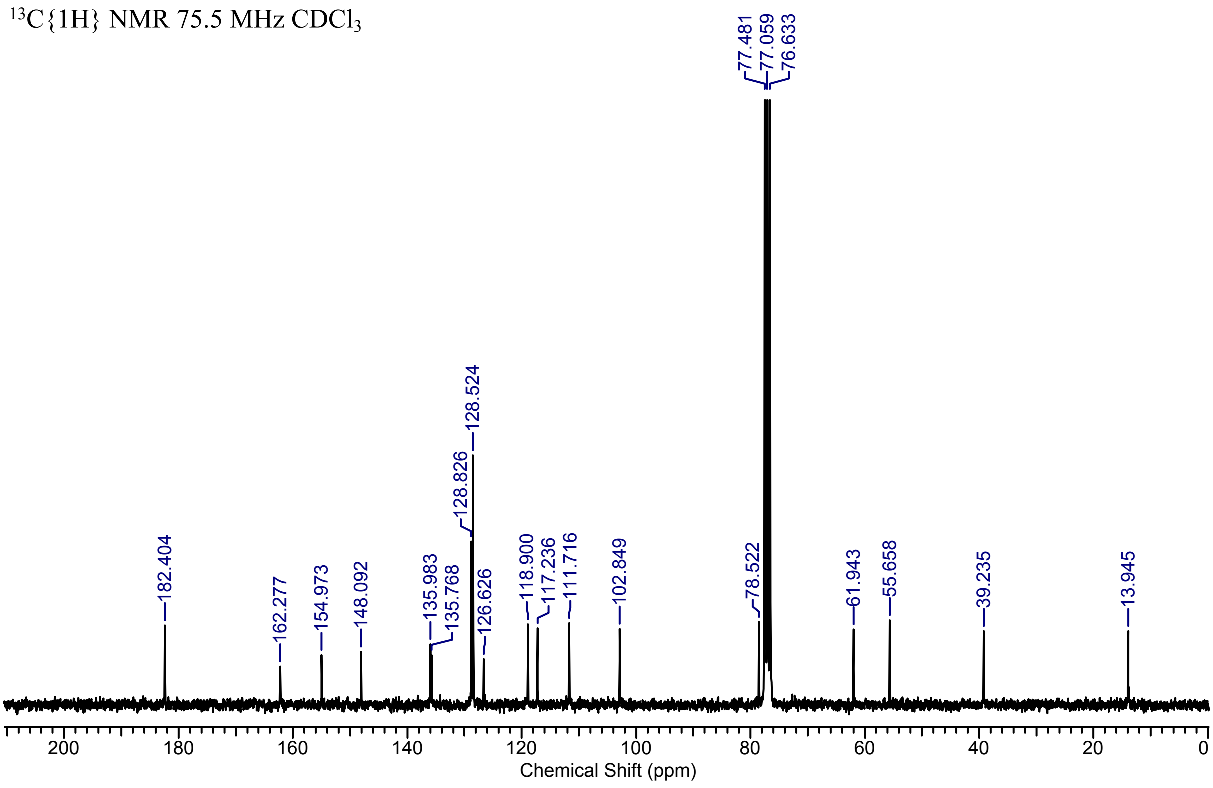


**Figure S5**. $^{13}$C spectrum of compound **1b**.

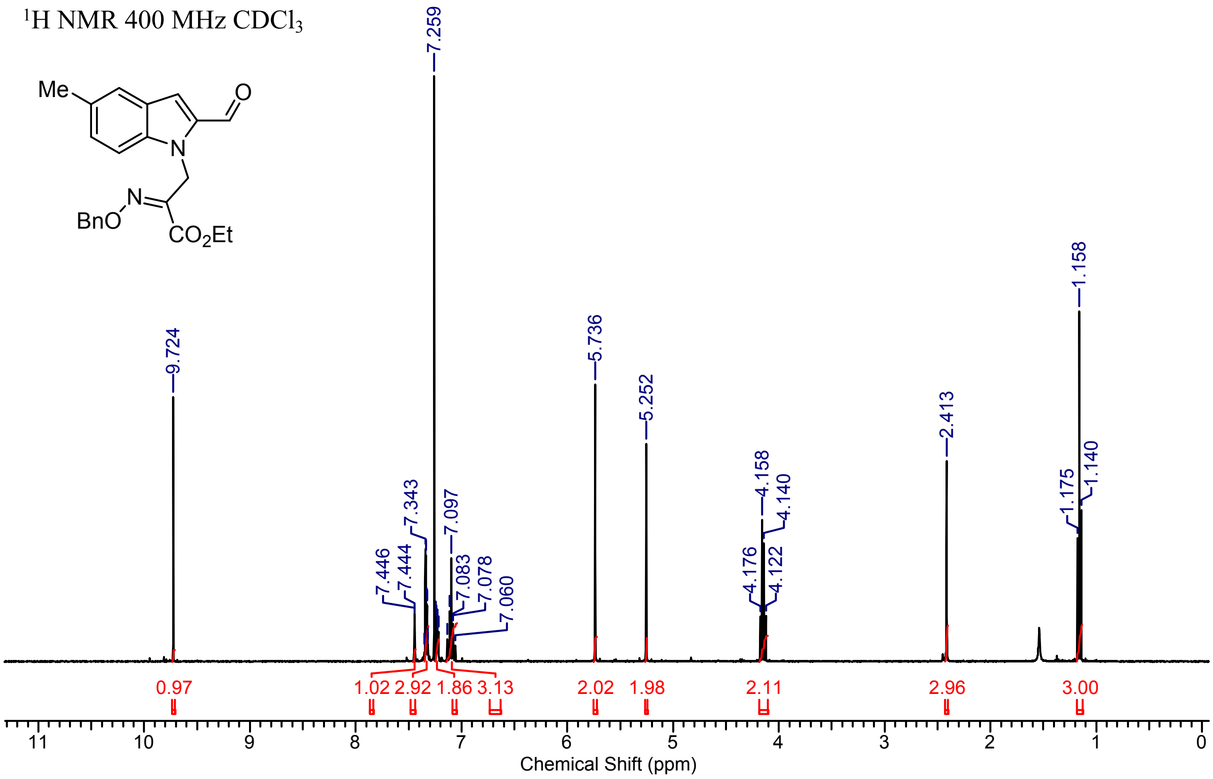


**Figure S6**. $^{1}H$ spectrum of compound **1c**.

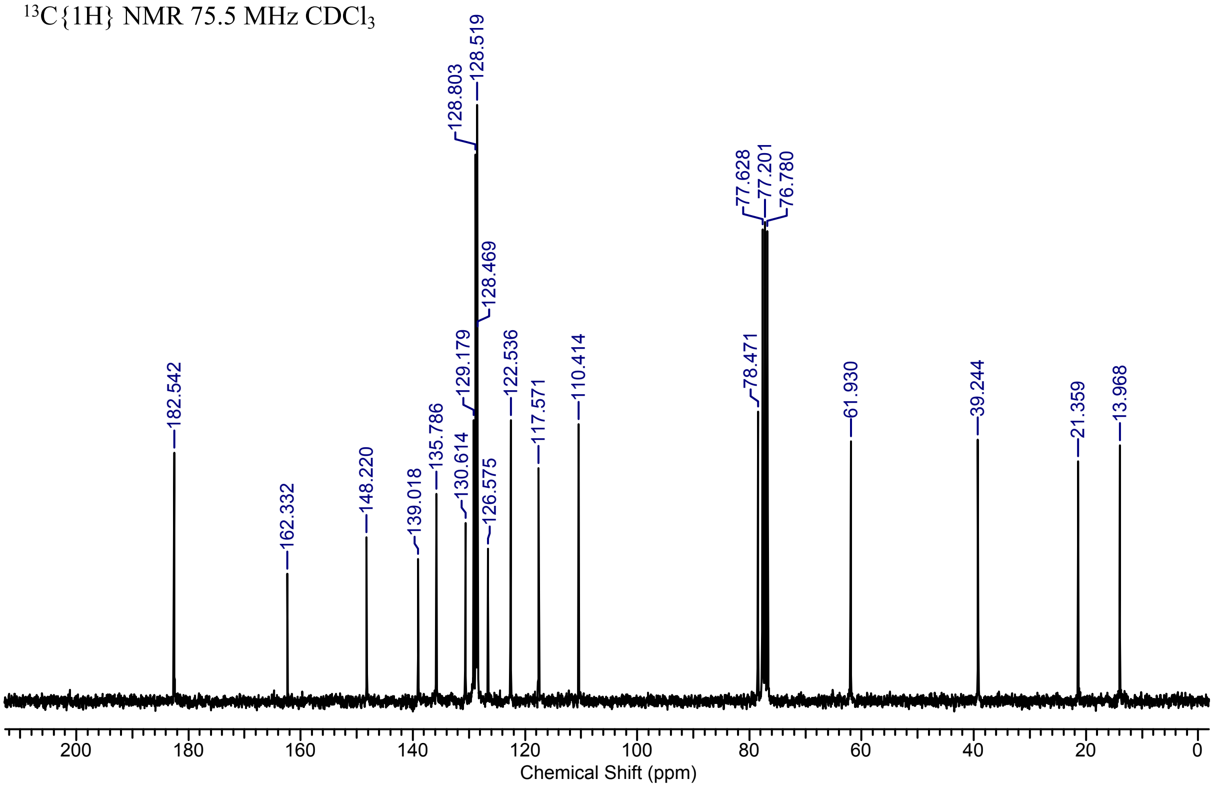


**Figure S7**. $^{13}C$ spectrum of compound **1c**.

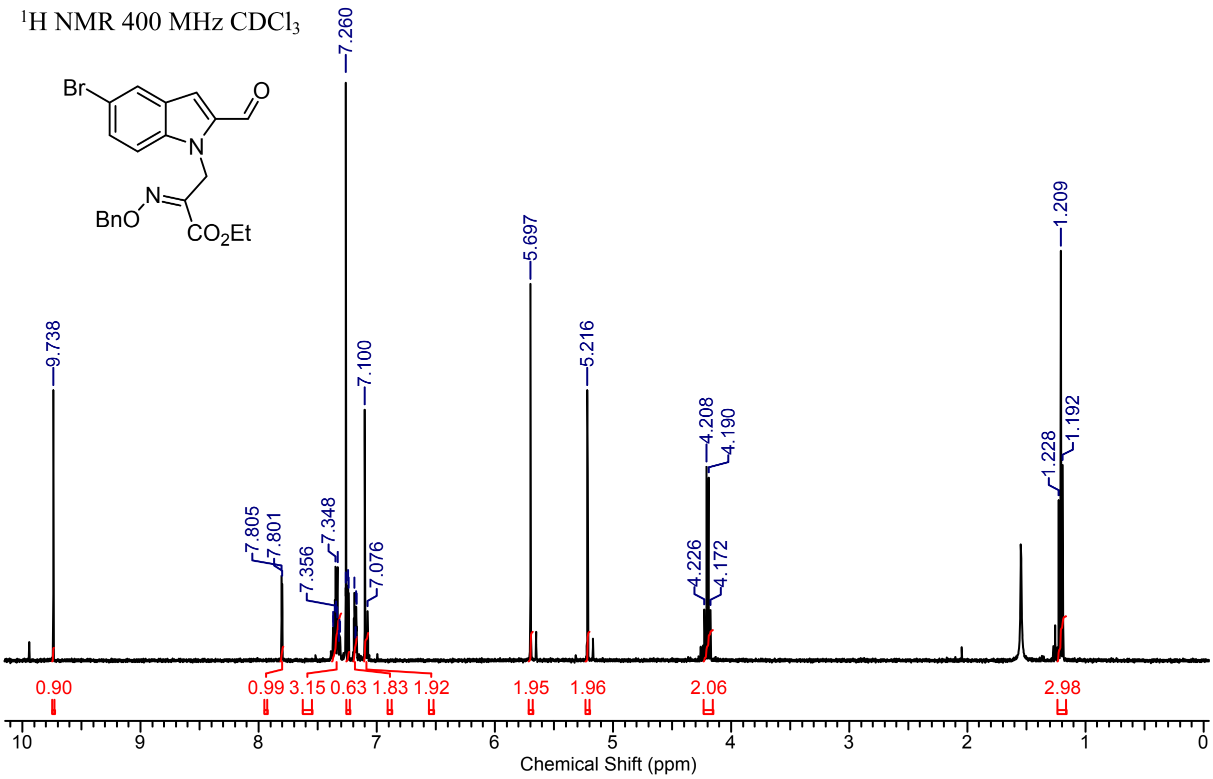


**Figure S8**. $^1$H spectrum of compound **1d**.

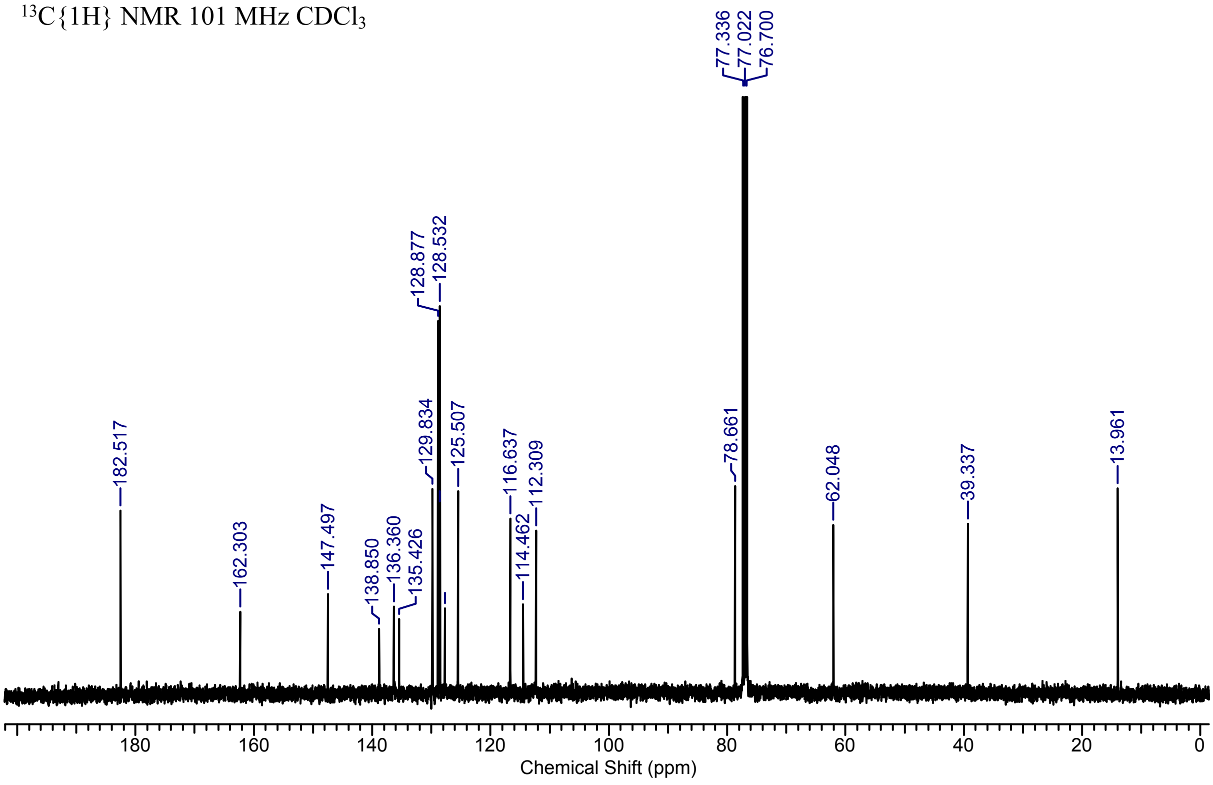


**Figure S9**. $^{13}$C spectrum of compound **1d**.

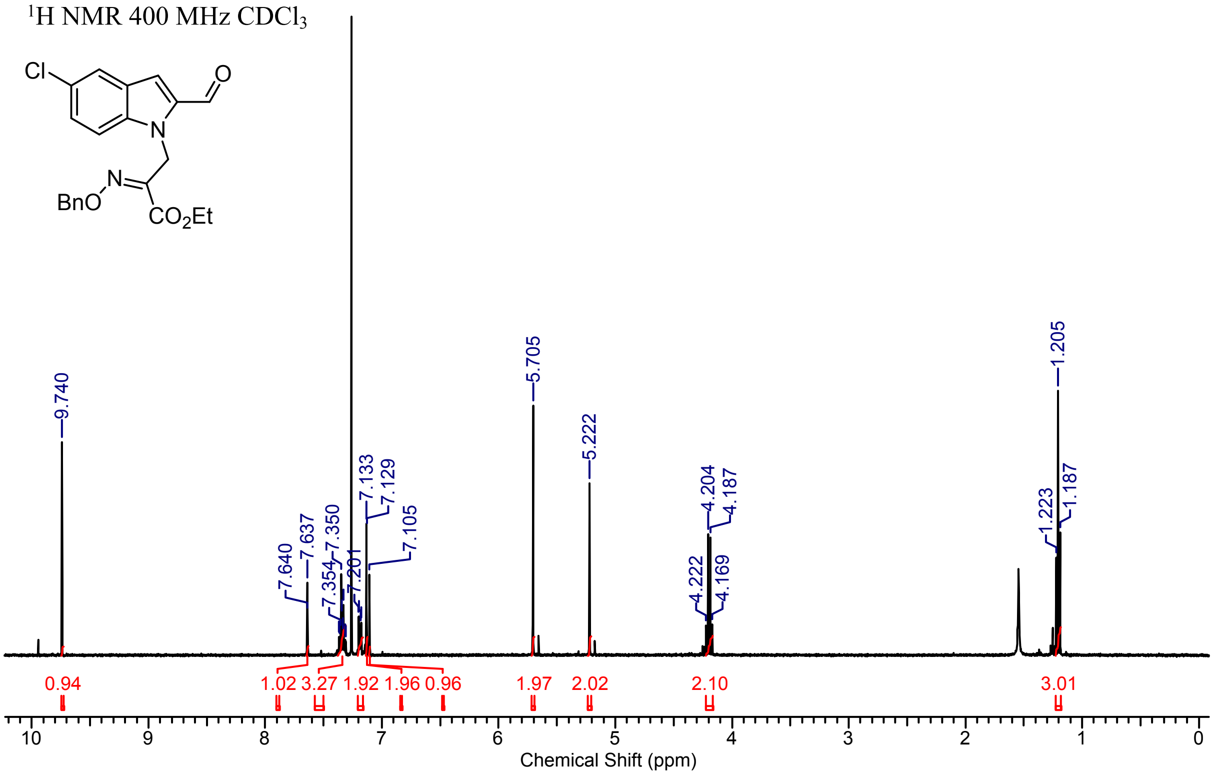


**Figure S10**. $^{1}$H spectrum of compound **1e**.

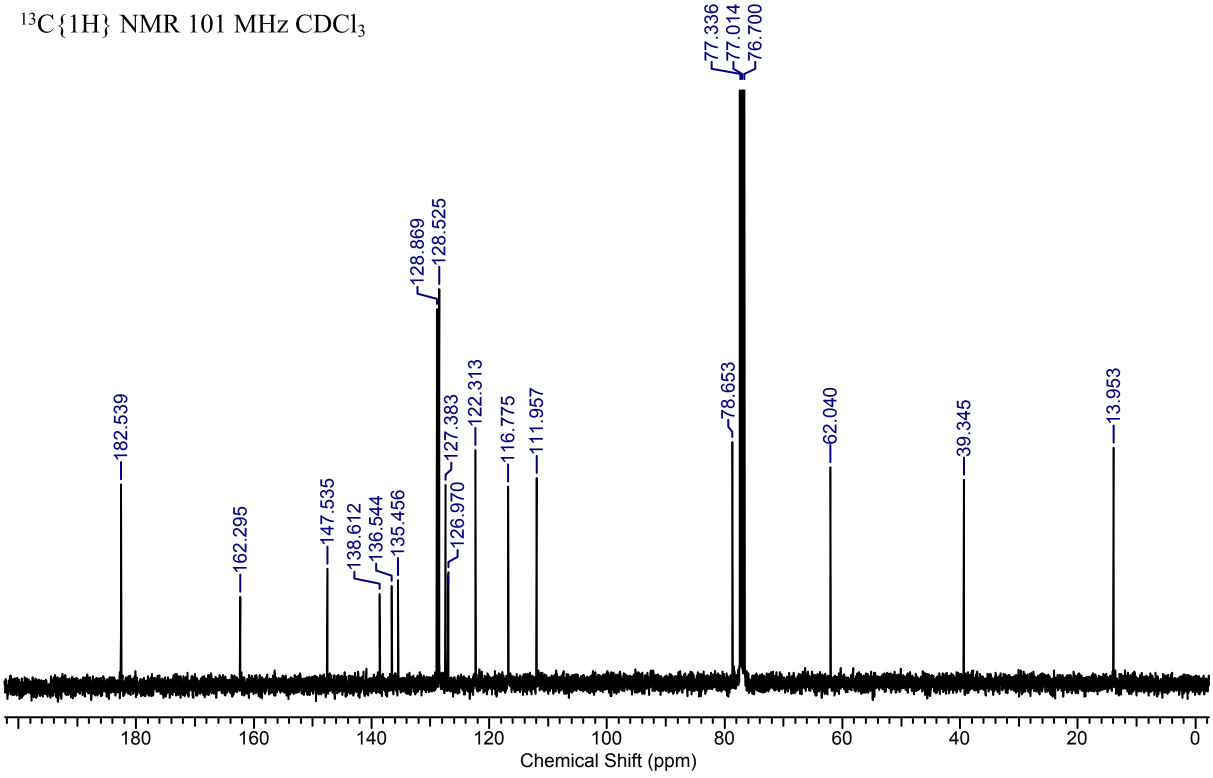


**Figure S11.** $^{13}$C spectrum of compound **1e**.

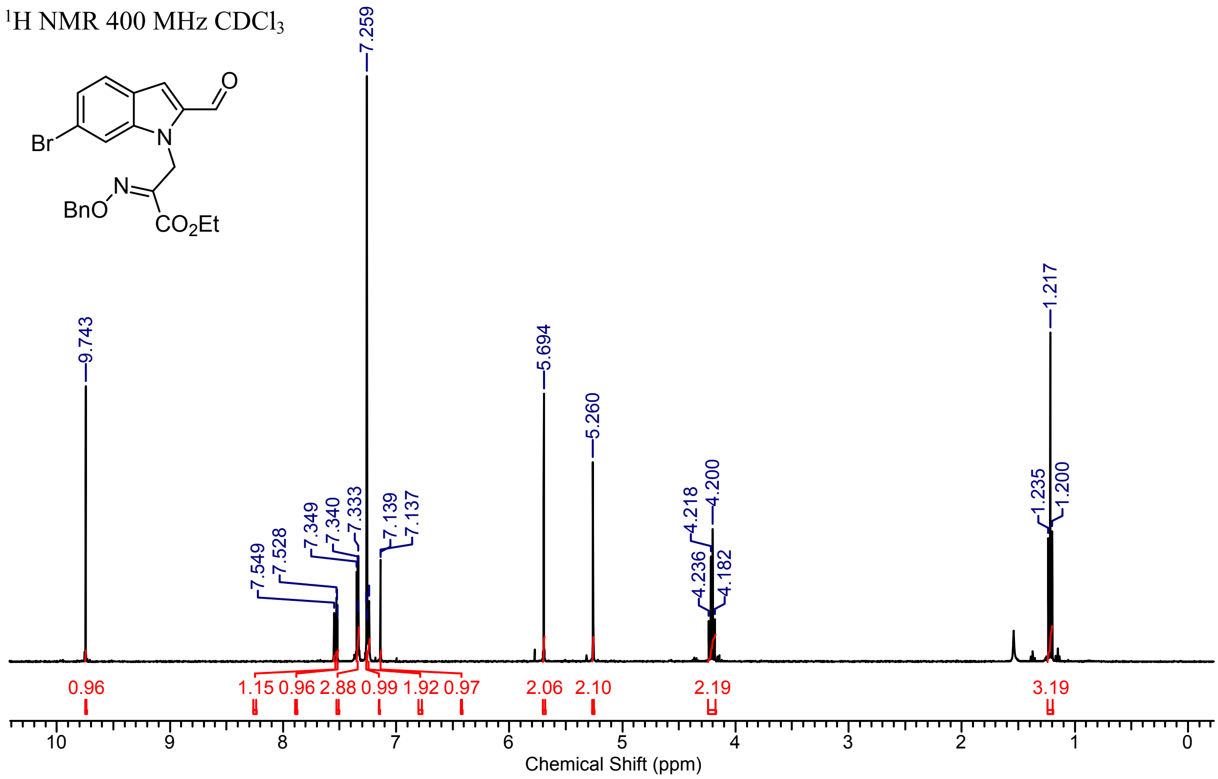


**Figure S12.** $^1$H spectrum of compound **1f**.

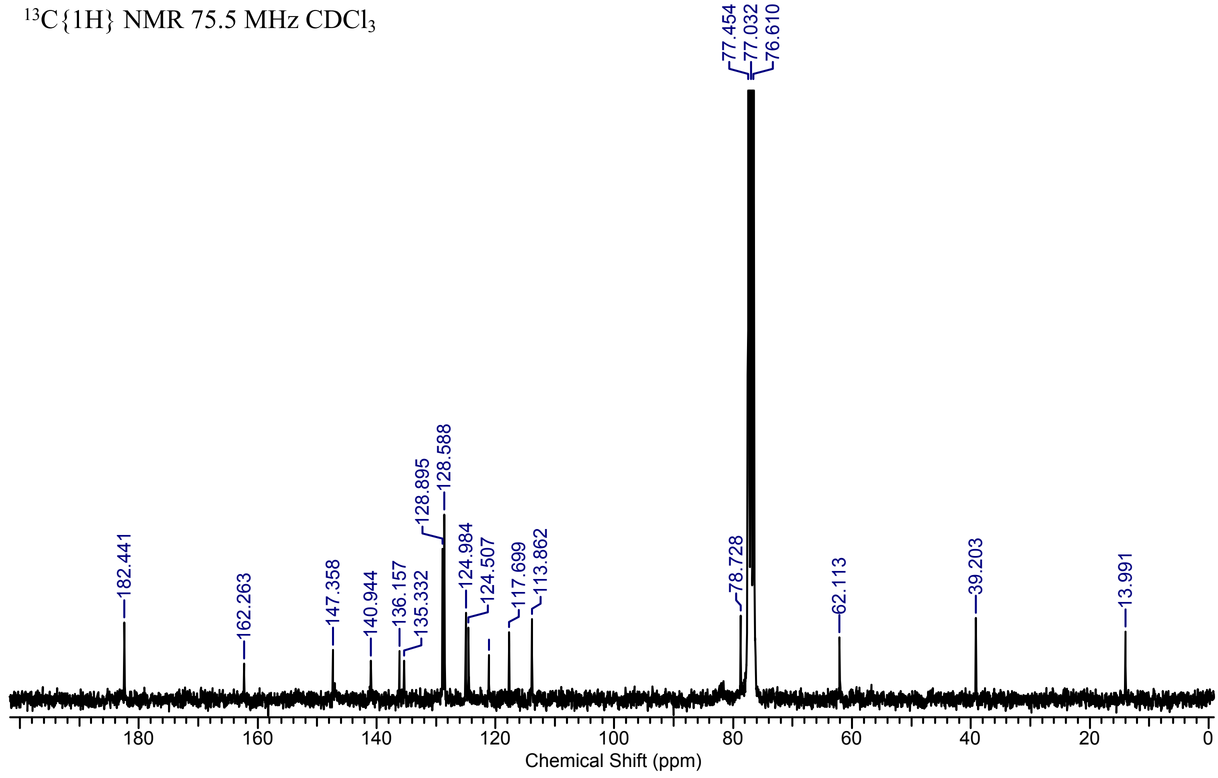


**Figure S13.** $^{13}$C spectrum of compound **1f**.

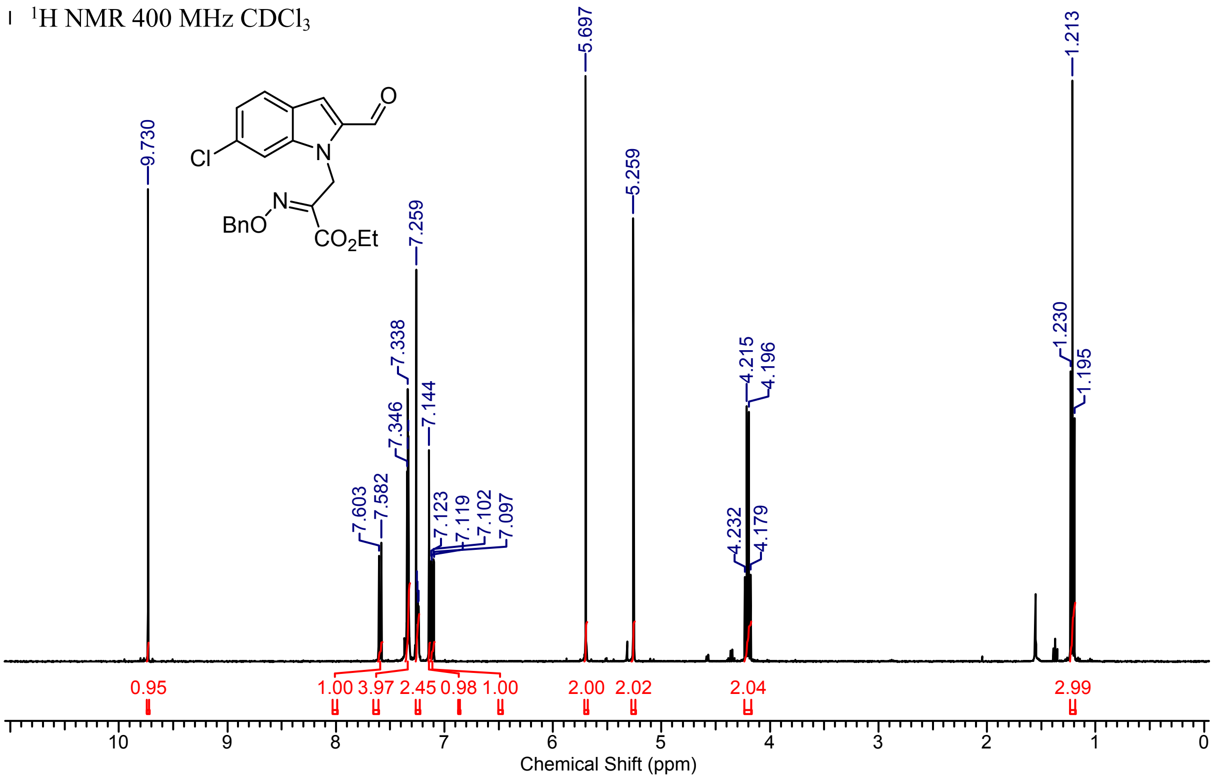


**Figure S14.** $^{1}H$ spectrum of compound **1g**.

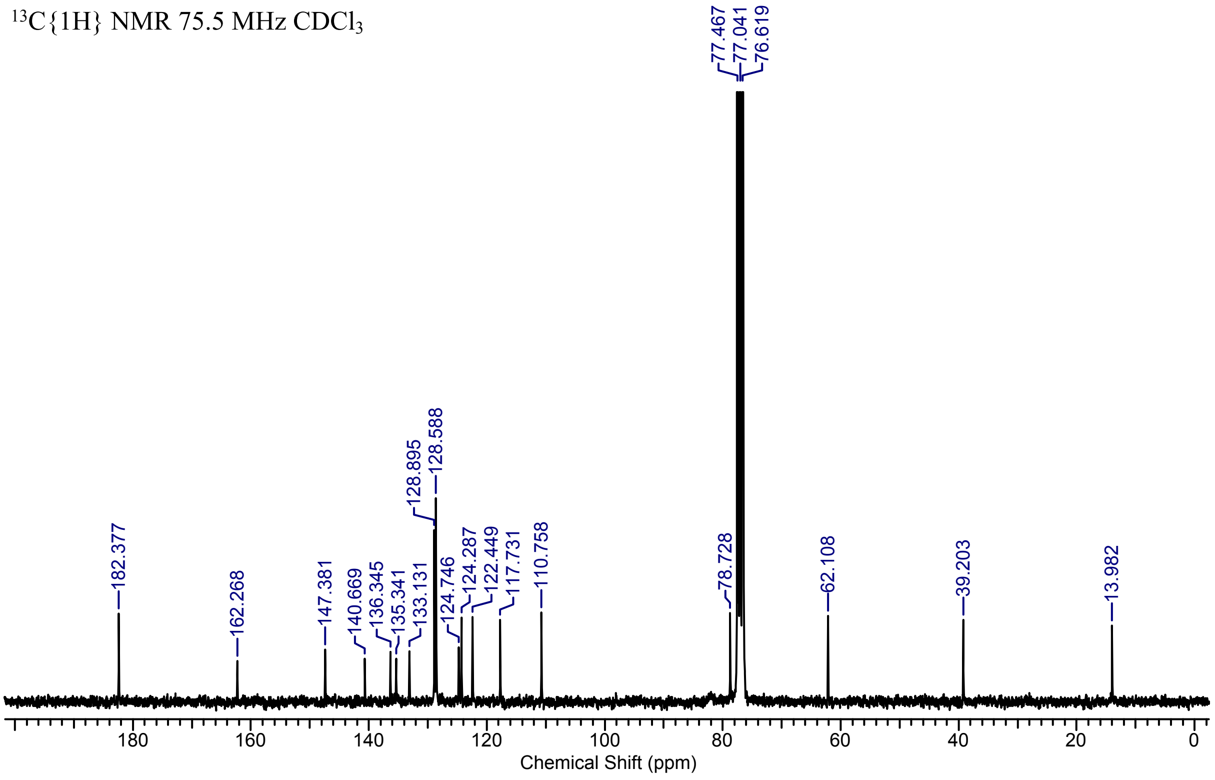


**Figure S15.** $^{13}C$ spectrum of compound **1g**.

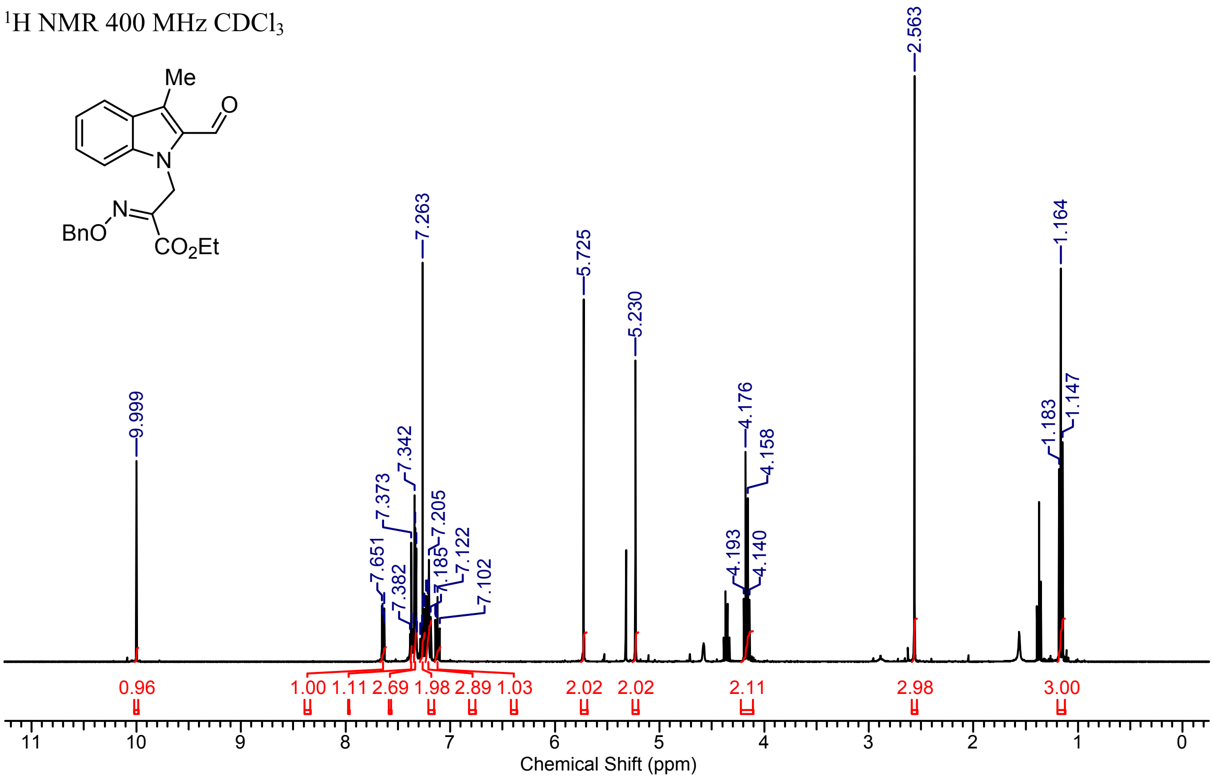


**Figure S16.** $^{1}H$ spectrum of compound **1h**.

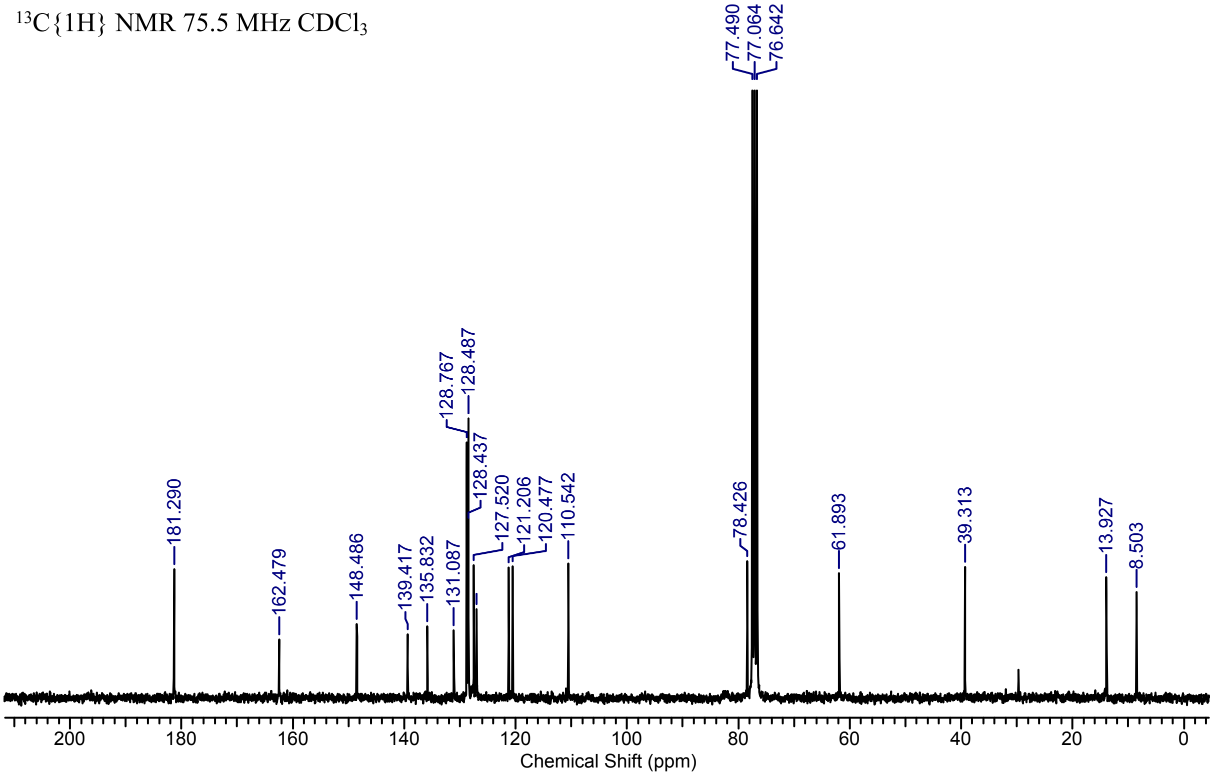


**Figure S17.** $^{13}C$ spectrum of compound **1h**.

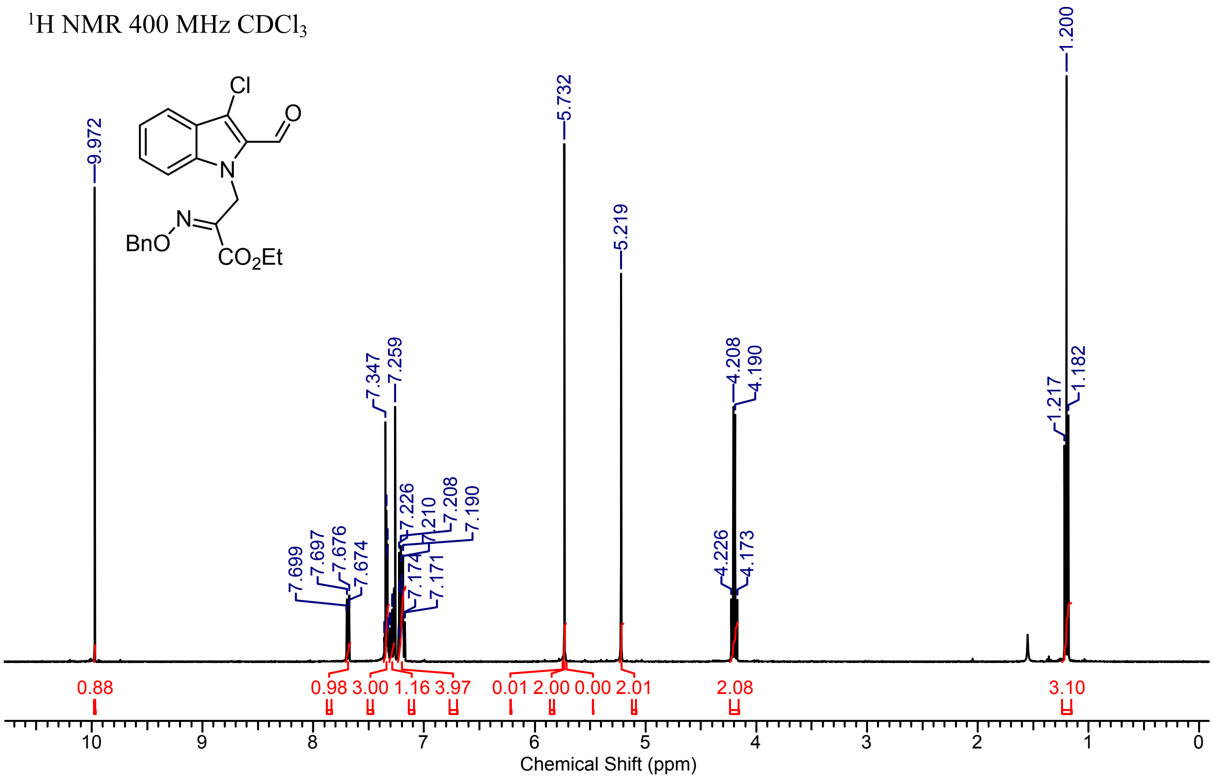


**Figure S18.** $^{1}H$ spectrum of compound **1i**.

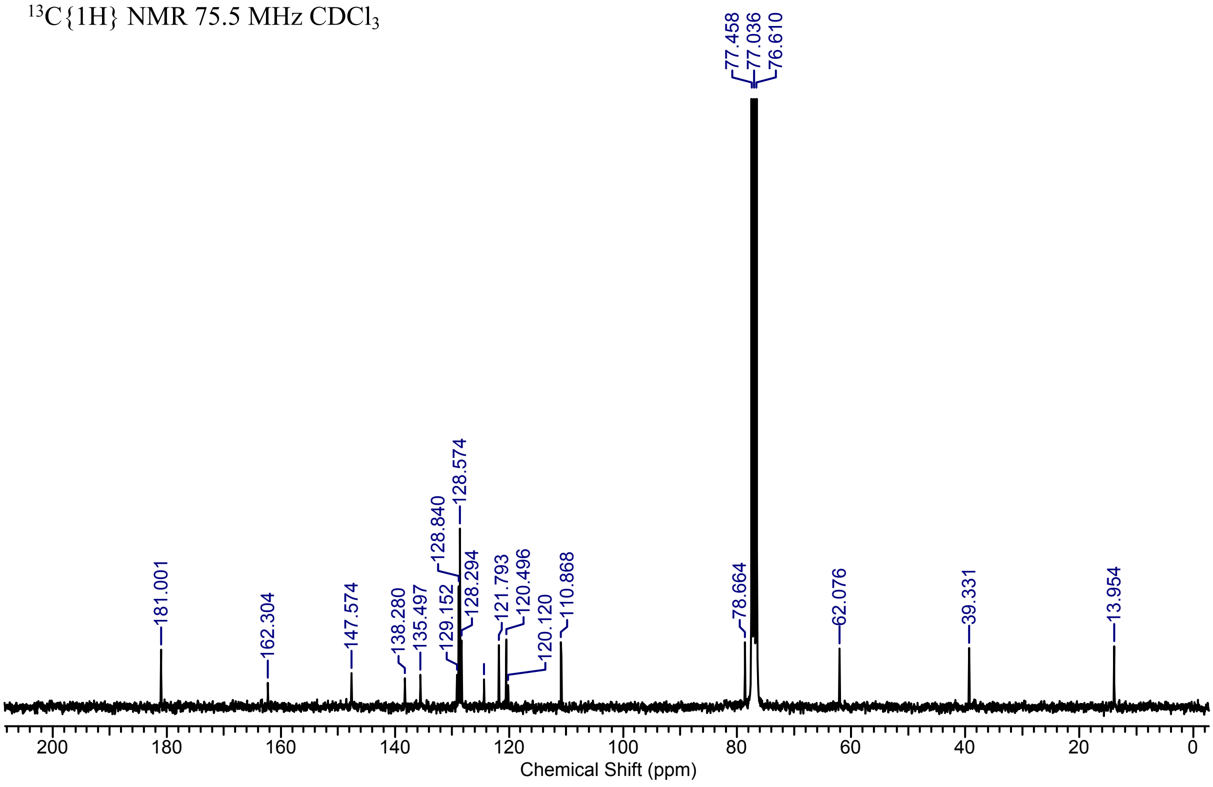


**Figure S19.** $^{13}C$ spectrum of compound **1i**.

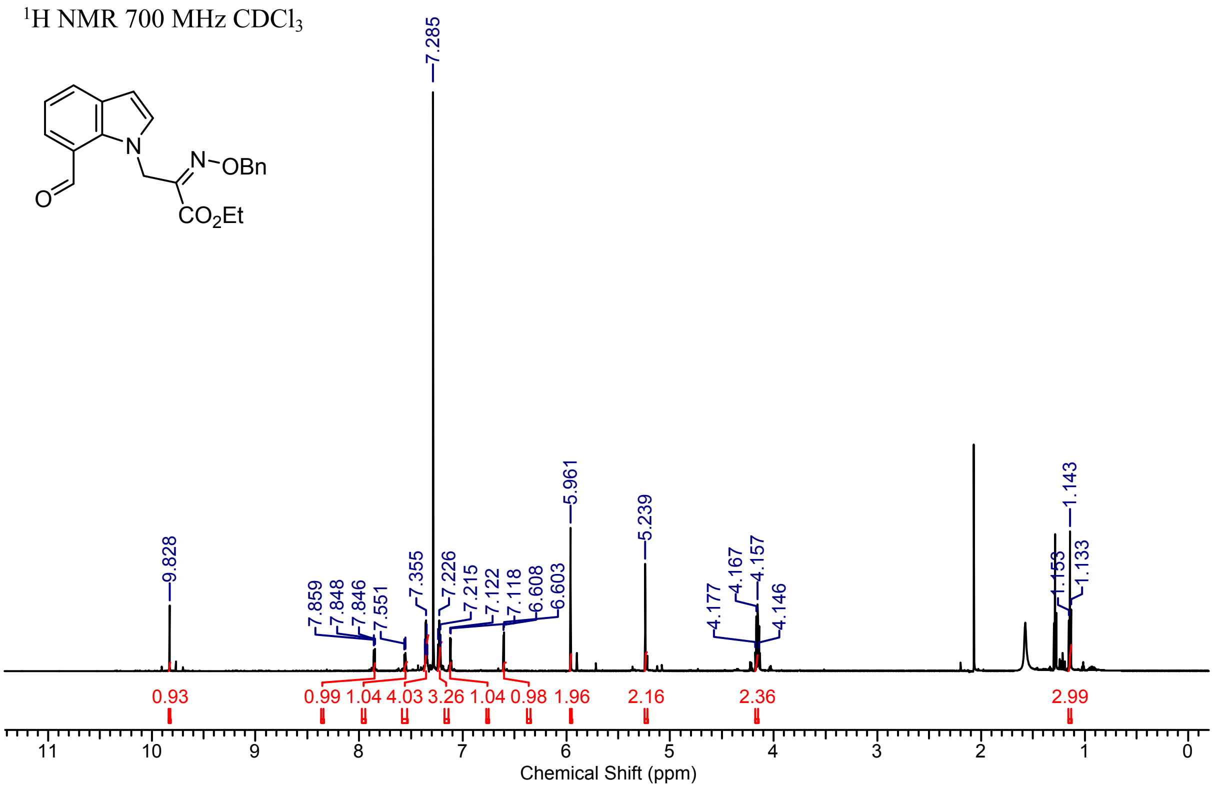


**Figure S20.** $^{1}$H spectrum of compound **1j**.

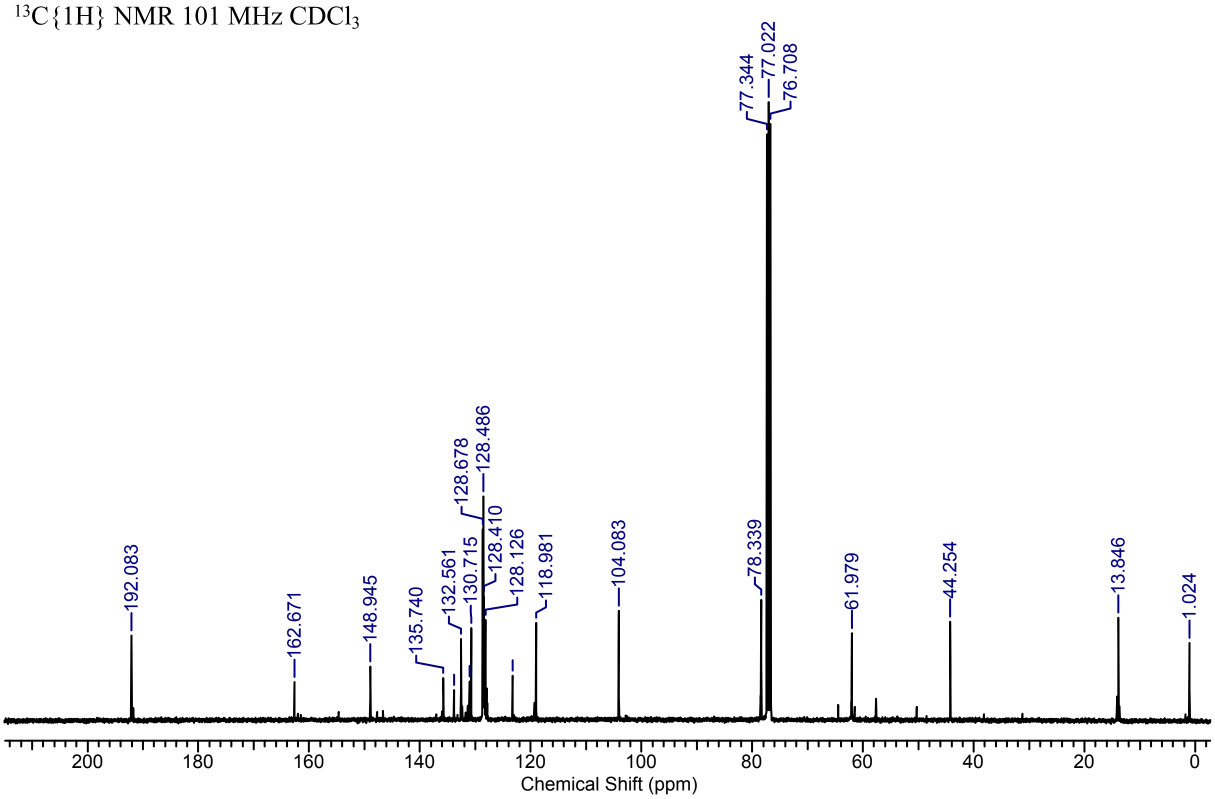


**Figure S21.** $^{13}$C spectrum of compound **1j**.

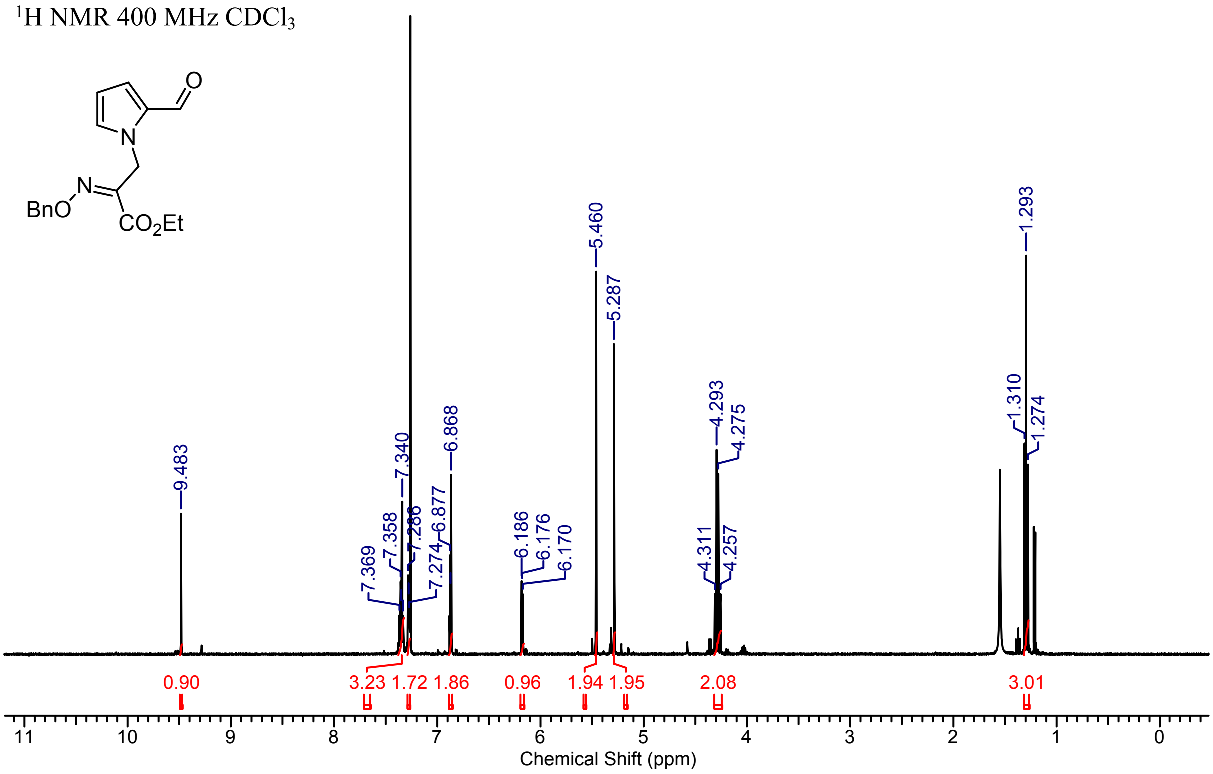


**Figure S22.** ¹H spectrum of compound **1k**.

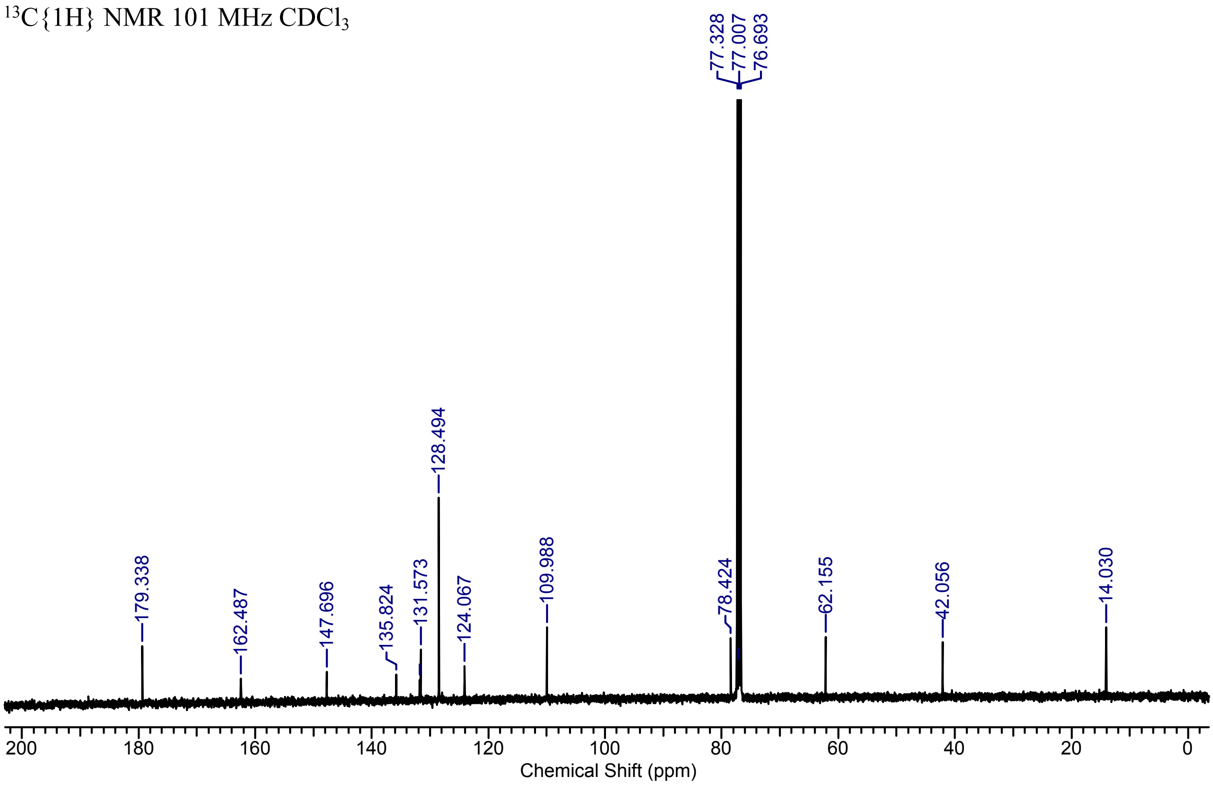


**Figure S23.** ¹³C spectrum of compound **1k**.

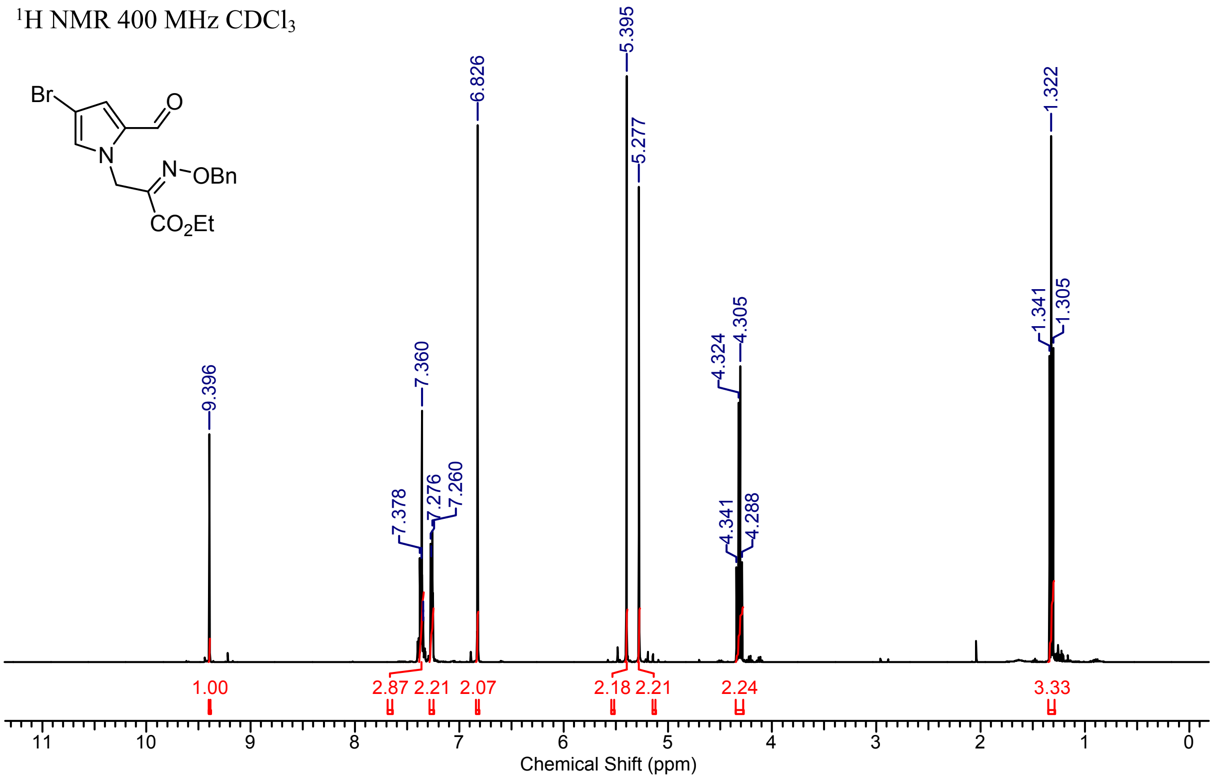


**Figure S24.** $^1$H spectrum of compound **1l**.

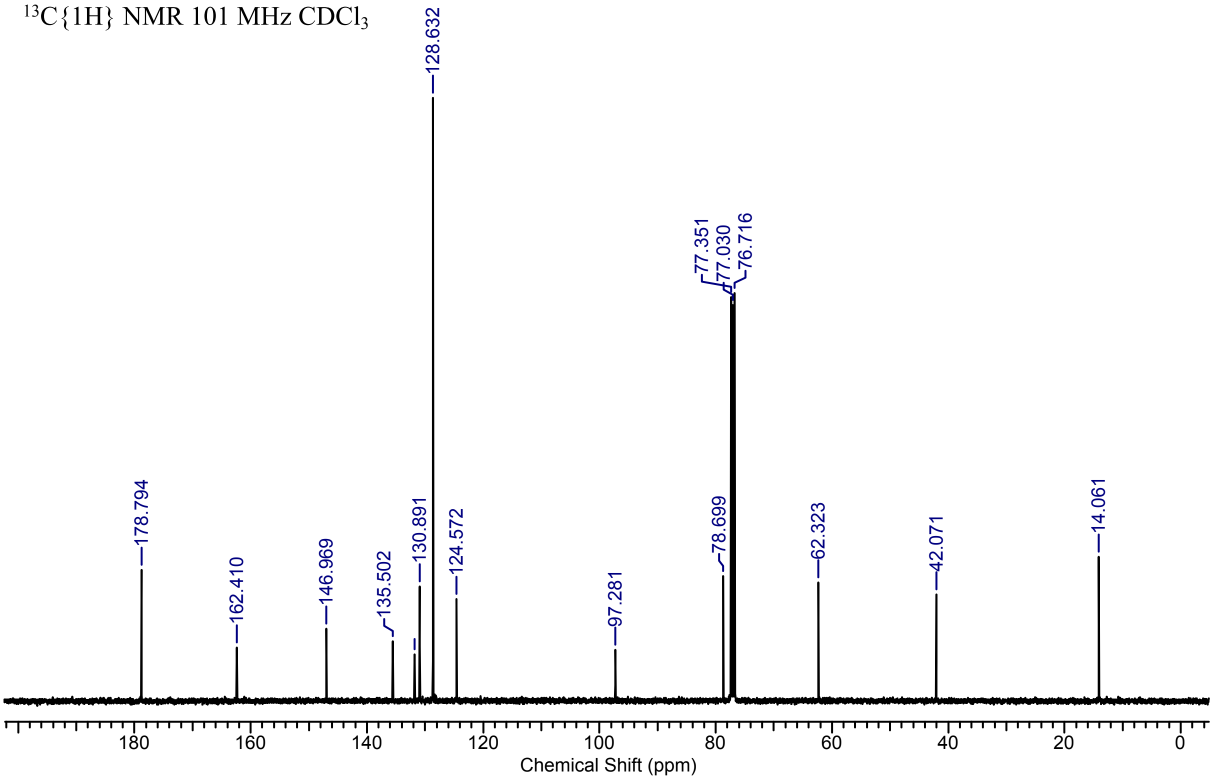


**Figure S25.** $^{13}$C spectrum of compound **1l**.

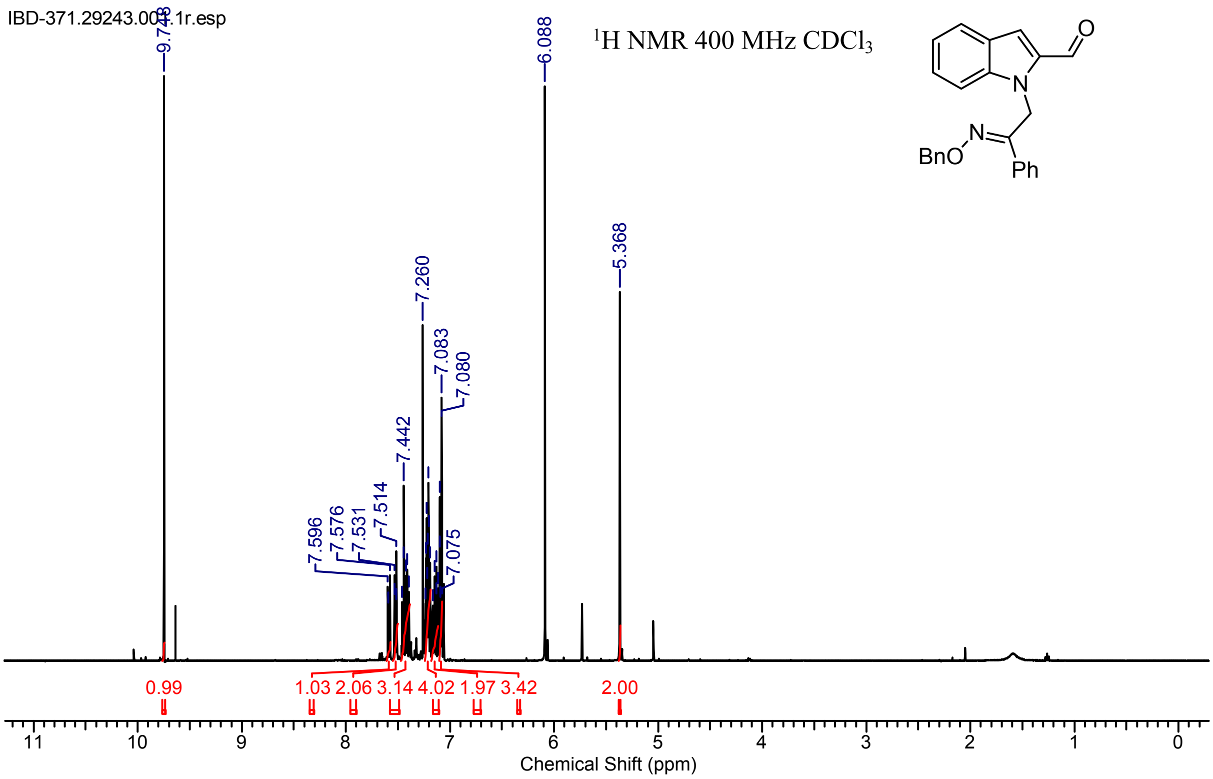


**Figure S26.** $^{13}$C spectrum of compound **1m**.

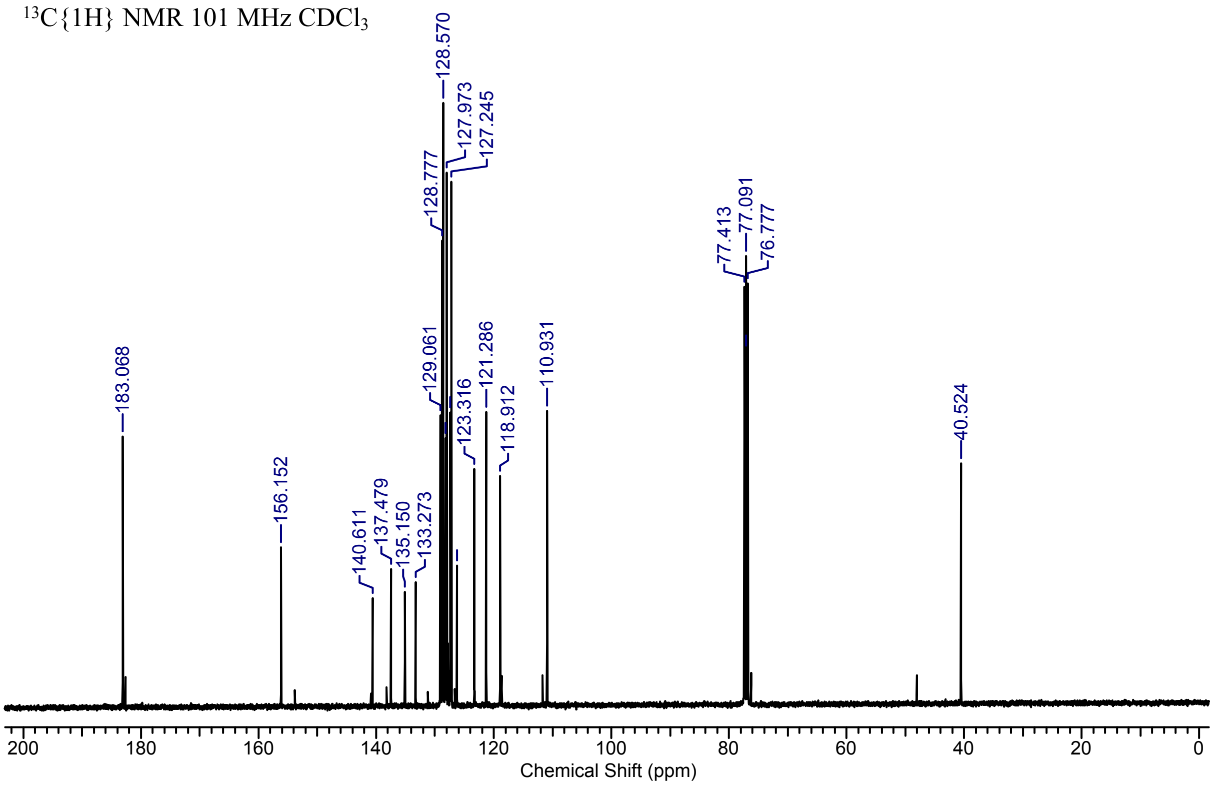


**Figure S27.** $^{13}$C spectrum of compound **1m**.

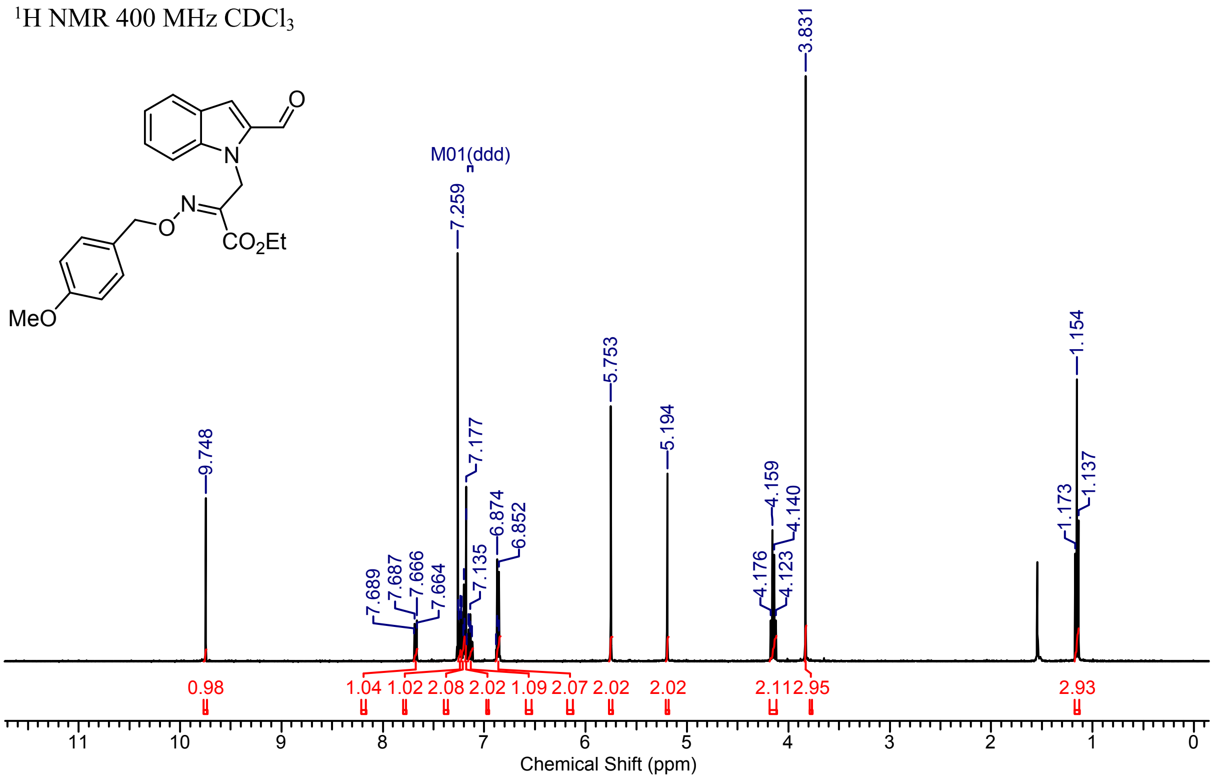


**Figure S28.** $^{1}$H spectrum of compound **1n**.

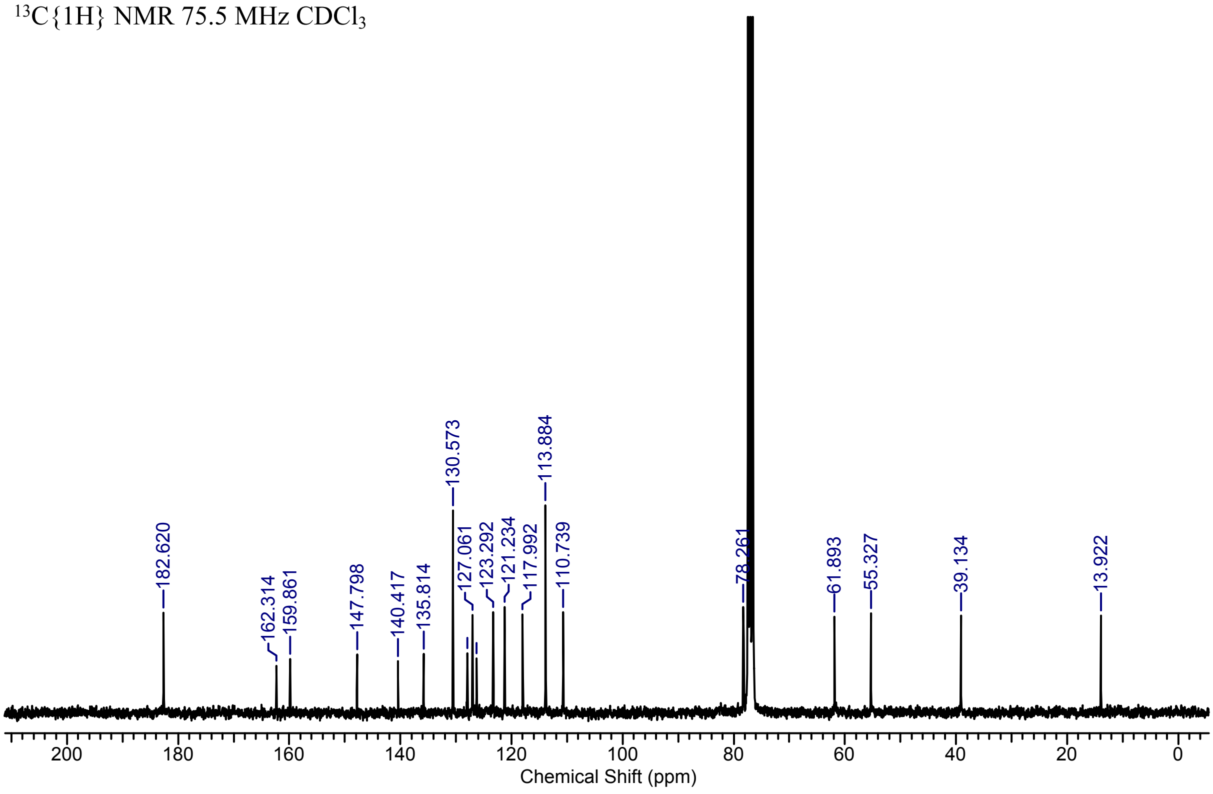


**Figure S29.** $^{13}$C spectrum of compound **1n**.

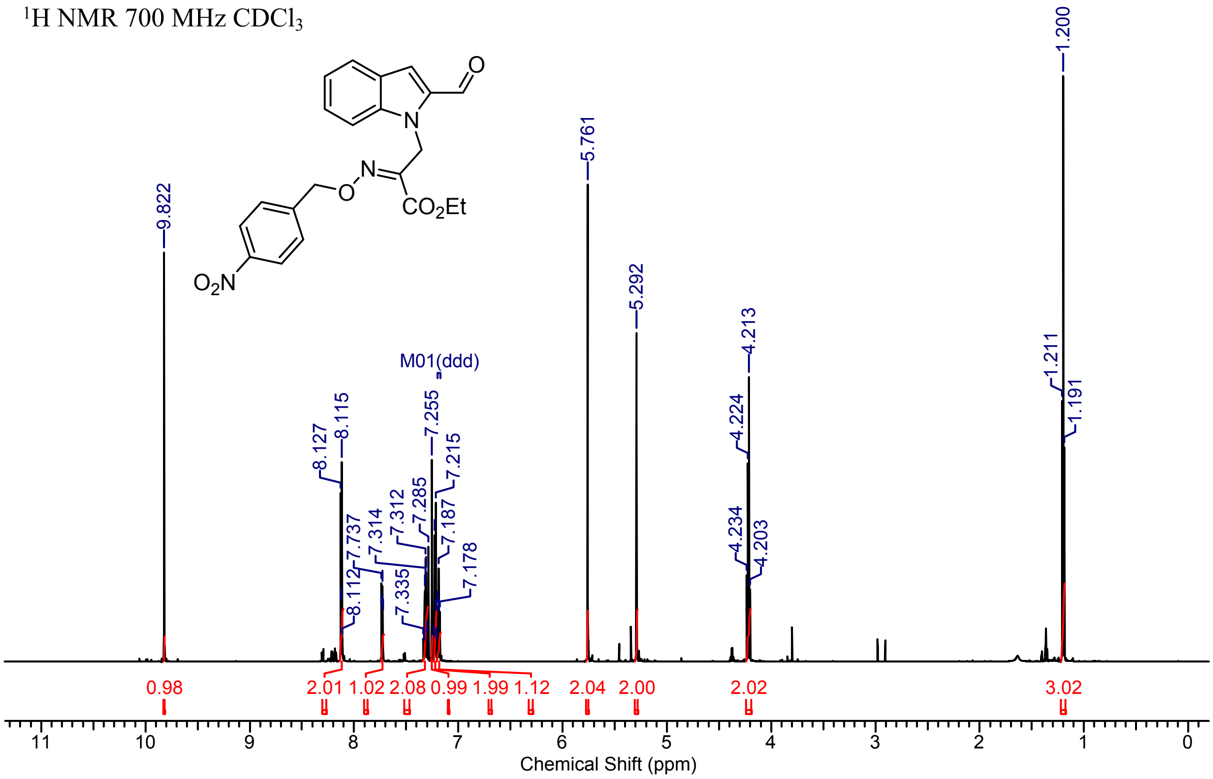


**Figure S30.** $^{1}$H spectrum of compound **1o**.

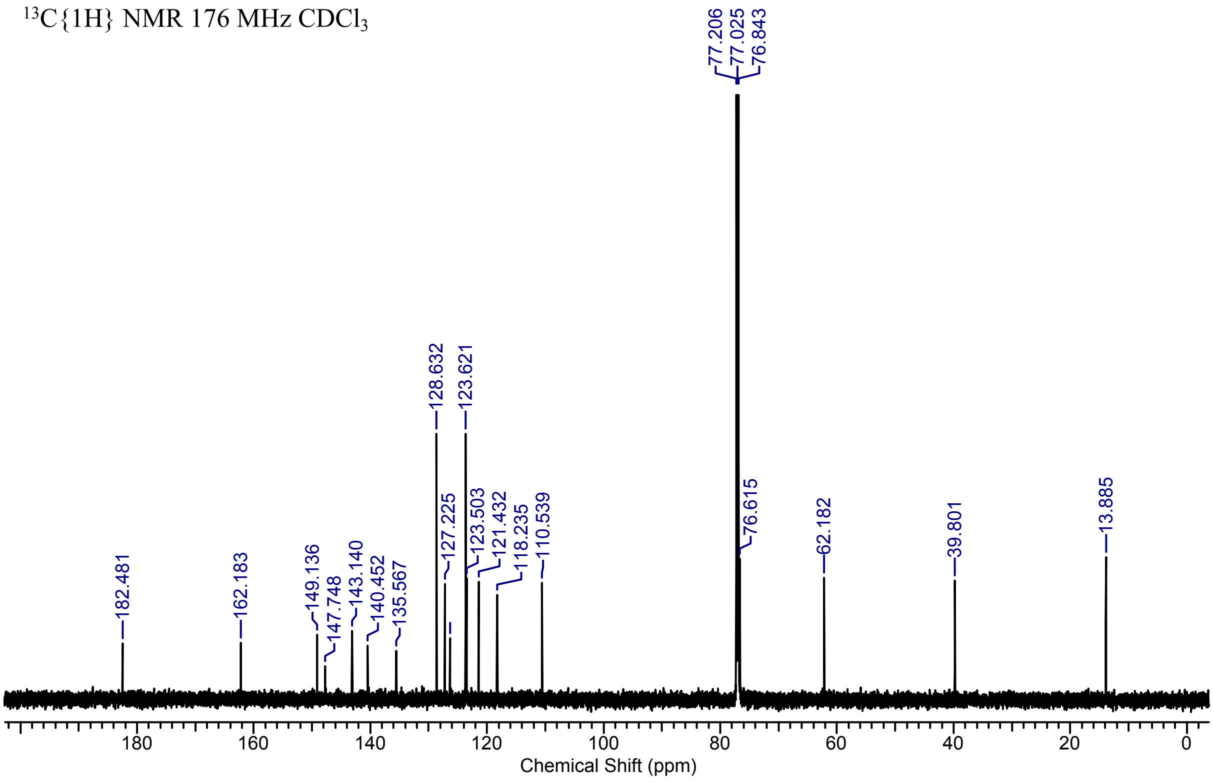


**Figure S31.** $^{13}$C spectrum of compound **1o**.

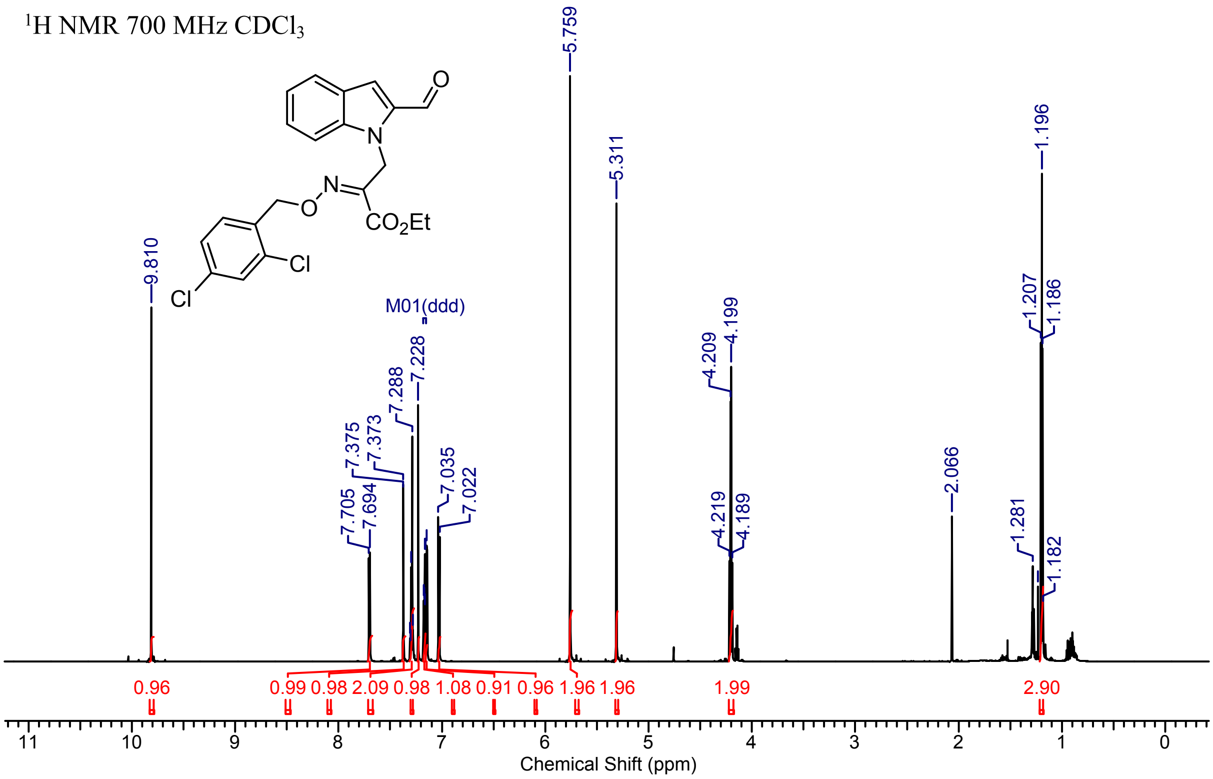


**Figure S32.** $^{1}$H spectrum of compound **1p**.

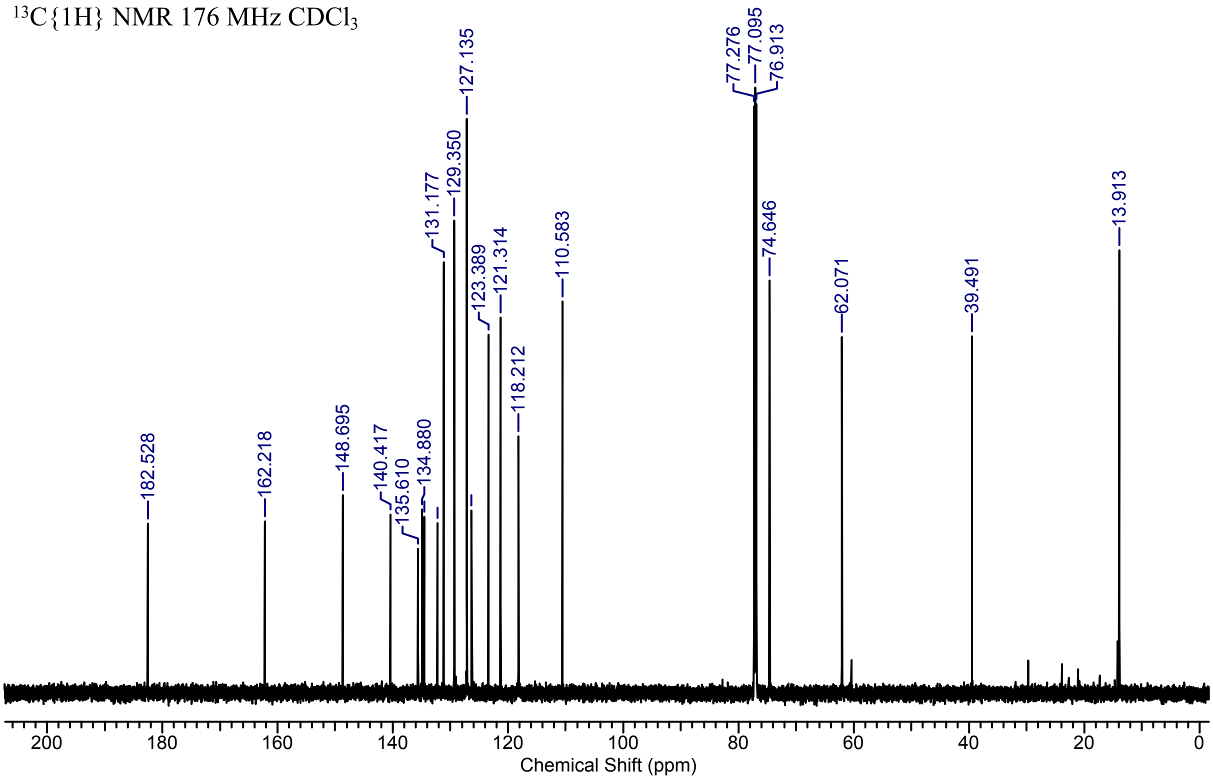


**Figure S33.** $^{13}$C spectrum of compound **1p**.

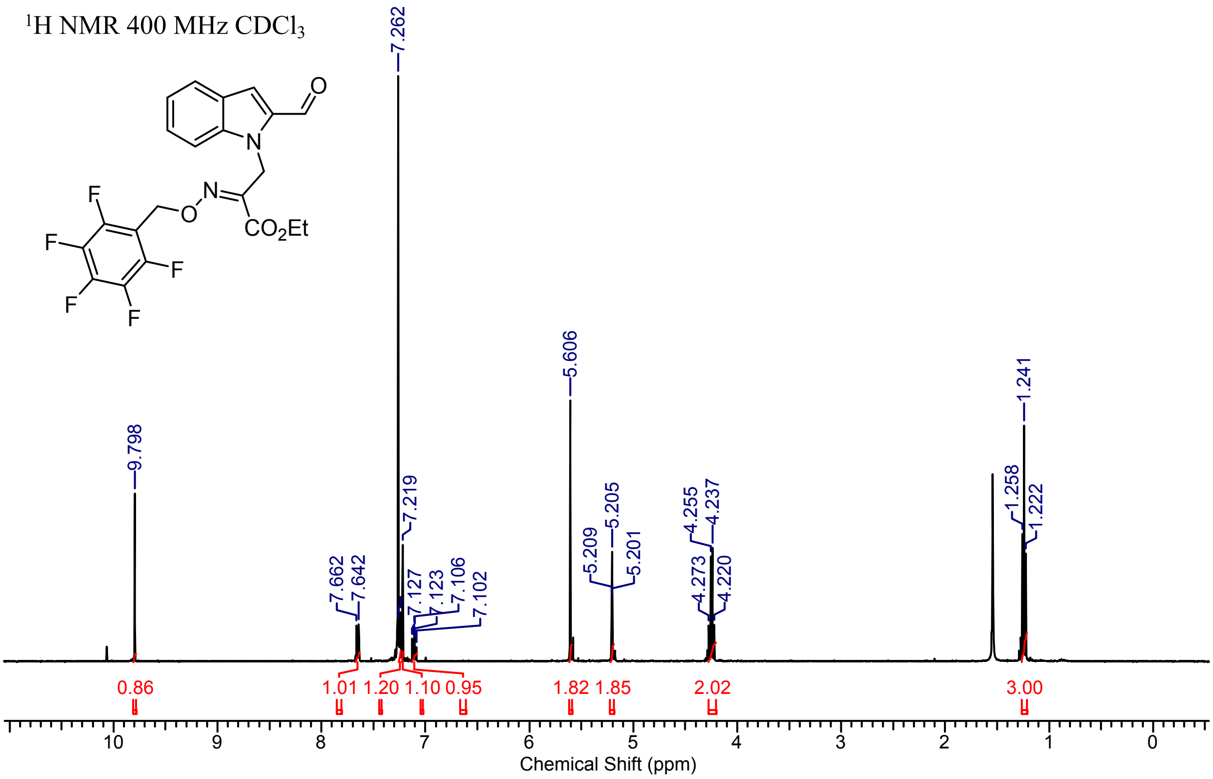


**Figure S34.** $^1$H spectrum of compound **1q**.

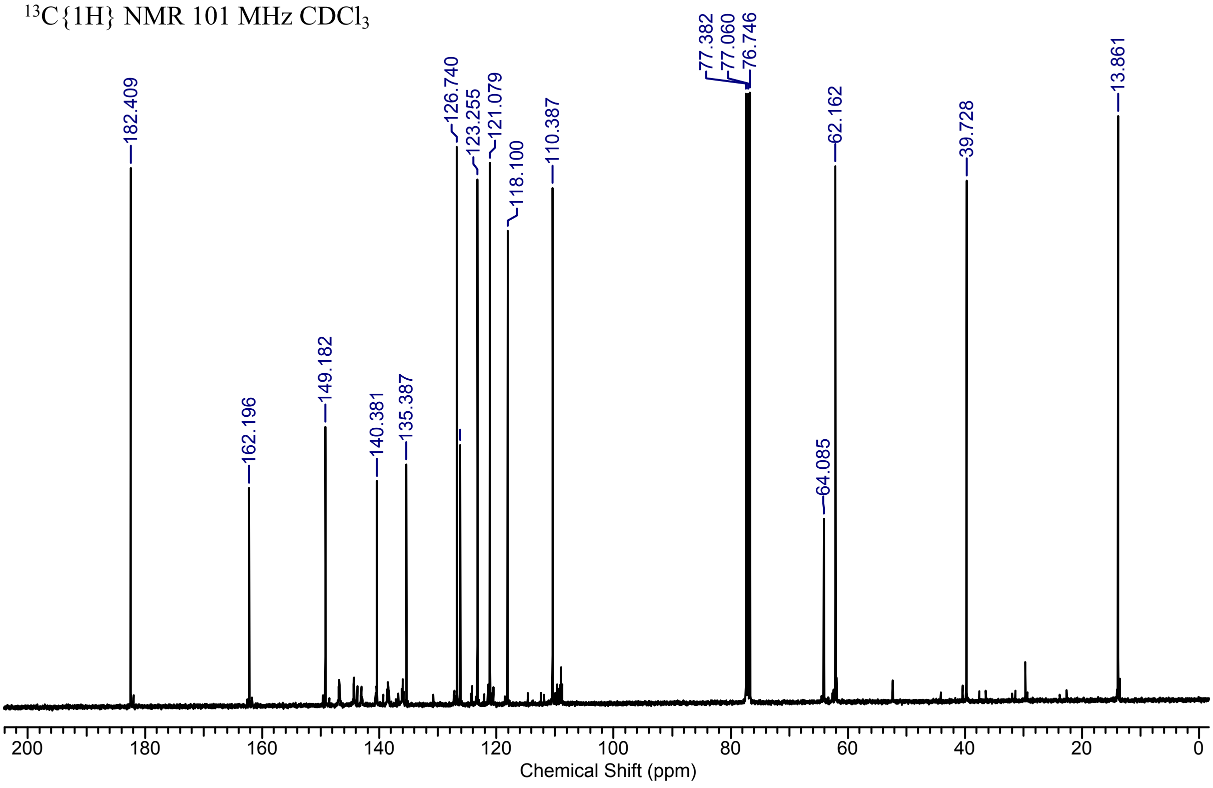


**Figure S35.** $^{13}$C spectrum of compound **1q**.

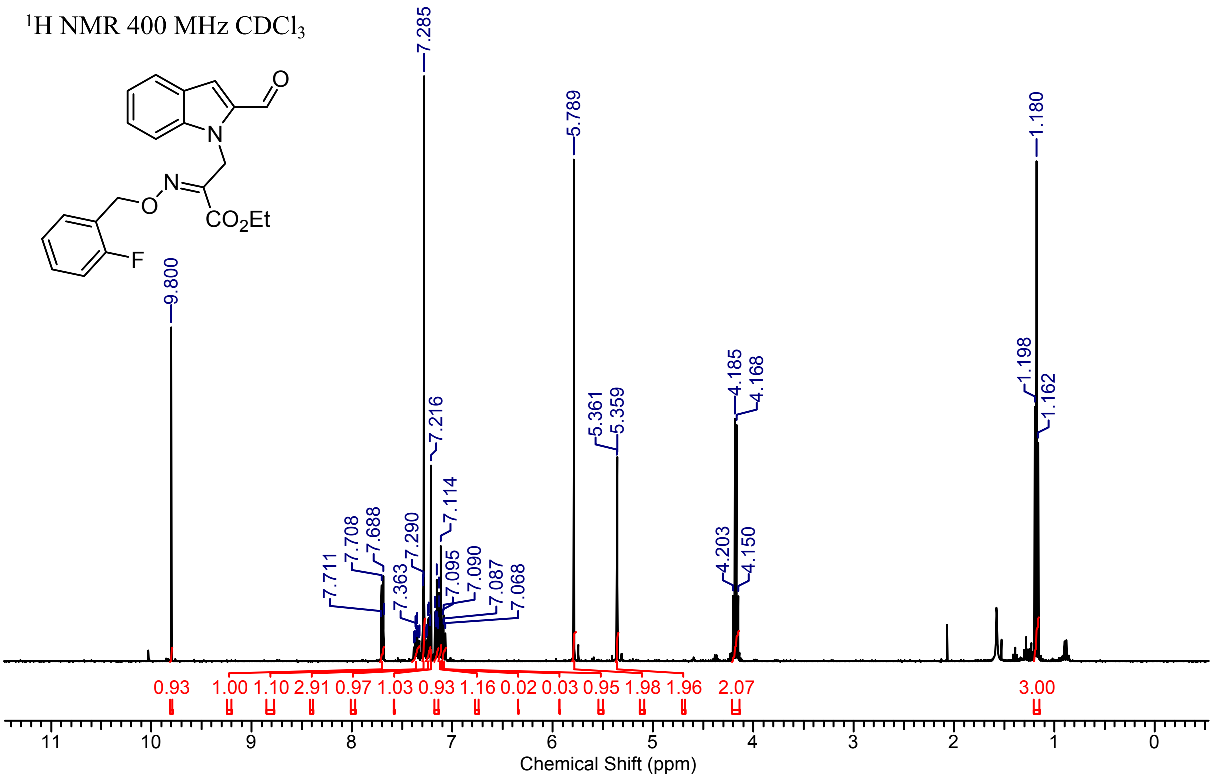


**Figure S36.** $^1H$ spectrum of compound **1r**.

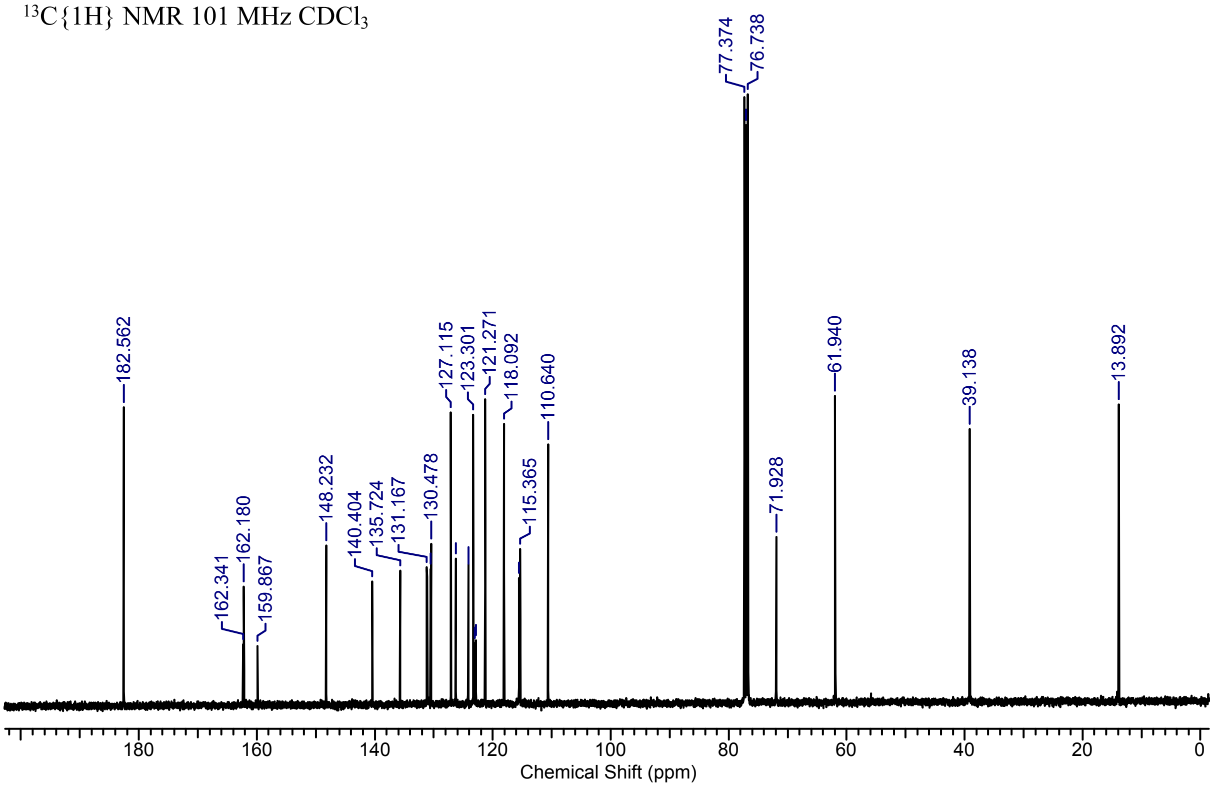


**Figure S37.** $^{13}C$ spectrum of compound **1r**.

$^1H$ NMR 400 MHz $CDCl_3$

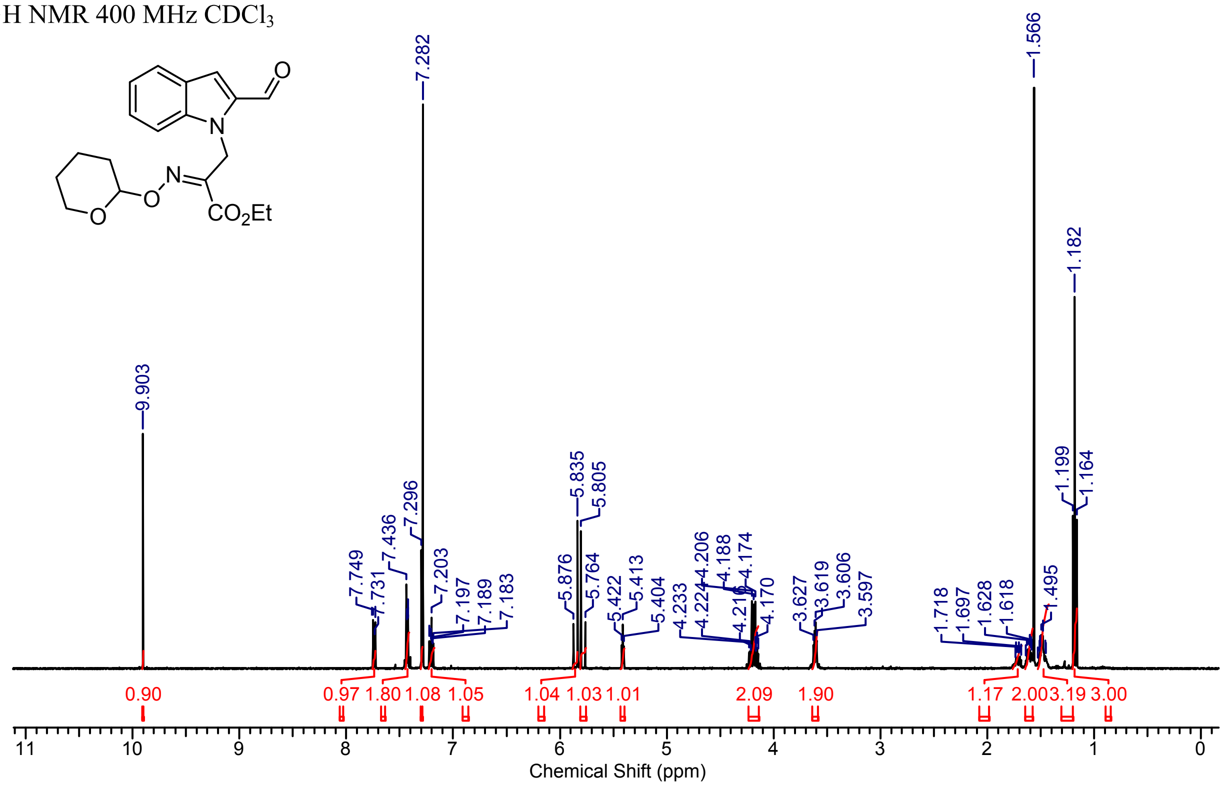

**Figure S38.** $^1H$ spectrum of compound **1s**.

$^{13}C\{1H\}$ NMR 176 MHz $CDCl_3$

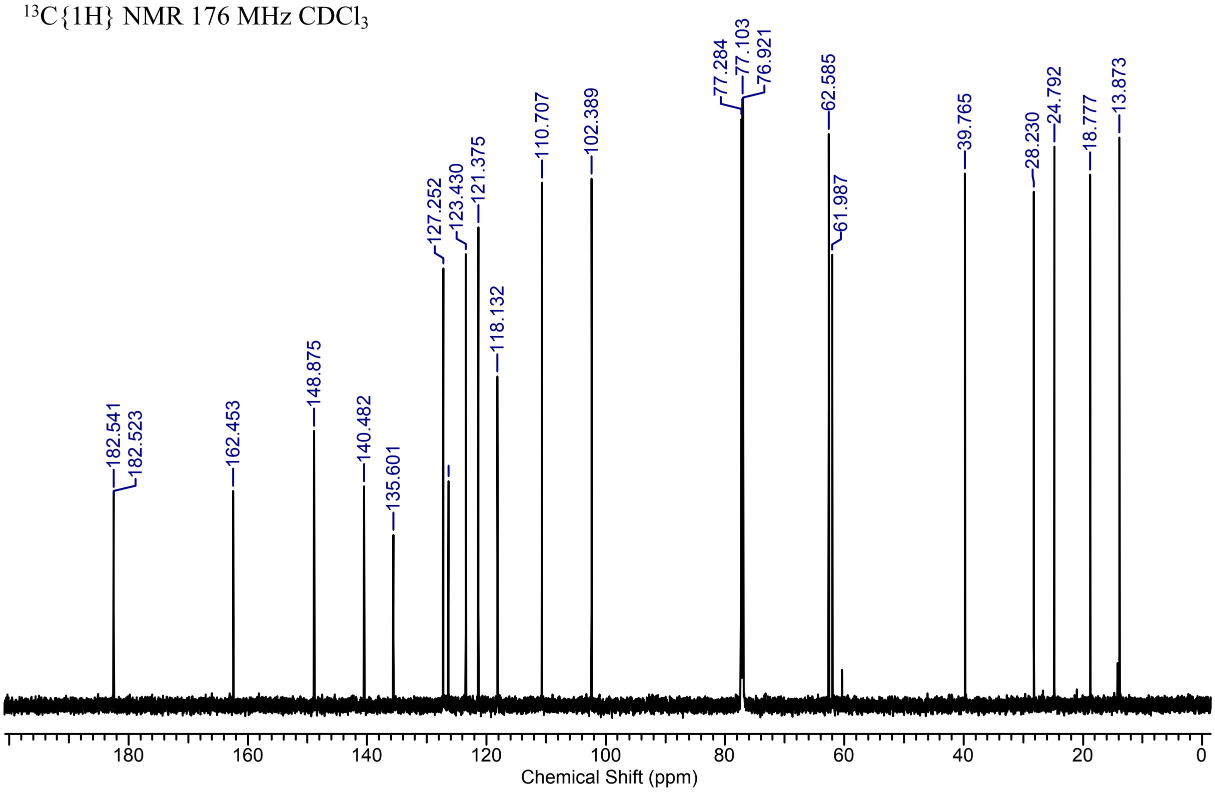

**Figure S39.** $^{13}C$ spectrum of compound **1s**.

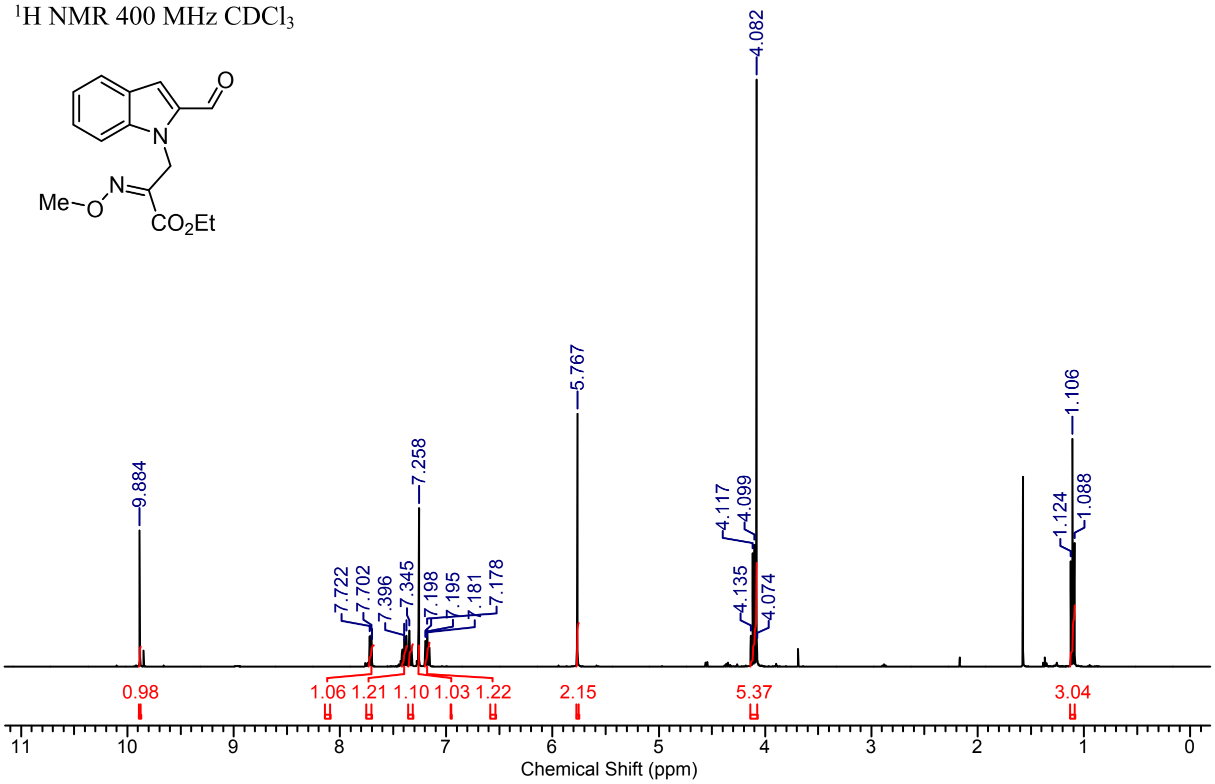


**Figure S40.** ¹H spectrum of compound **1t**.

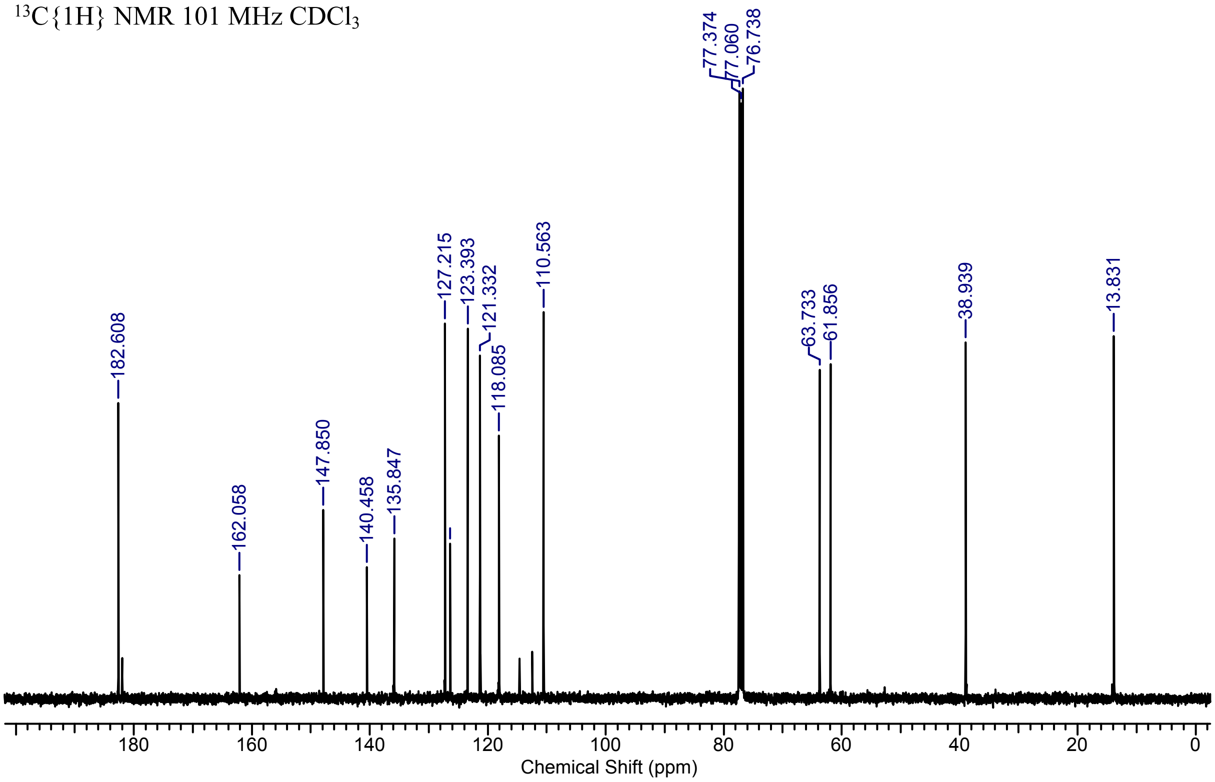


**Figure S41.** ¹³C spectrum of compound **1t**.

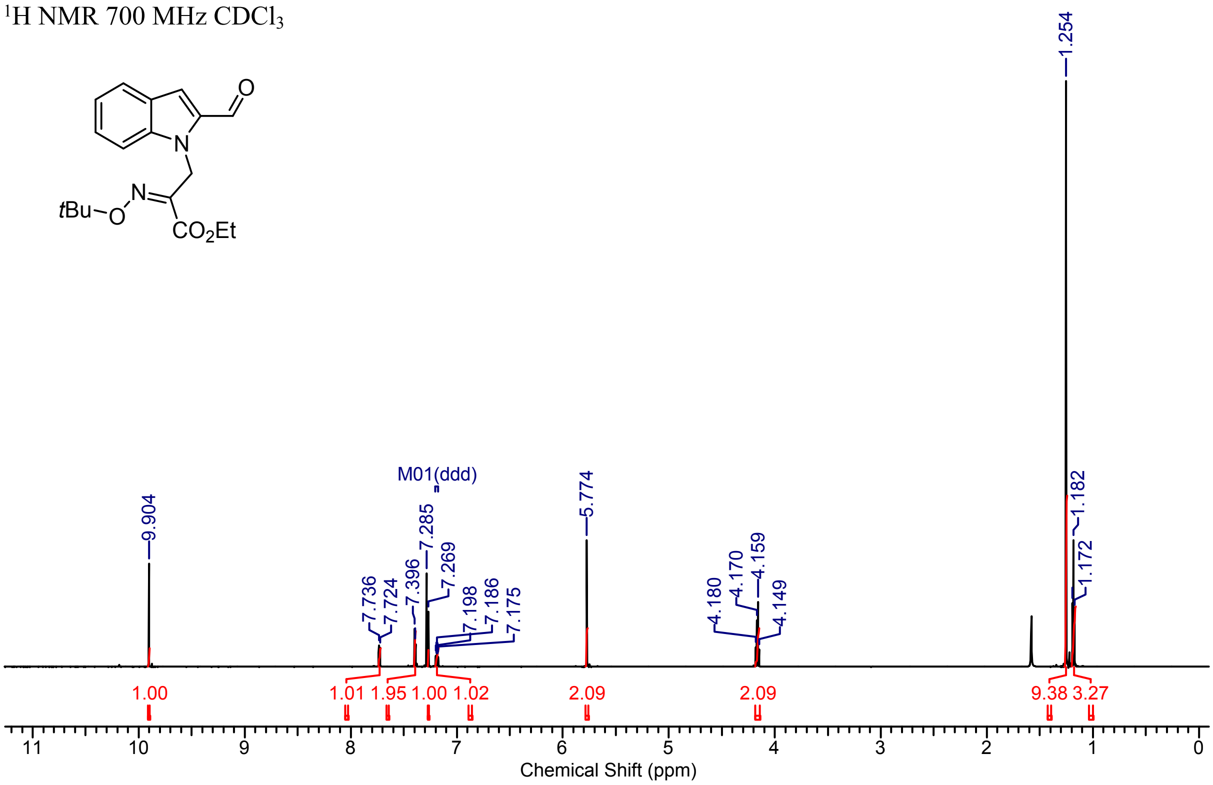


**Figure S42.** $^{1}H$ spectrum of compound **1u**.

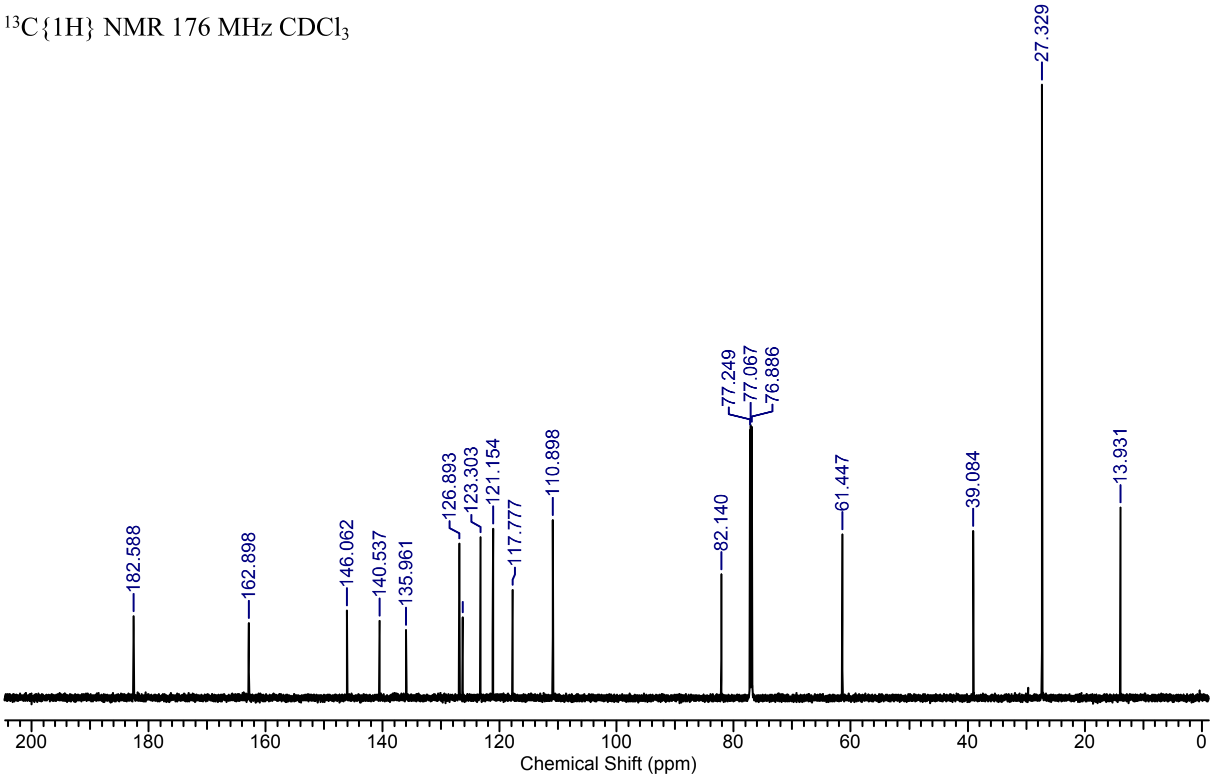


**Figure S43.** $^{13}C$ spectrum of compound **1u**.

$^{1}H$ NMR 700 MHz $CDCl_3$

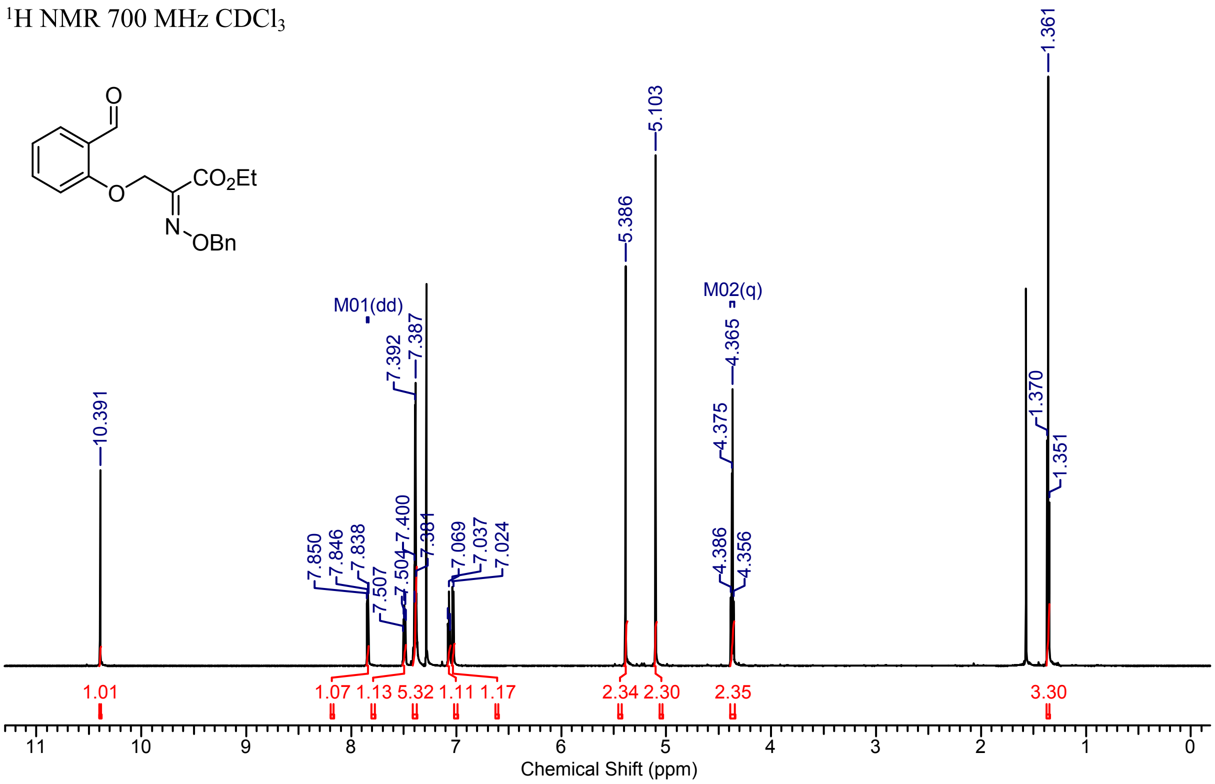


**Figure S44.** $^{1}H$ spectrum of compound **3a**.

$^{13}C\{1H\}$ NMR 101 MHz $CDCl_3$

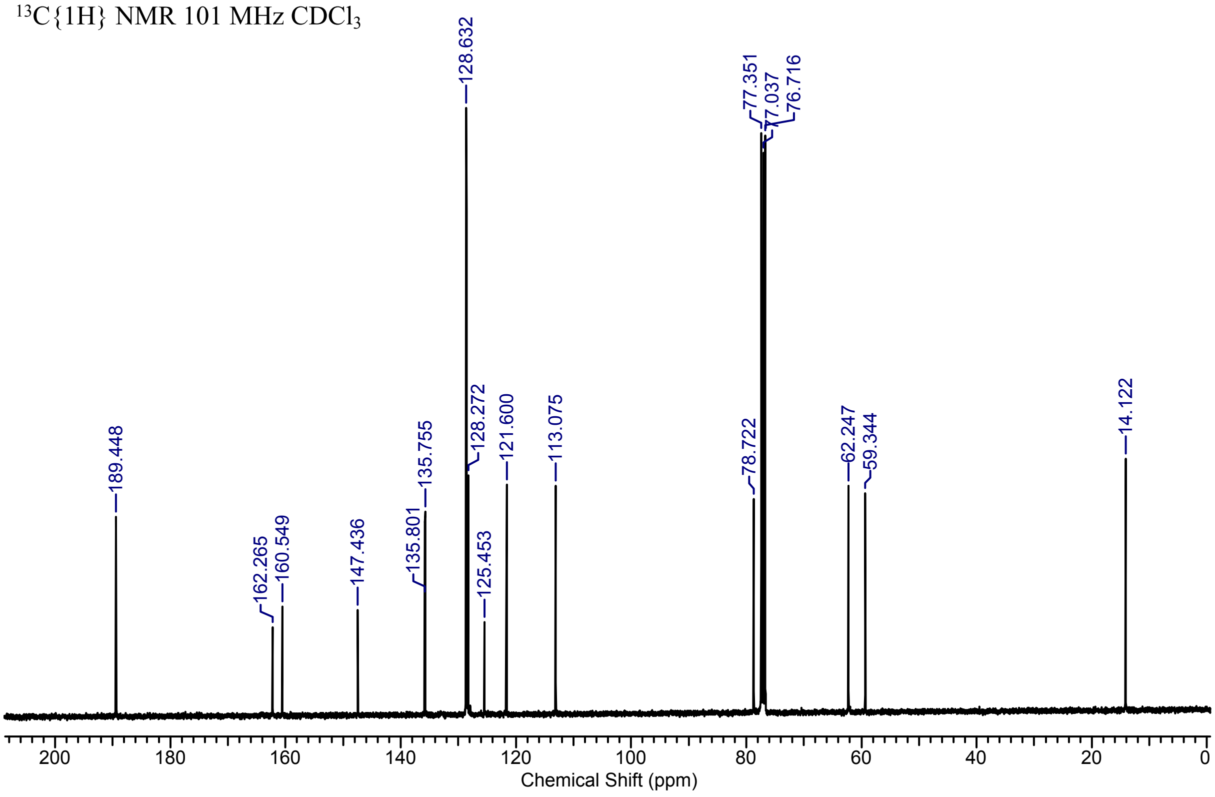


**Figure S45.** $^{13}C$ spectrum of compound **3a**.

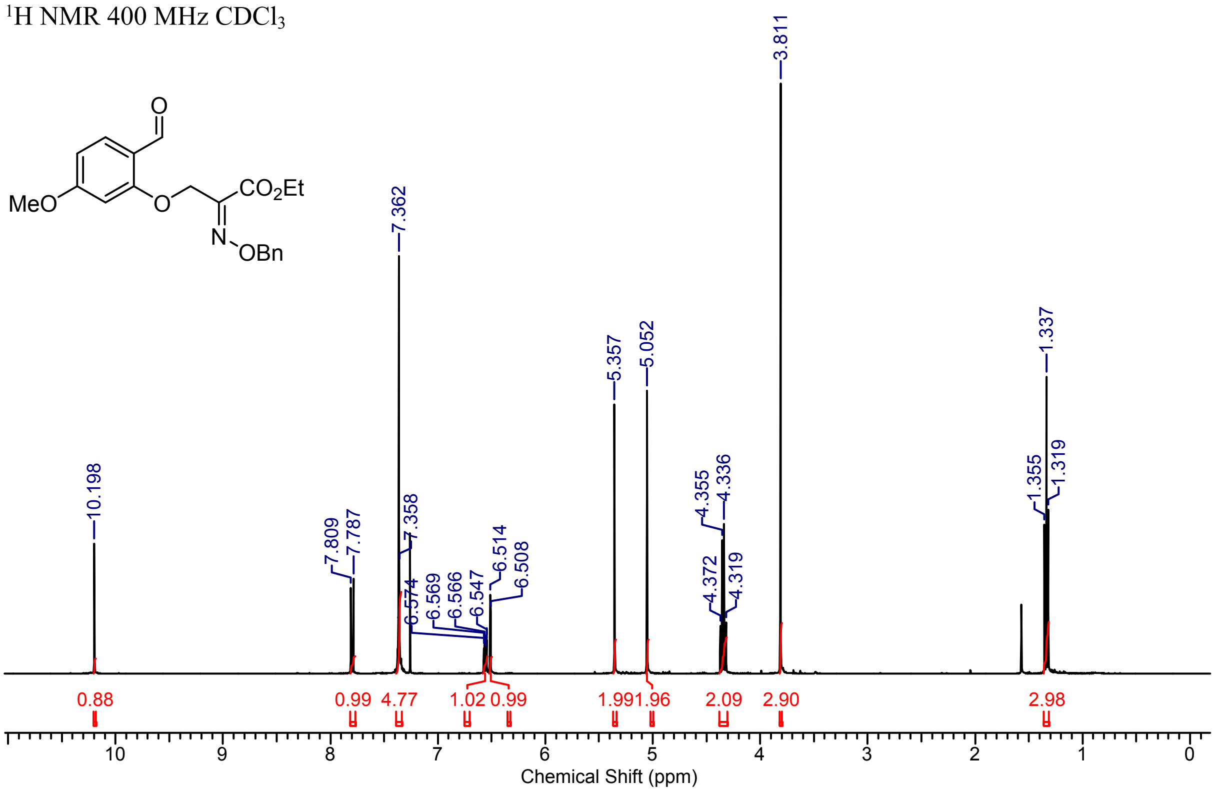


**Figure S46.** $^{1}H$ spectrum of compound **3b**.

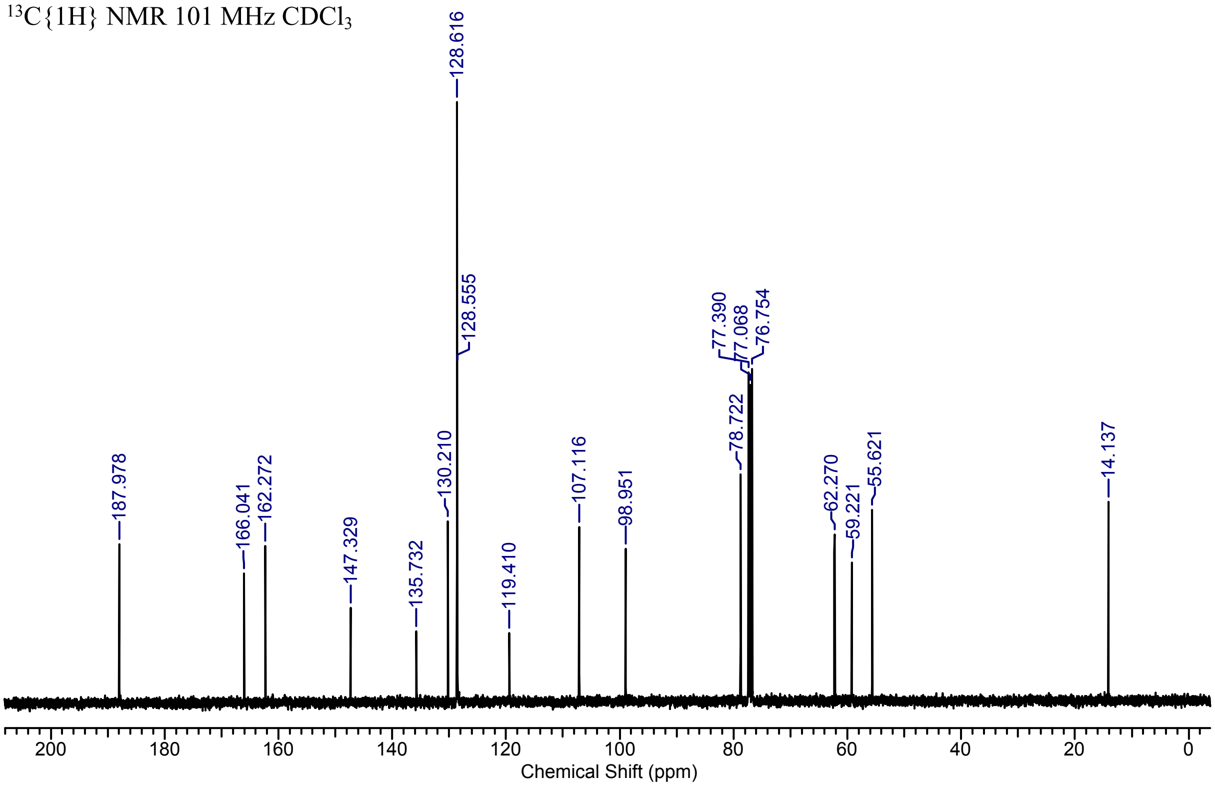


**Figure S47.** $^{13}C$ spectrum of compound **3b**.

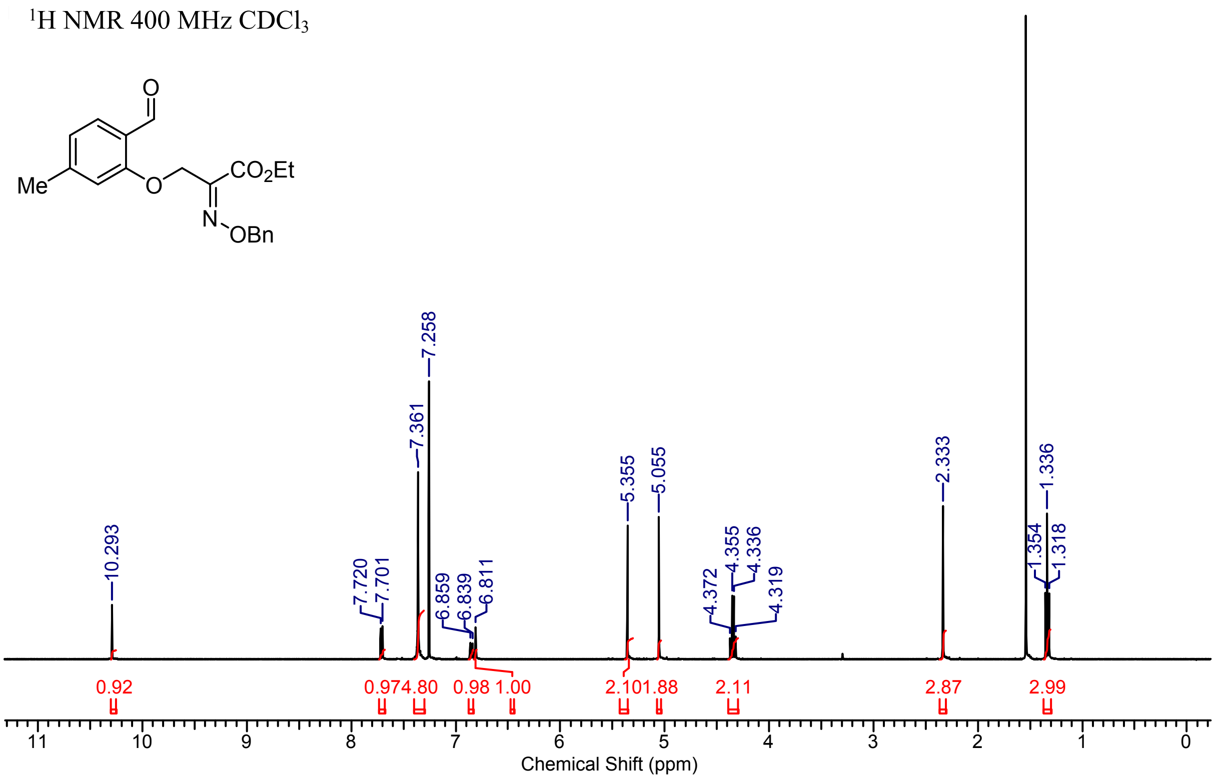


**Figure S48.** $^1$H spectrum of compound **3c**.

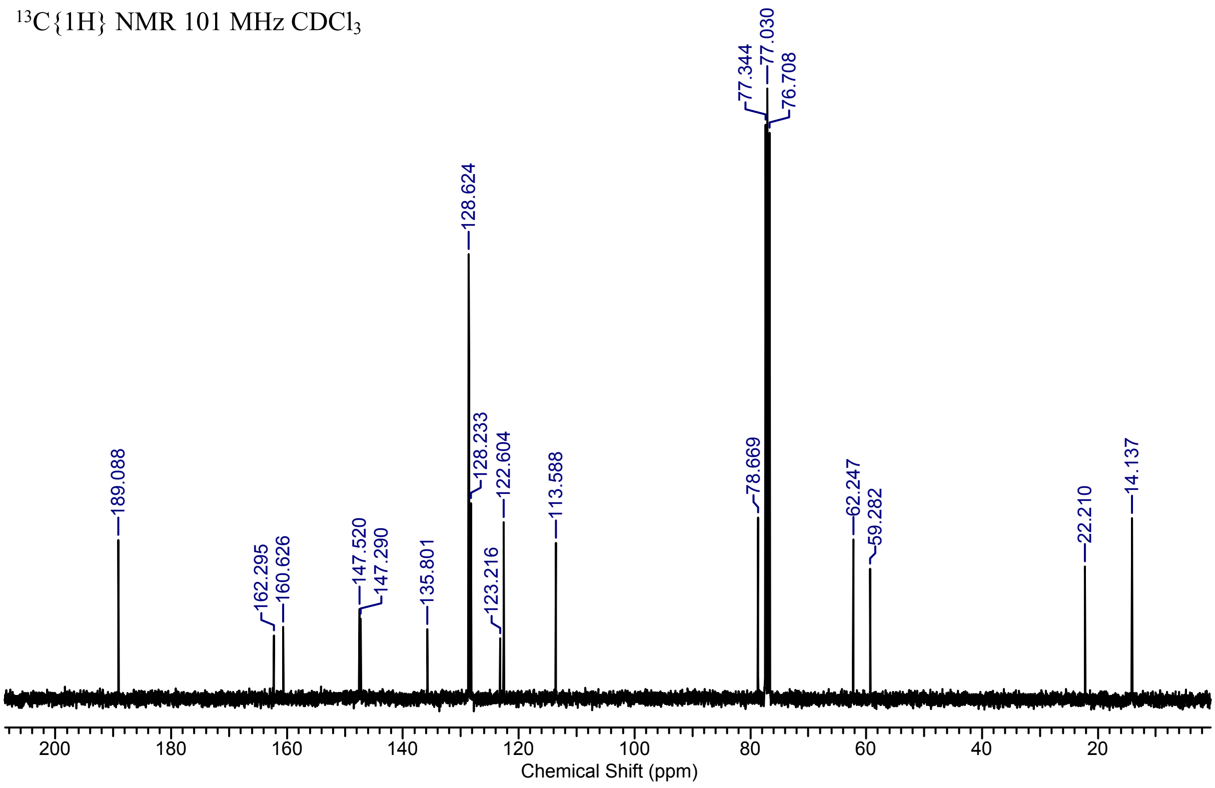


**Figure S49.** $^{13}$C spectrum of compound **3c**.

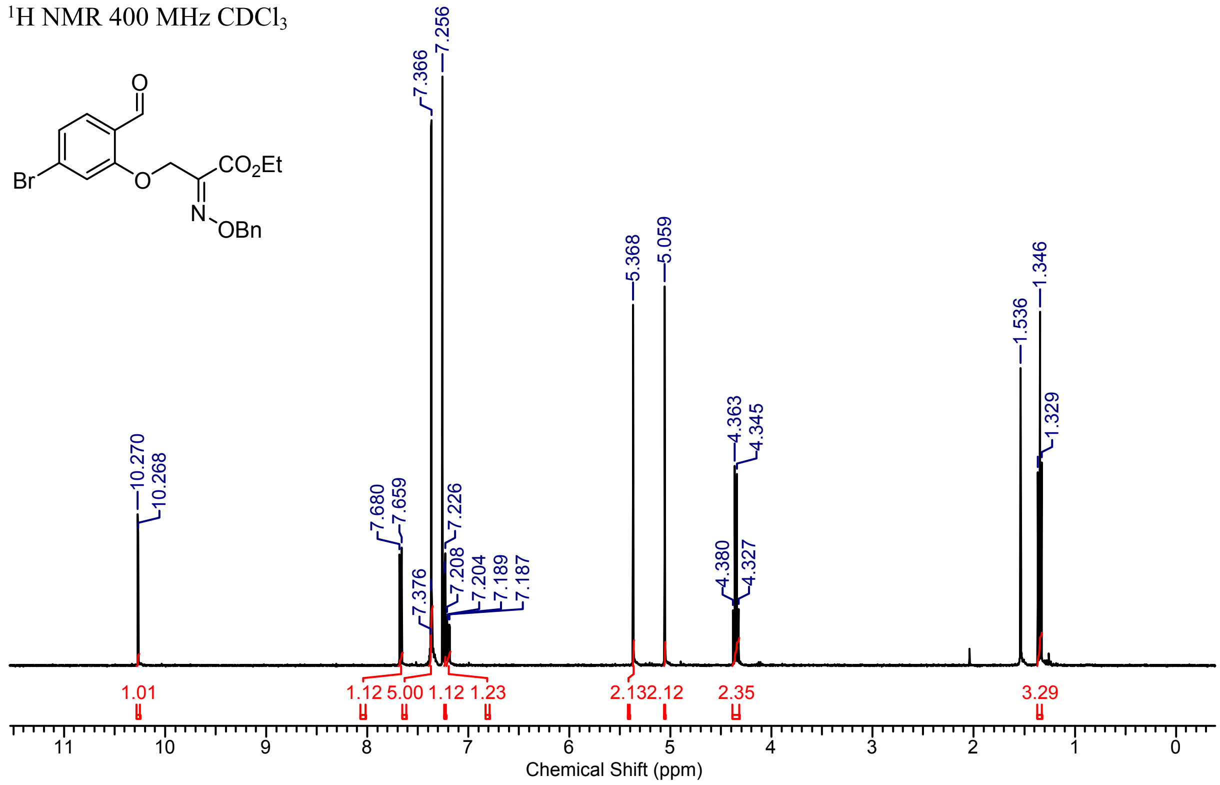


**Figure S50.** $^{1}$H spectrum of compound **3d**.

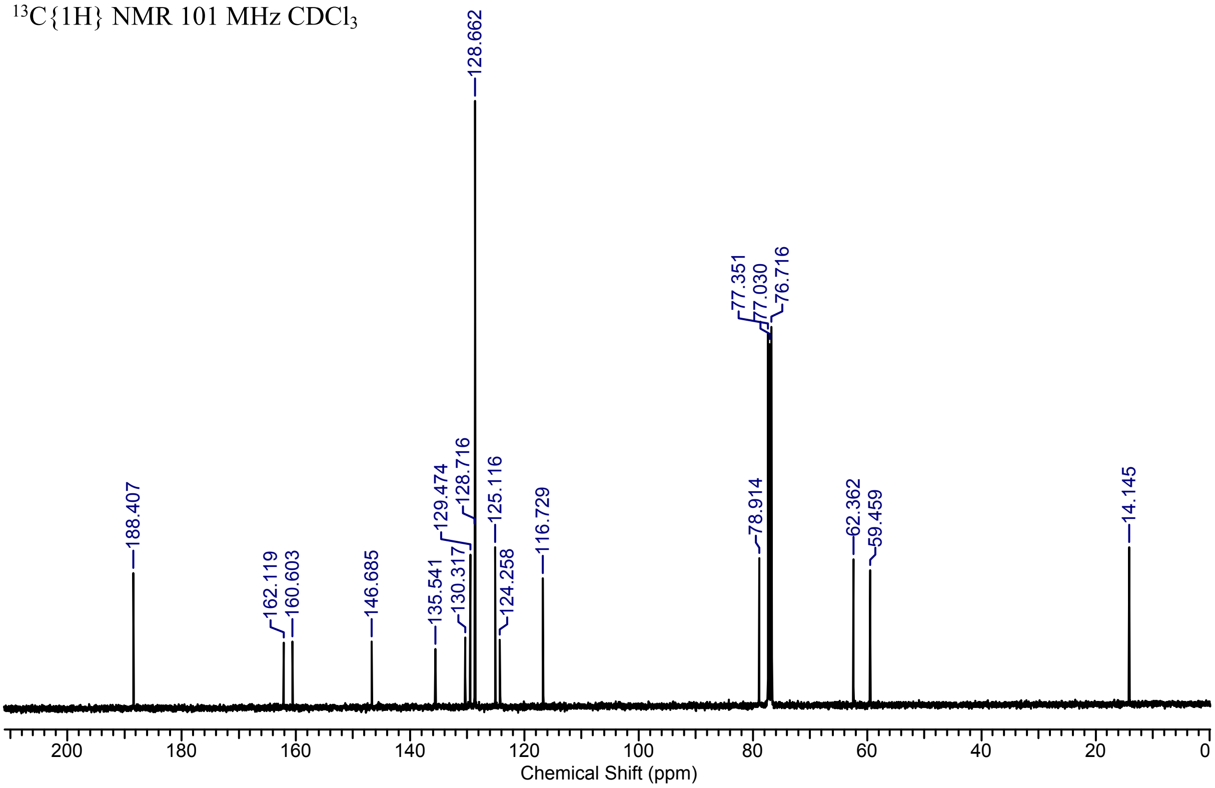


**Figure S51.** $^{13}$C spectrum of compound **3d**.

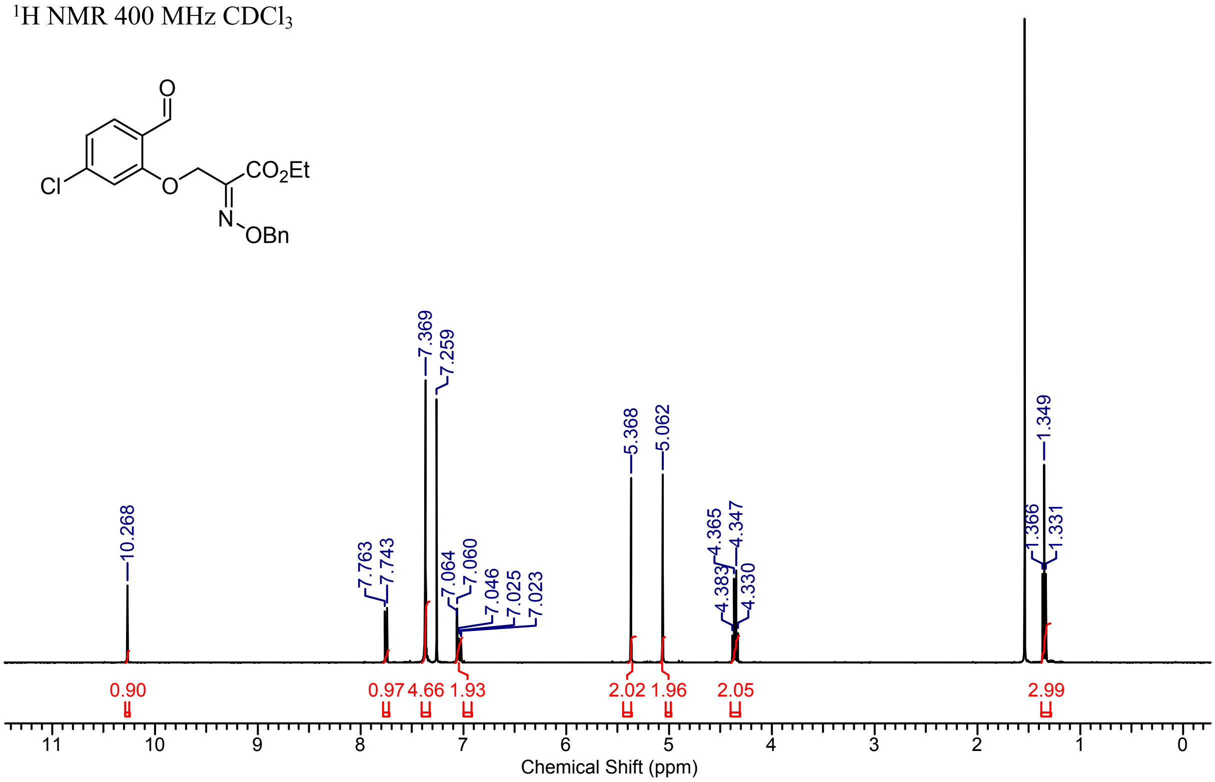


**Figure S52.** $^1$H spectrum of compound **3e**.

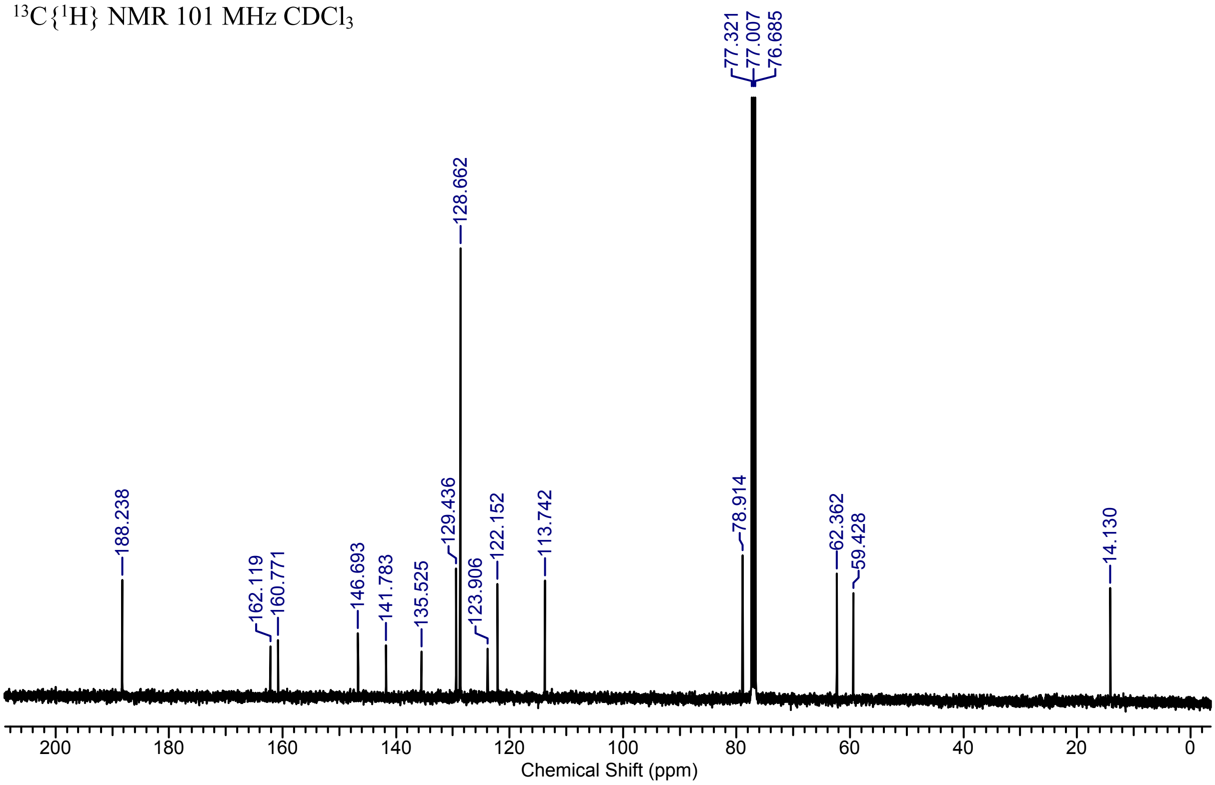


**Figure S53.** $^{13}$C spectrum of compound **3e**.

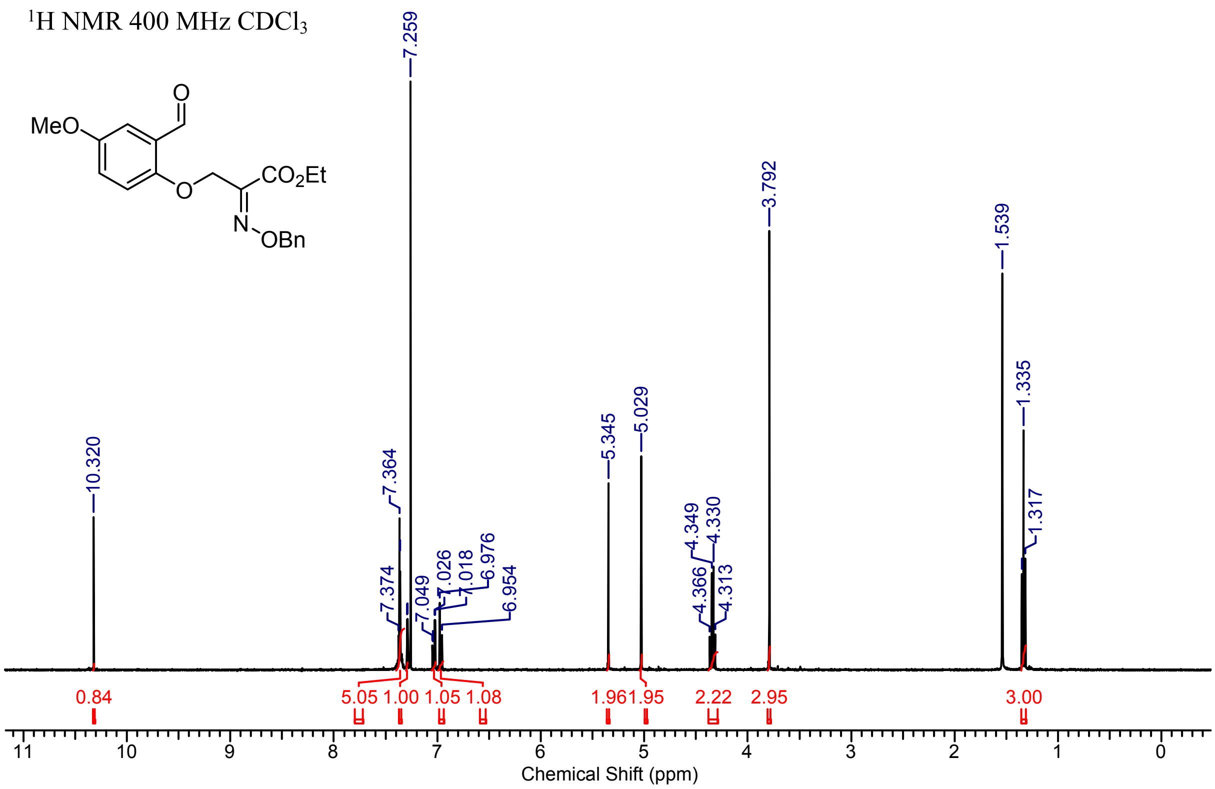


**Figure S54.** $^1$H spectrum of compound **3f**.

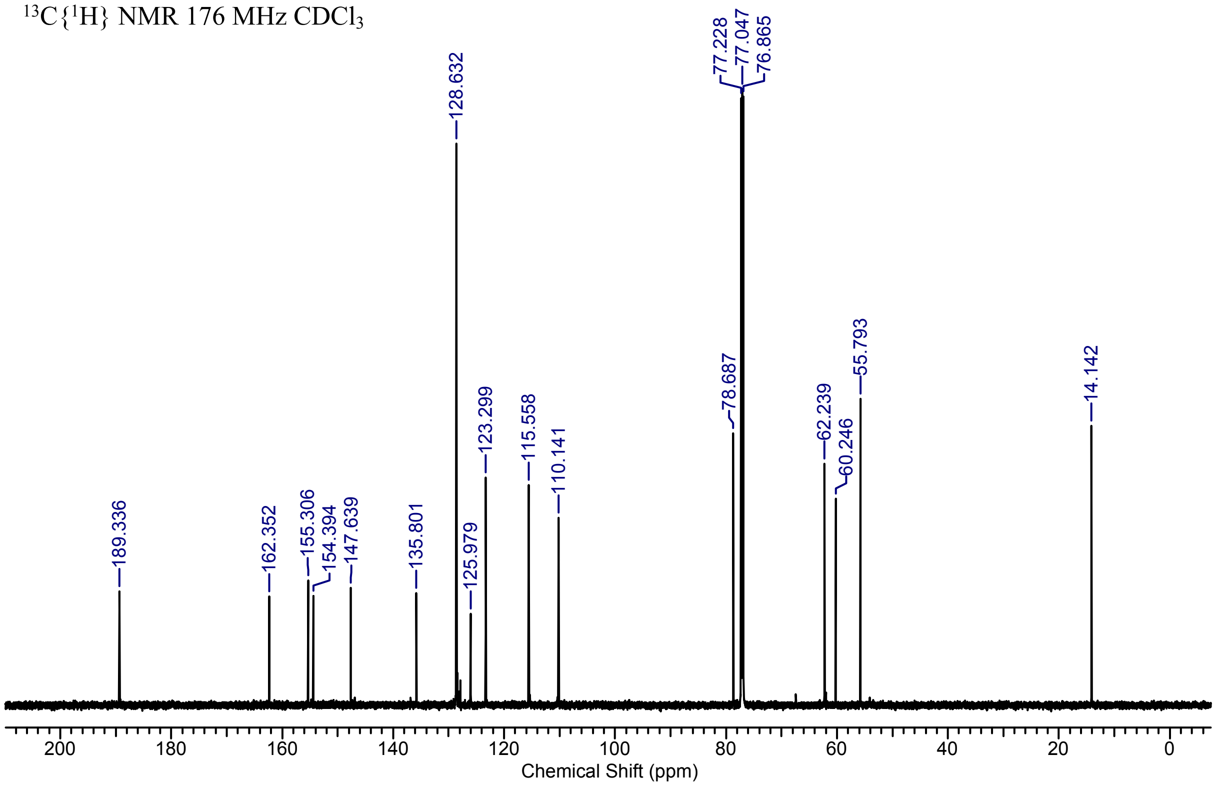


**Figure S55.** $^{13}$C spectrum of compound **3f**.

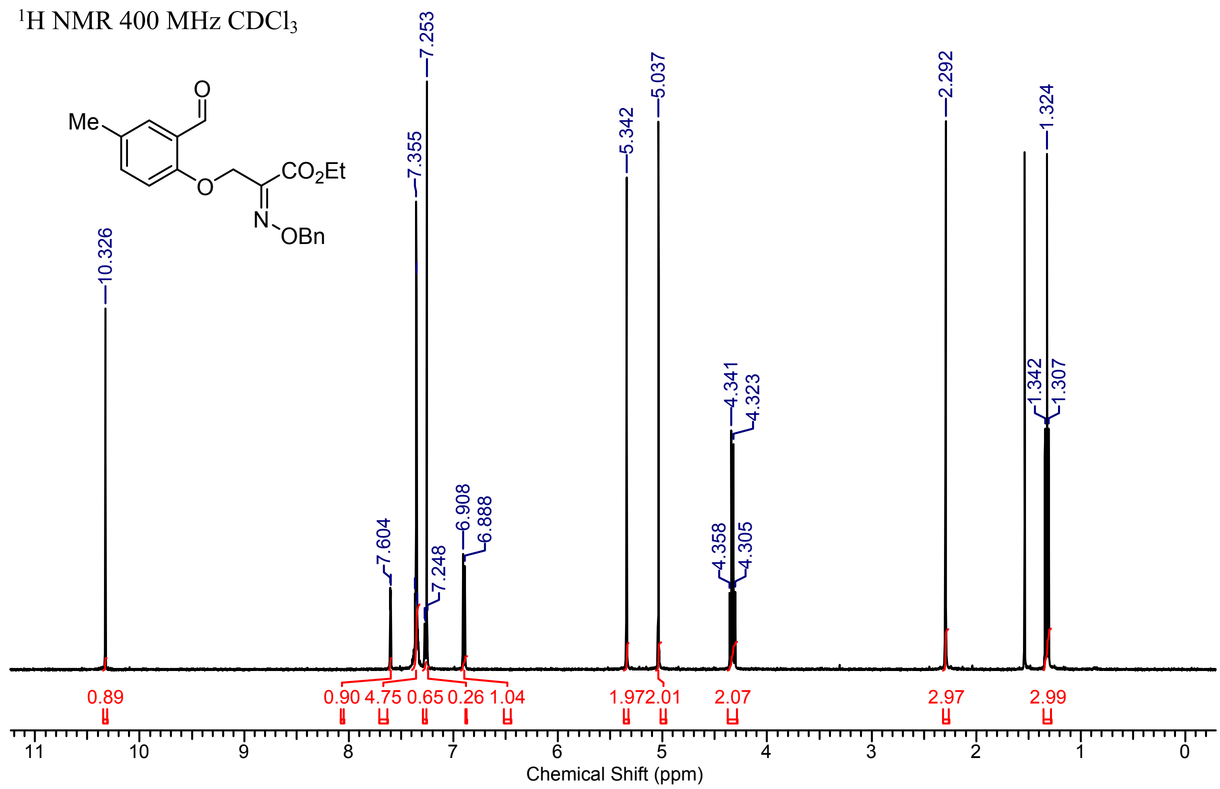


**Figure S56.** $^{1}$H spectrum of compound **3g**.

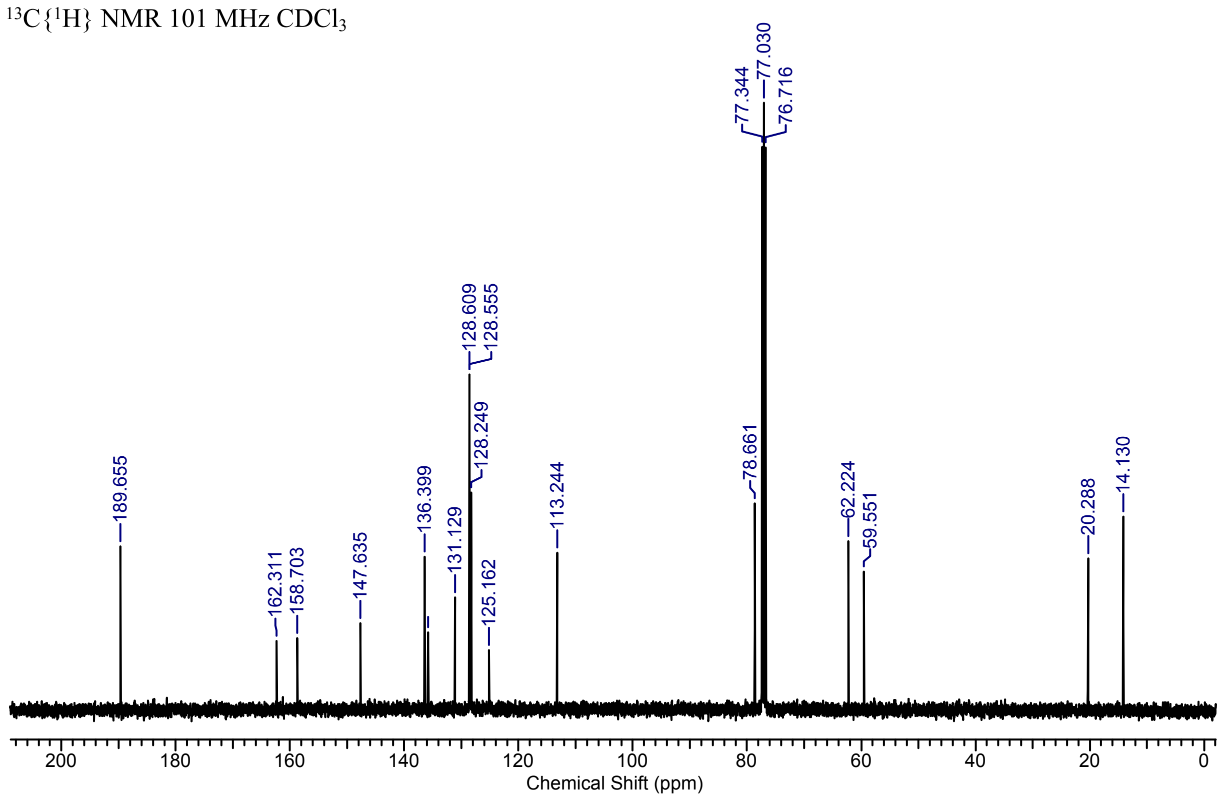


**Figure S57.** $^{13}$C spectrum of compound **3g**.

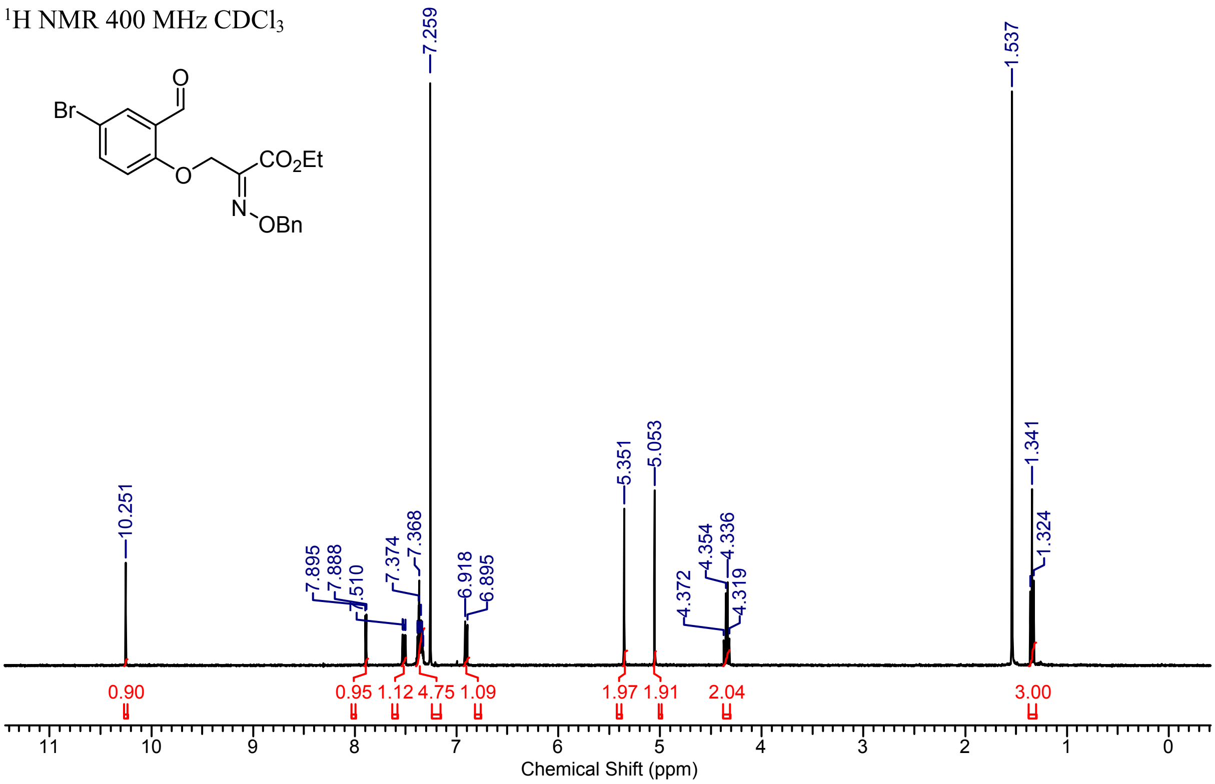


**Figure S58.** $^{1}$H spectrum of compound **3h**.

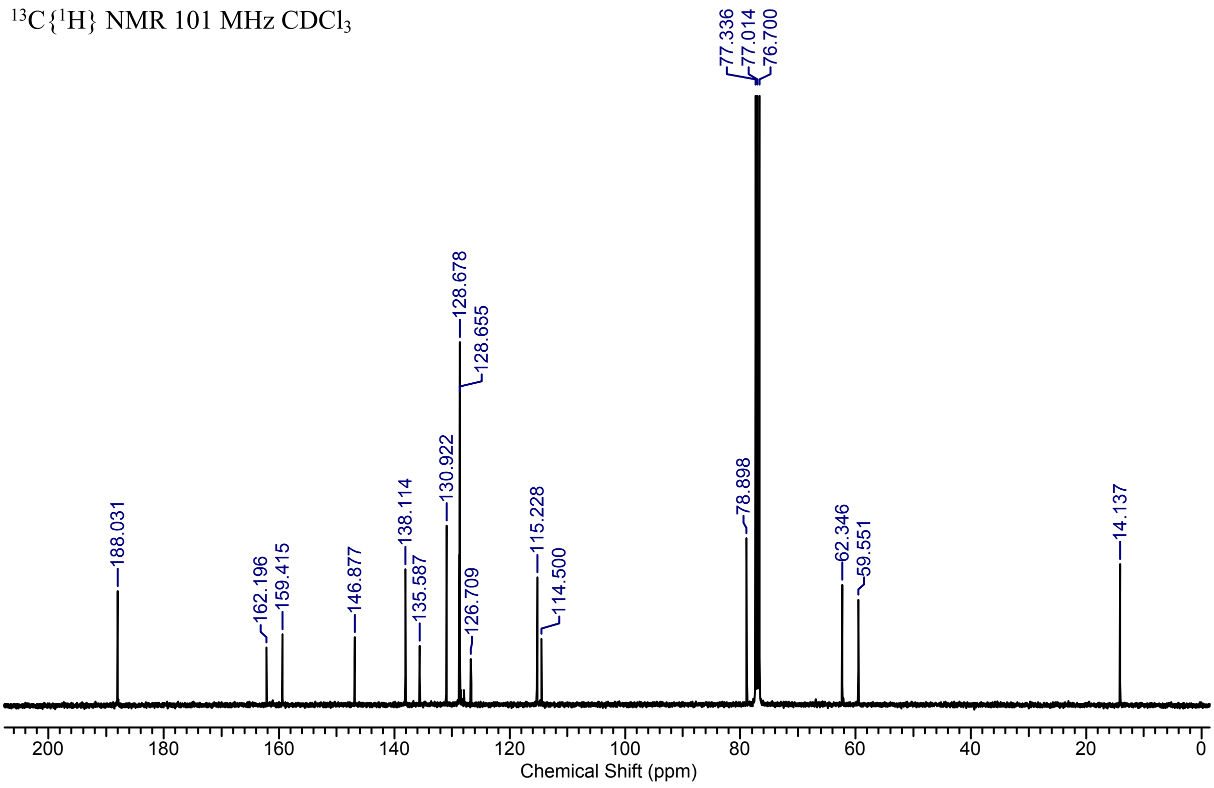


**Figure S59.** $^{13}$C spectrum of compound **3h**.

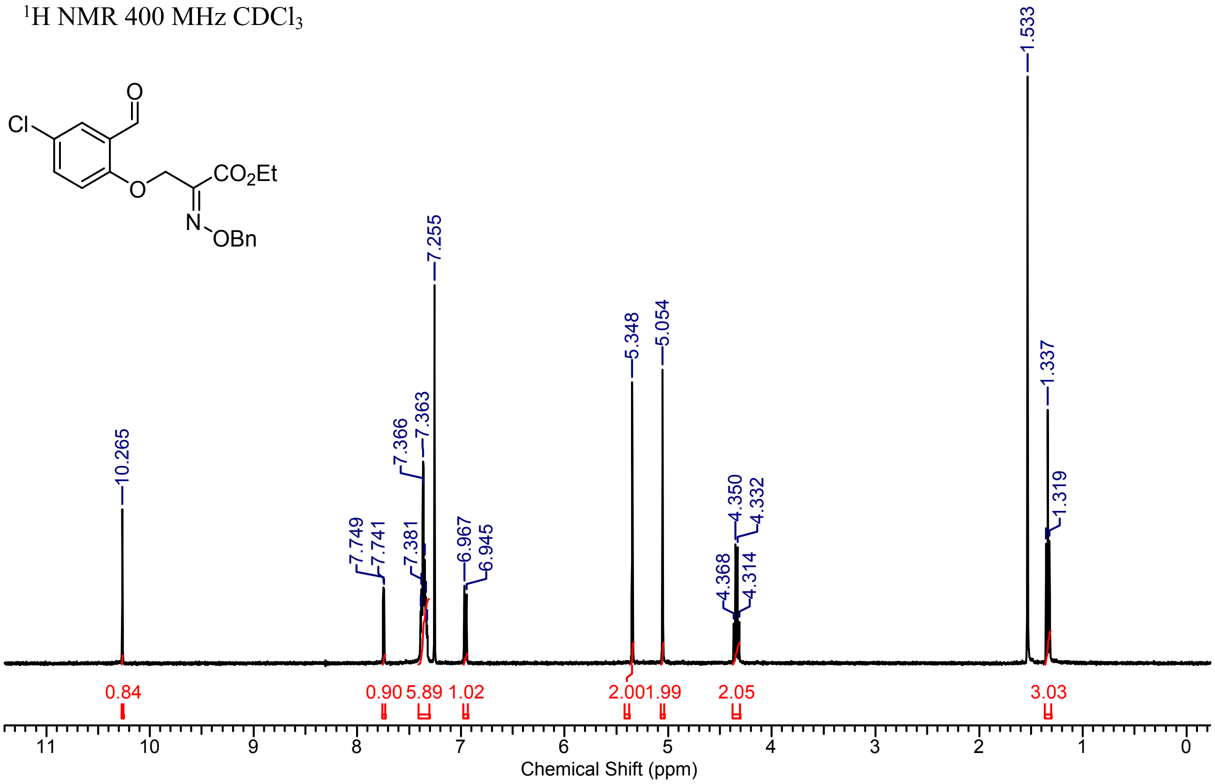


**Figure S60.** $^1H$ spectrum of compound **3i**.

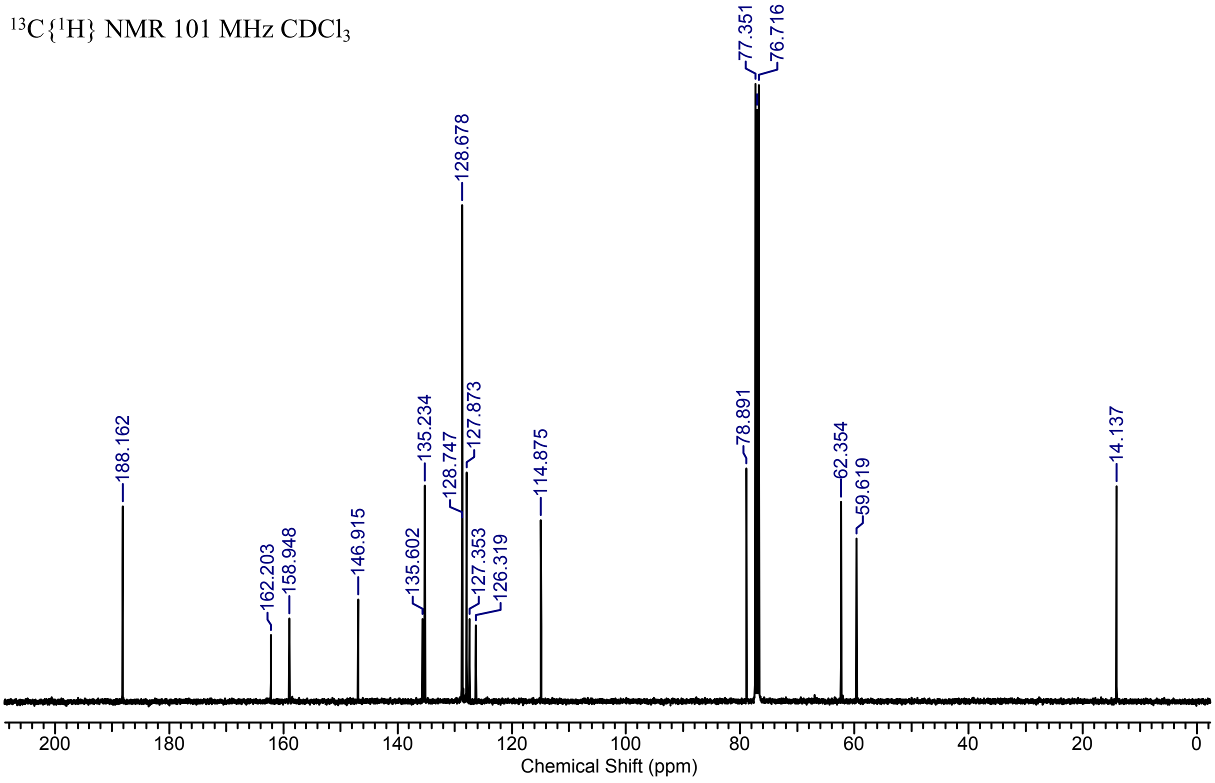


**Figure S61.** $^{13}C$ spectrum of compound **3i**.

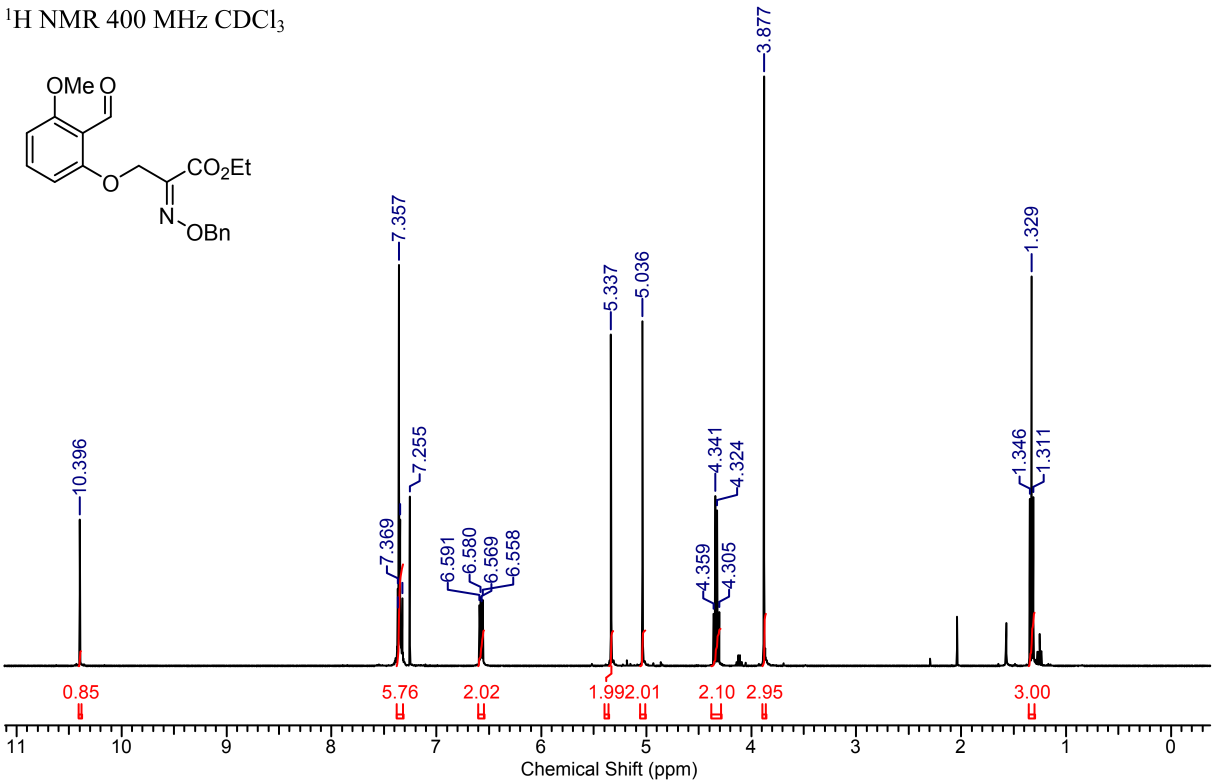


**Figure S62.** $^{1}$H spectrum of compound **3j**.

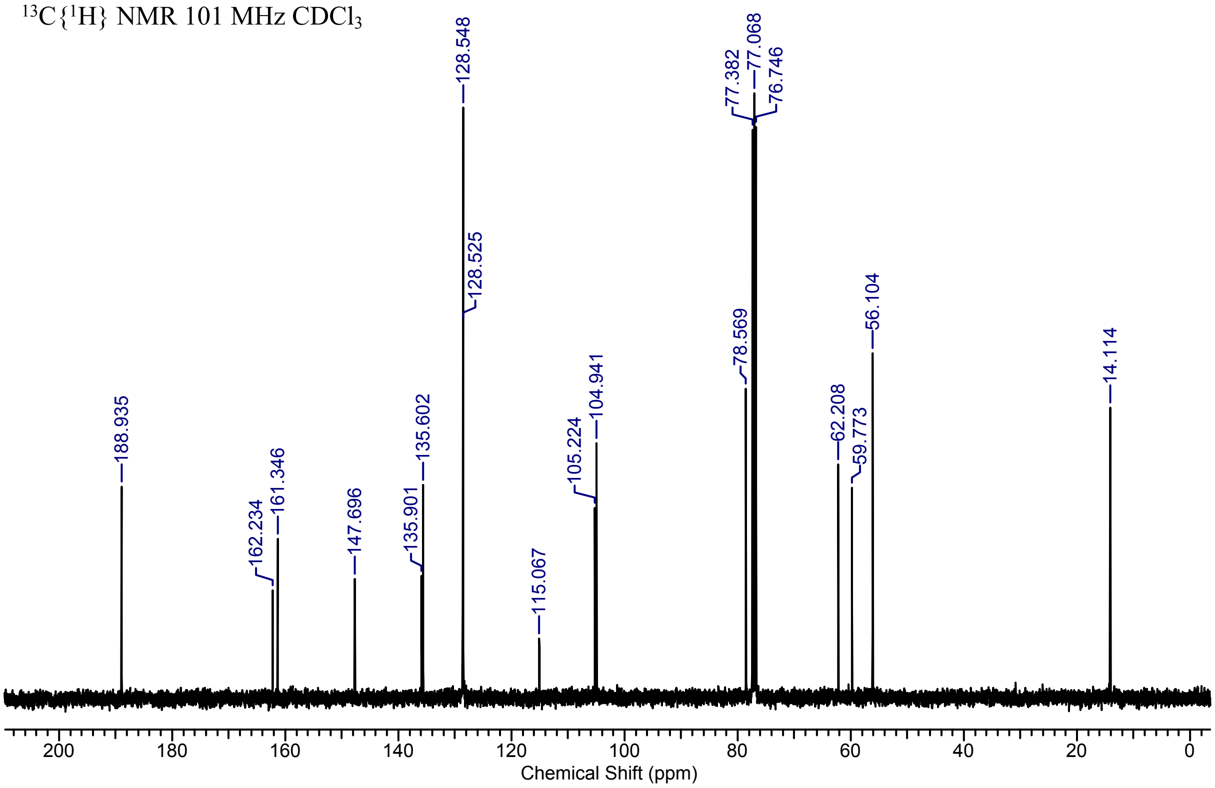


**Figure S63.** $^{13}$C spectrum of compound **3j**.

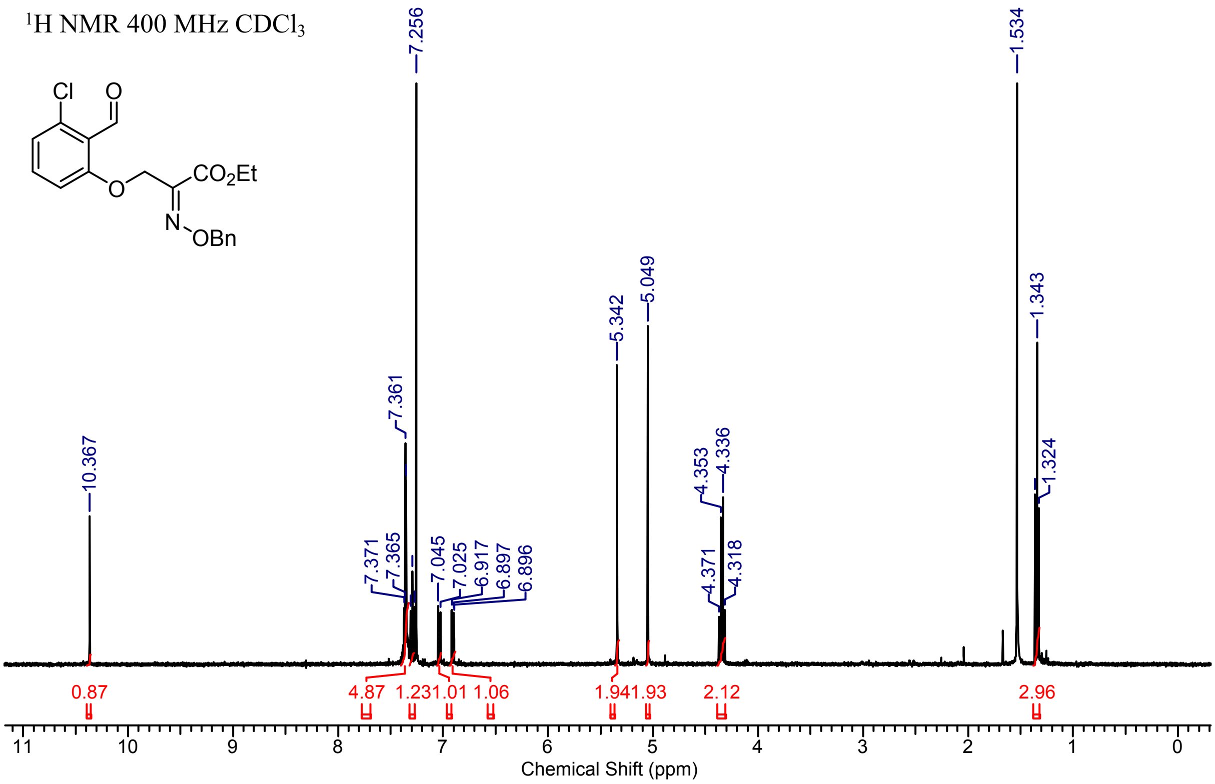


**Figure S64.** $^1$H spectrum of compound **3k**.

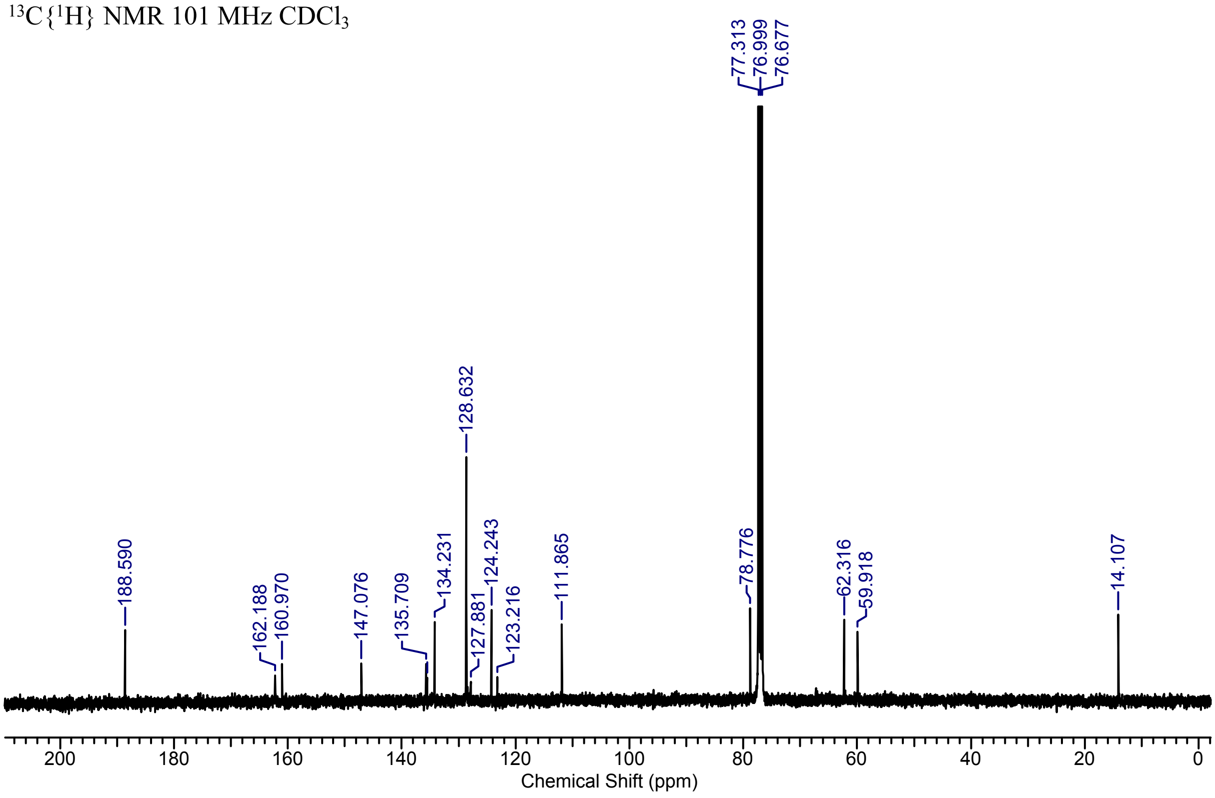


**Figure S65.** $^{13}$C spectrum of compound **3k**.

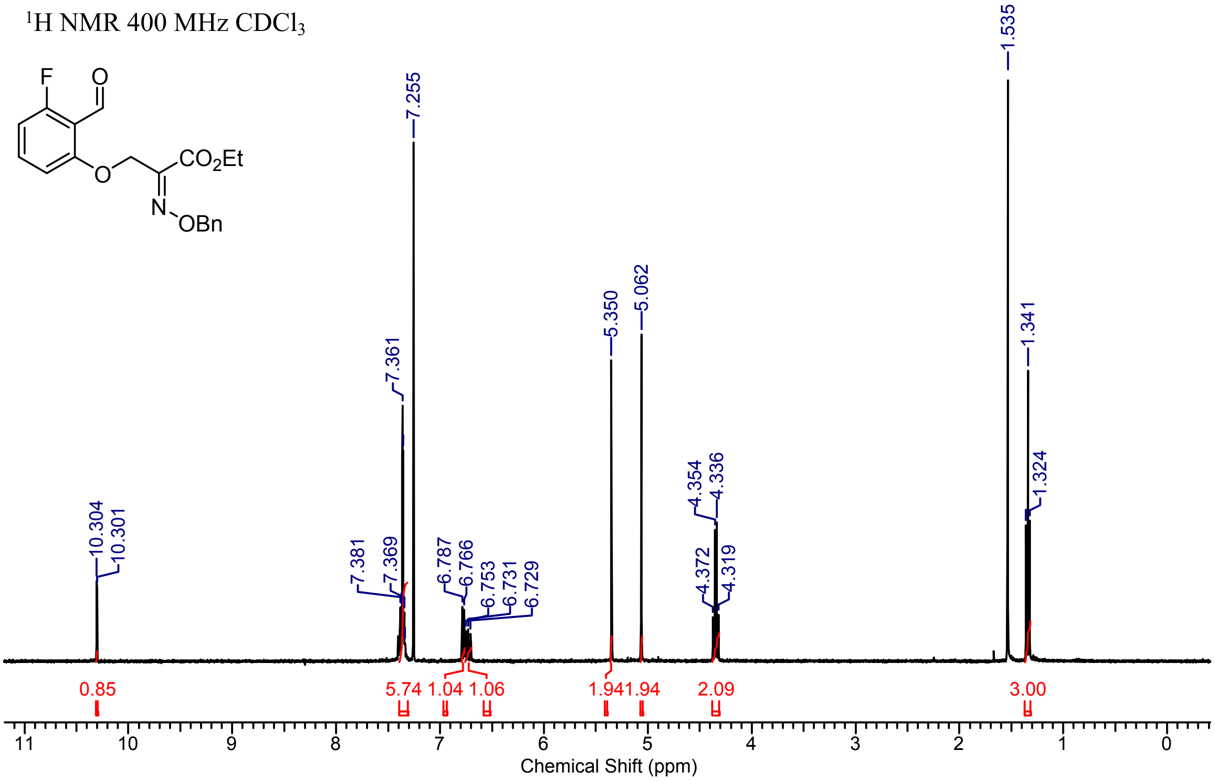


**Figure S66.** $^1$H spectrum of compound **3l**.

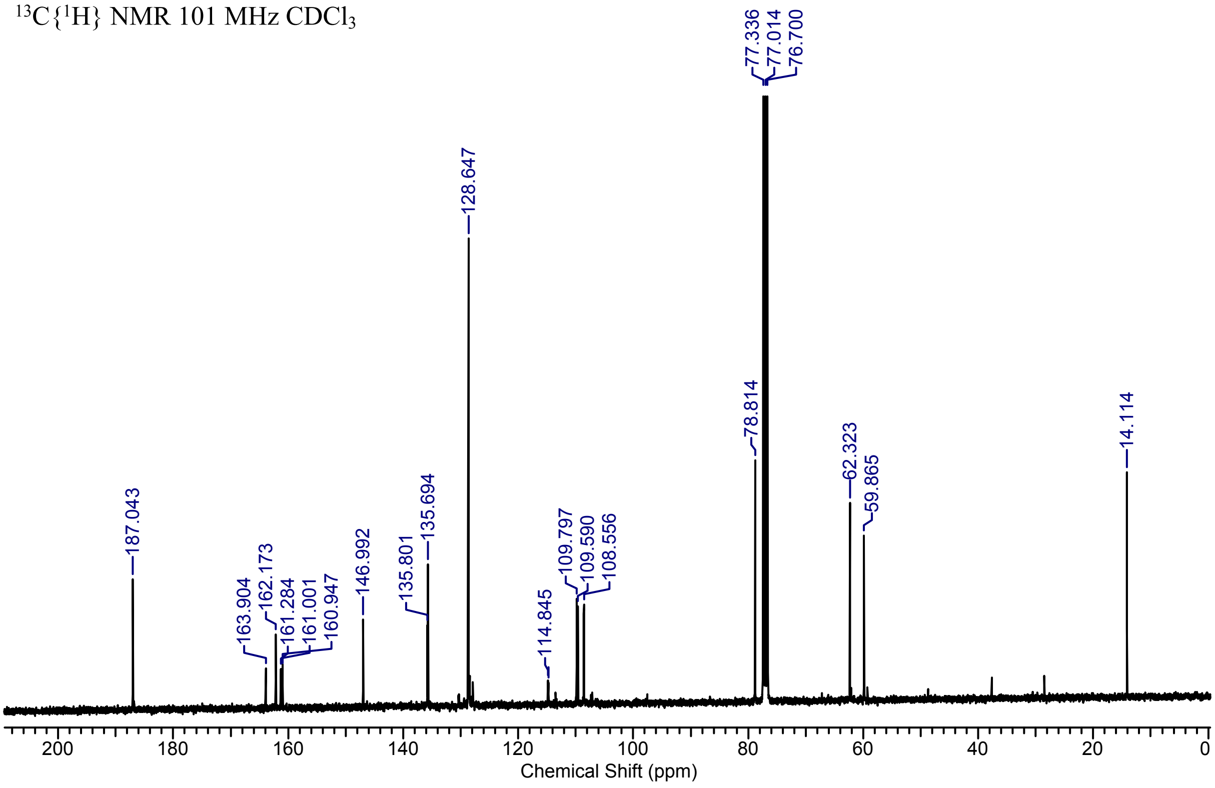


**Figure S67.** $^{13}$C spectrum of compound **3l**.

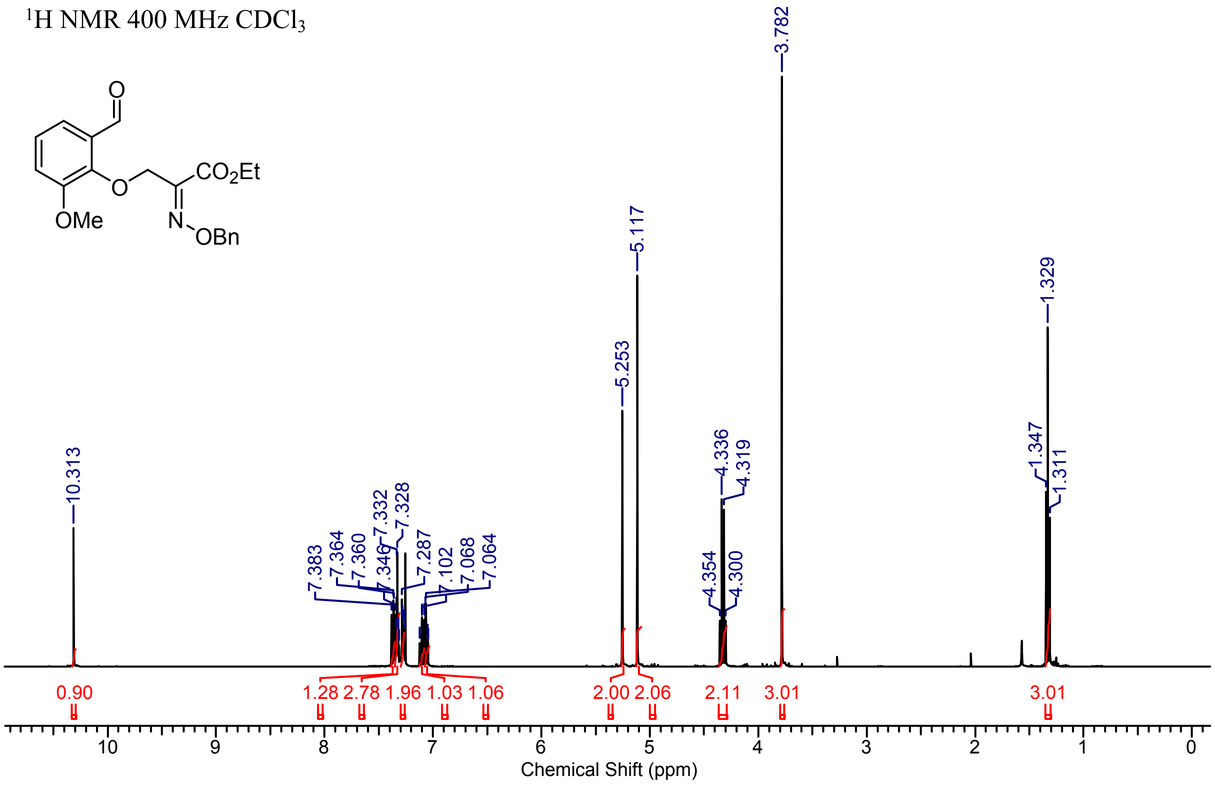


**Figure S68.** $^{1}$H spectrum of compound **3m**.

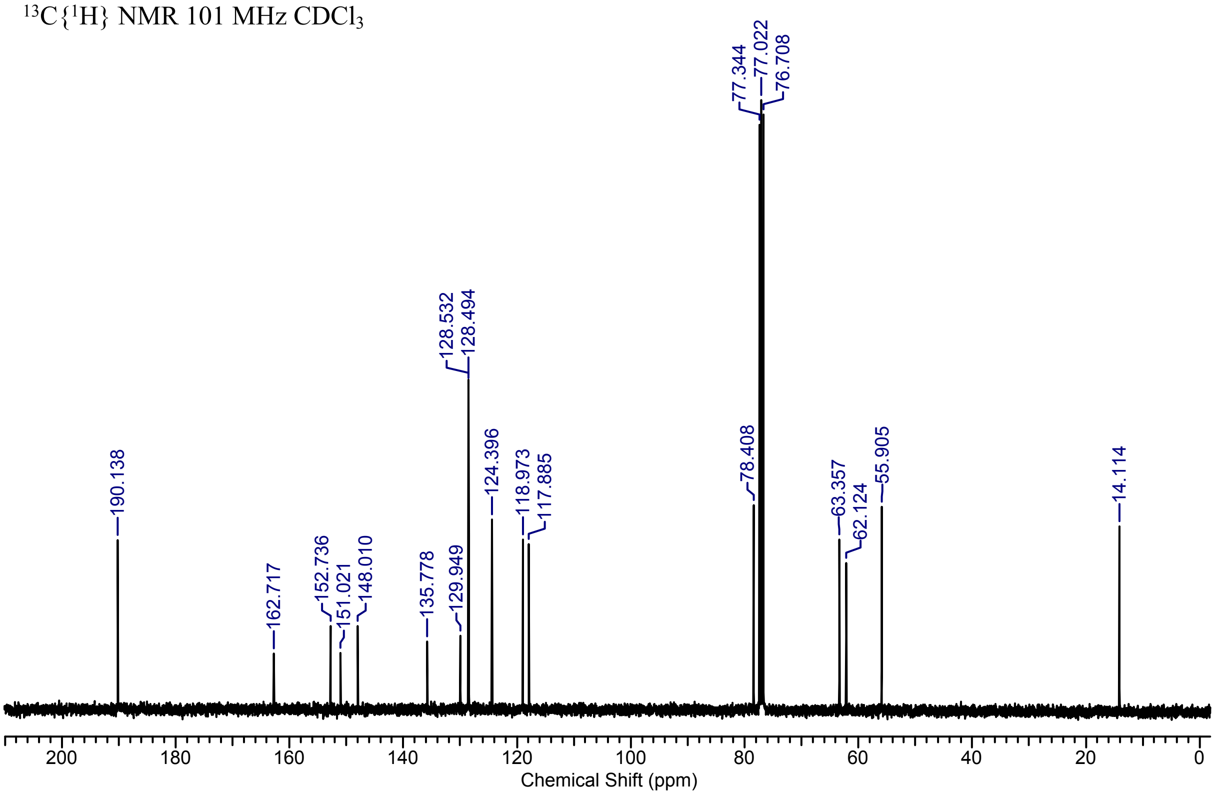


**Figure S69.** $^{13}$C spectrum of compound **3m**.

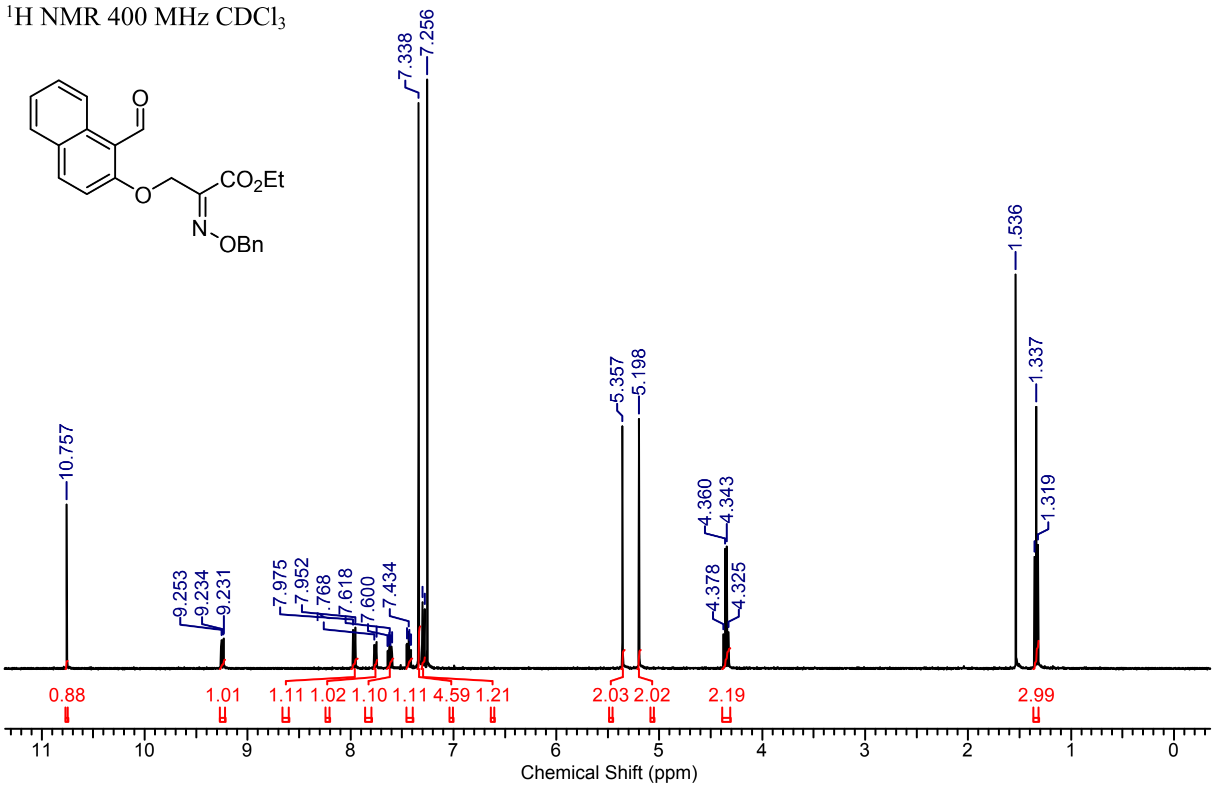


**Figure S70.** $^{1}H$ spectrum of compound **3n**.

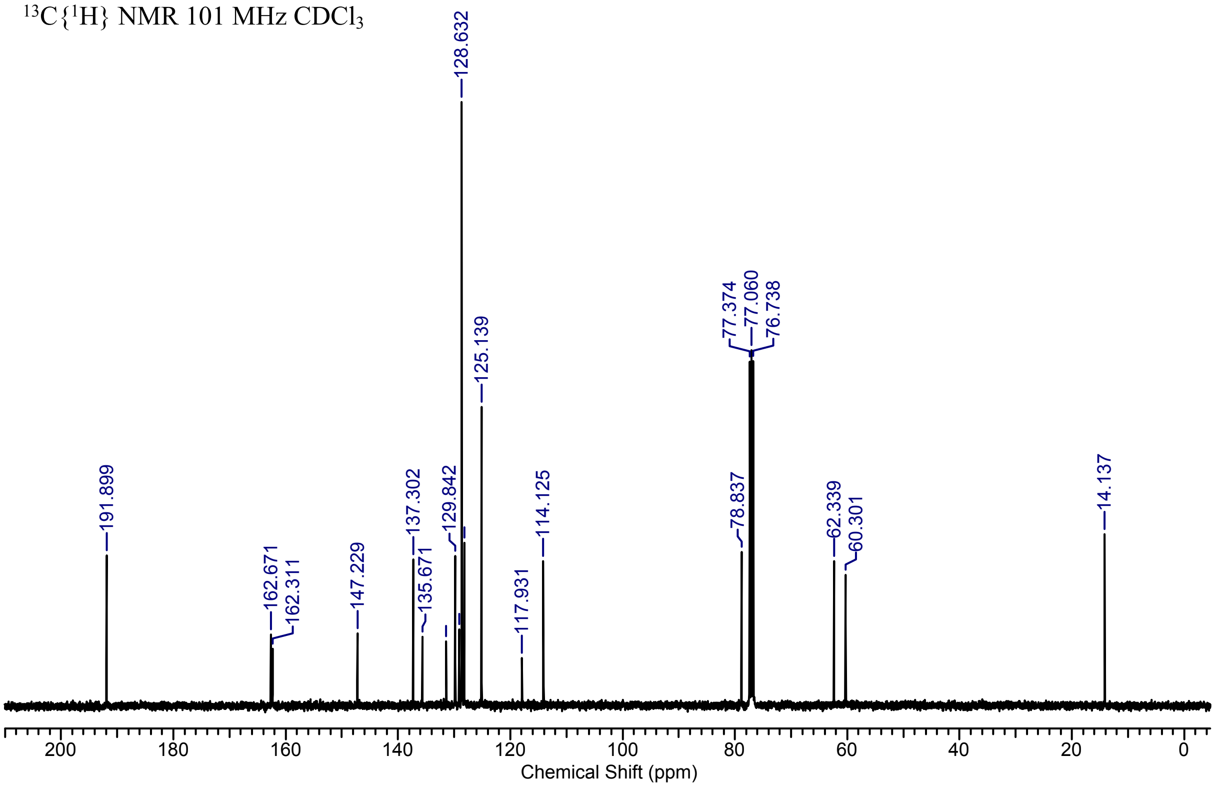


**Figure S71.** $^{13}C$ spectrum of compound **3n**.

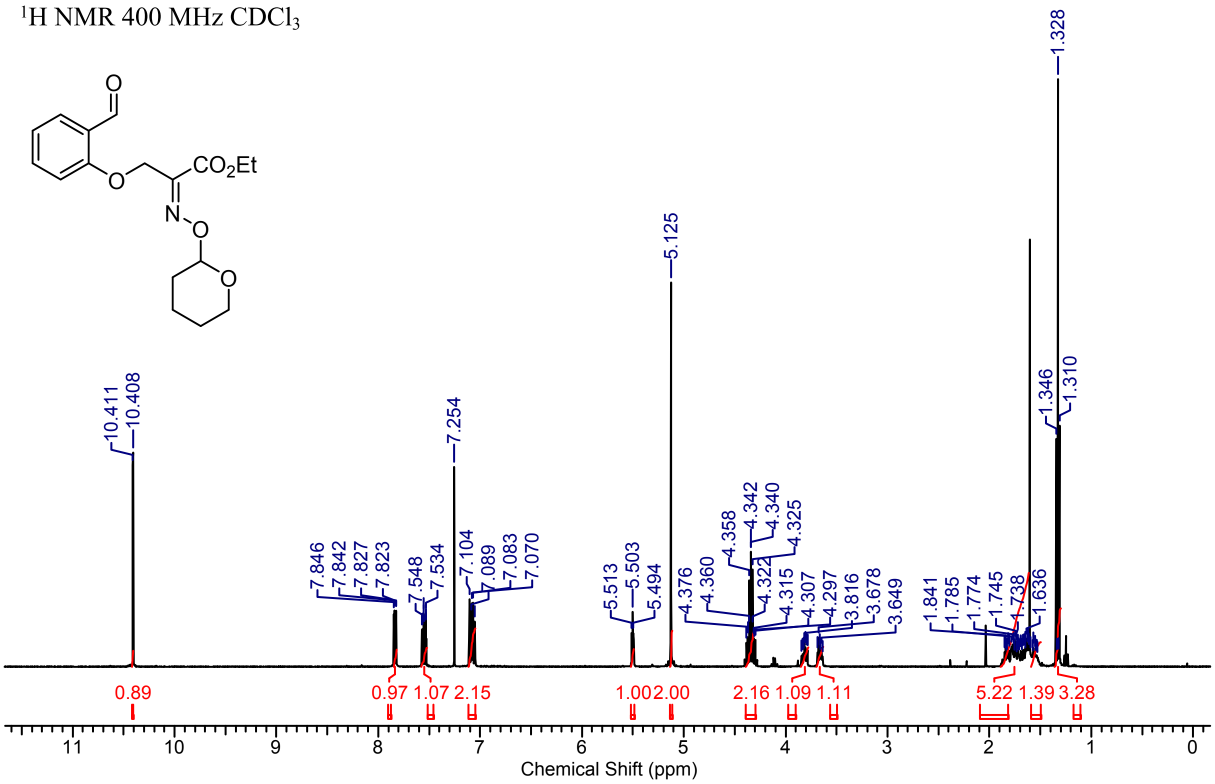


**Figure S72.** $^{1}H$ spectrum of compound **3o**.

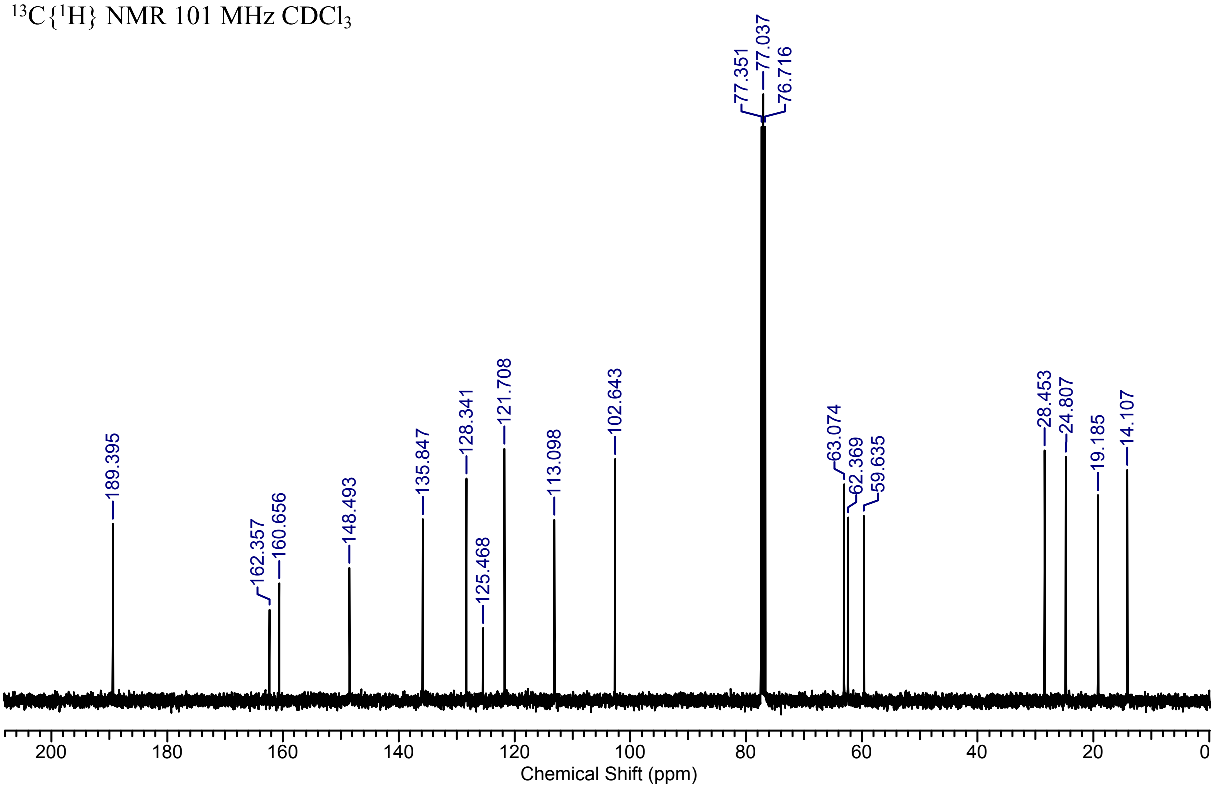


**Figure S73.** $^{13}C$ spectrum of compound **3o**.

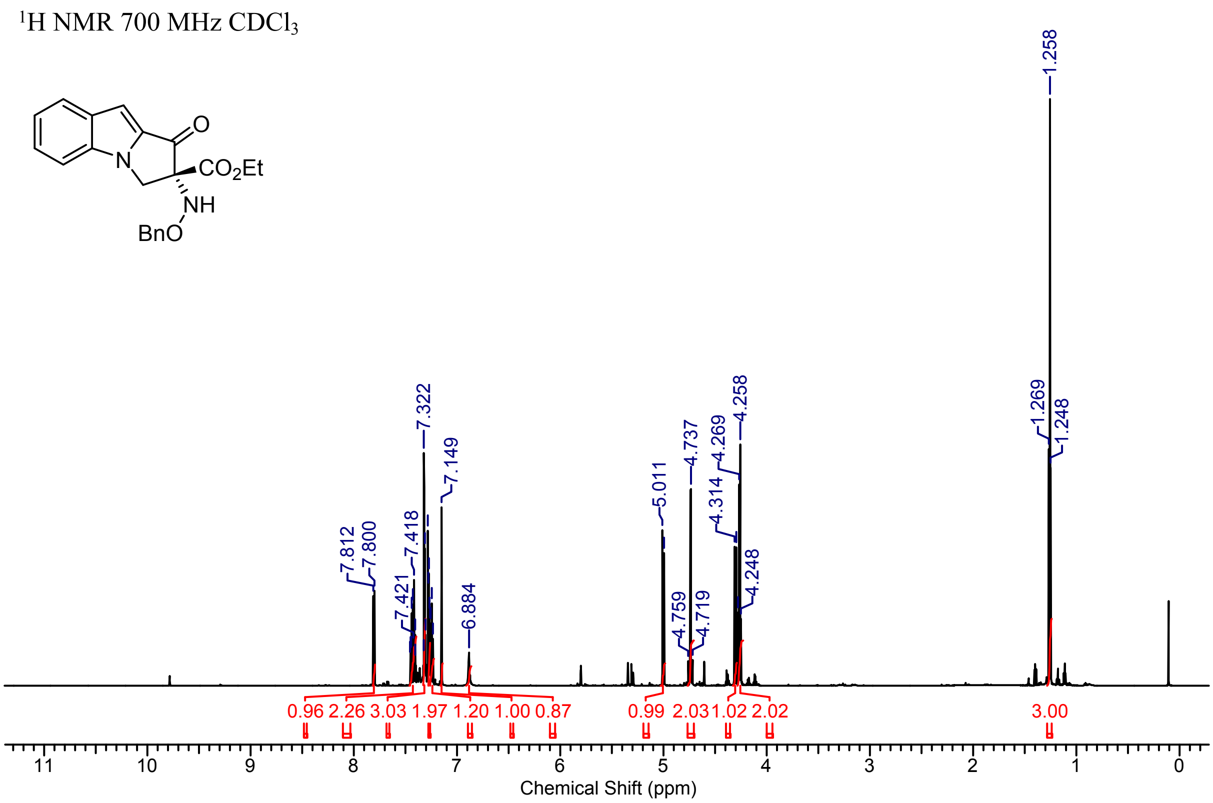


**Figure S74.** $^1H$ spectrum of compound **2a**.

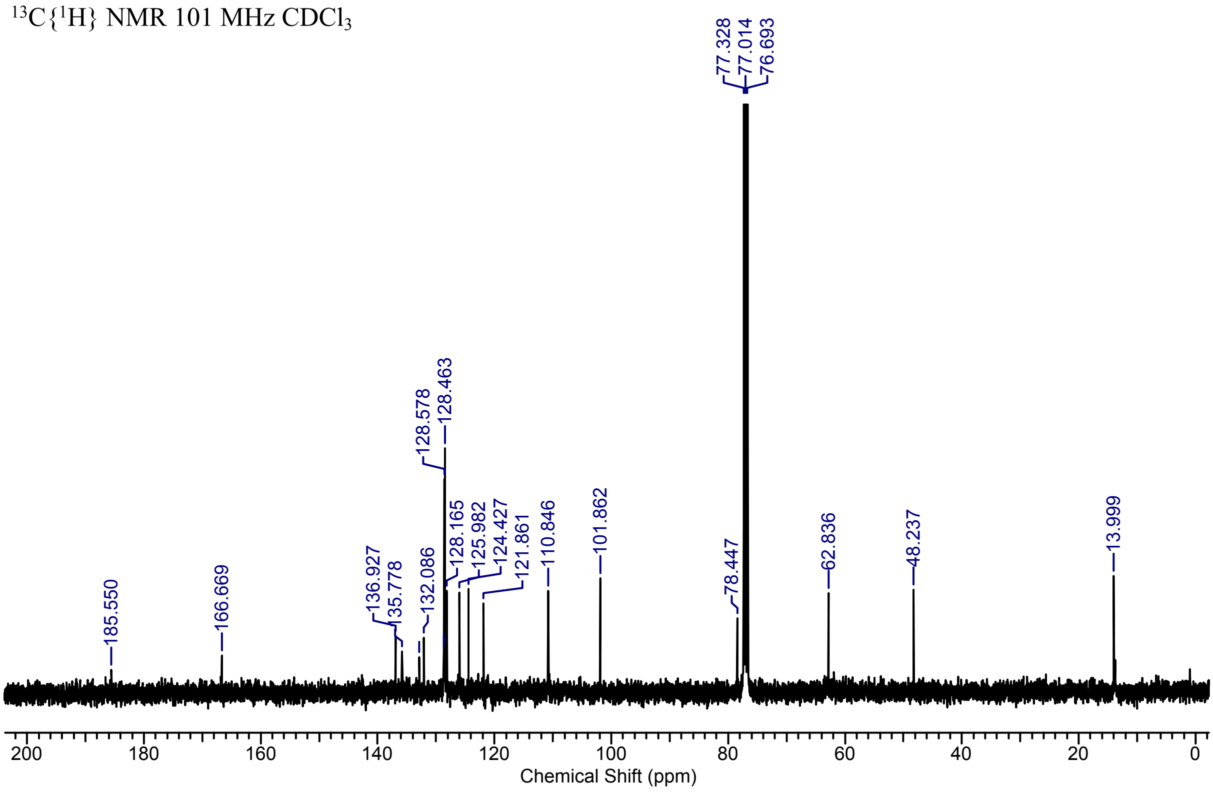


**Figure S75.** $^{13}C$ spectrum of compound **2a**.

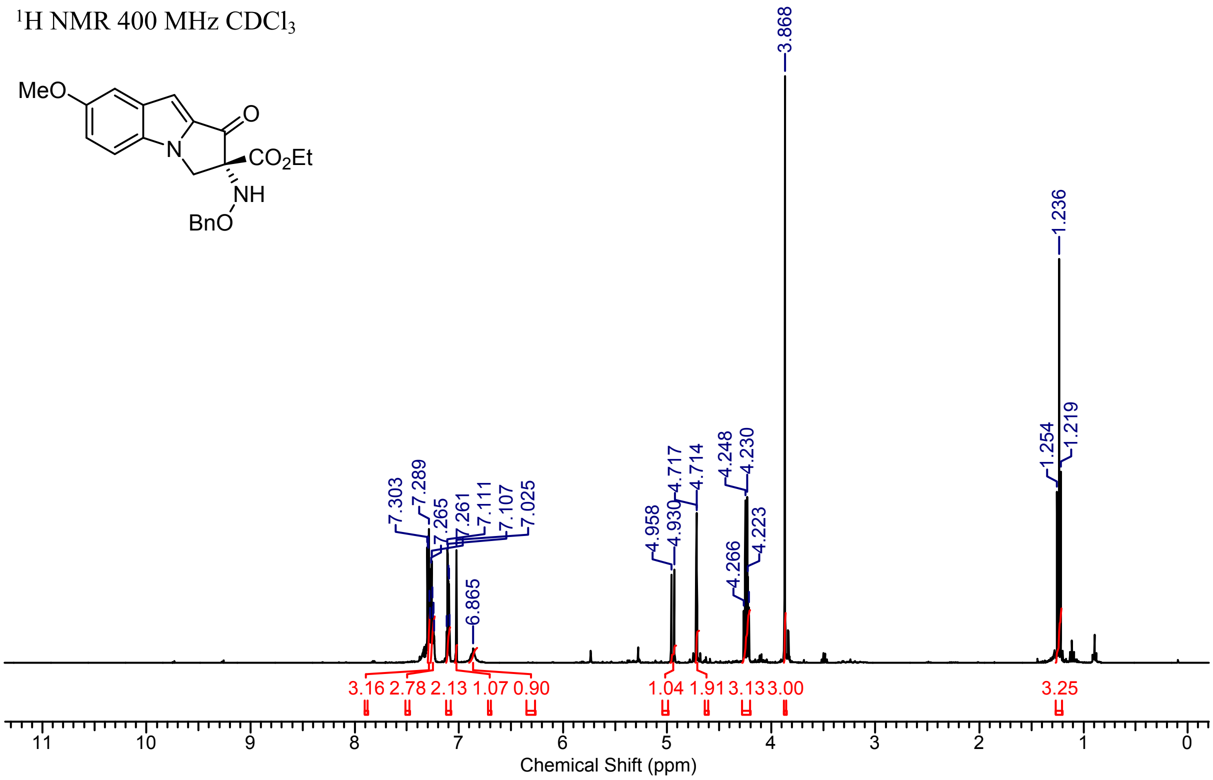


**Figure S76.** $^{1}H$ spectrum of compound **2b**.

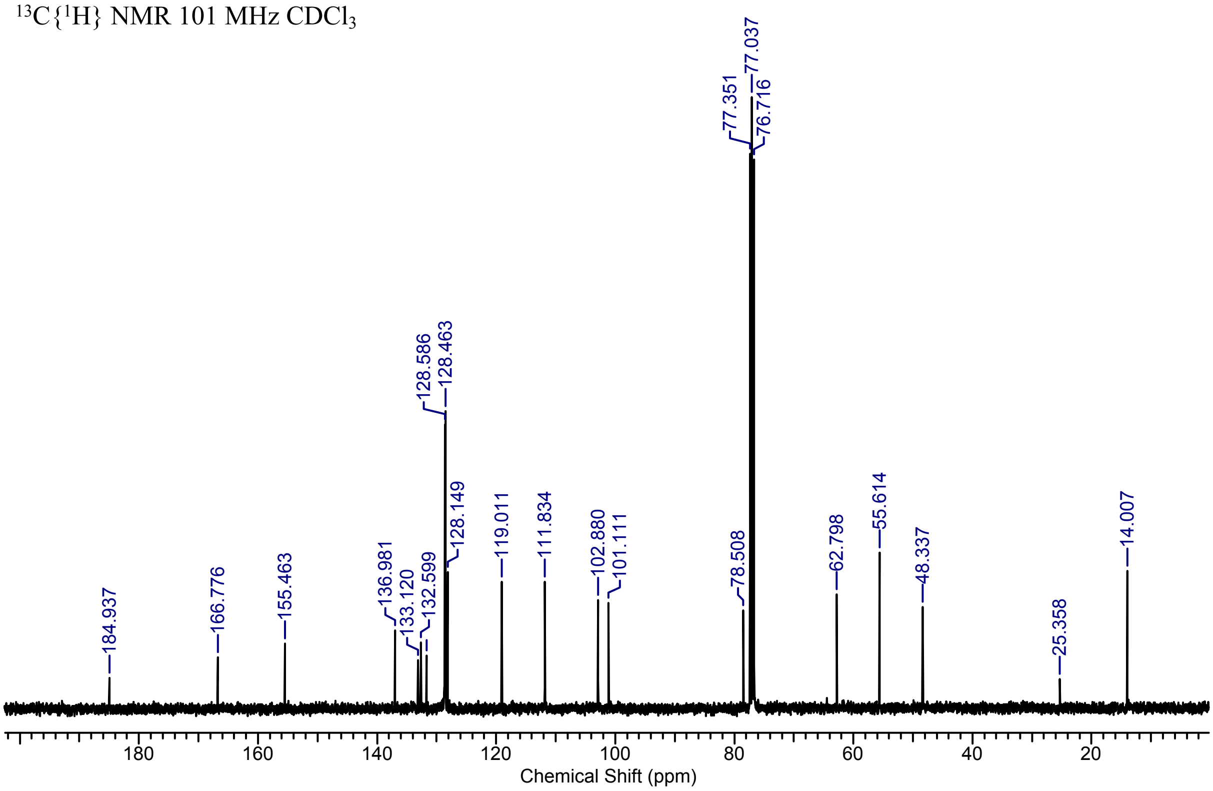


**Figure S77.** $^{13}C$ spectrum of compound **2b**.

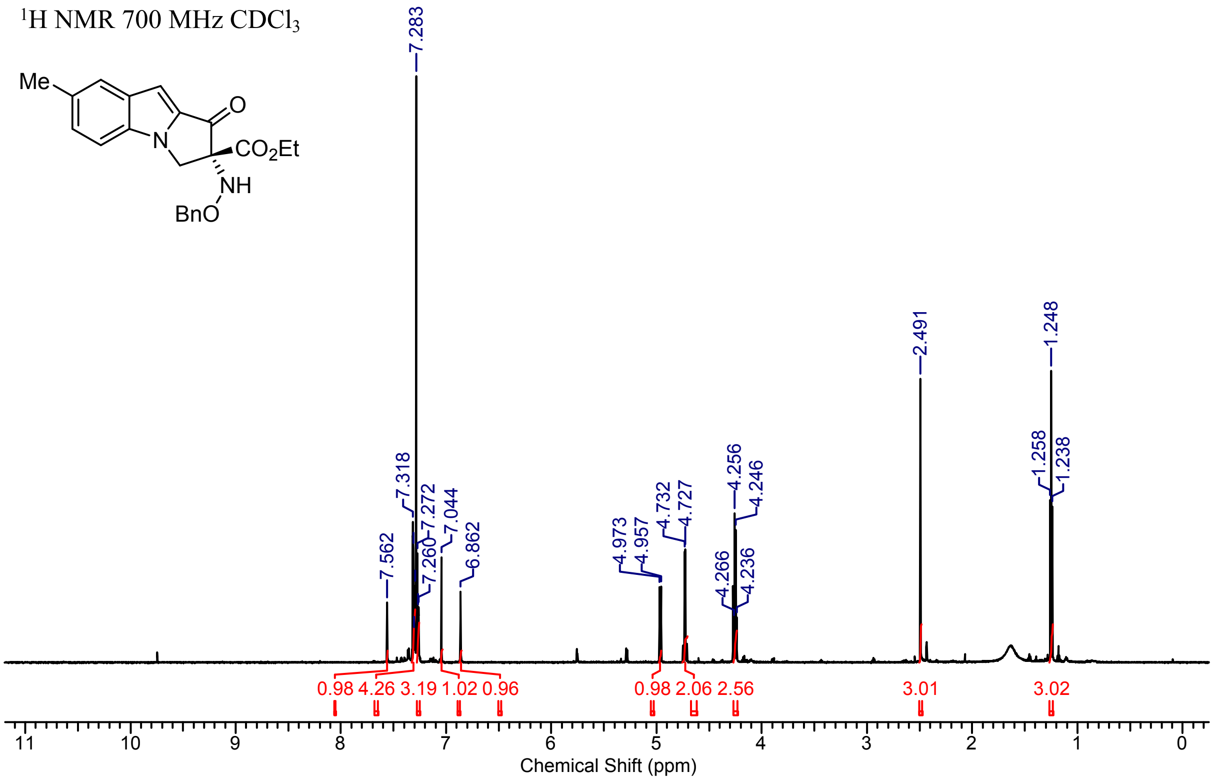


**Figure S78.** $^1$H spectrum of compound **2c**.

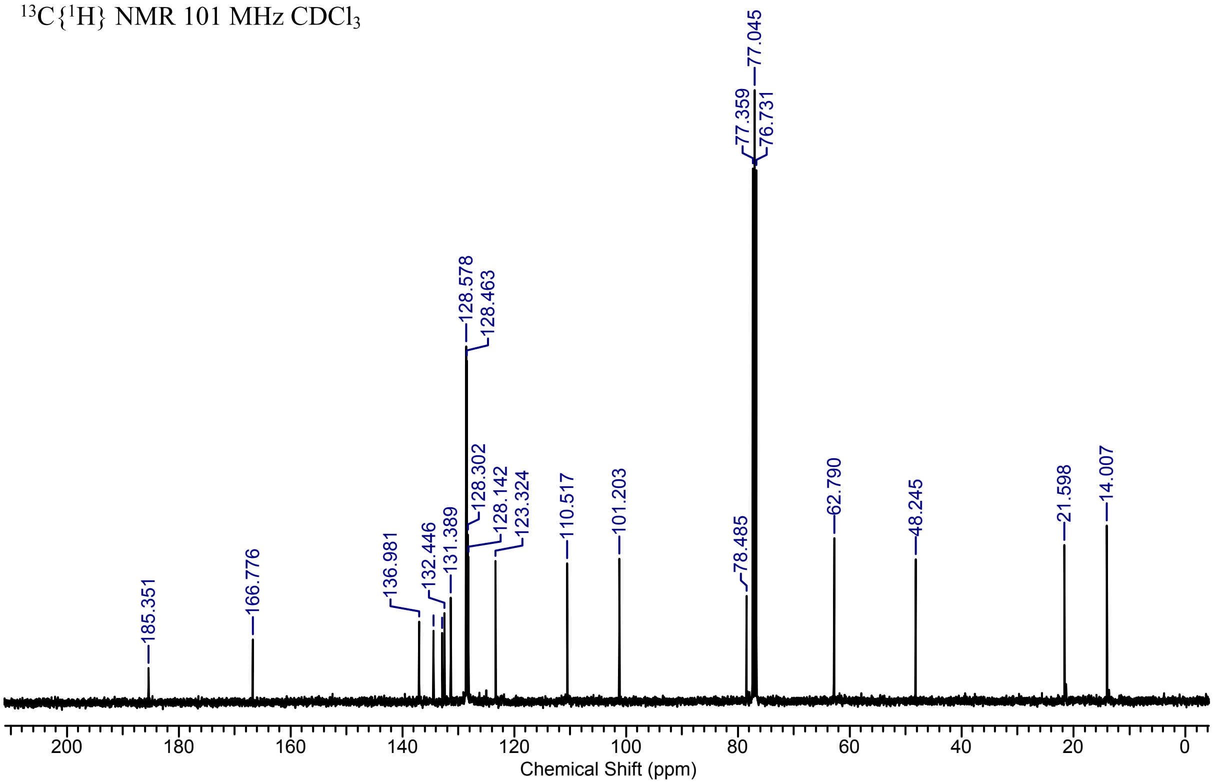


**Figure S79.** $^{13}$C spectrum of compound **2c**.

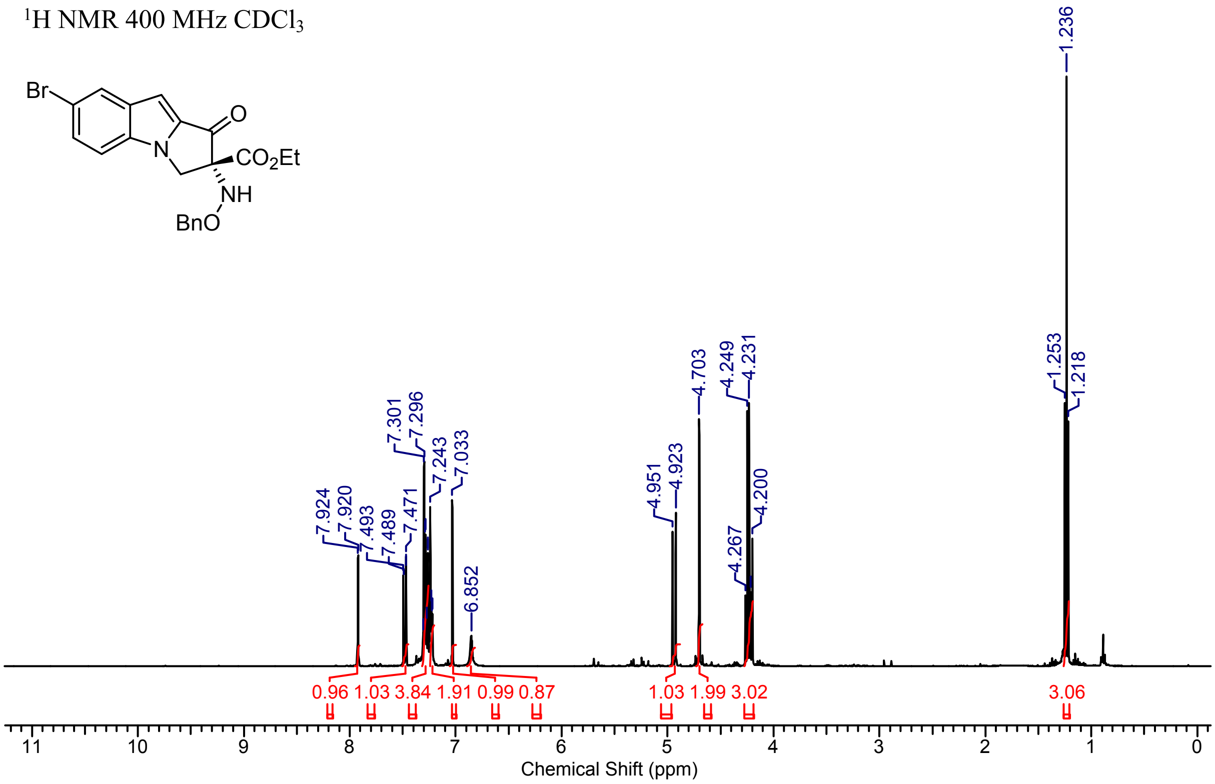


**Figure S80.** $^1$H spectrum of compound **2d**.

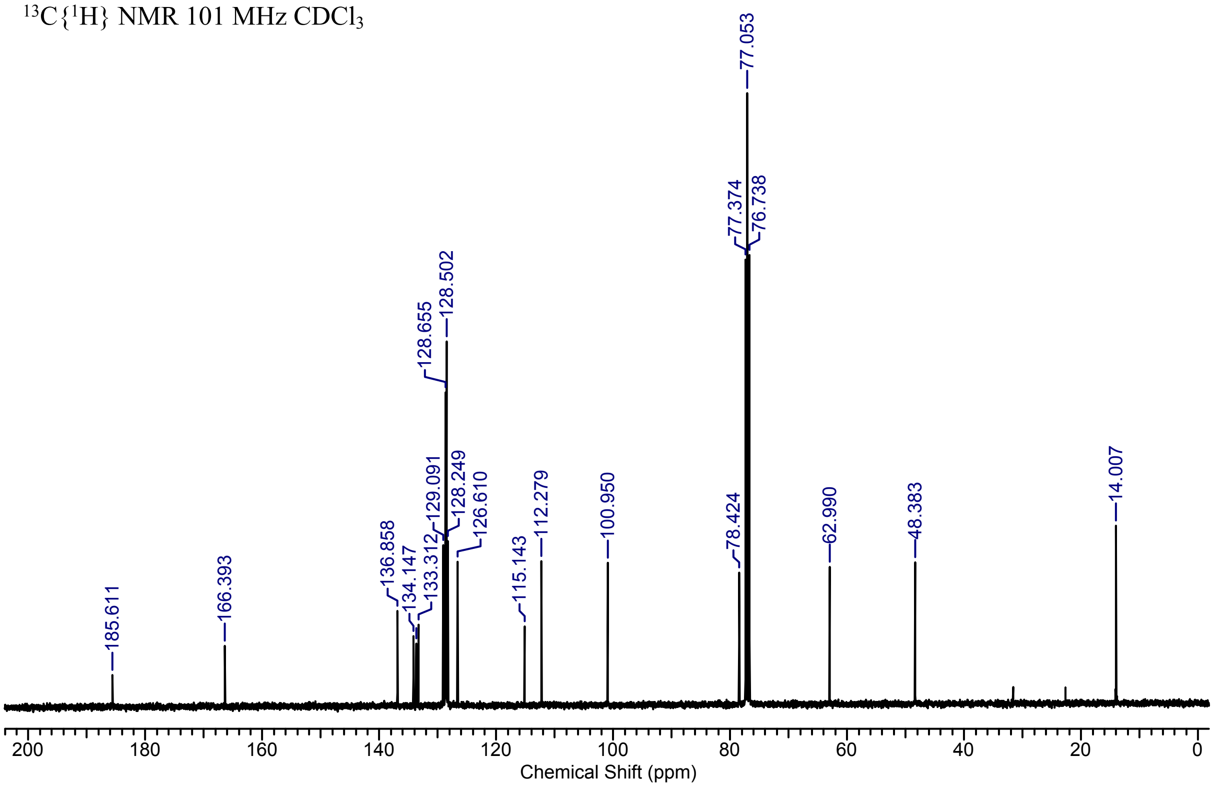


**Figure S81.** $^{13}$C spectrum of compound **2d**.

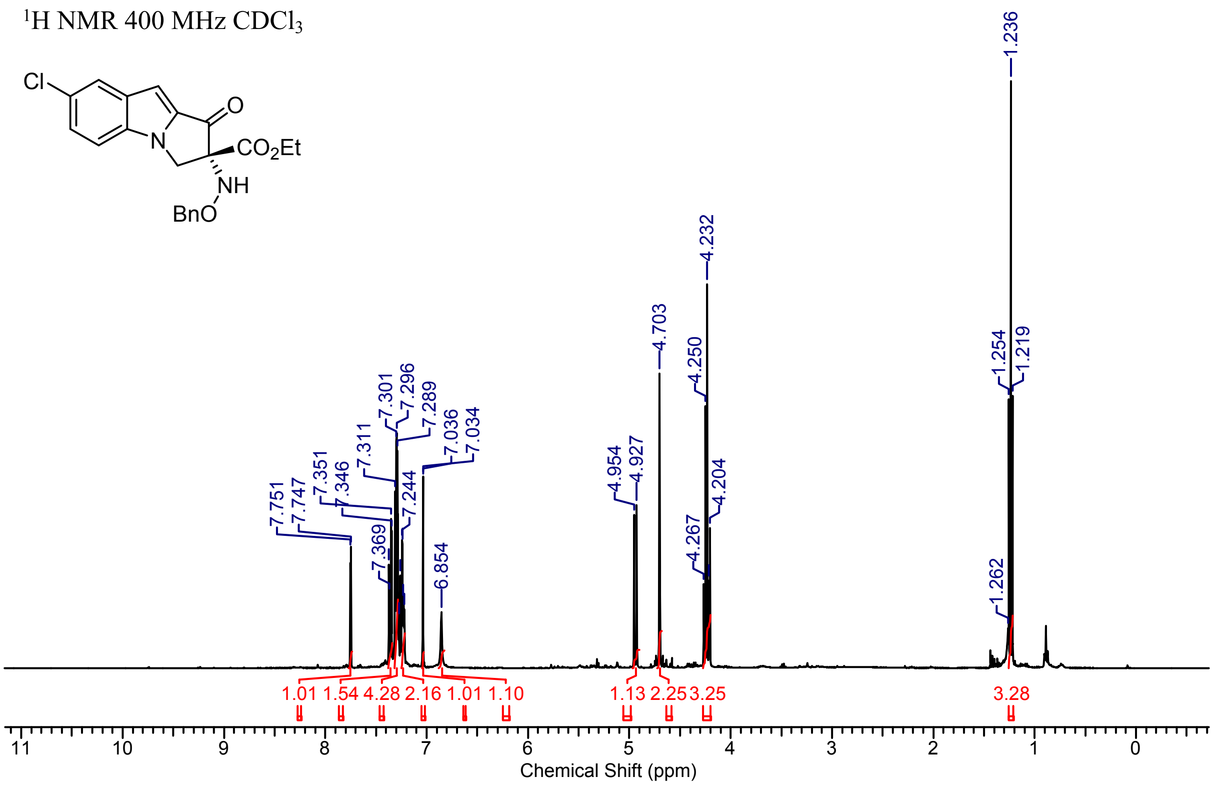


**Figure S82.** $^{1}H$ spectrum of compound **2e**.

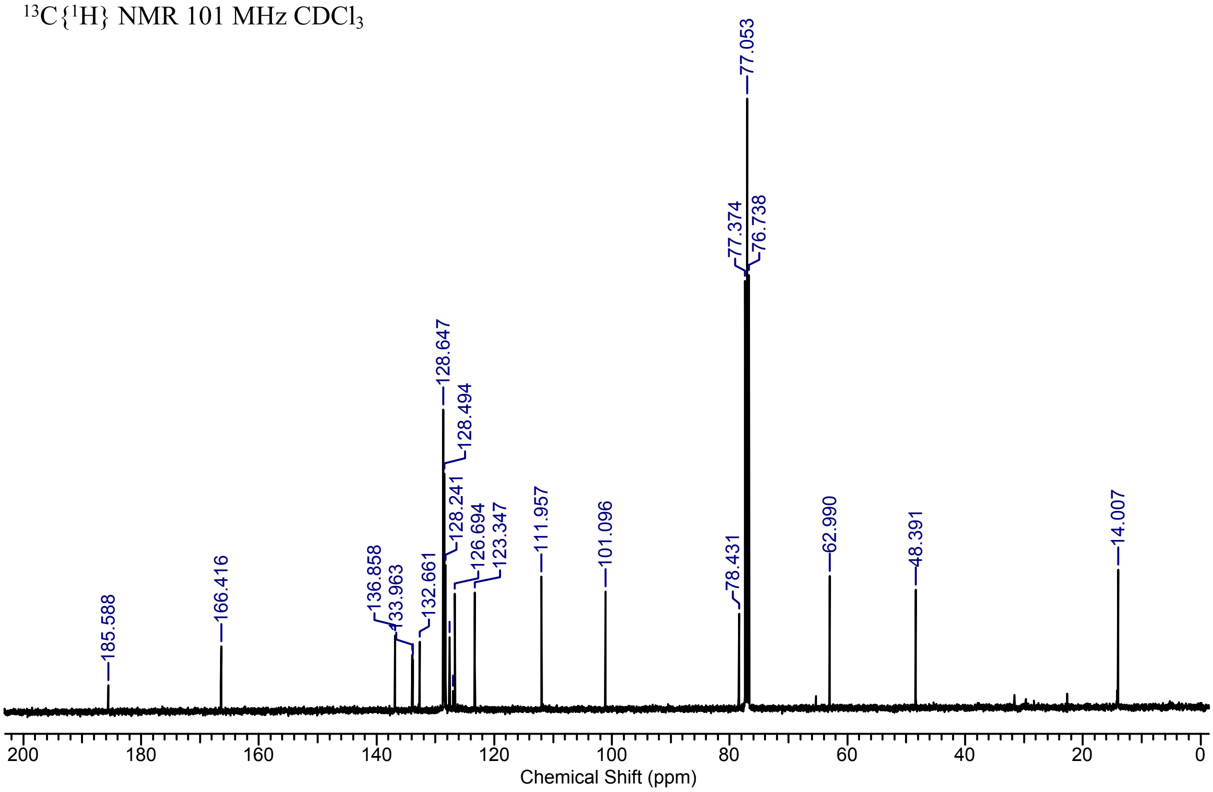


**Figure S83.** $^{13}C$ spectrum of compound **2e**.

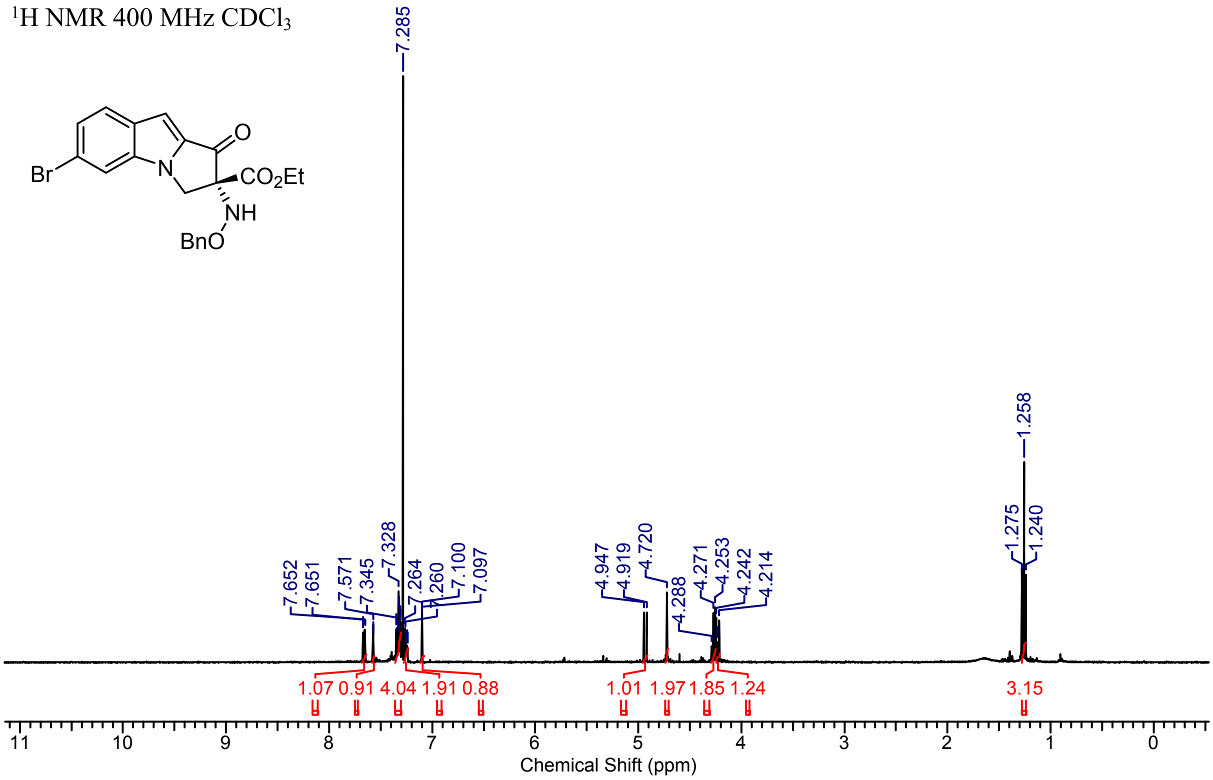


**Figure S84.** $^{1}$H spectrum of compound **2f**.

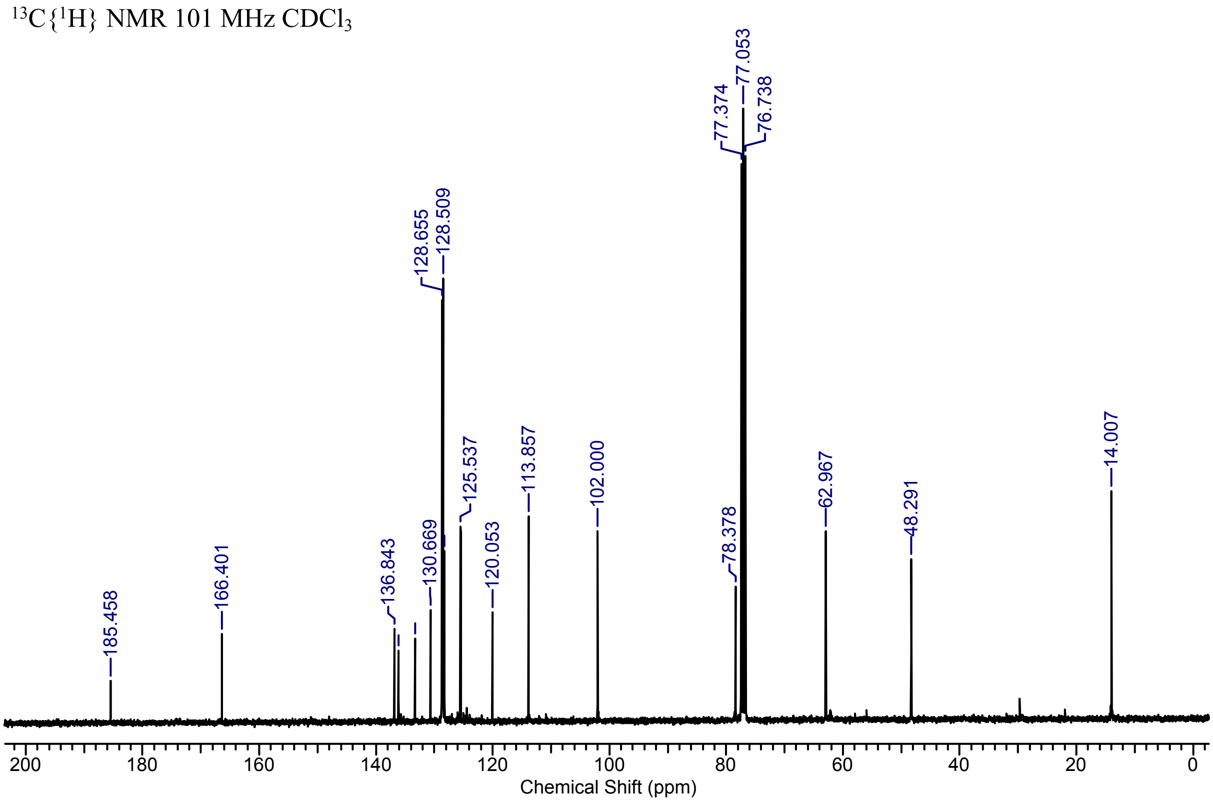


**Figure S85.** $^{13}$C spectrum of compound **2f**.

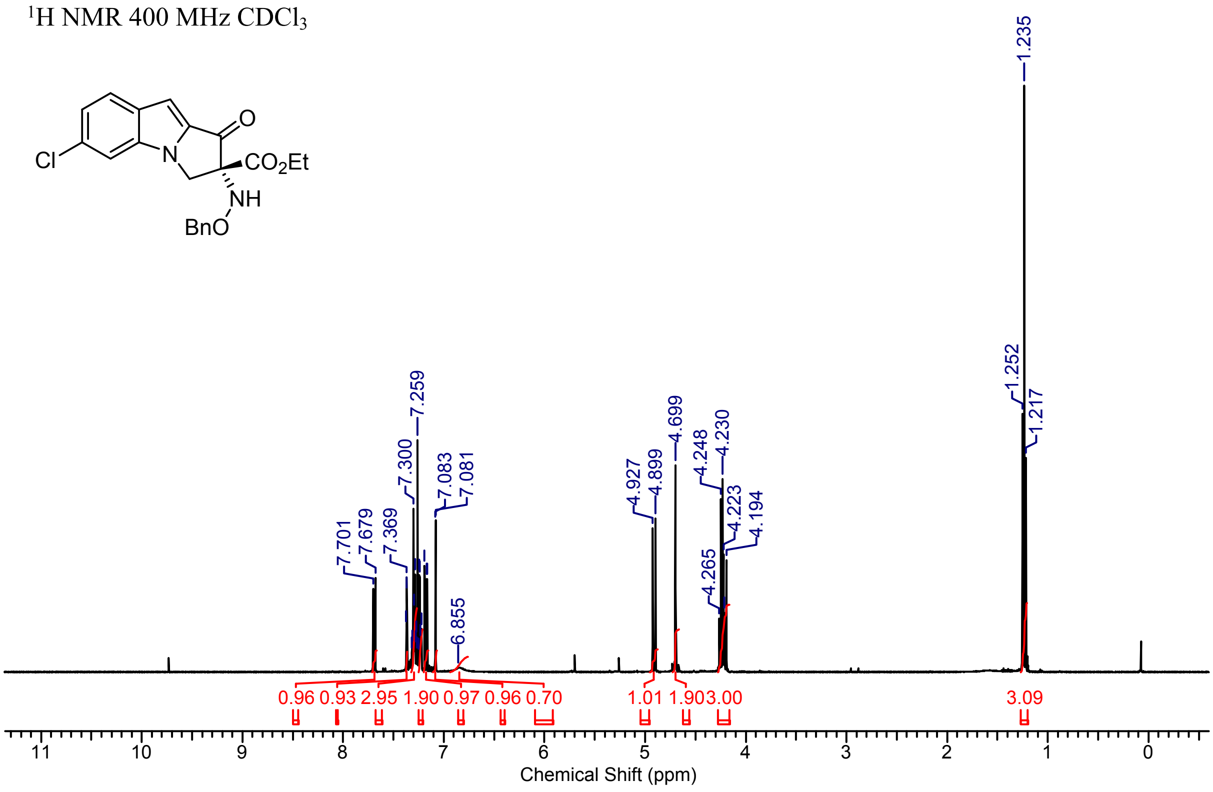


**Figure S86.** $^{1}H$ spectrum of compound **2g**.

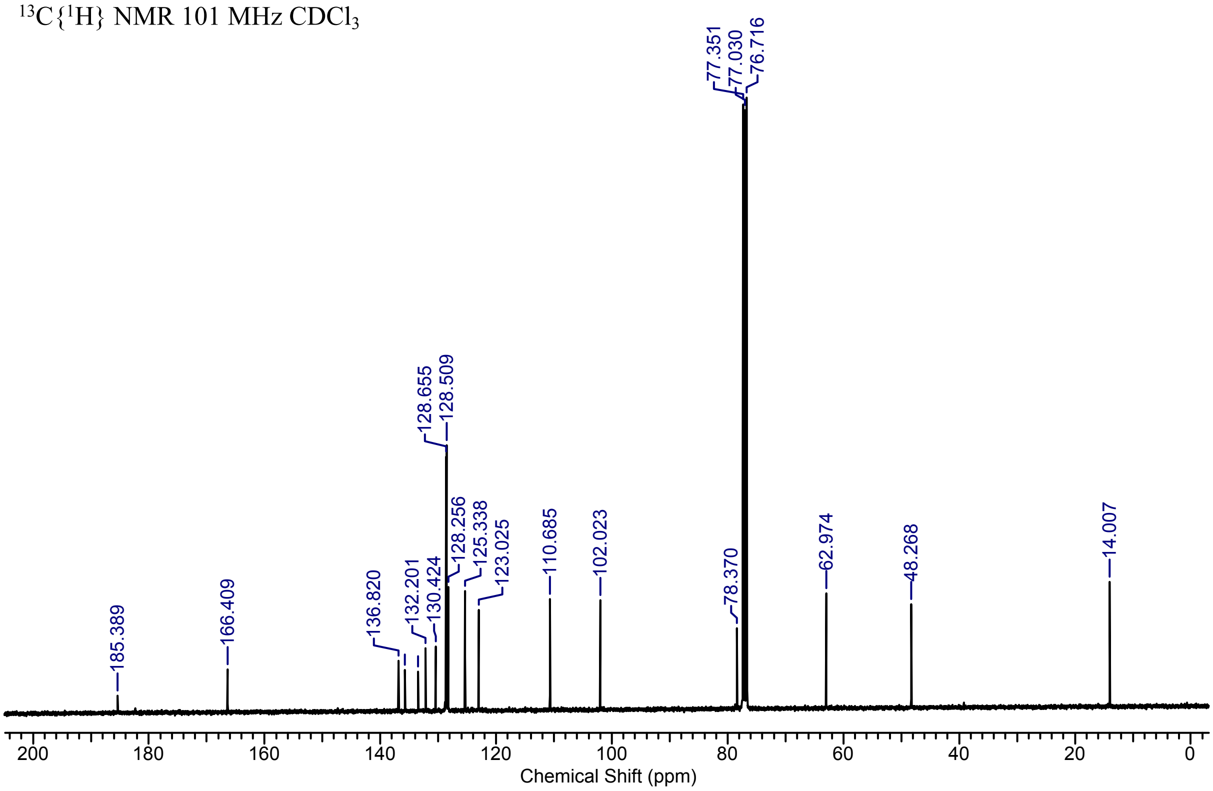


**Figure S87.** $^{13}C$ spectrum of compound **2g**.

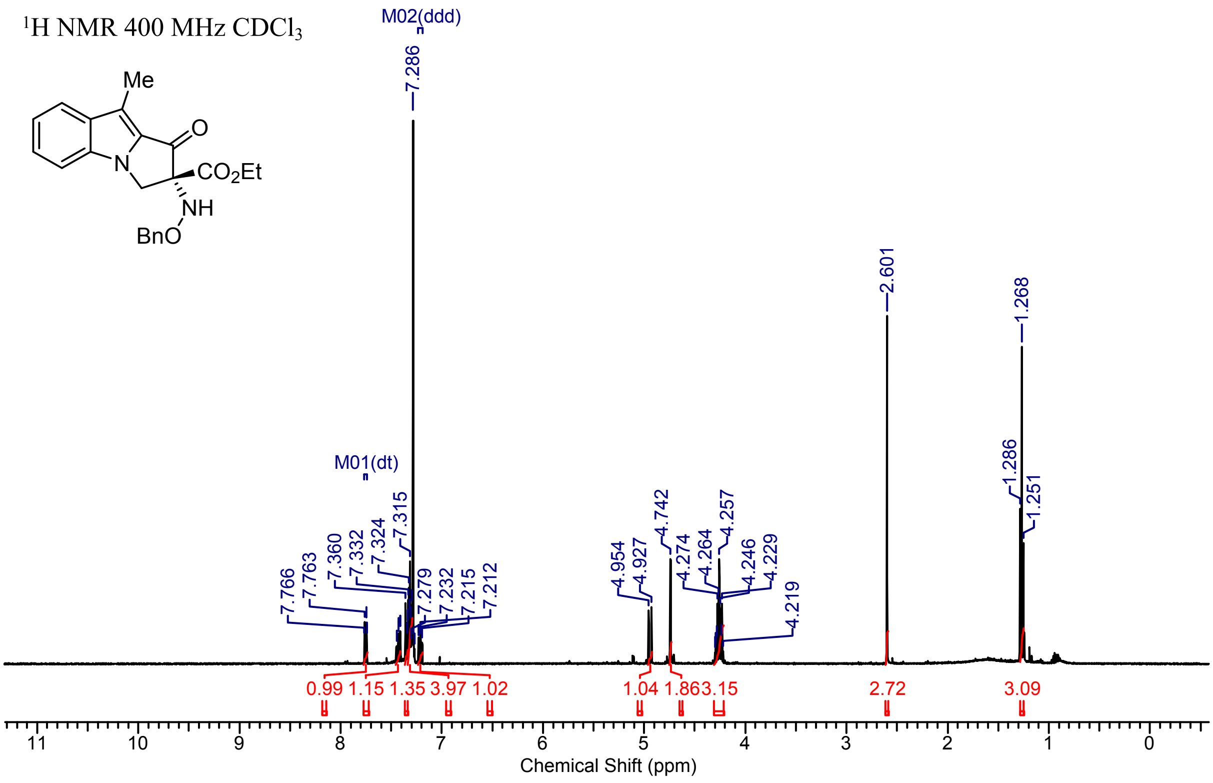


**Figure S88.** $^1$H spectrum of compound **2h**.

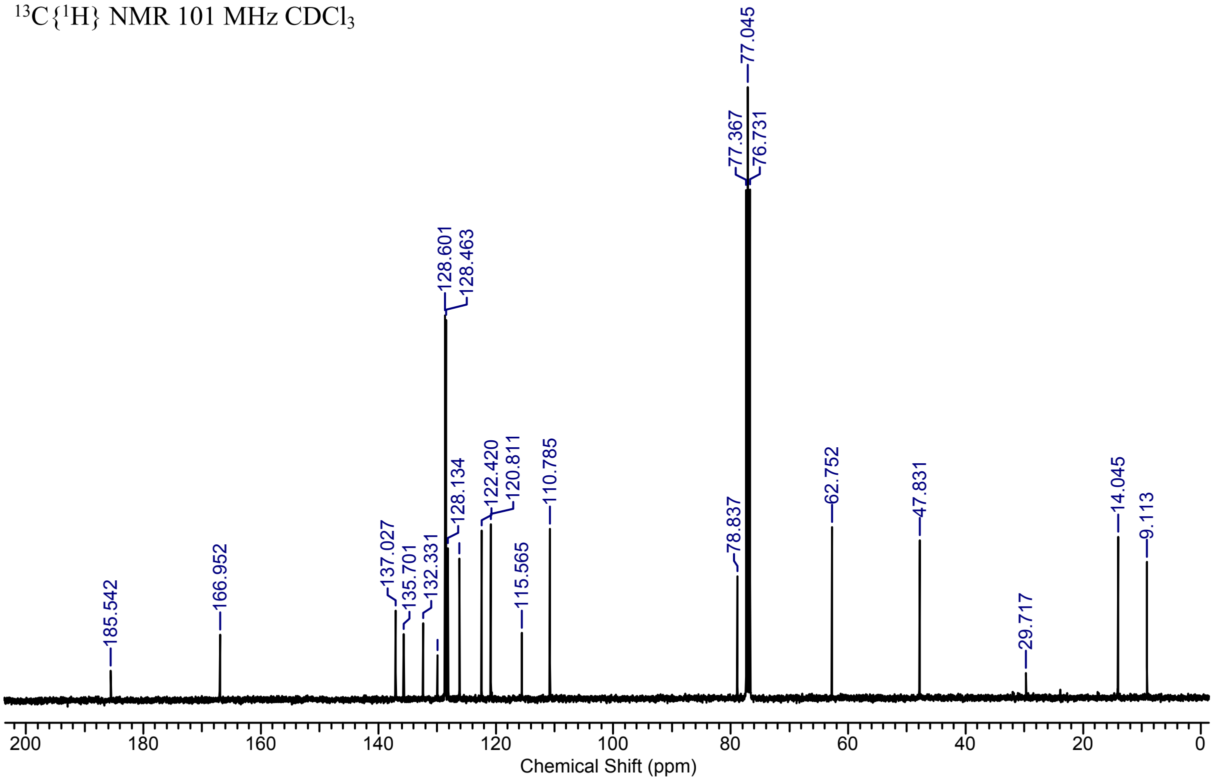


**Figure S89.** $^{13}$C spectrum of compound **2h**.

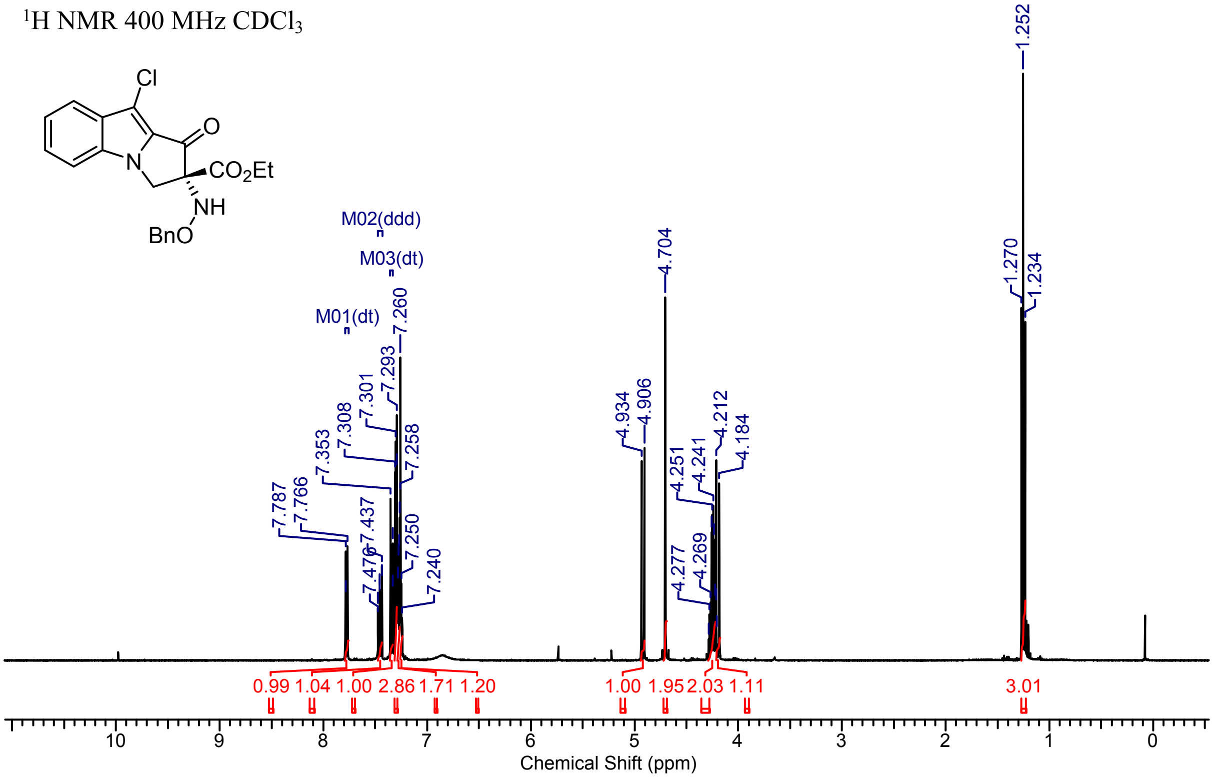


**Figure S90.** $^1$H spectrum of compound **2i**.

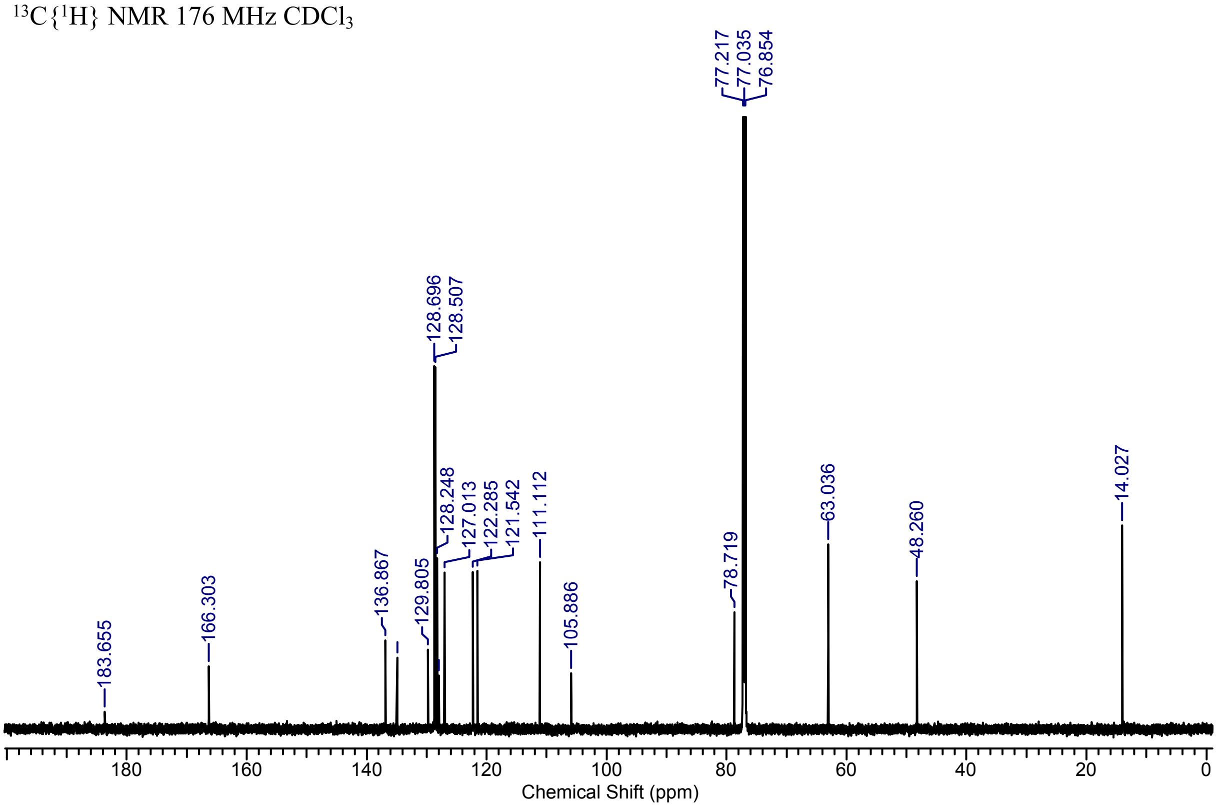


**Figure S91.** $^{13}$C spectrum of compound **2i**.

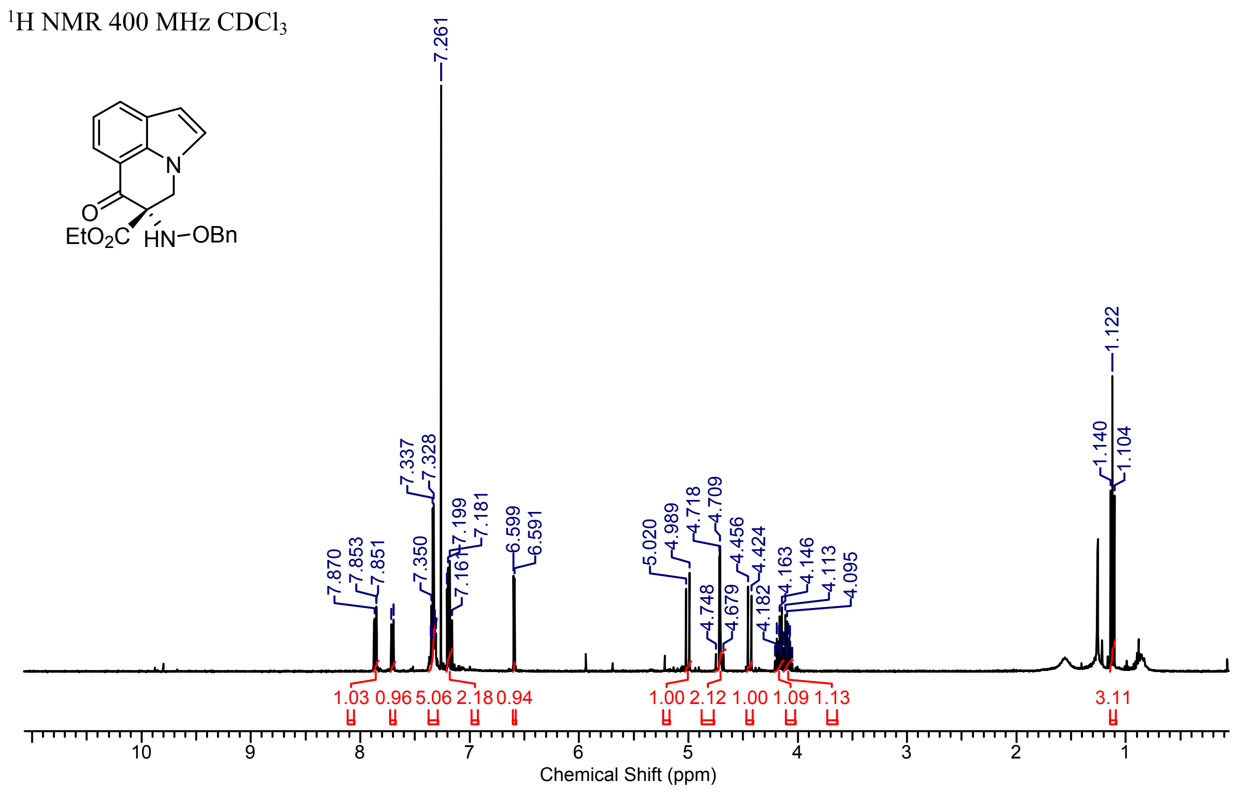


**Figure S92.** $^{1}$H spectrum of compound **2j**.

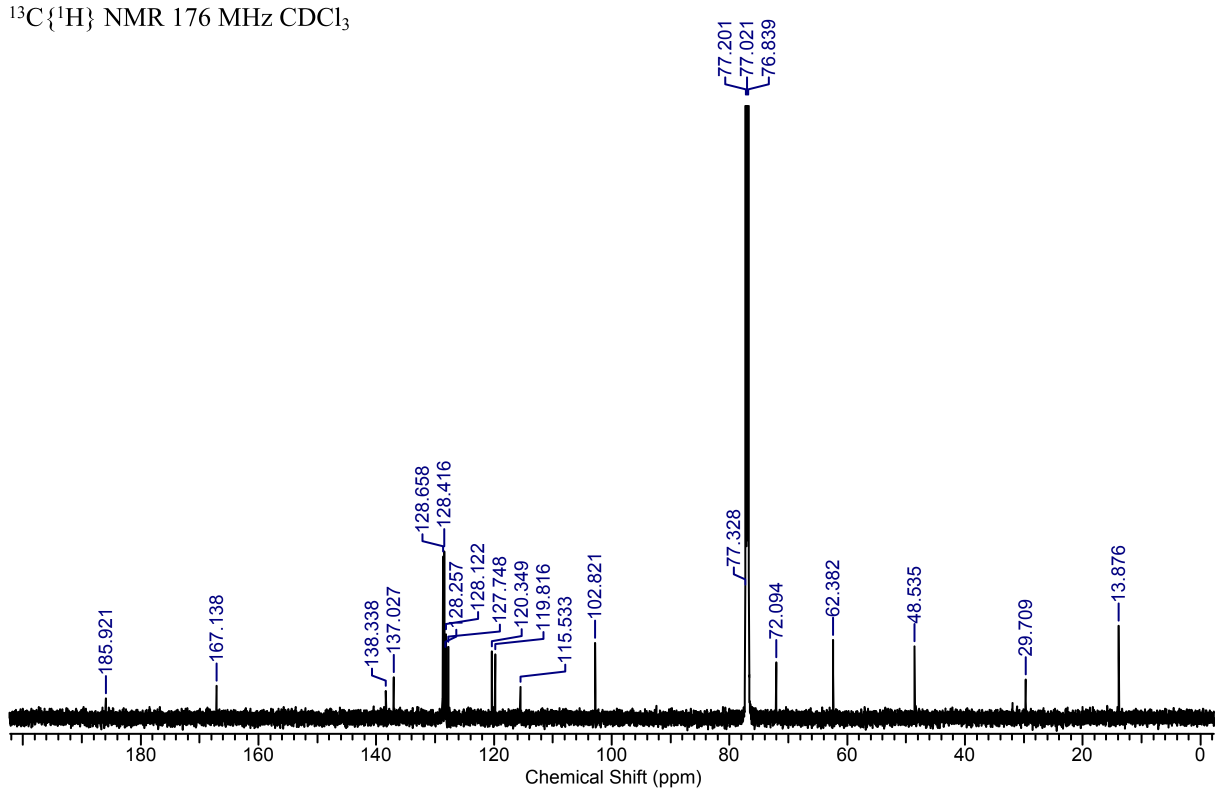


**Figure S93.** $^{13}$C spectrum of compound **2j**.

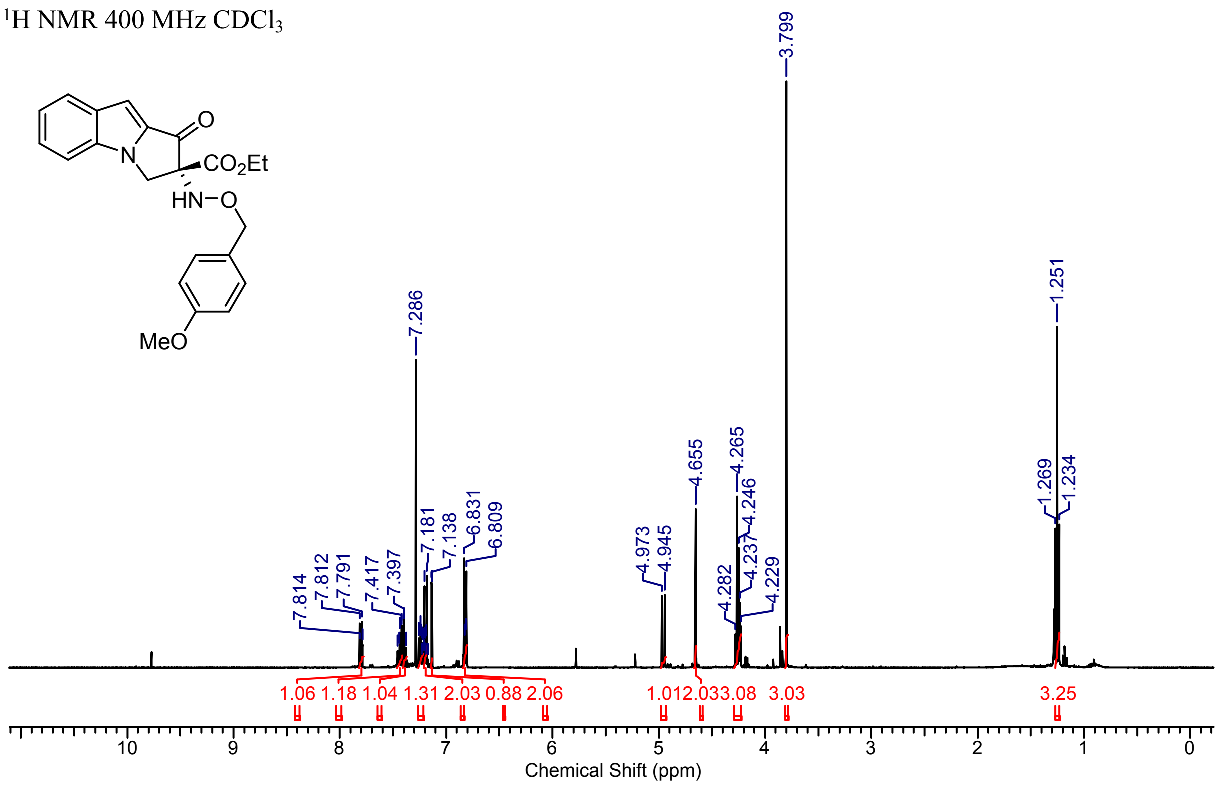


**Figure S94.** $^{1}H$ spectrum of compound **2n**.

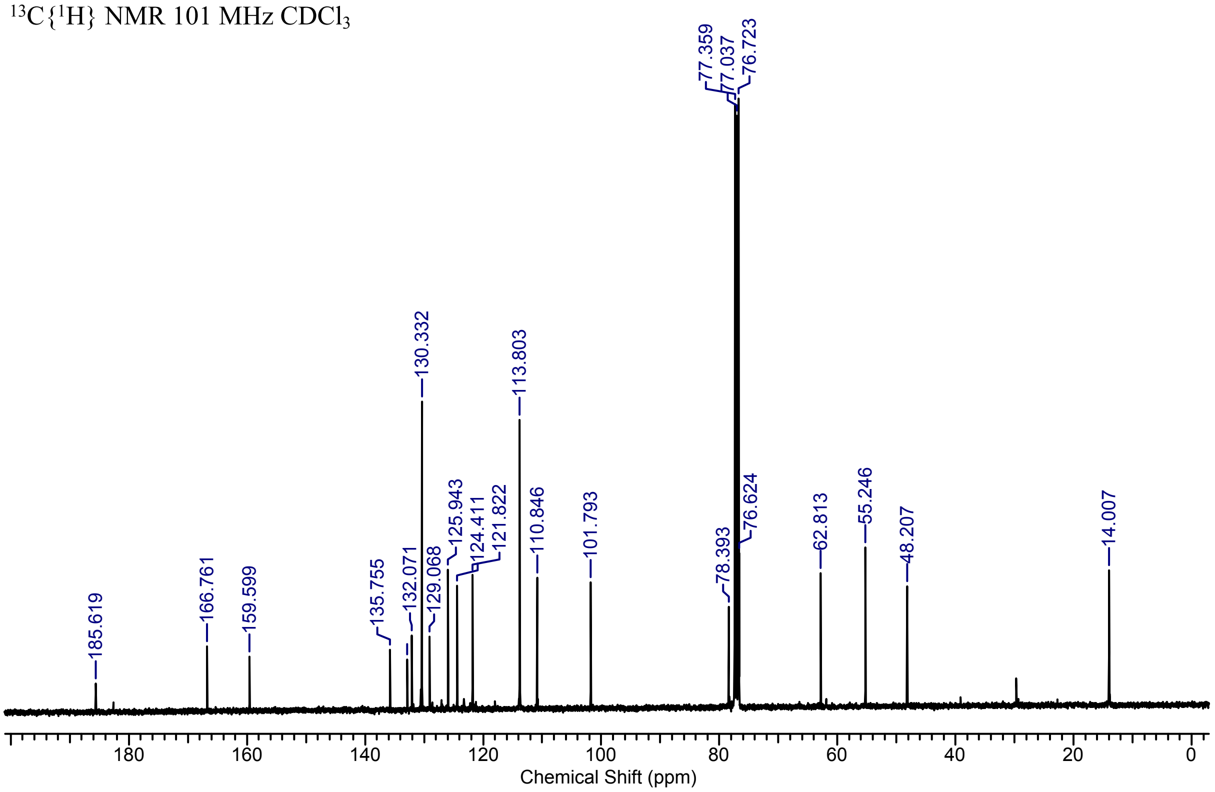


**Figure S95.** $^{13}C$ spectrum of compound **2n**.

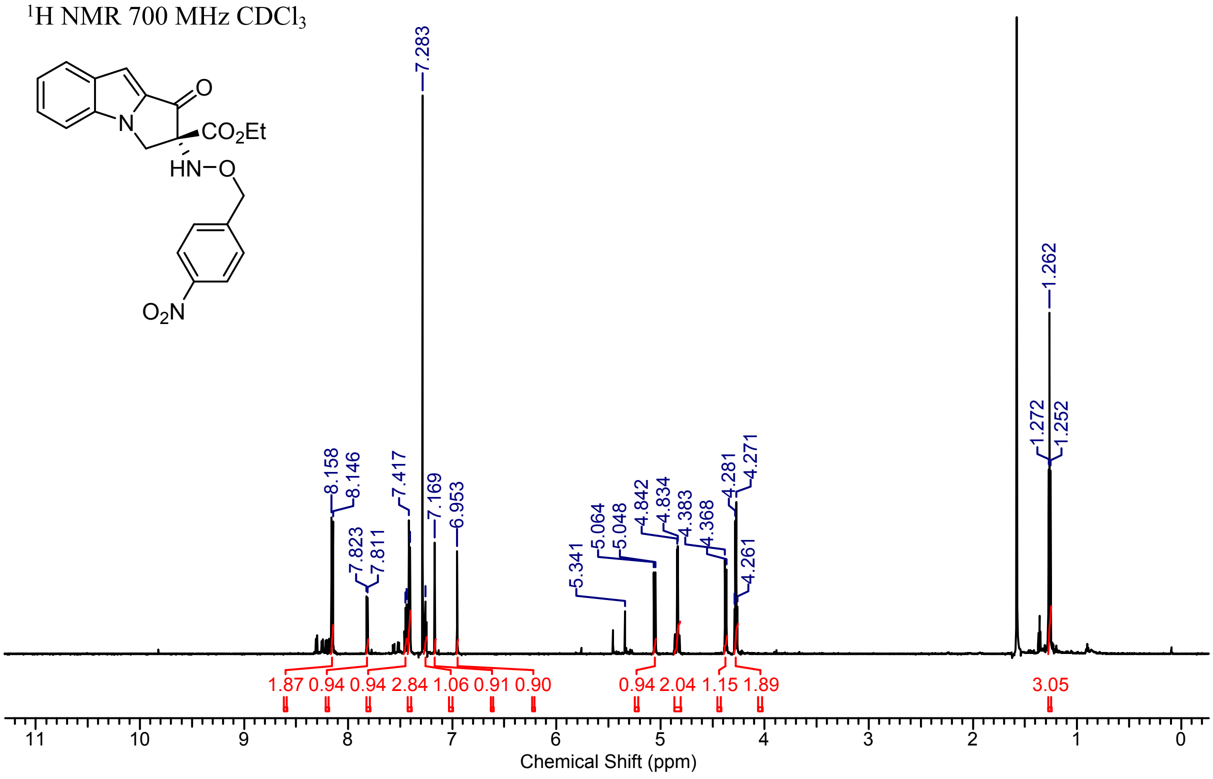


**Figure S96.** $^1H$ spectrum of compound **2o**.

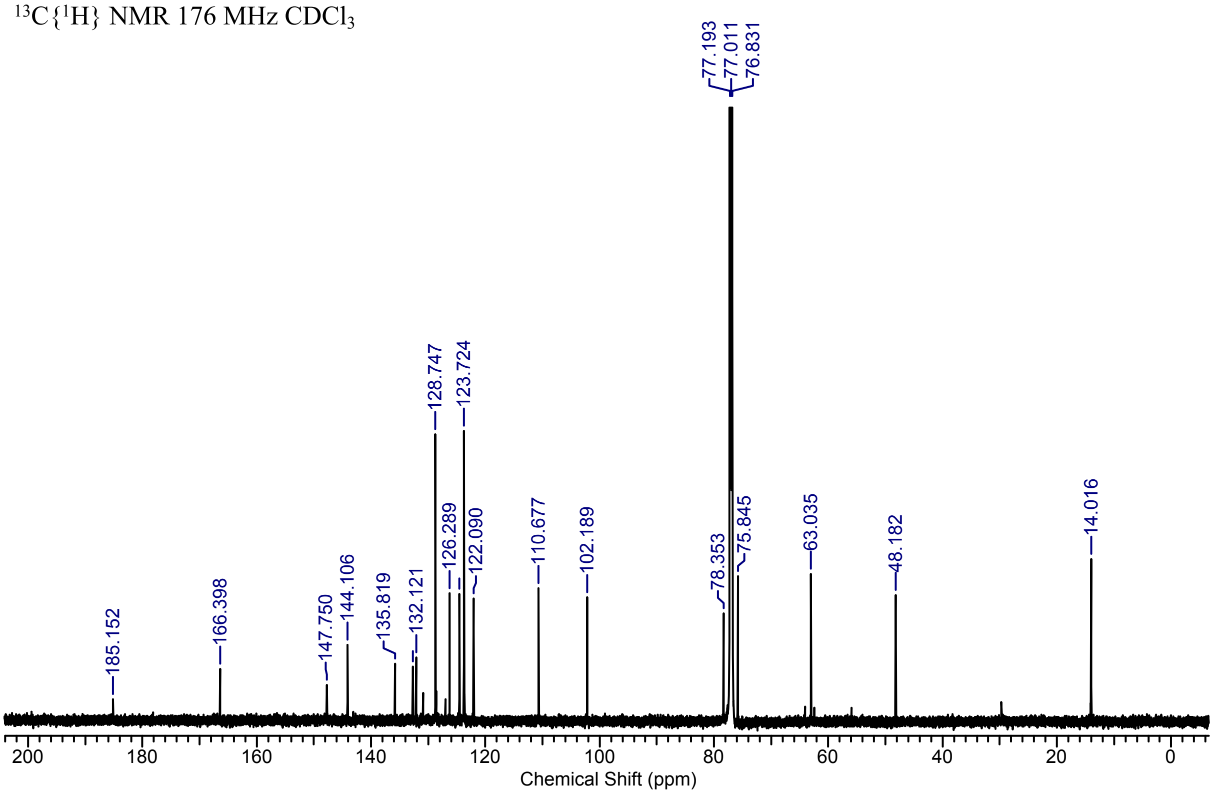


**Figure S97.** $^{13}C$ spectrum of compound **2o**.

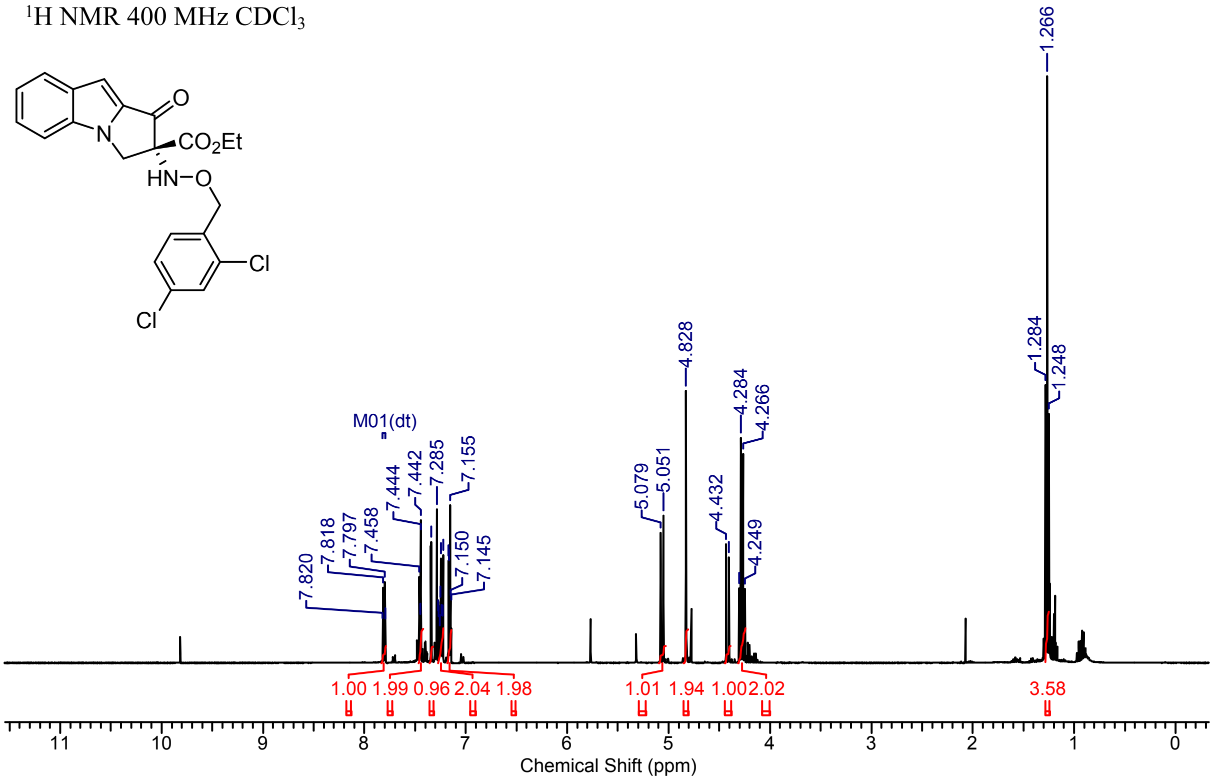


**Figure S98.** $^{1}H$ spectrum of compound **2p**.

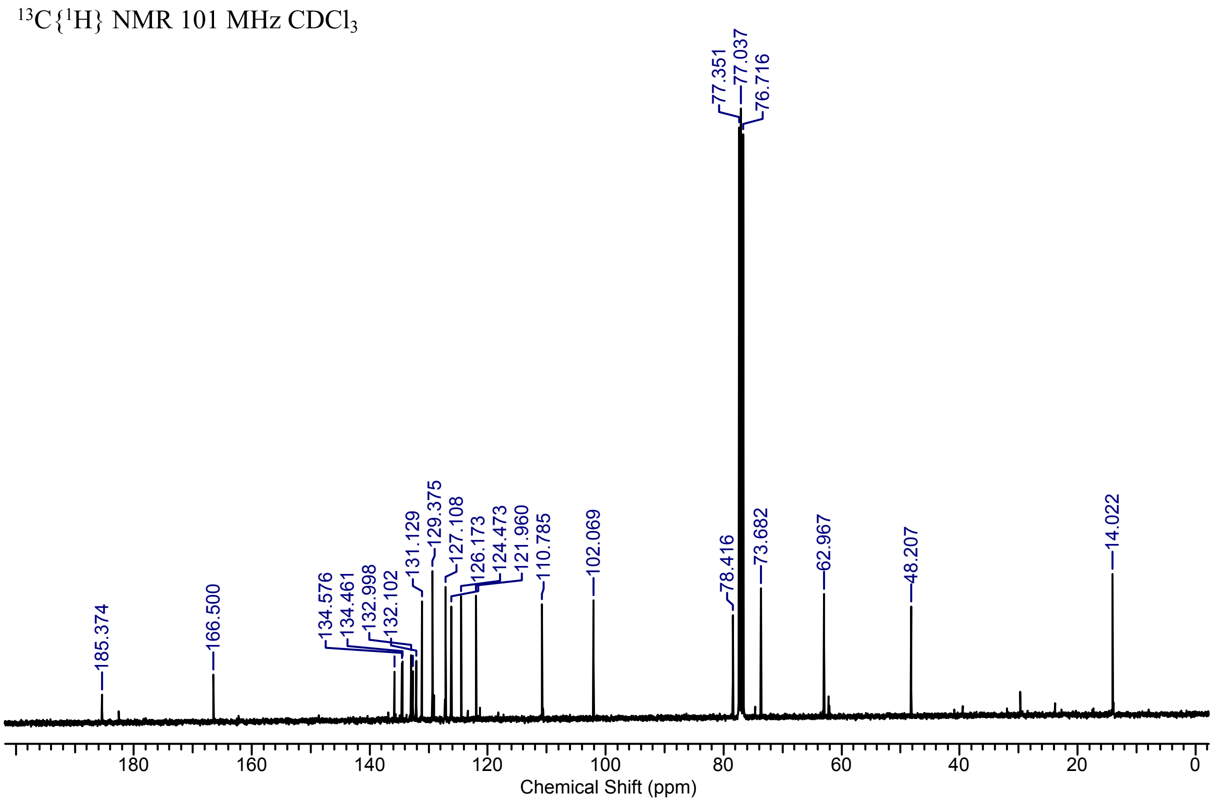


**Figure S99.** $^{13}C$ spectrum of compound **2p**.

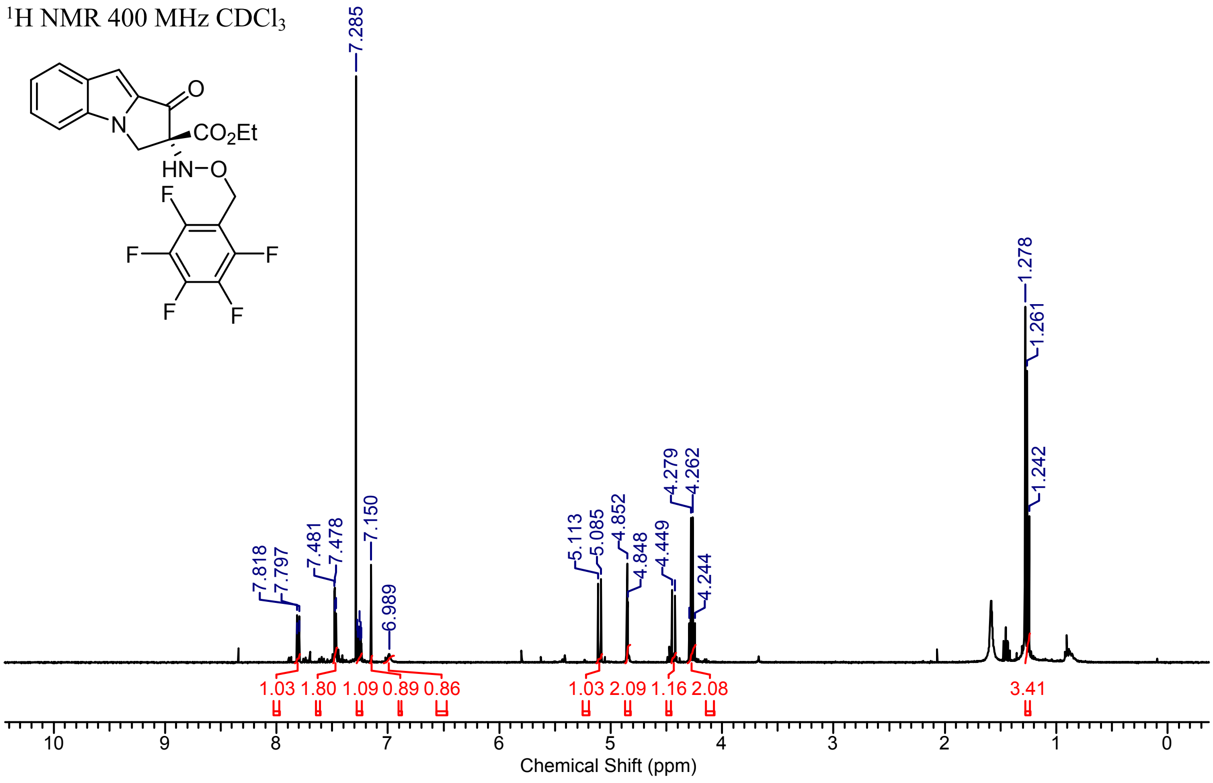


**Figure S100.** $^{1}$H spectrum of compound **2q**.

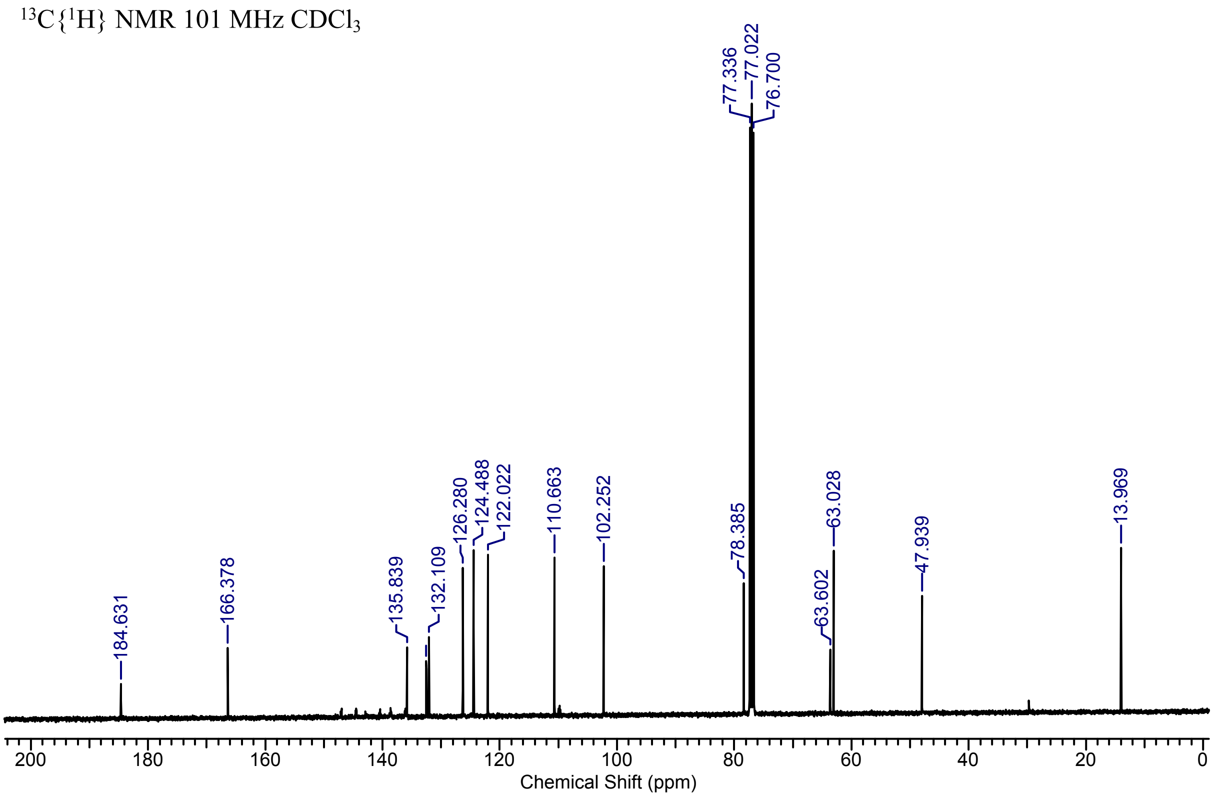


**Figure S101.** $^{13}$C spectrum of compound **2q**.

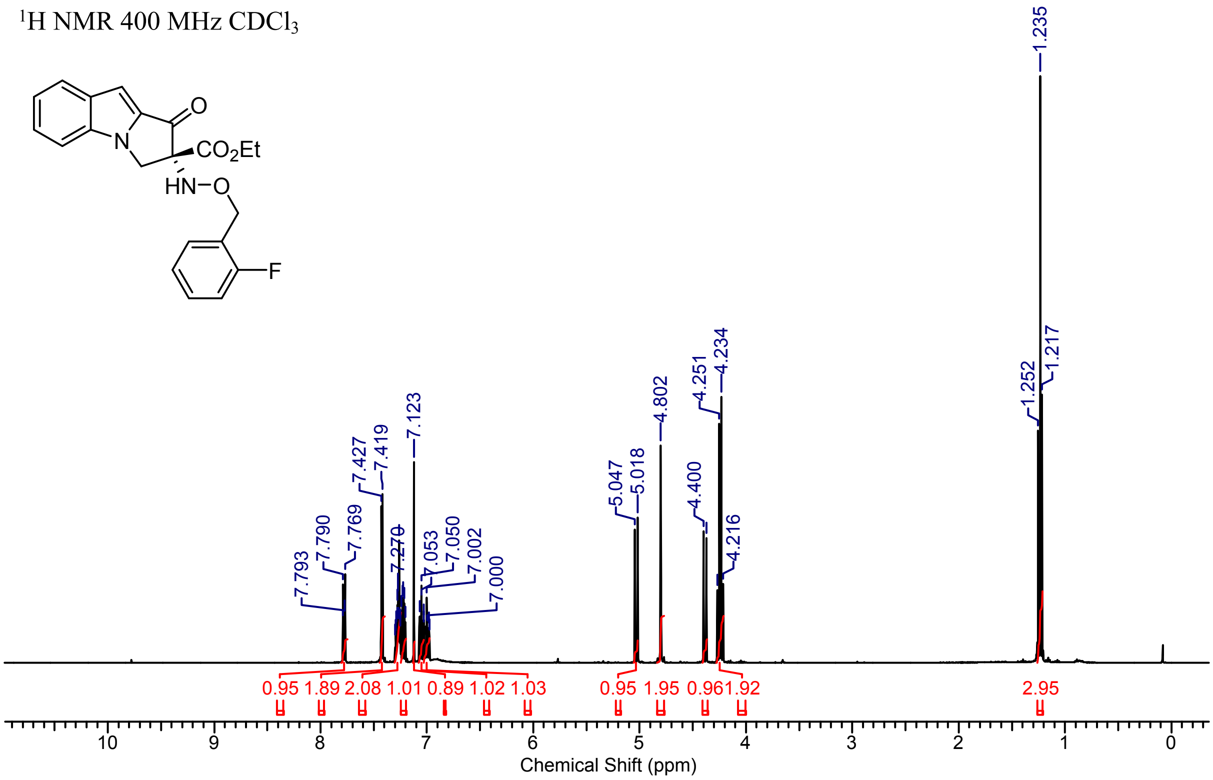


**Figure S102.** $^{1}$H spectrum of compound **2r**.

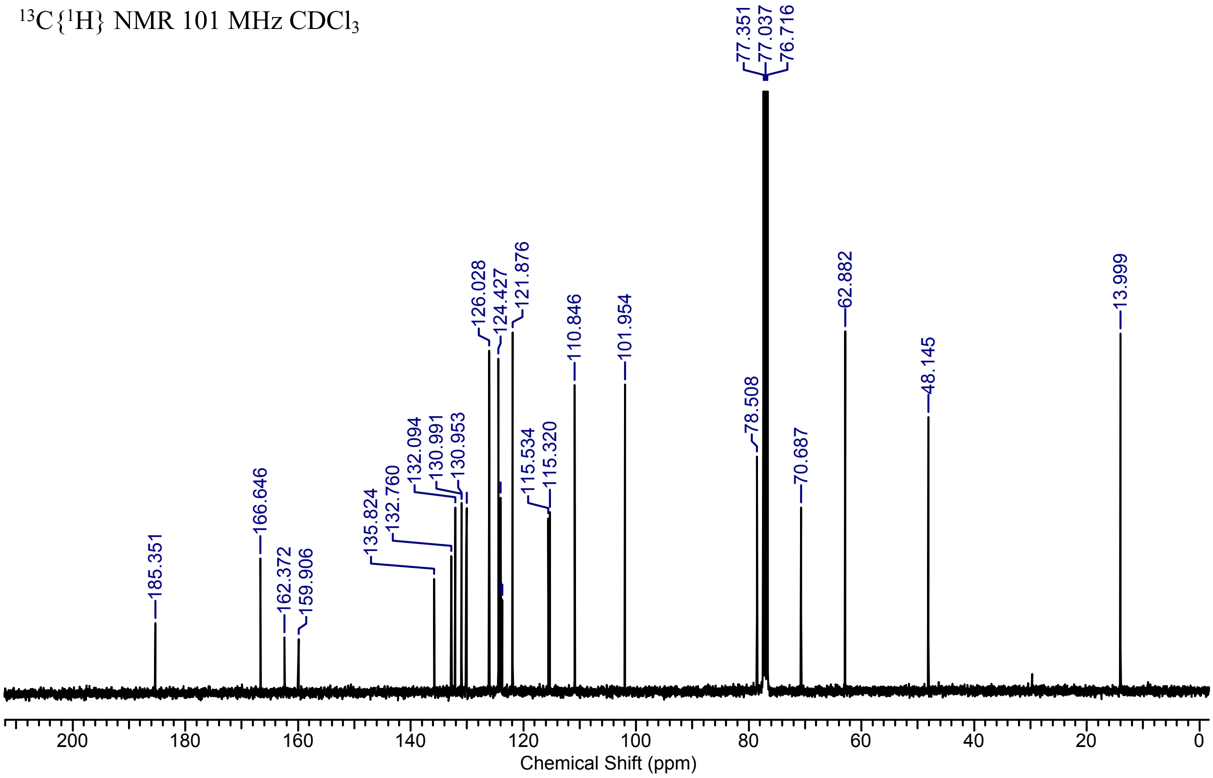


**Figure S103.** $^{13}$C spectrum of compound **2r**.

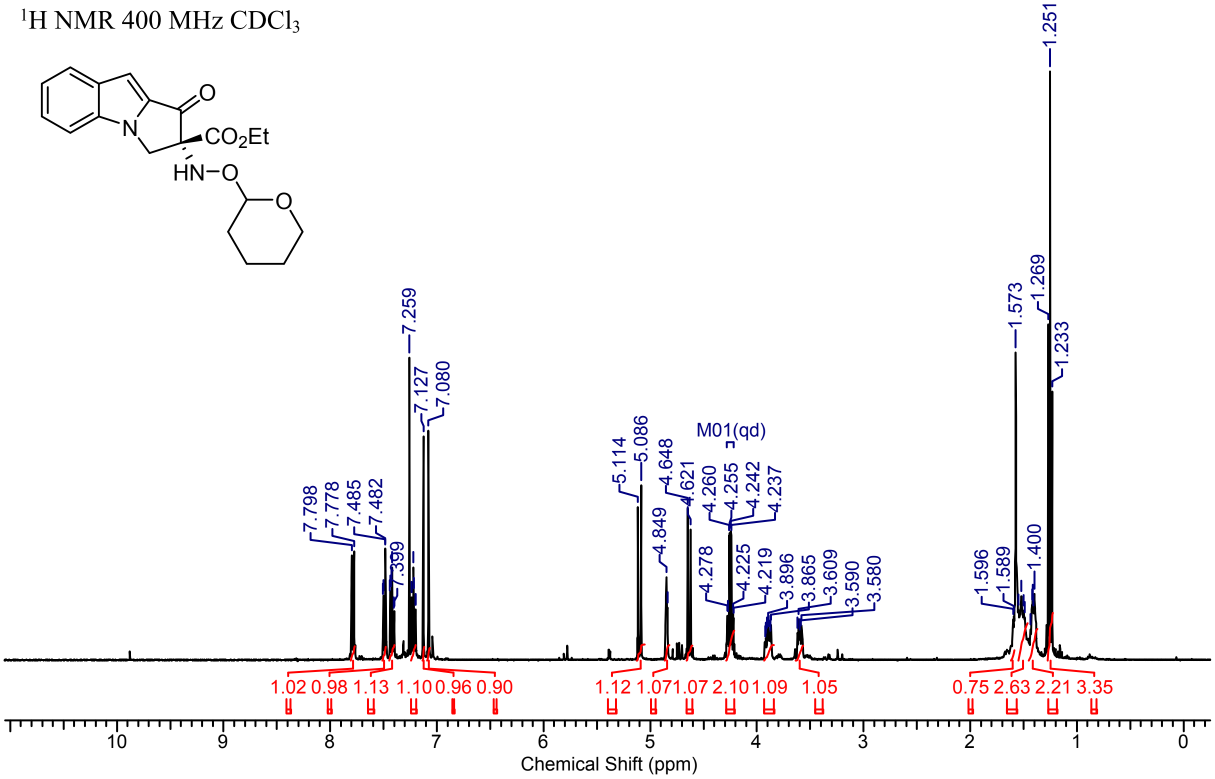


**Figure S104.** $^{1}$H spectrum of compound **2s**.

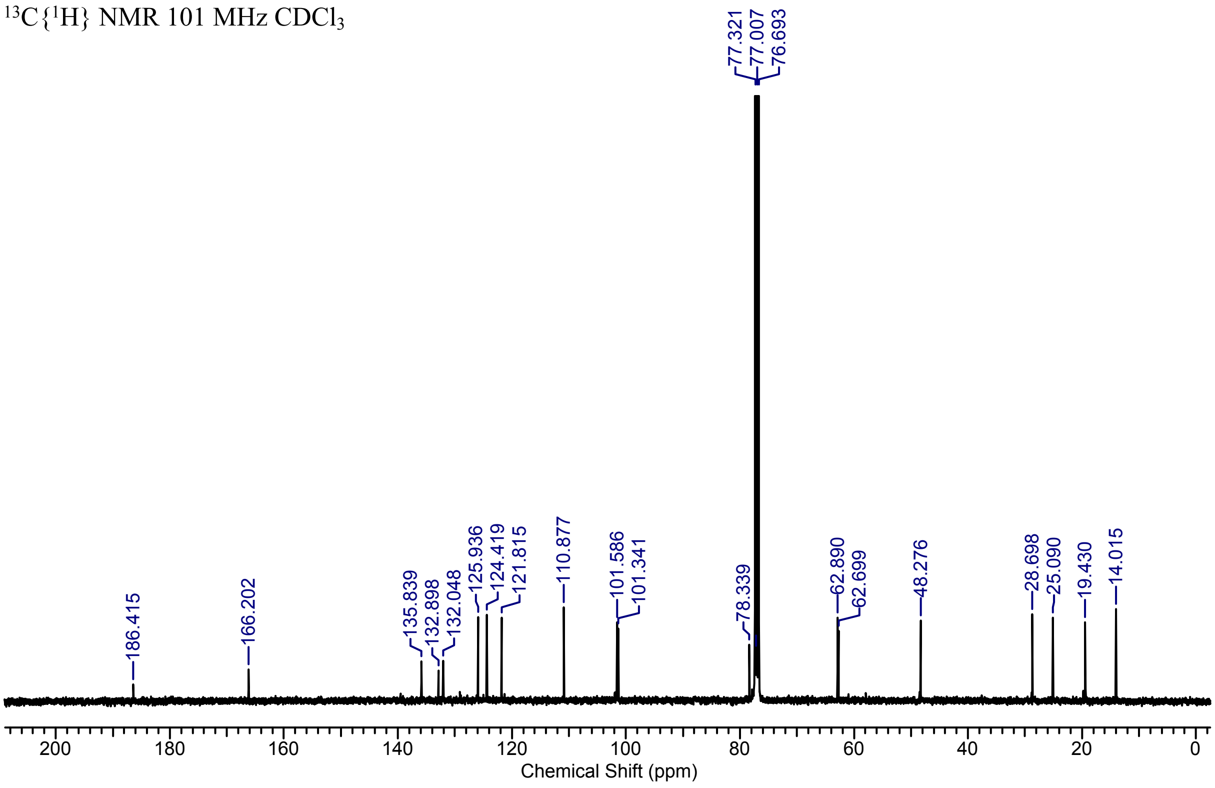


**Figure S105.** $^{13}$C spectrum of compound **2s**.

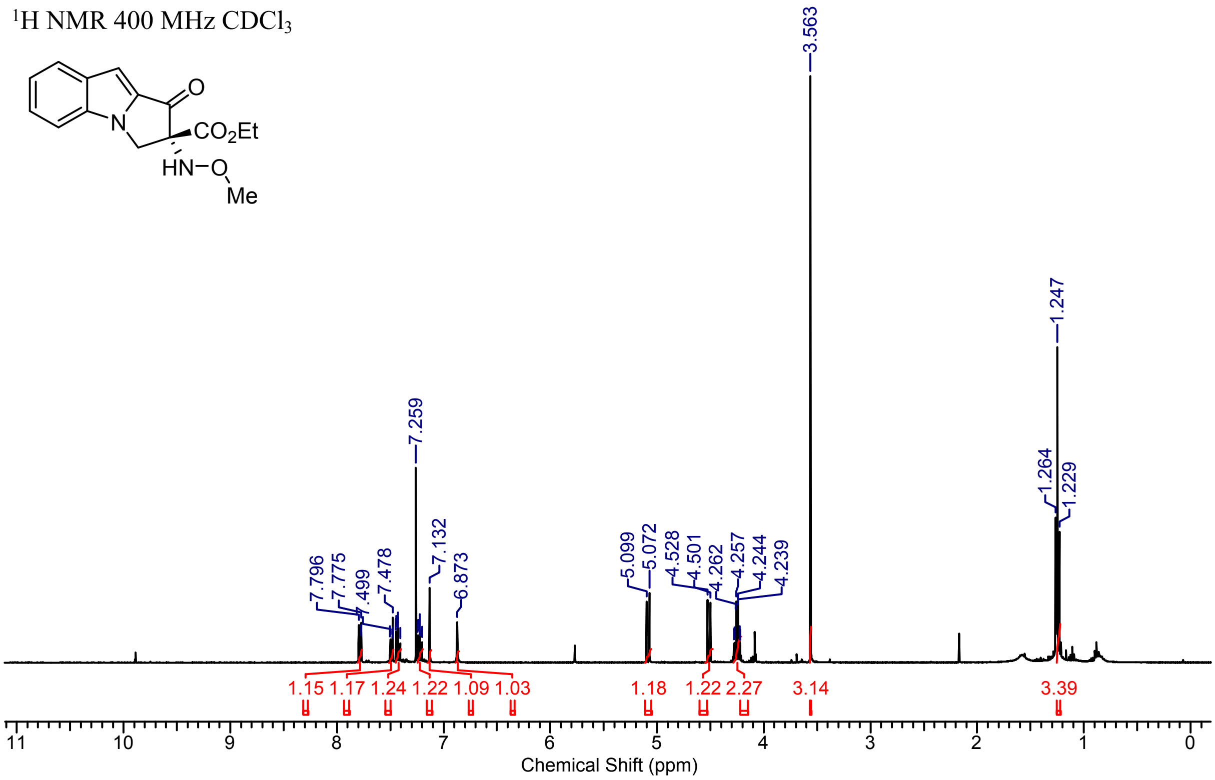


**Figure S106.** $^{1}H$ spectrum of compound **2t**.

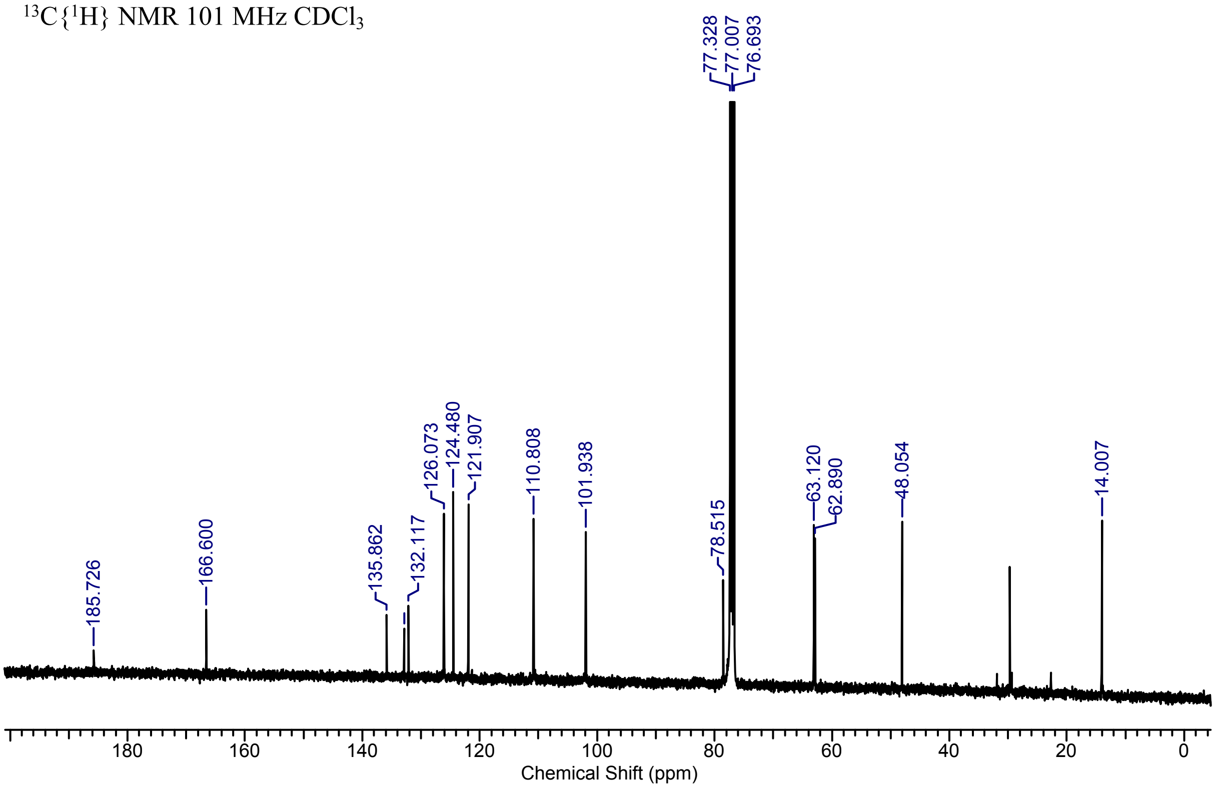


**Figure S107.** $^{13}C$ spectrum of compound **2t**.

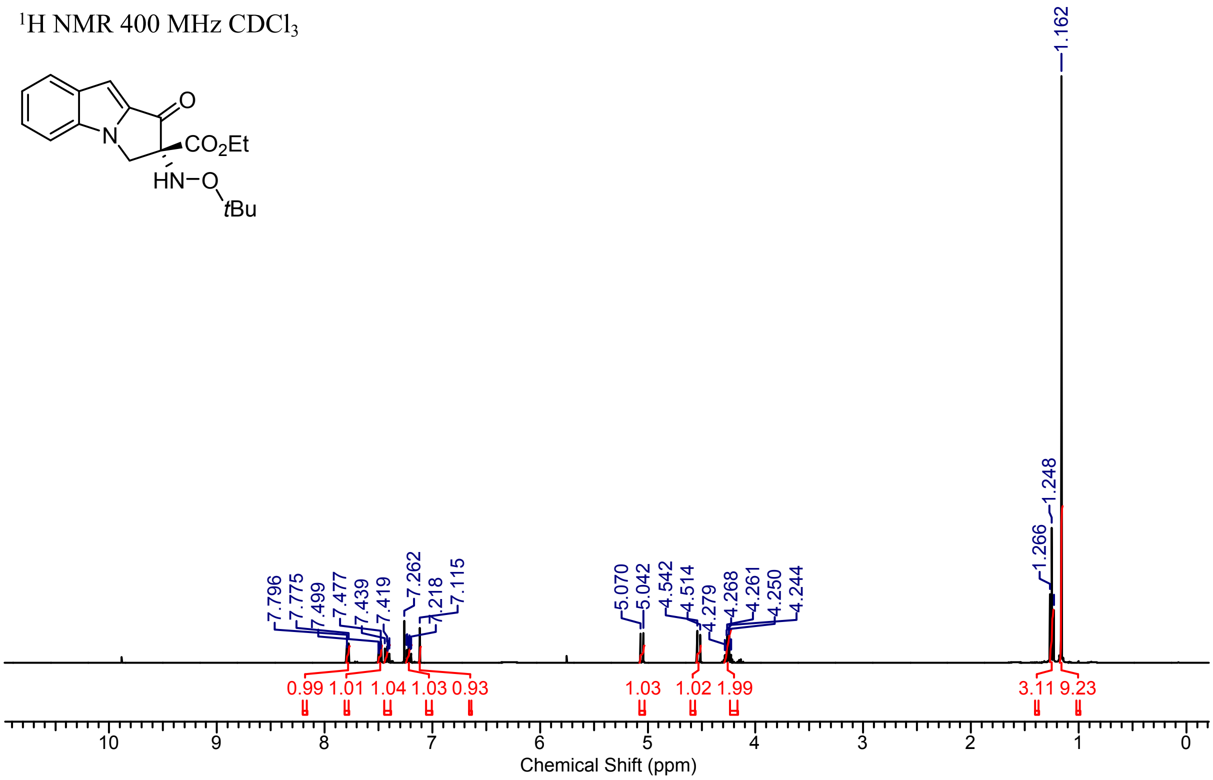


**Figure S108.** $^{1}H$ spectrum of compound **2u**.

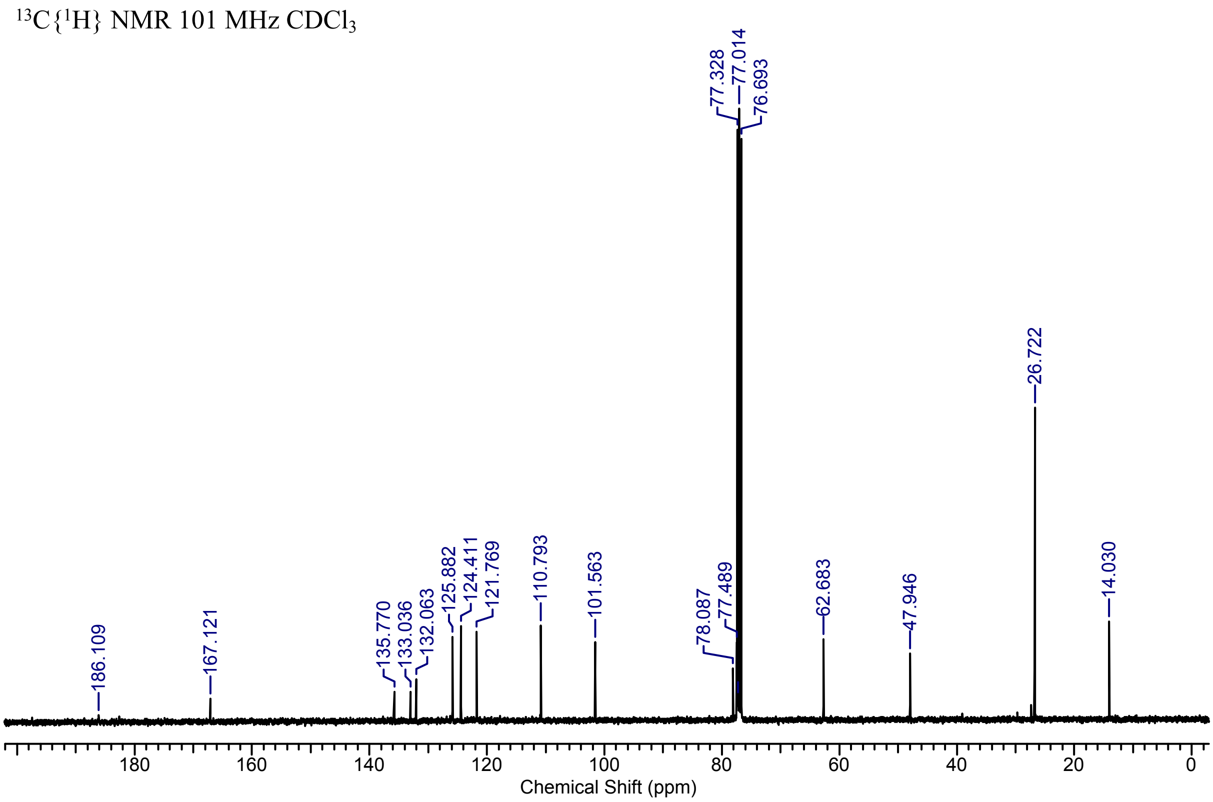


**Figure S109.** $^{13}C$ spectrum of compound **2u**.

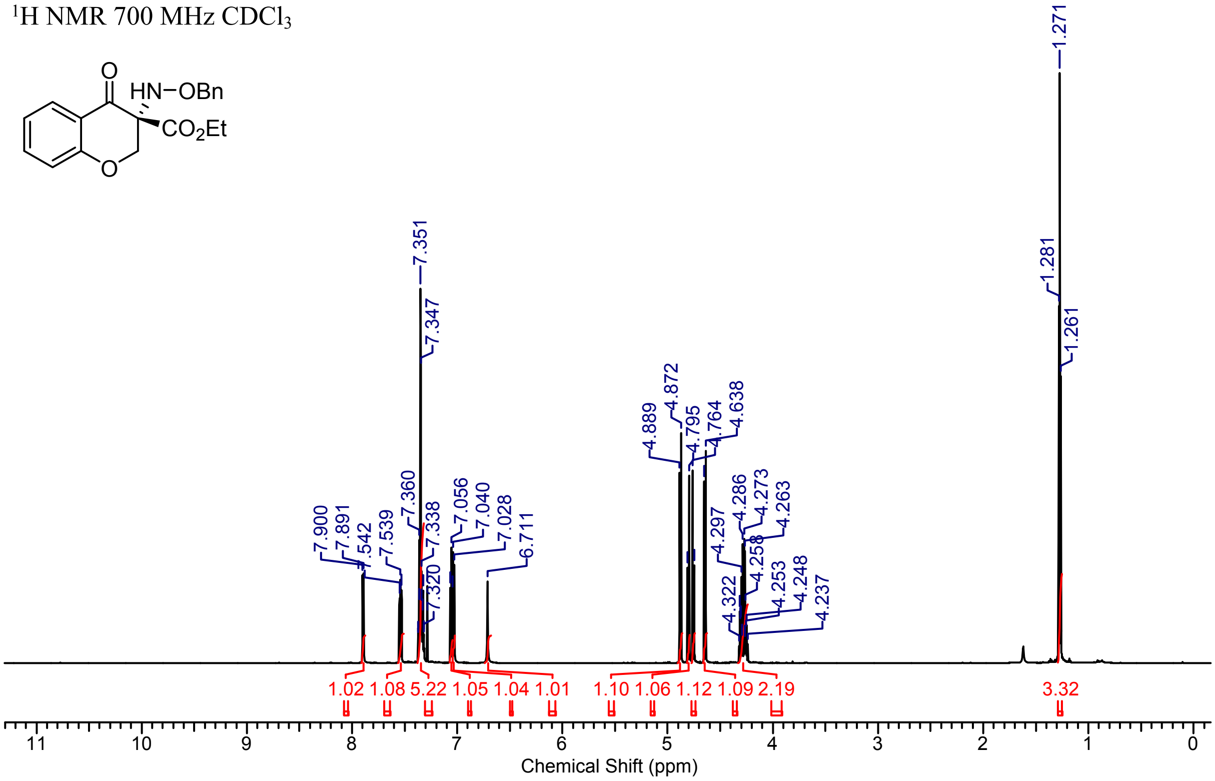


**Figure S110.** $^1$H spectrum of compound **4a**.

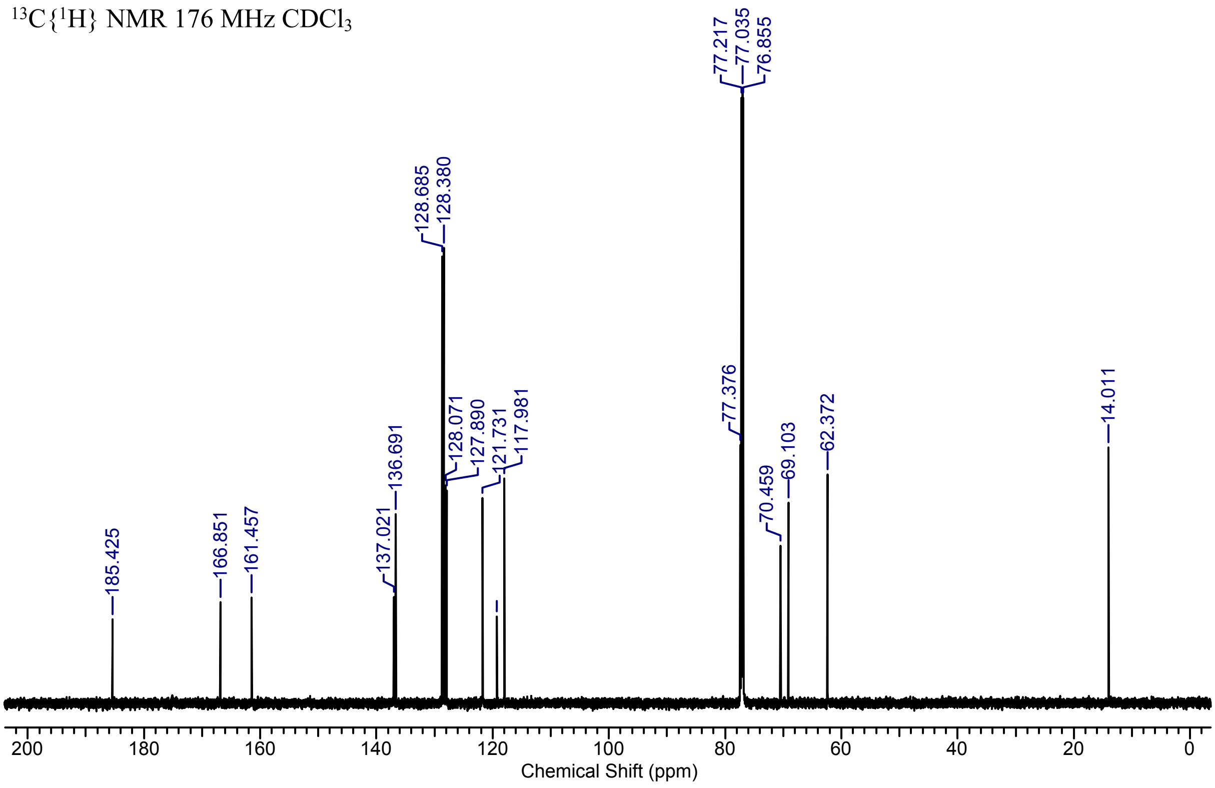


**Figure S111.** $^{13}$C spectrum of compound **4a**.

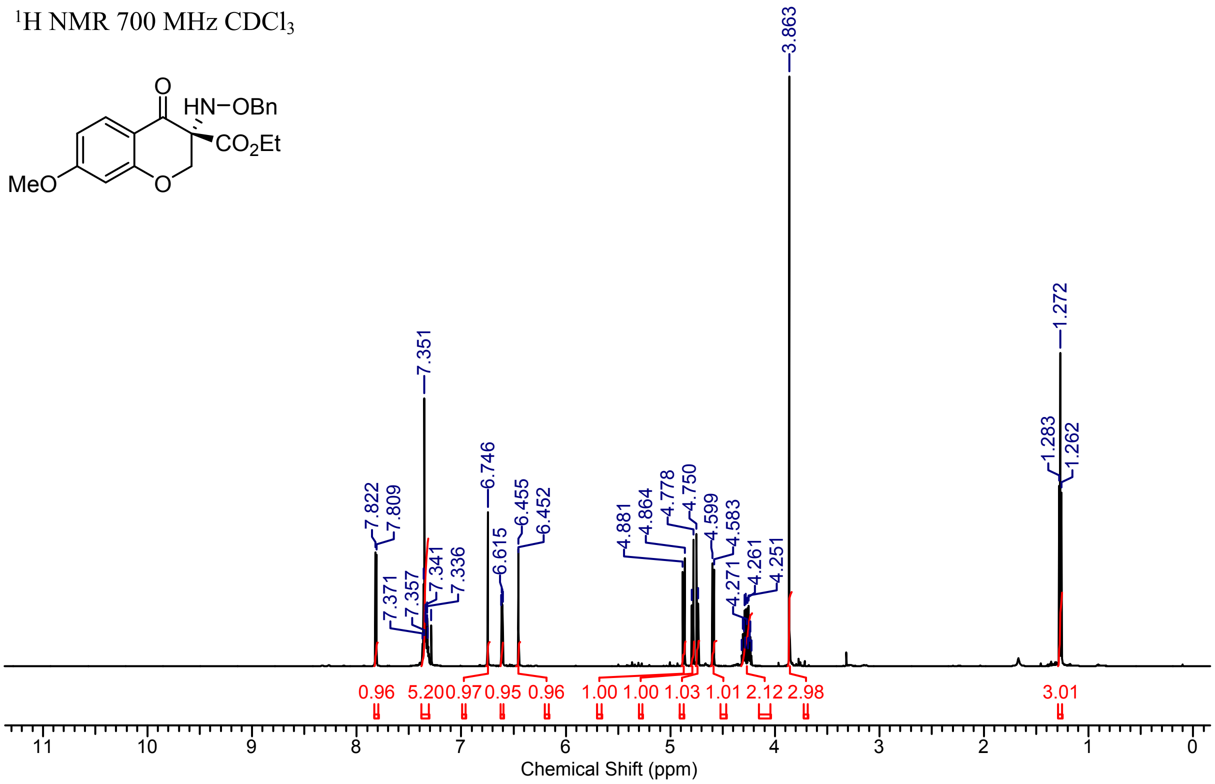


**Figure S112.** $^{1}H$ spectrum of compound **4b**.

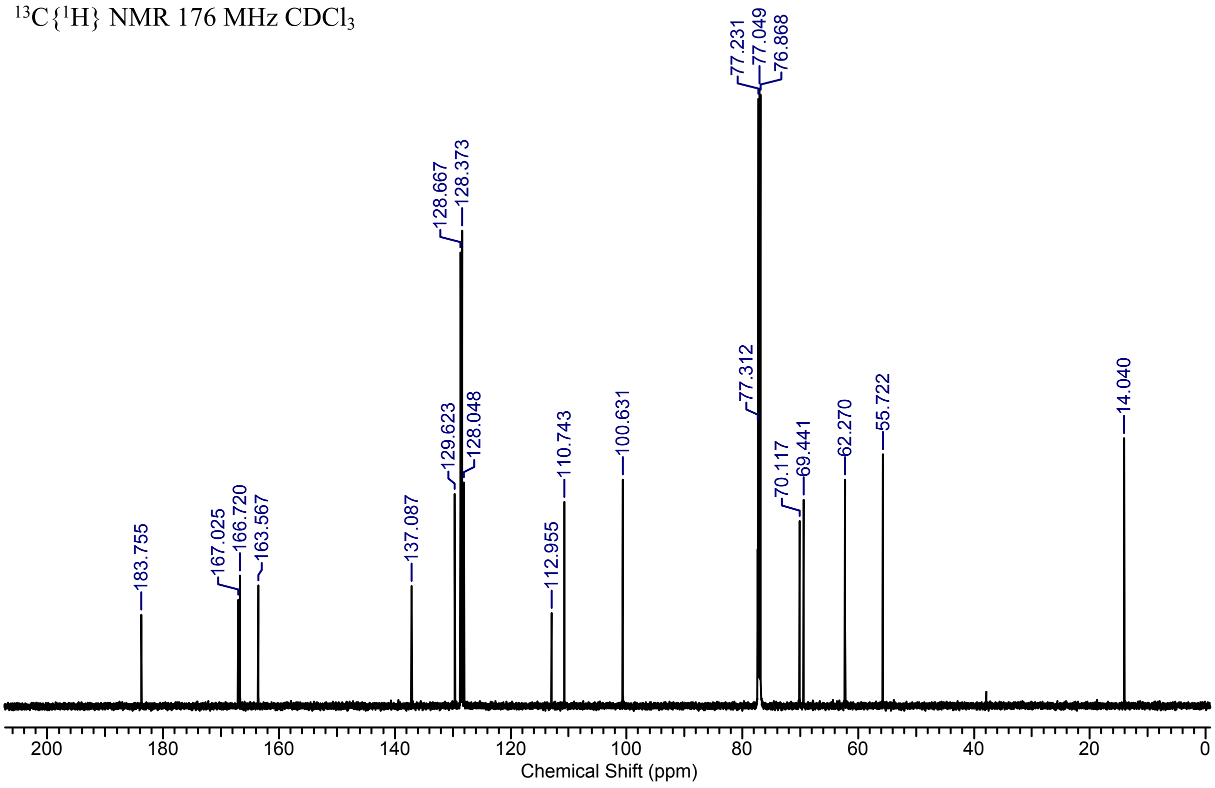


**Figure S113.** $^{13}C$ spectrum of compound **4b**.

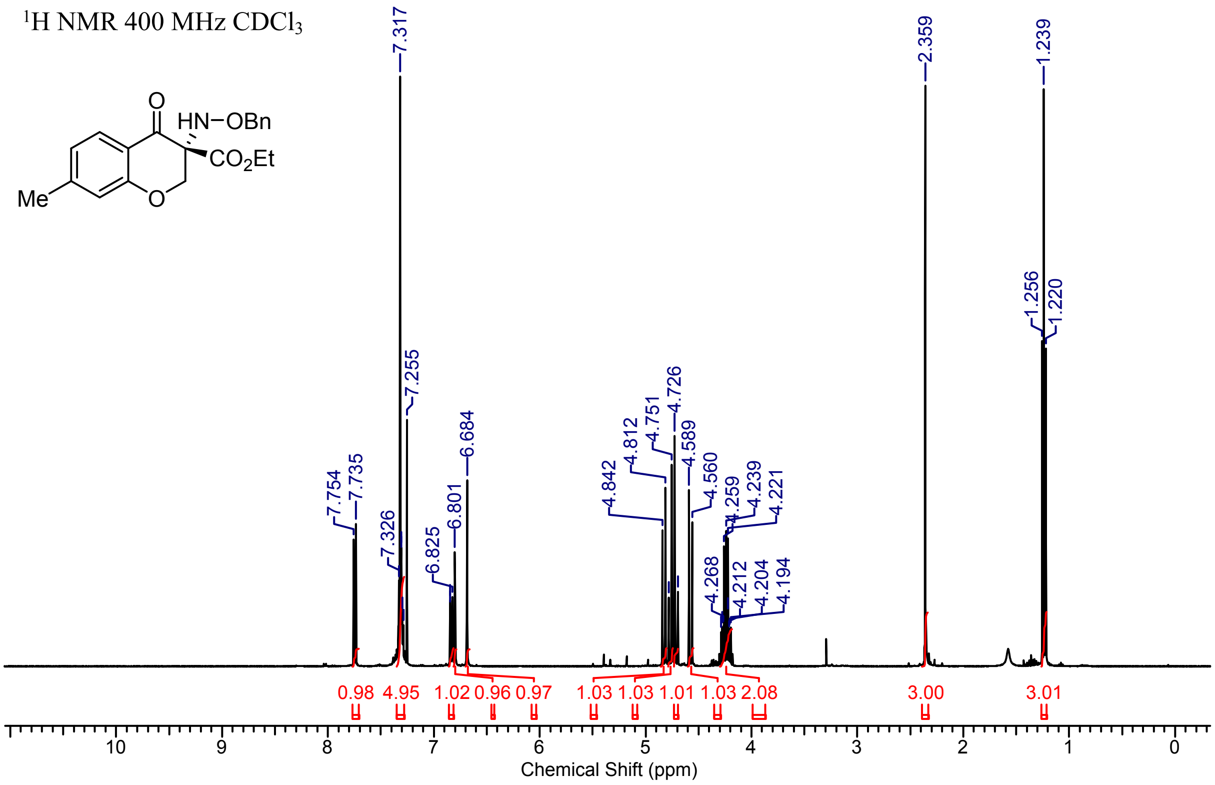


**Figure S114.** $^1$H spectrum of compound **4c**.

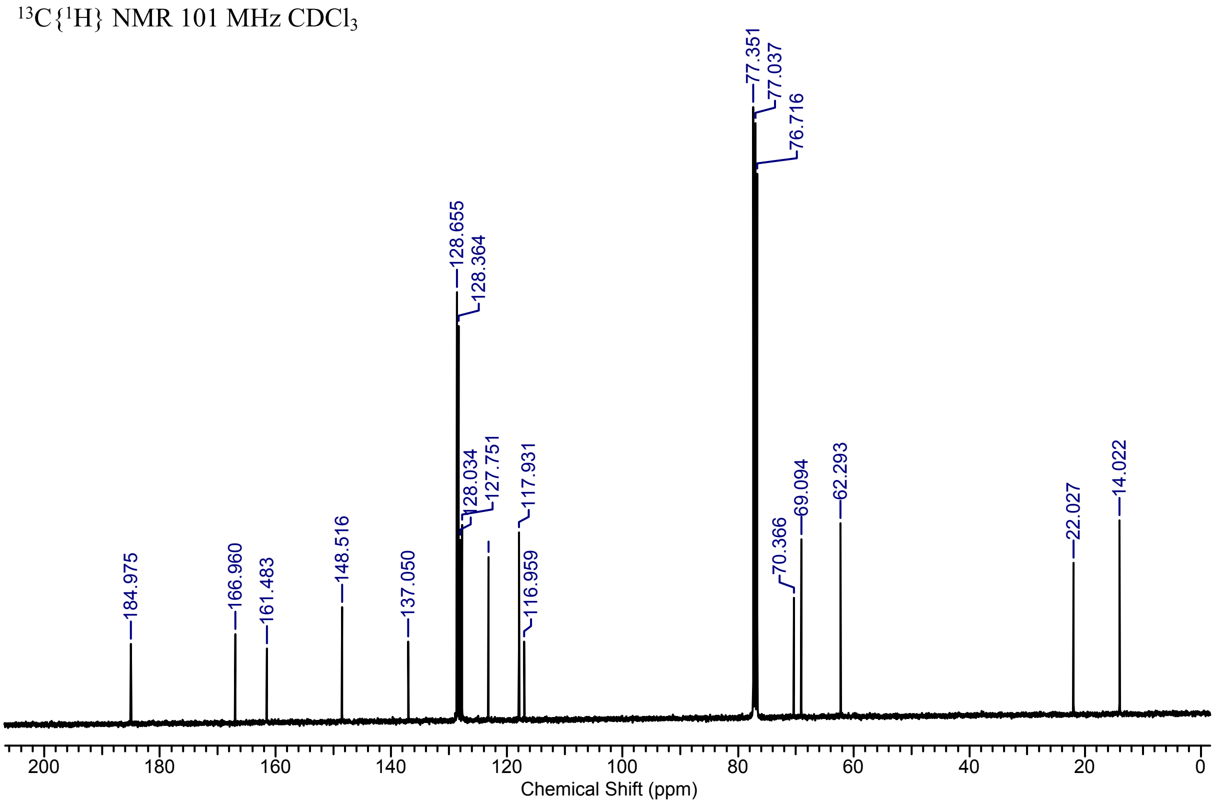


**Figure S115.** $^{13}$C spectrum of compound **4c**.

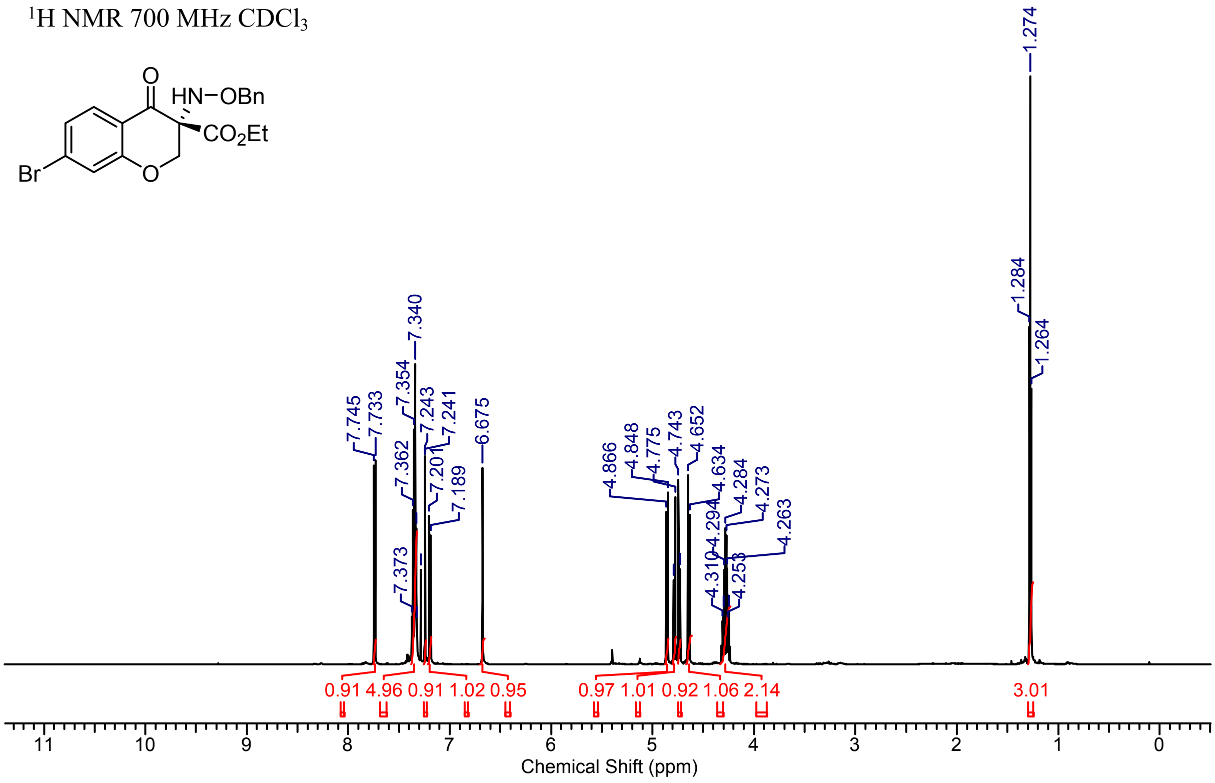


**Figure S116.** $^{1}$H spectrum of compound **4d**.

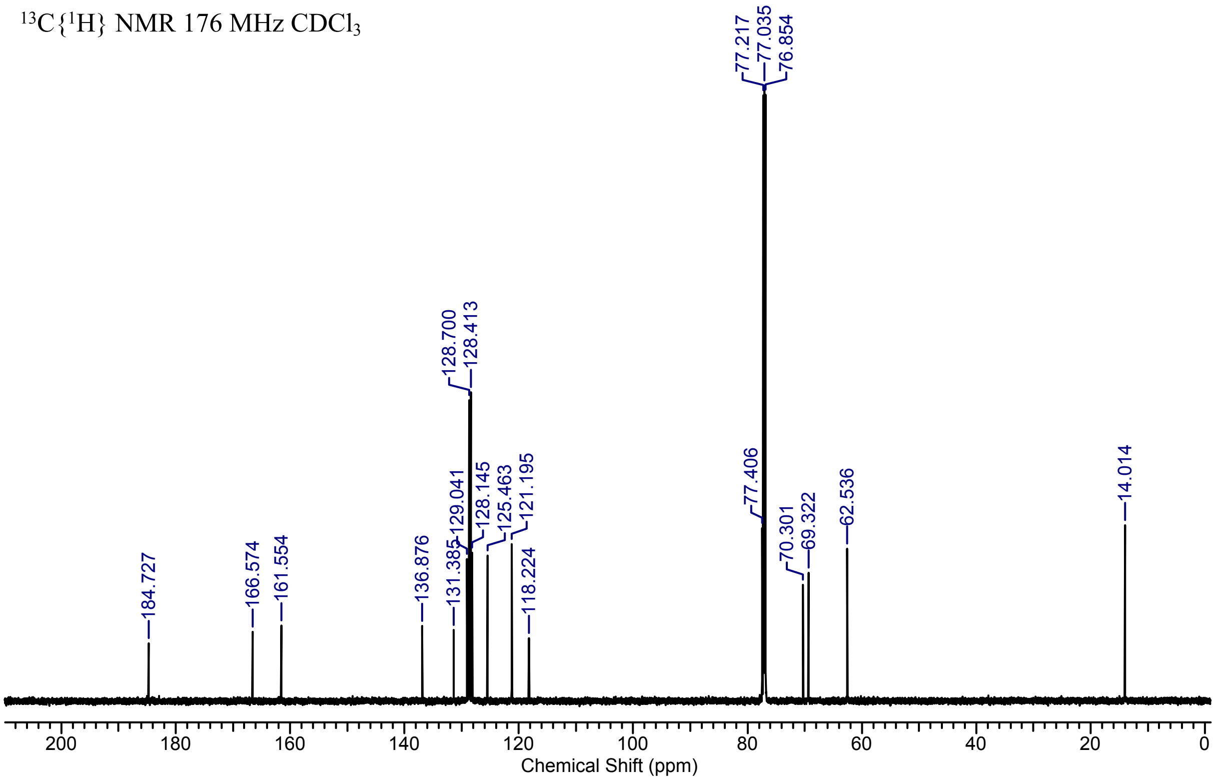


**Figure S117.** $^{13}$C spectrum of compound **4d**.

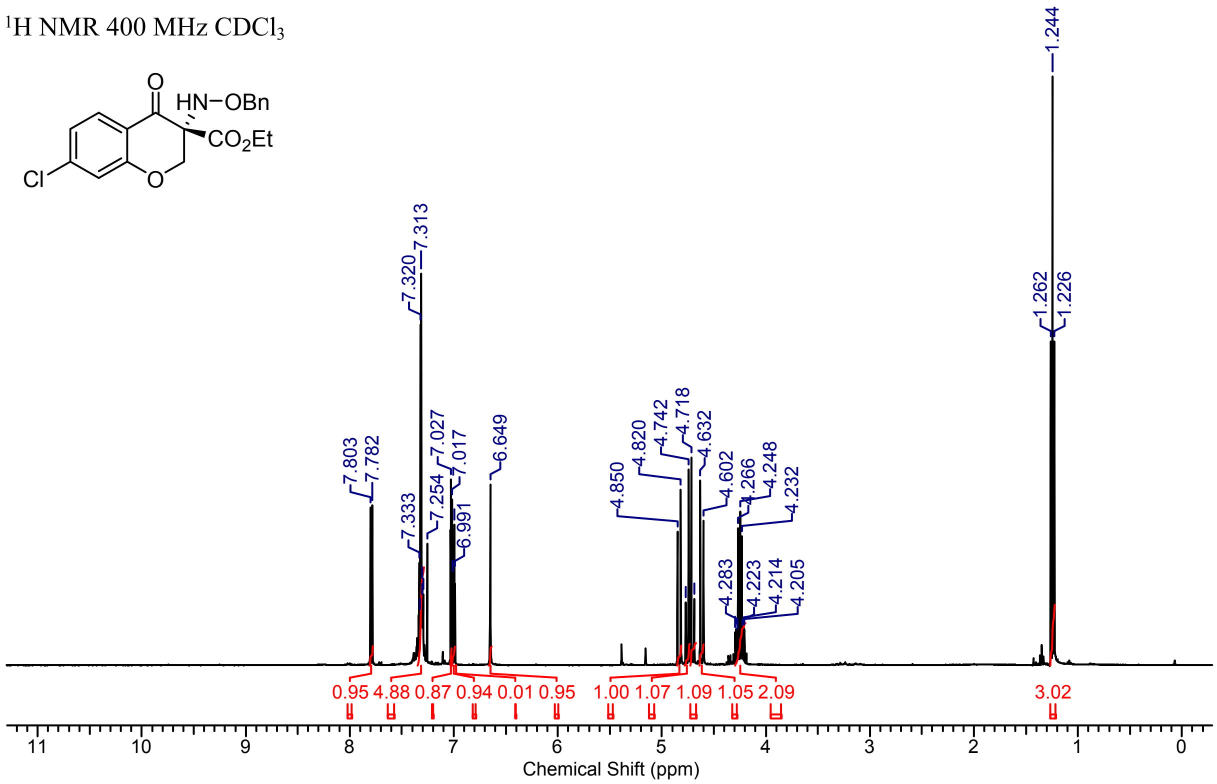


**Figure S118.** $^1$H spectrum of compound **4e**.

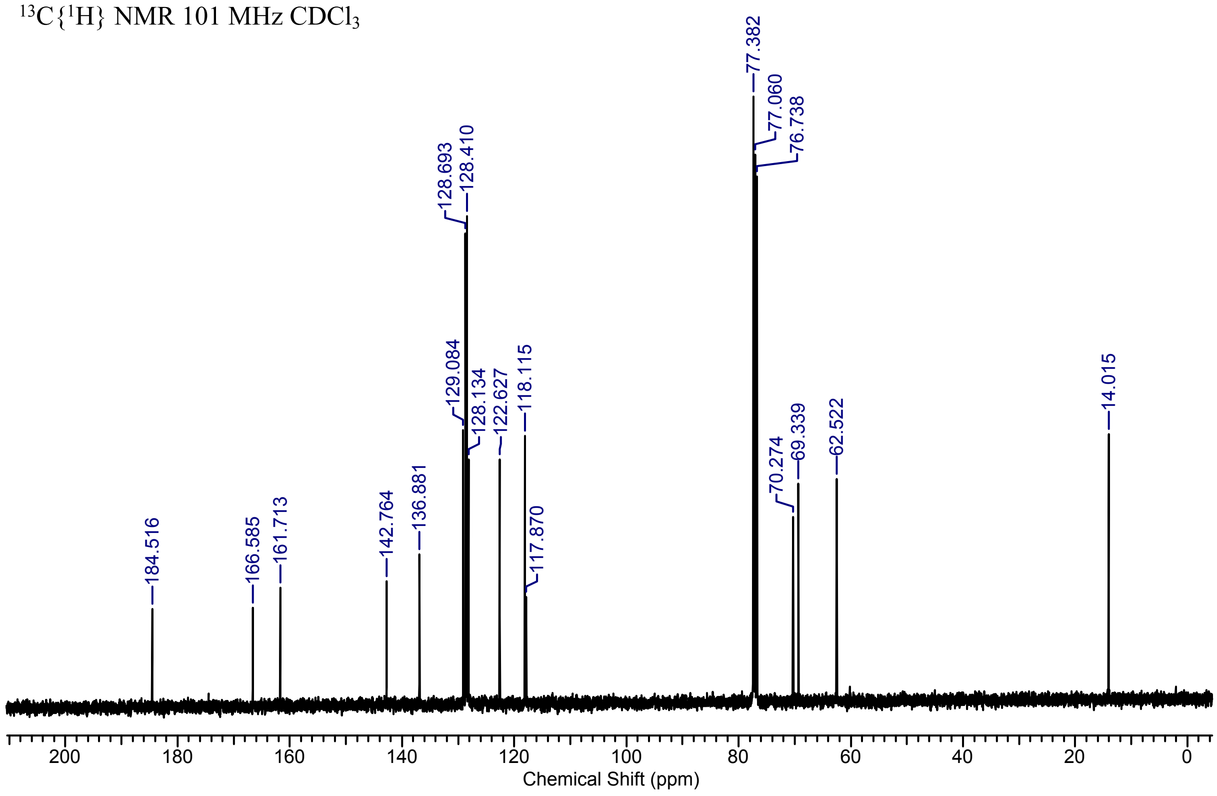


**Figure S119.** $^{13}$C spectrum of compound **4e**.

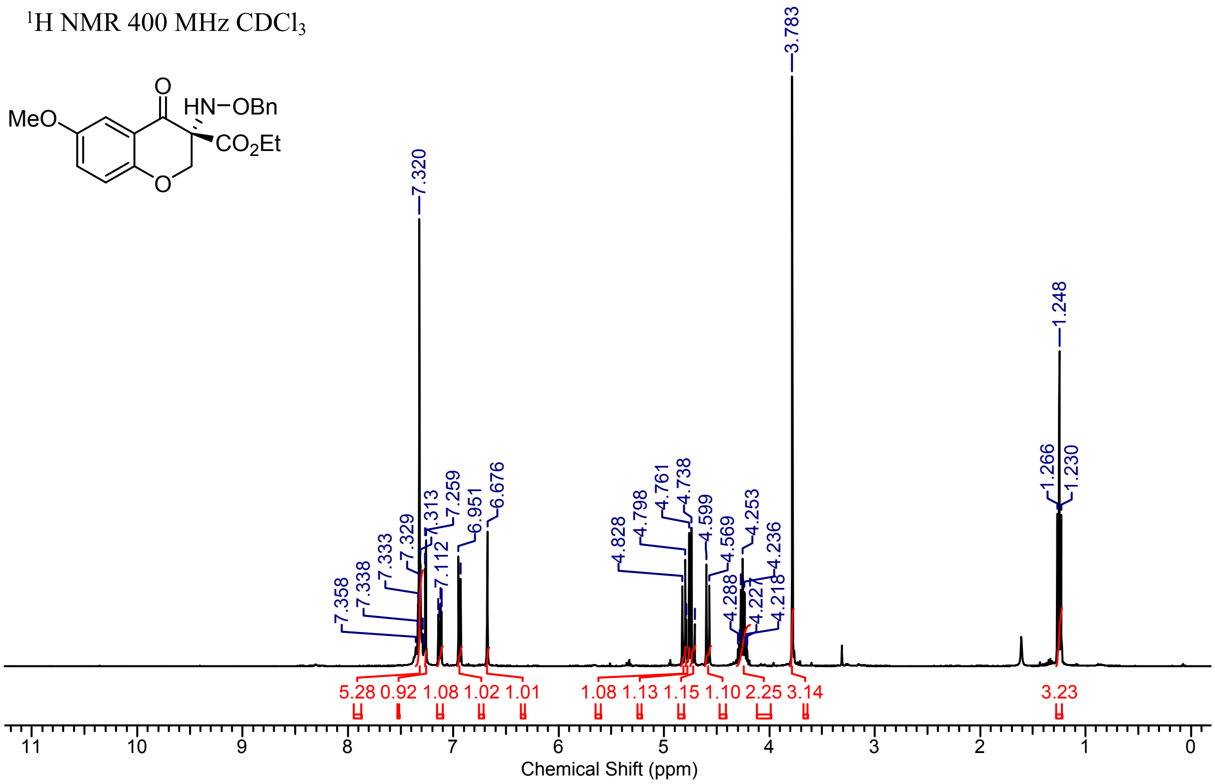


**Figure S120.** $^1$H spectrum of compound **4f**.

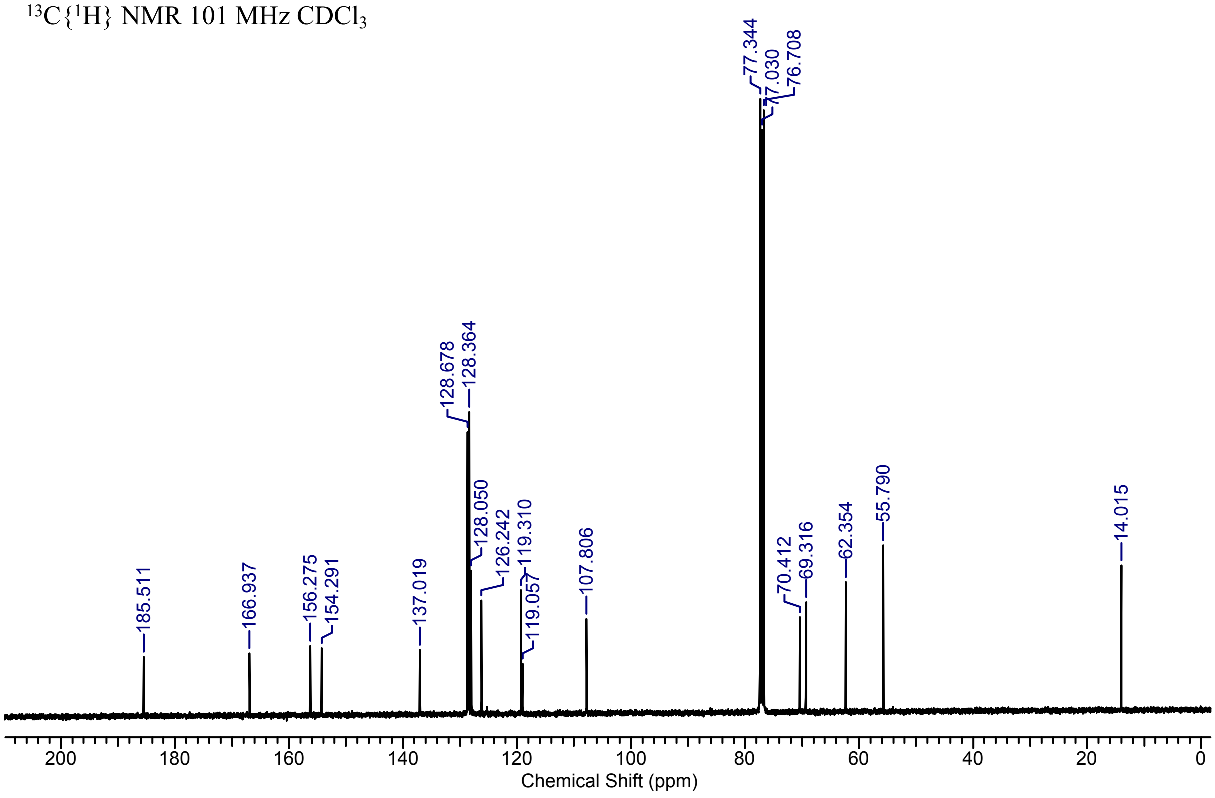


**Figure S121.** $^{13}$C spectrum of compound **4f**.

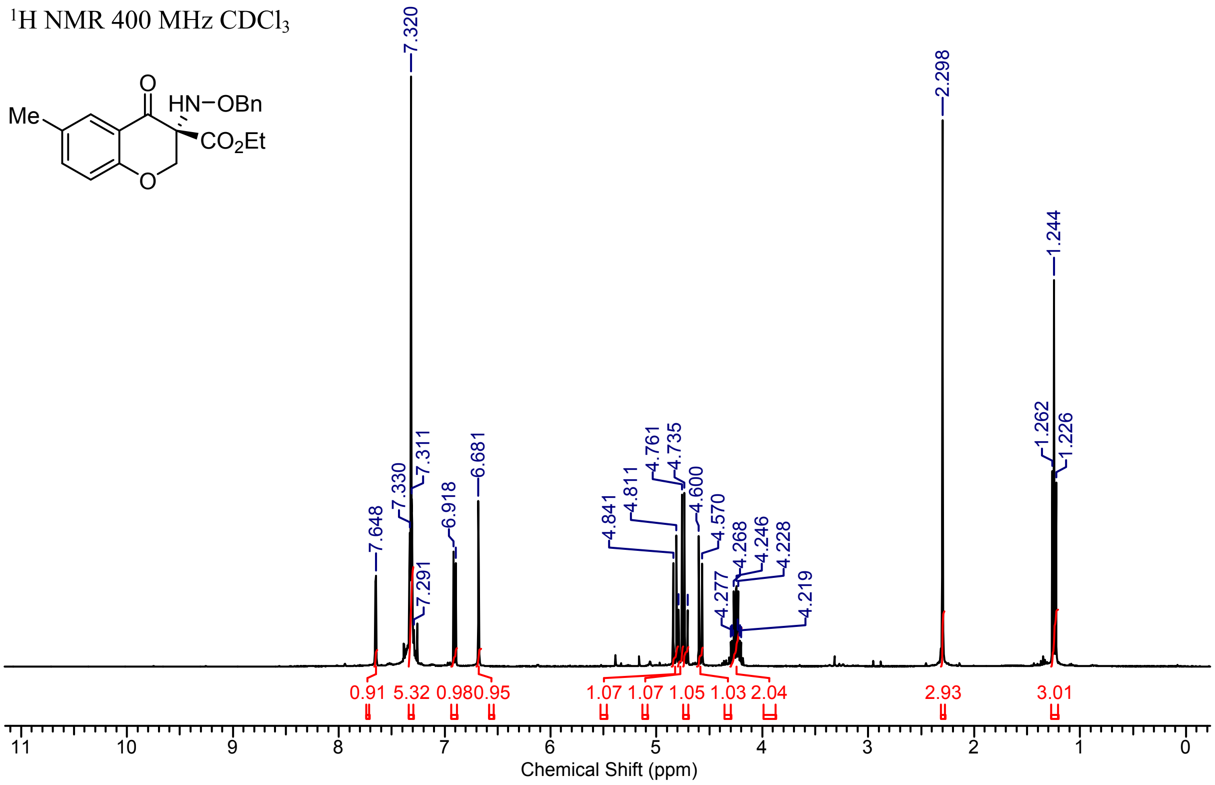


**Figure S122.** $^{1}H$ spectrum of compound **4g**.

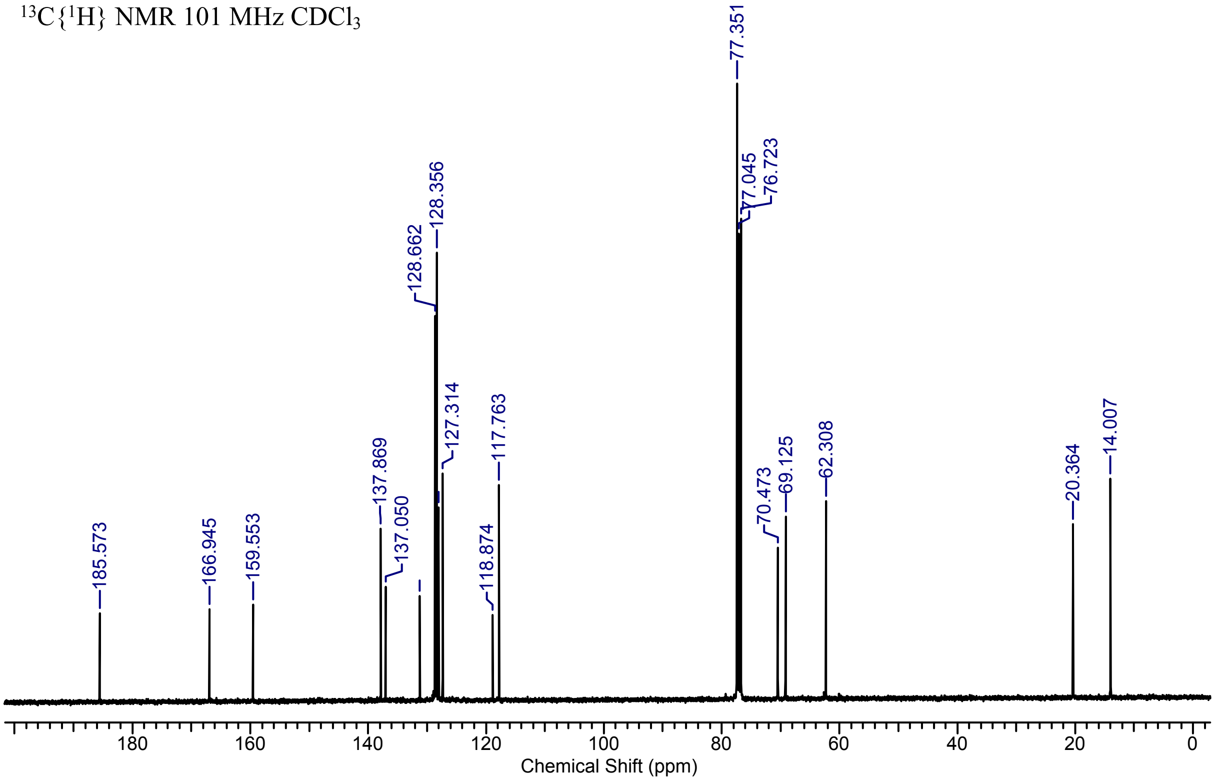


**Figure S123.** $^{13}C$ spectrum of compound **4g**.

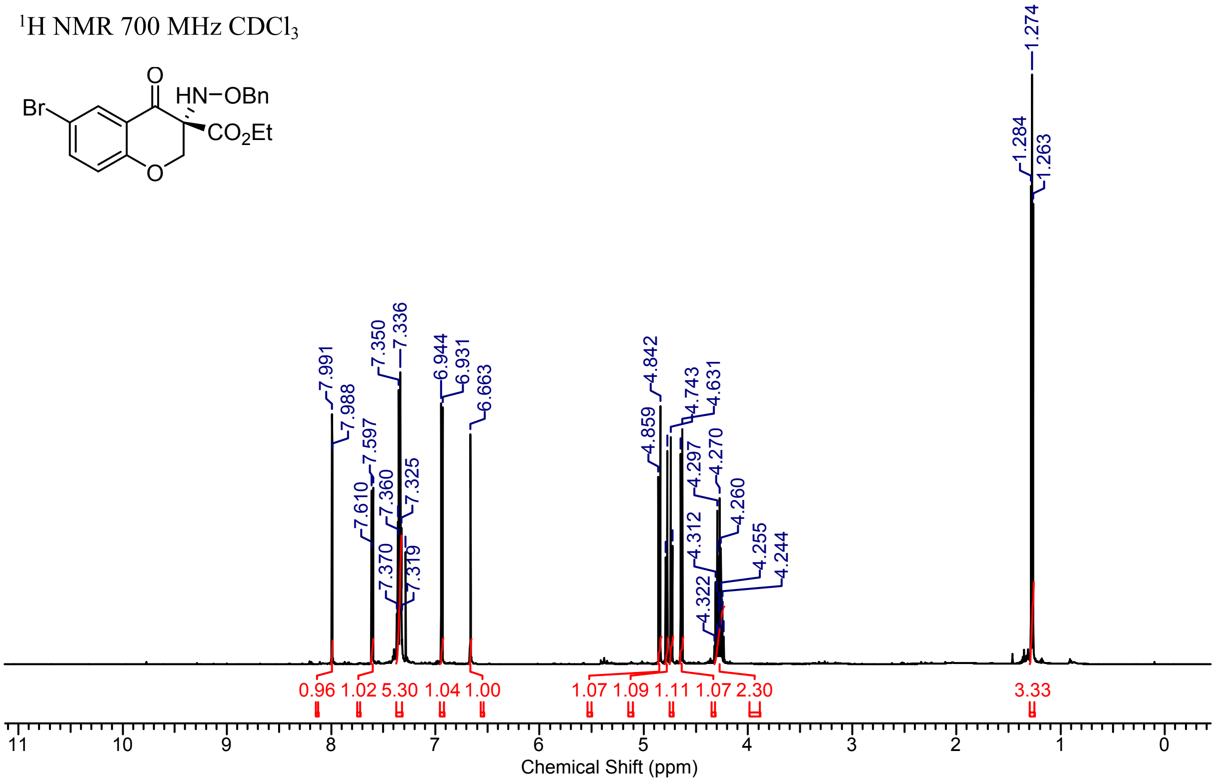


**Figure S124.** $^{1}H$ spectrum of compound **4h**.

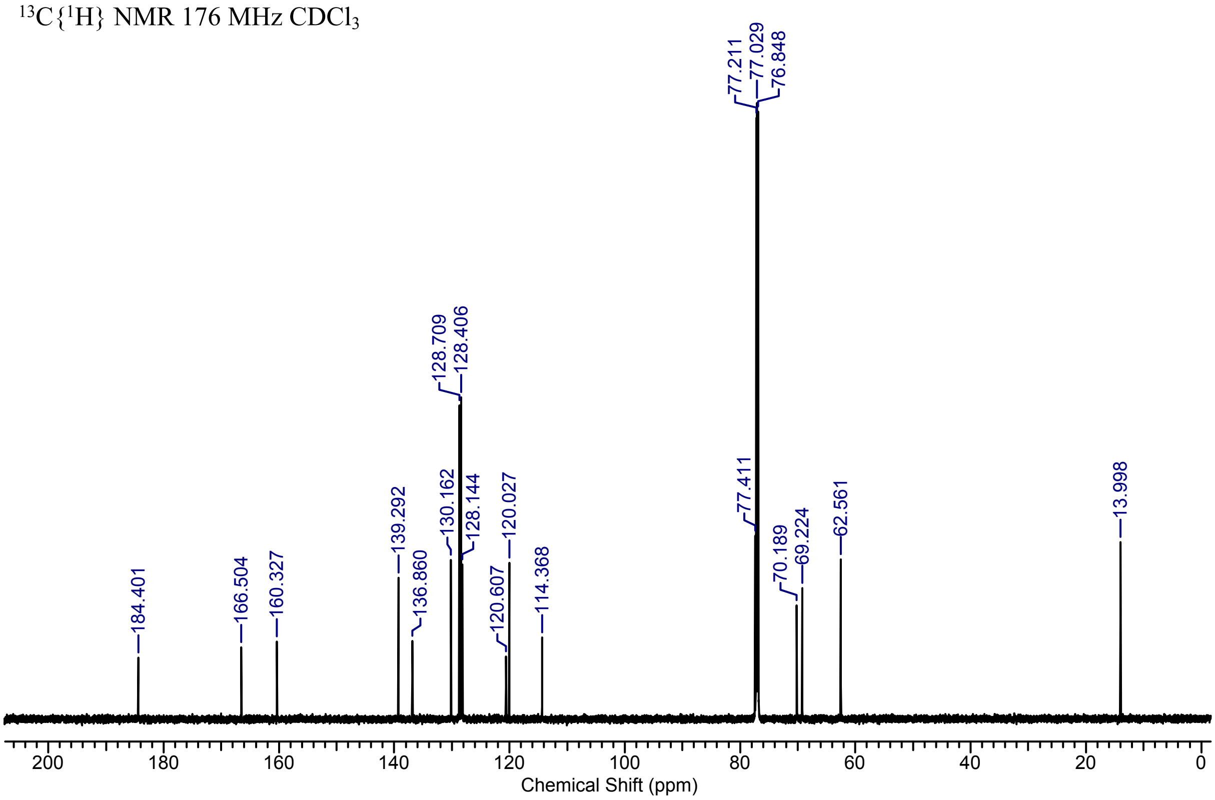


**Figure S125.** $^{13}C$ spectrum of compound **4h**.

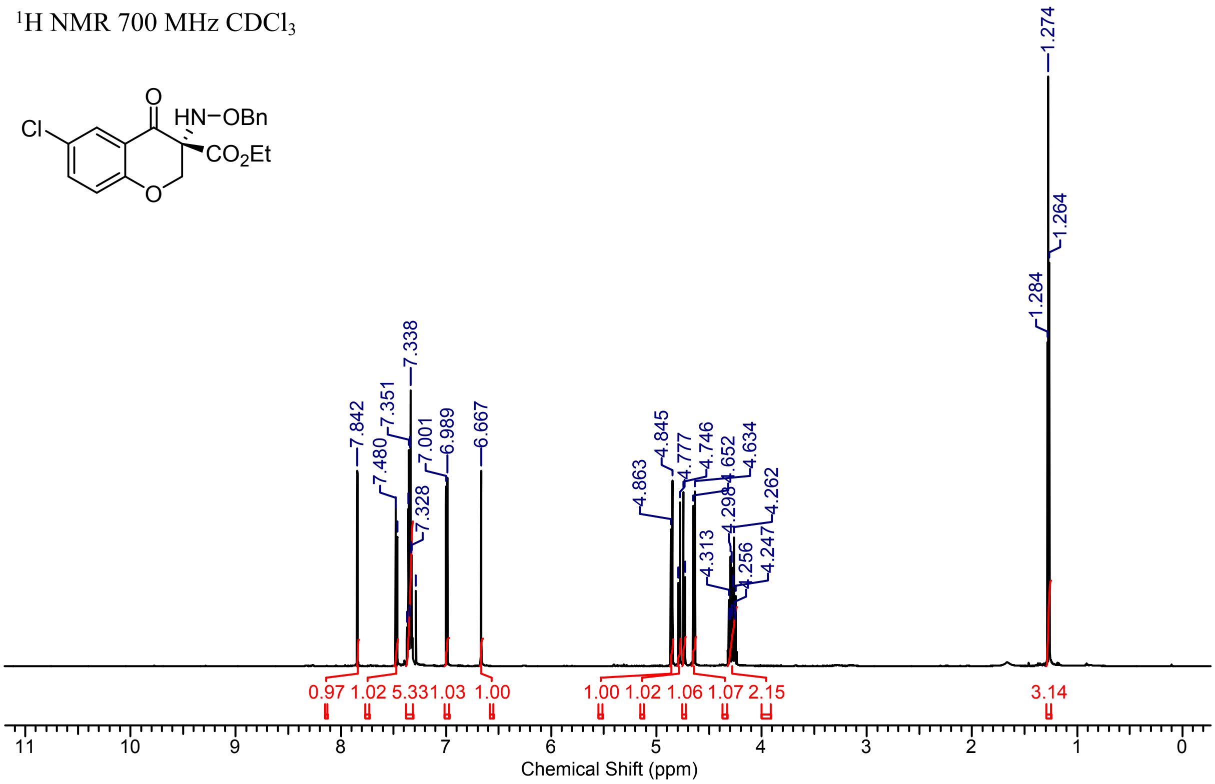


**Figure S126.** $^1$H spectrum of compound **4i**.

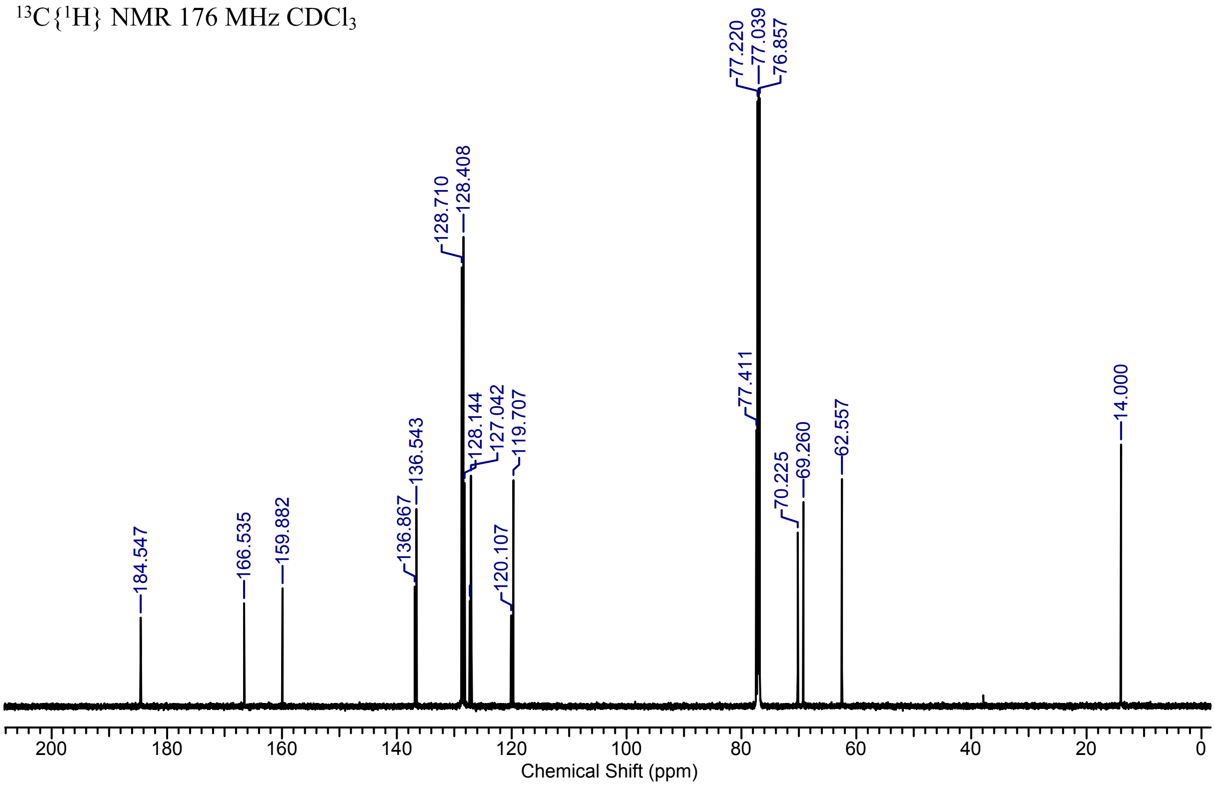


**Figure S127.** $^{13}$C spectrum of compound **4i**.

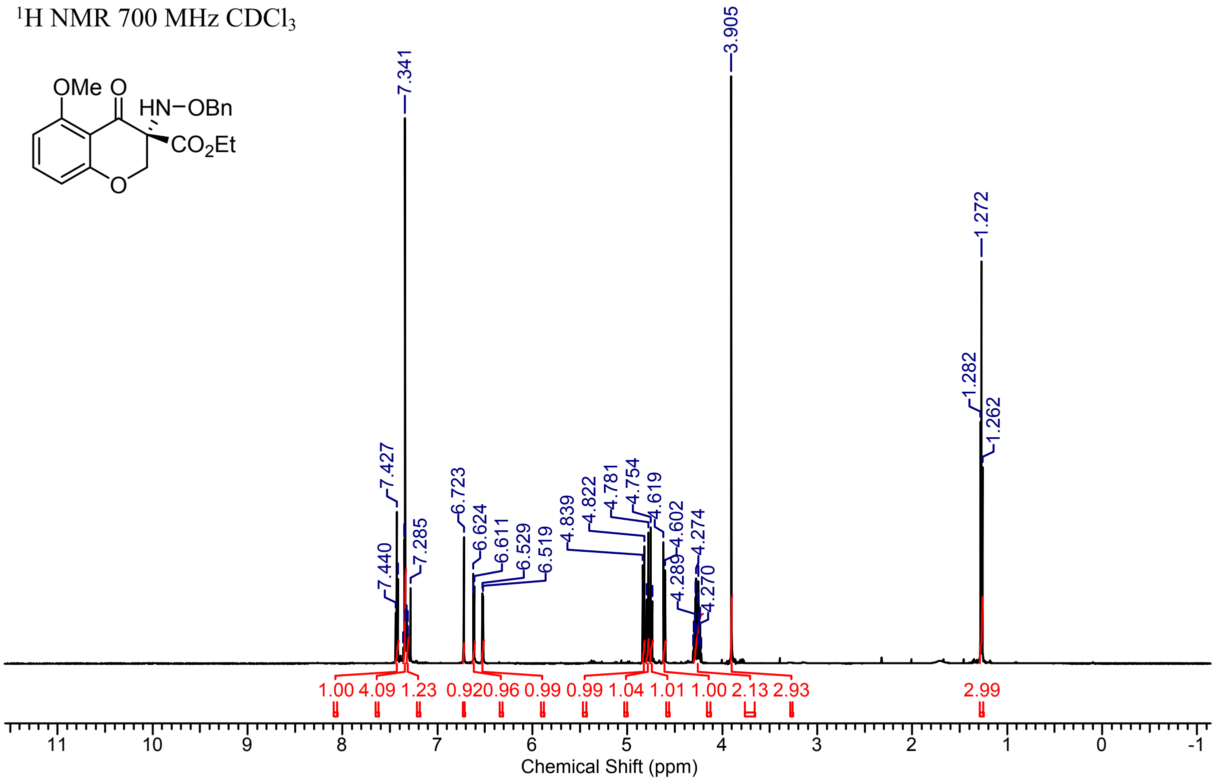


**Figure S128.** $^1$H spectrum of compound **4j**.

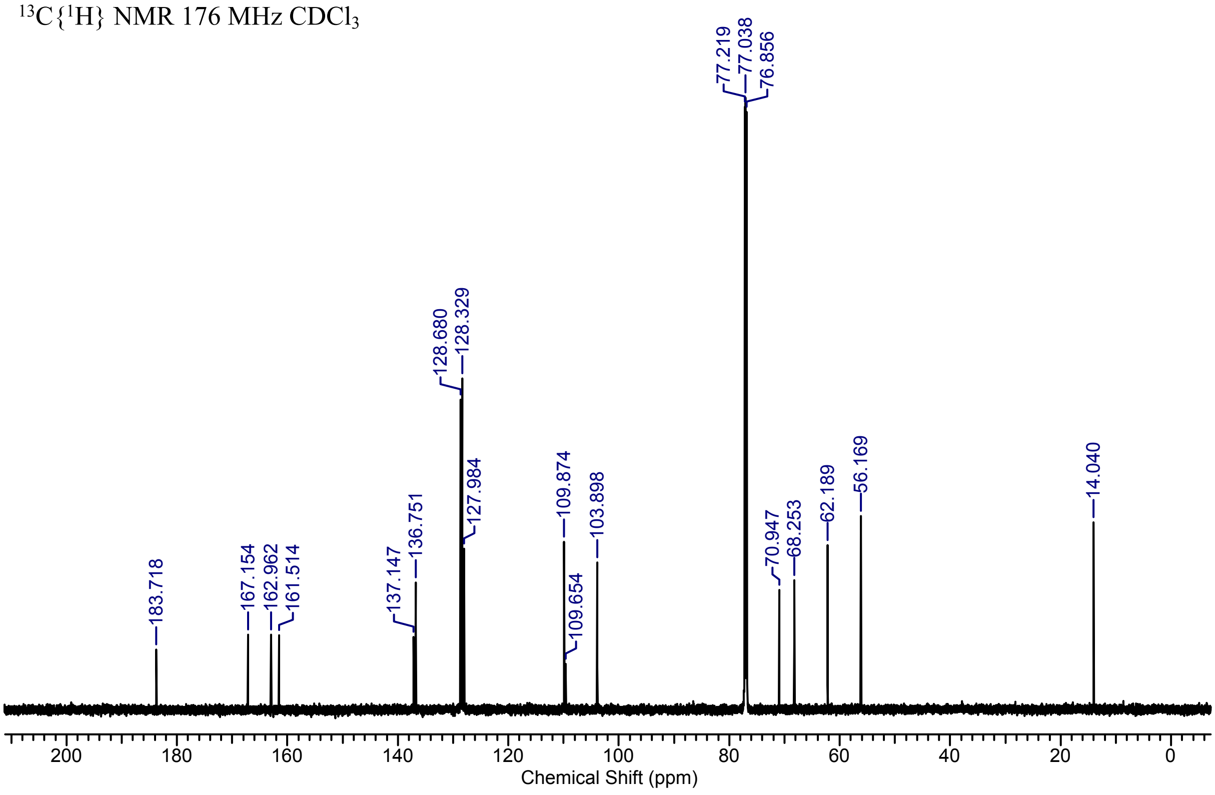


**Figure S129.** $^{13}$C spectrum of compound **4j**.

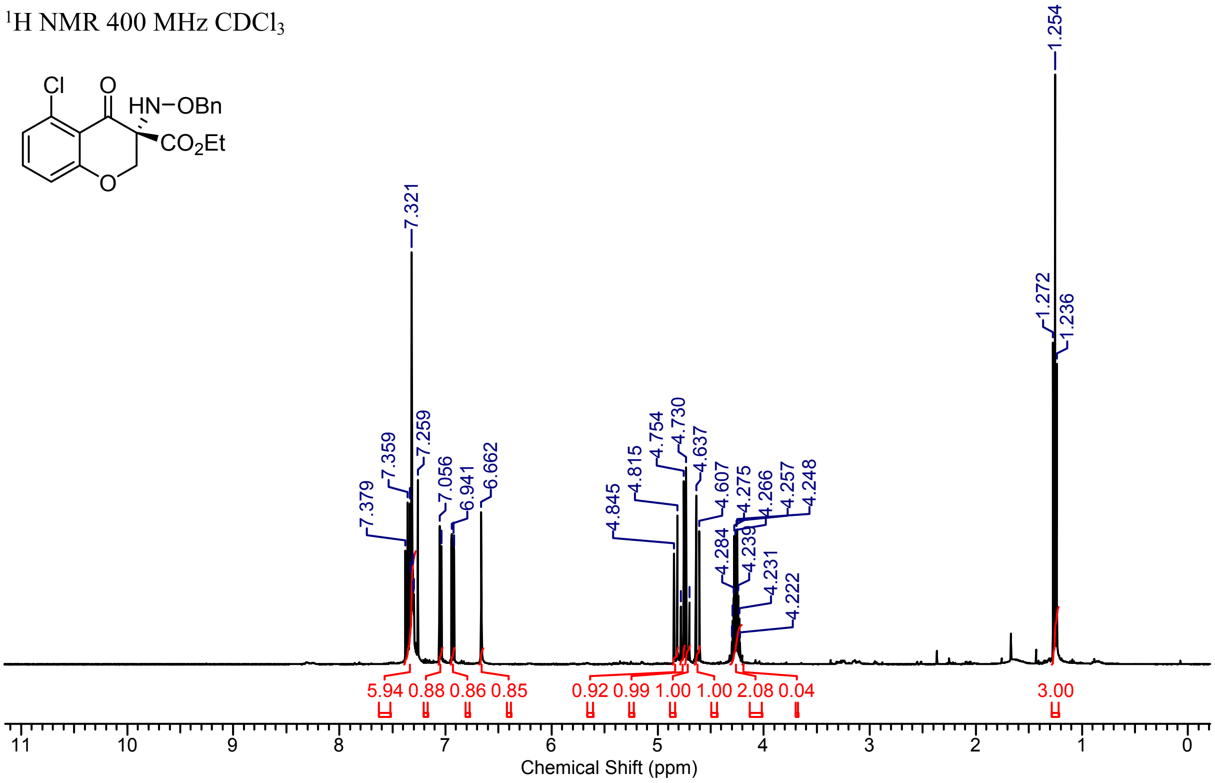


**Figure S130.** $^1$H spectrum of compound **4k**.

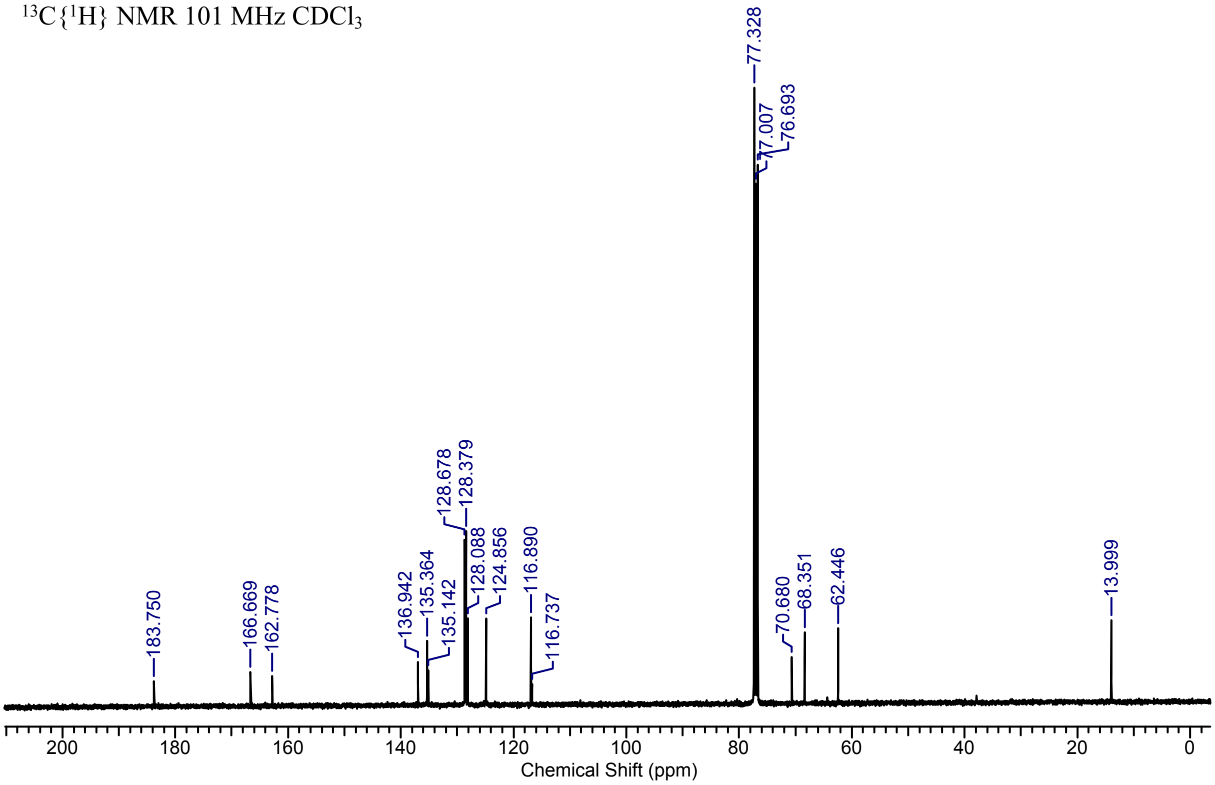


**Figure S131.** $^{13}$C spectrum of compound **4k**.

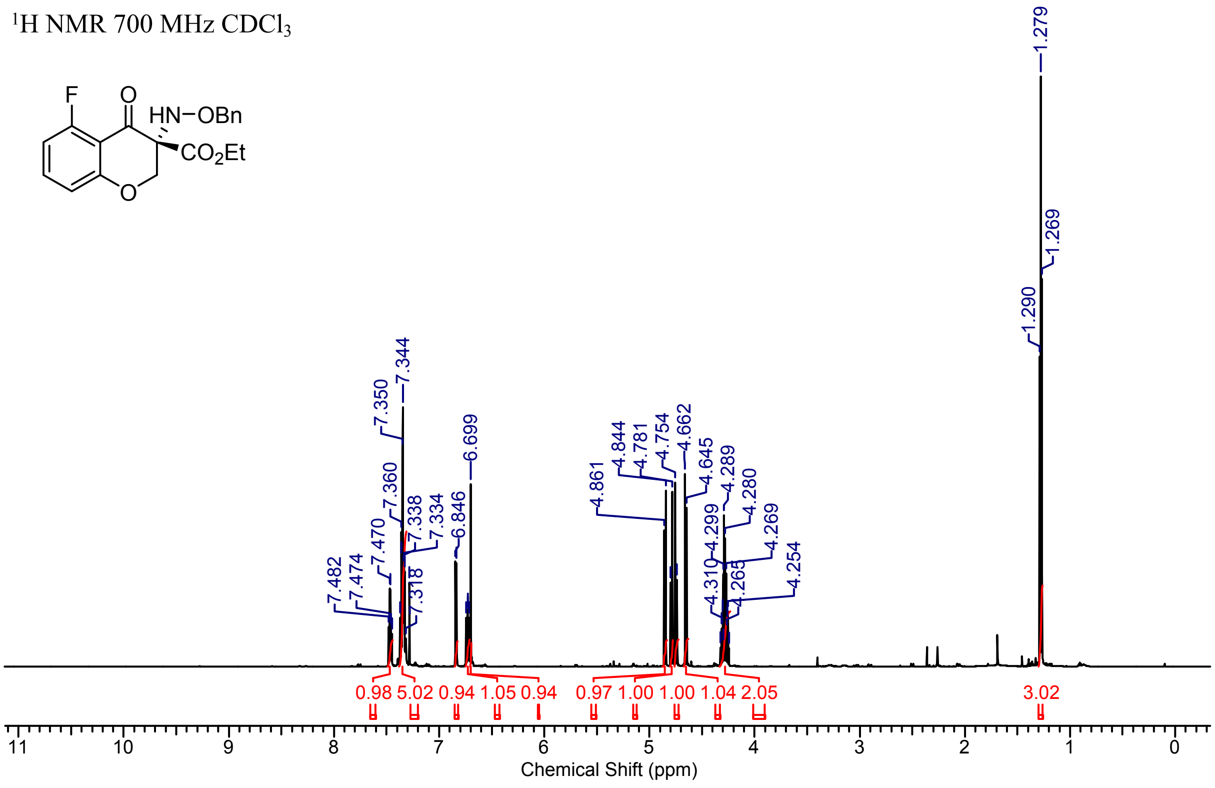


**Figure S132.** $^{1}H$ spectrum of compound **4l**.

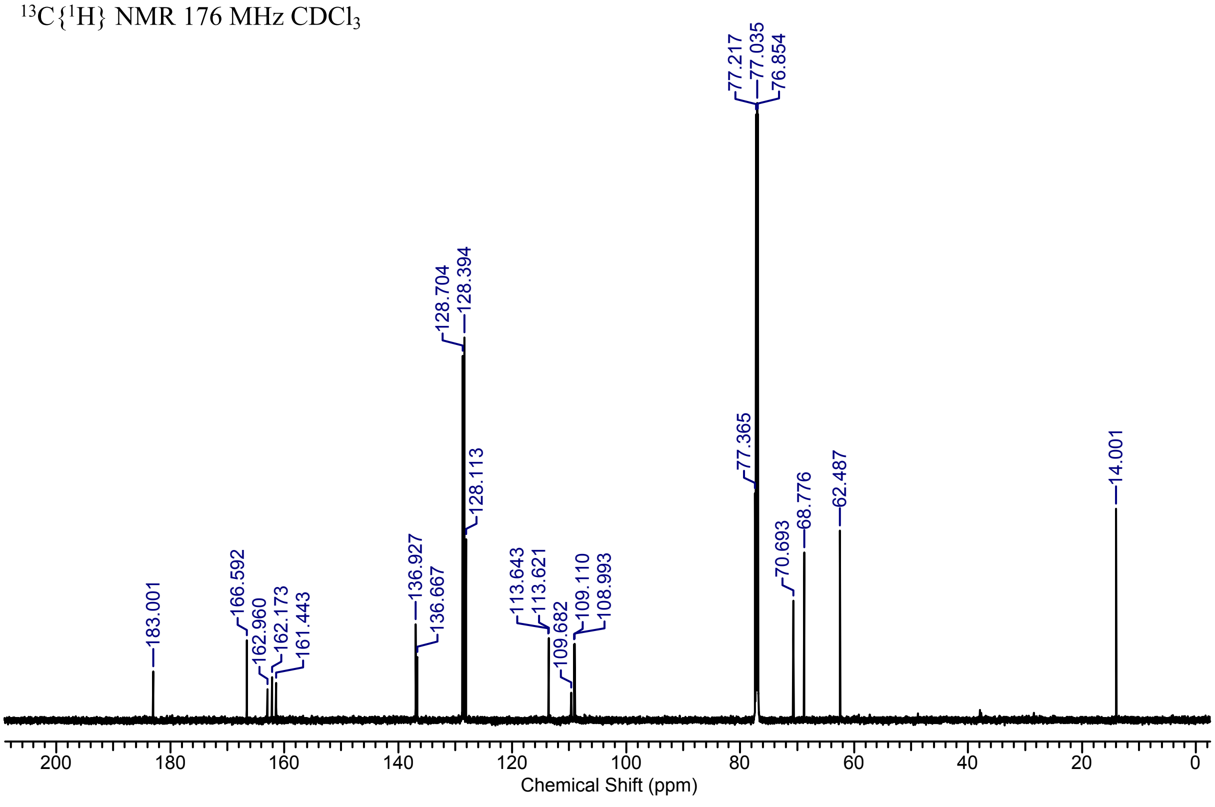


**Figure S133.** $^{13}C$ spectrum of compound **4l**.

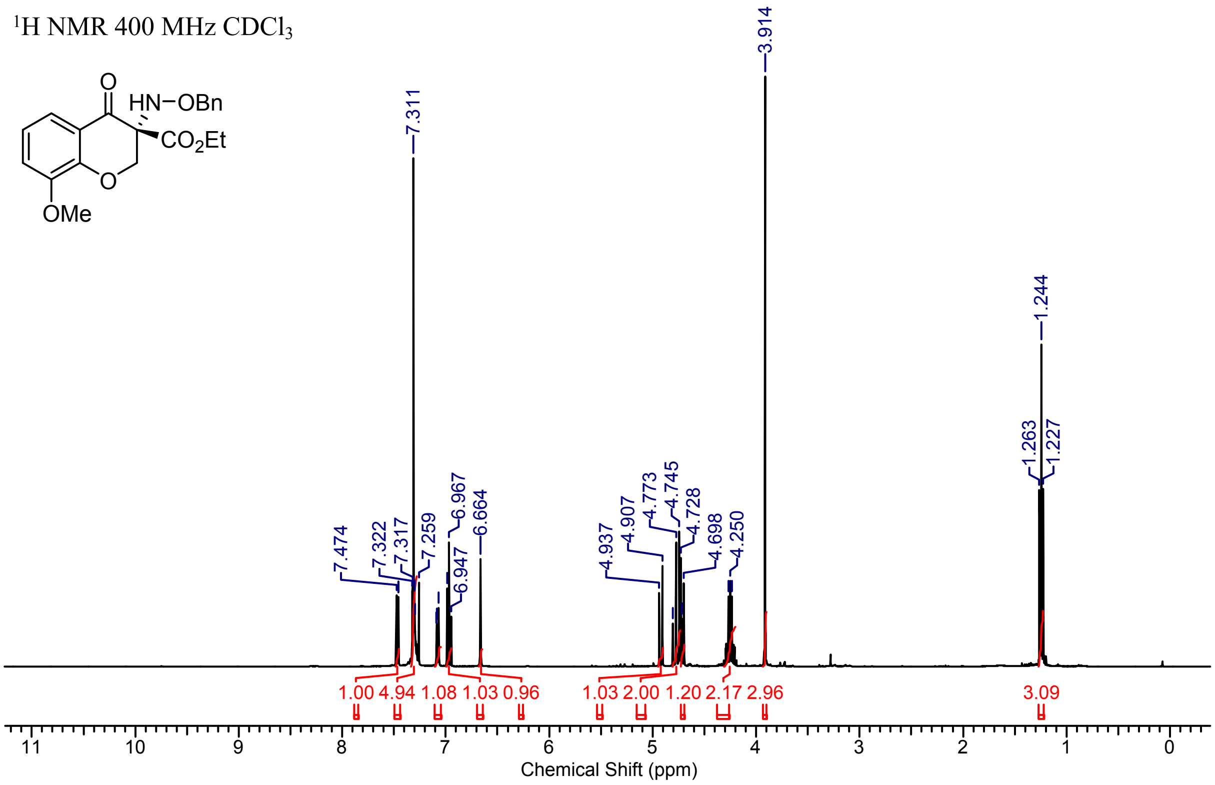


**Figure S134.** $^{1}H$ spectrum of compound **4m**.

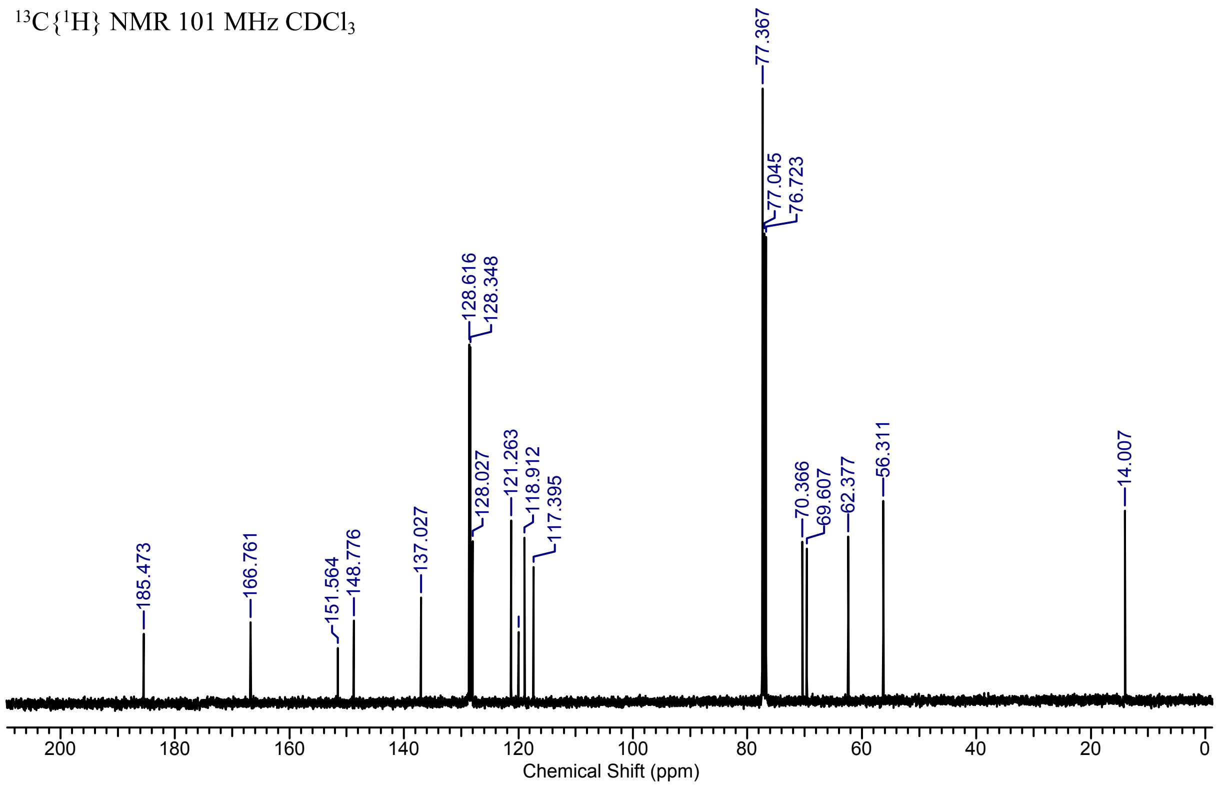


**Figure S135.** $^{13}C$ spectrum of compound **4m**.

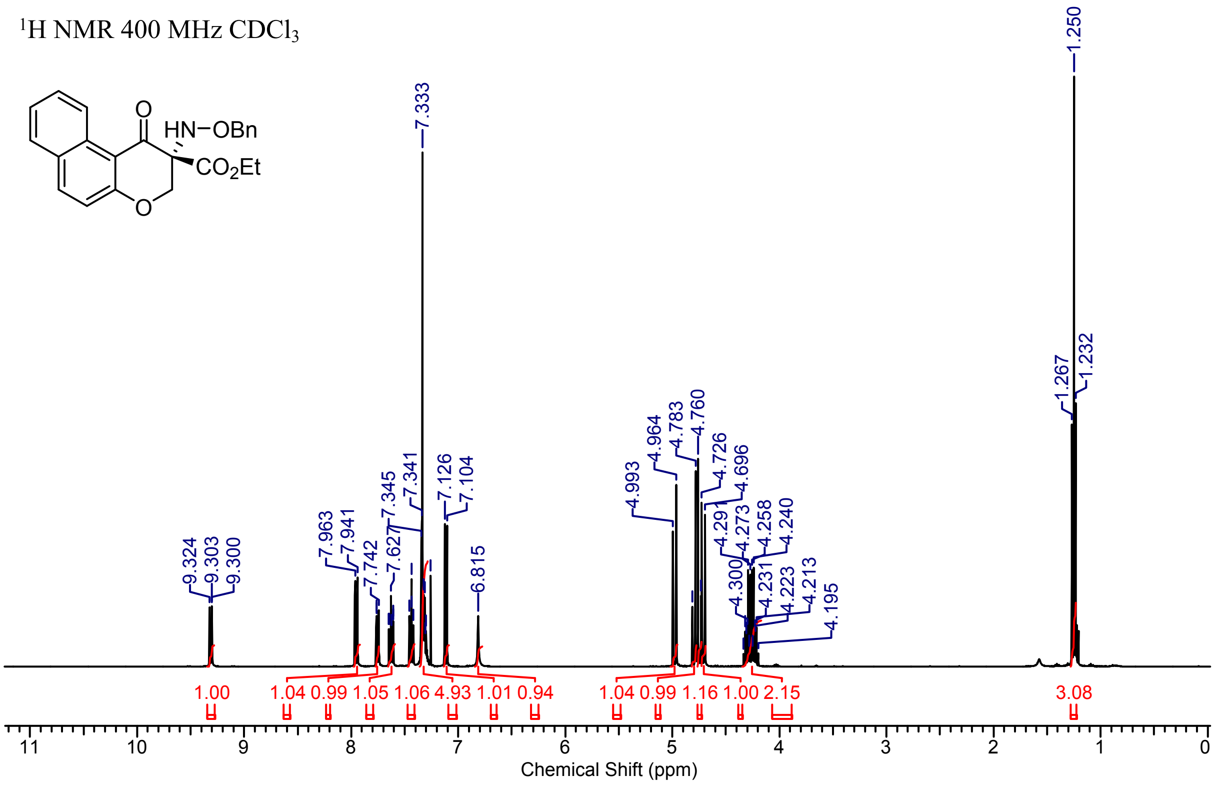


**Figure S136.** $^{1}H$ spectrum of compound **4n**.

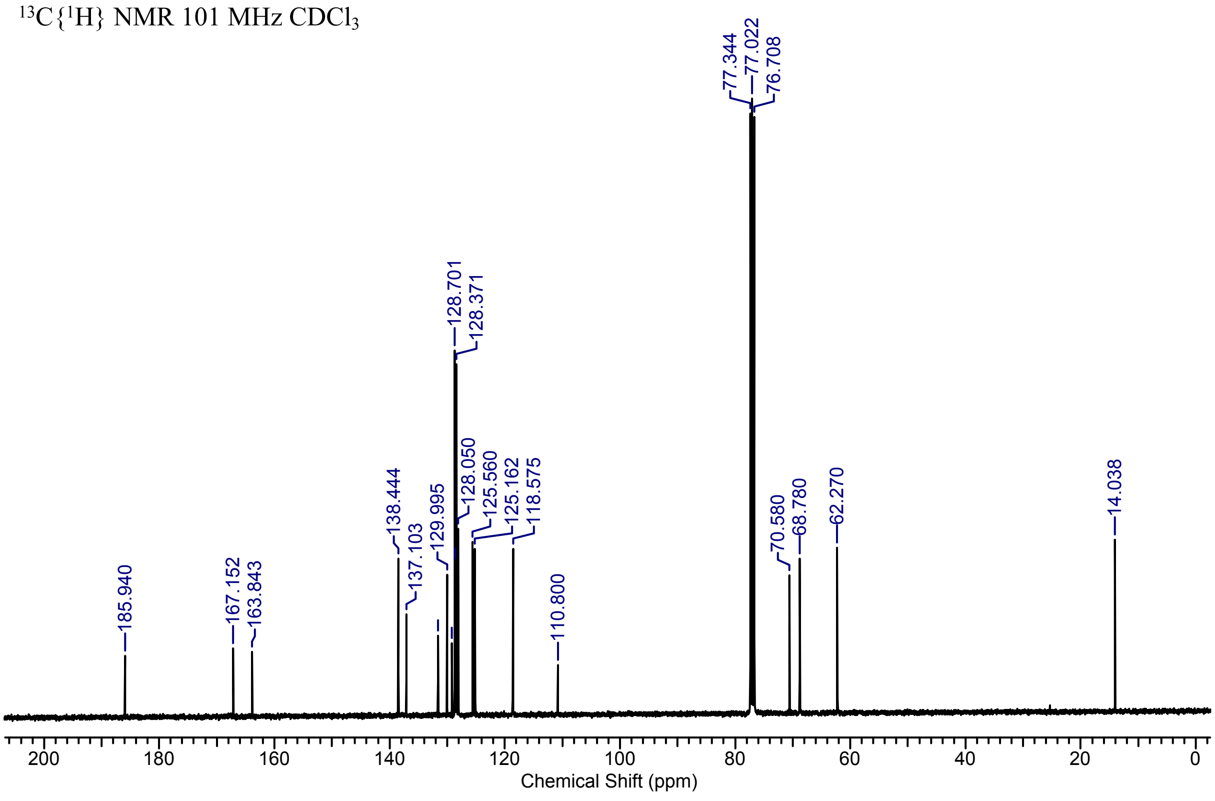


**Figure S137.** $^{13}C$ spectrum of compound **4n**.

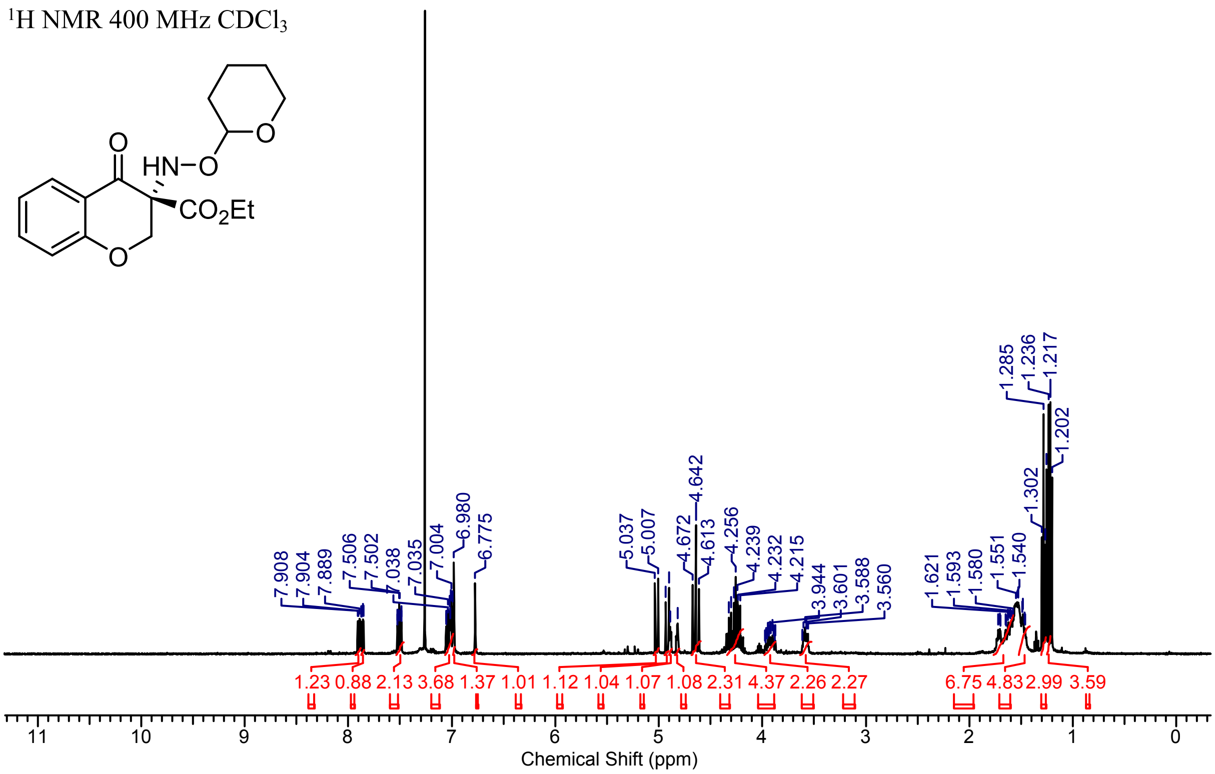


**Figure S138.** $^{1}H$ spectrum of compound **4o**.

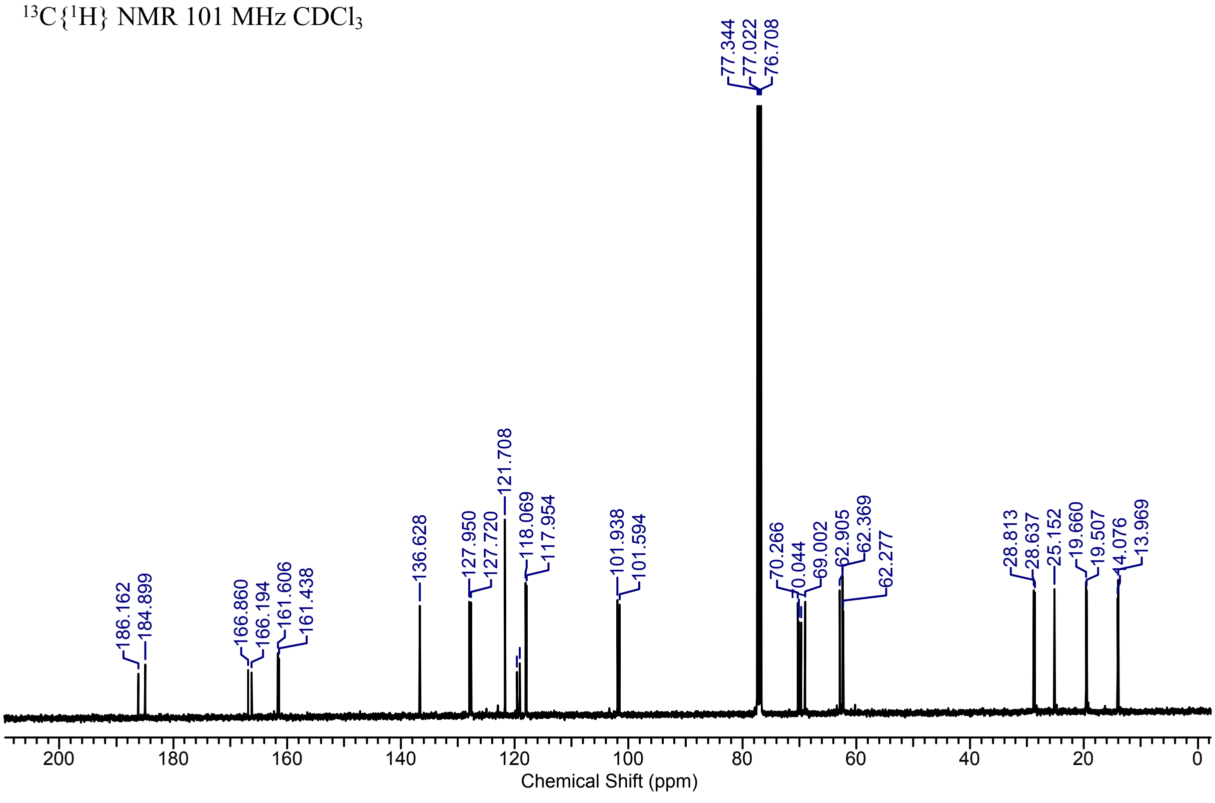


**Figure S139.** $^{13}C$ spectrum of compound **4o**.

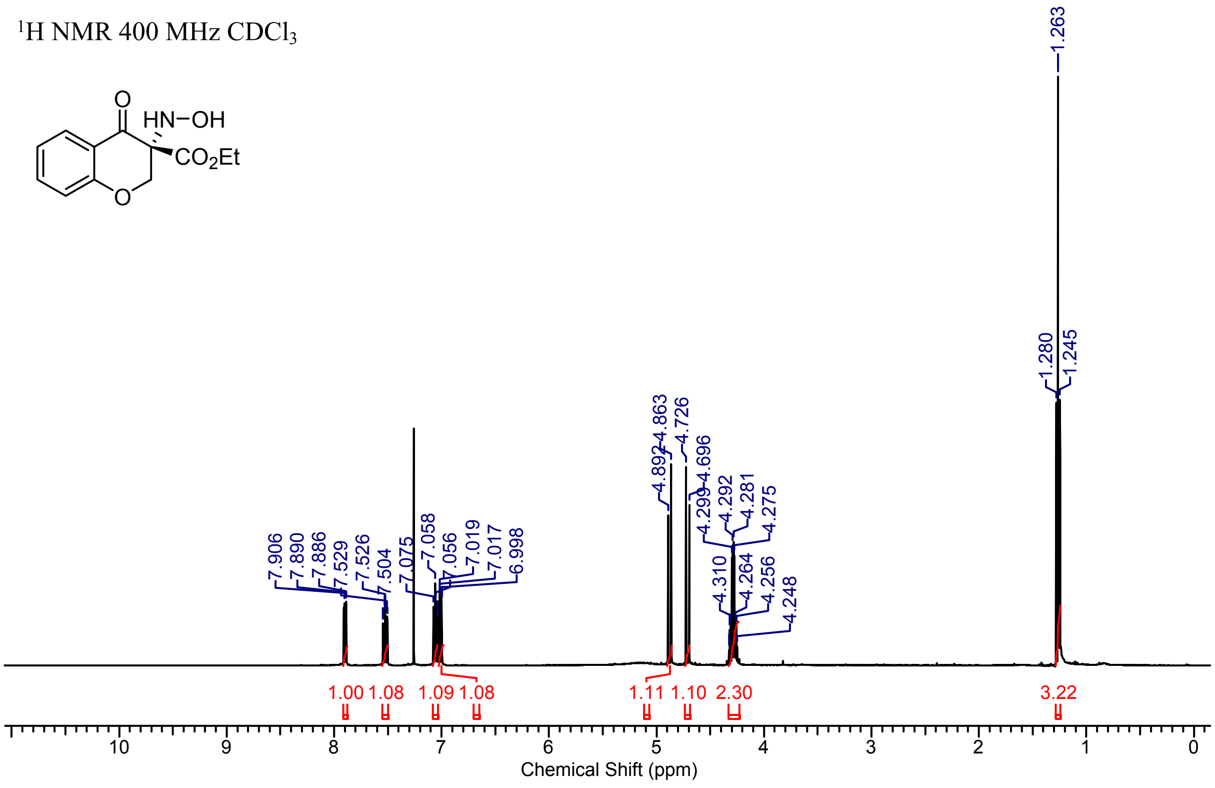


**Figure S140.** $^1$H spectrum of compound **4p**.

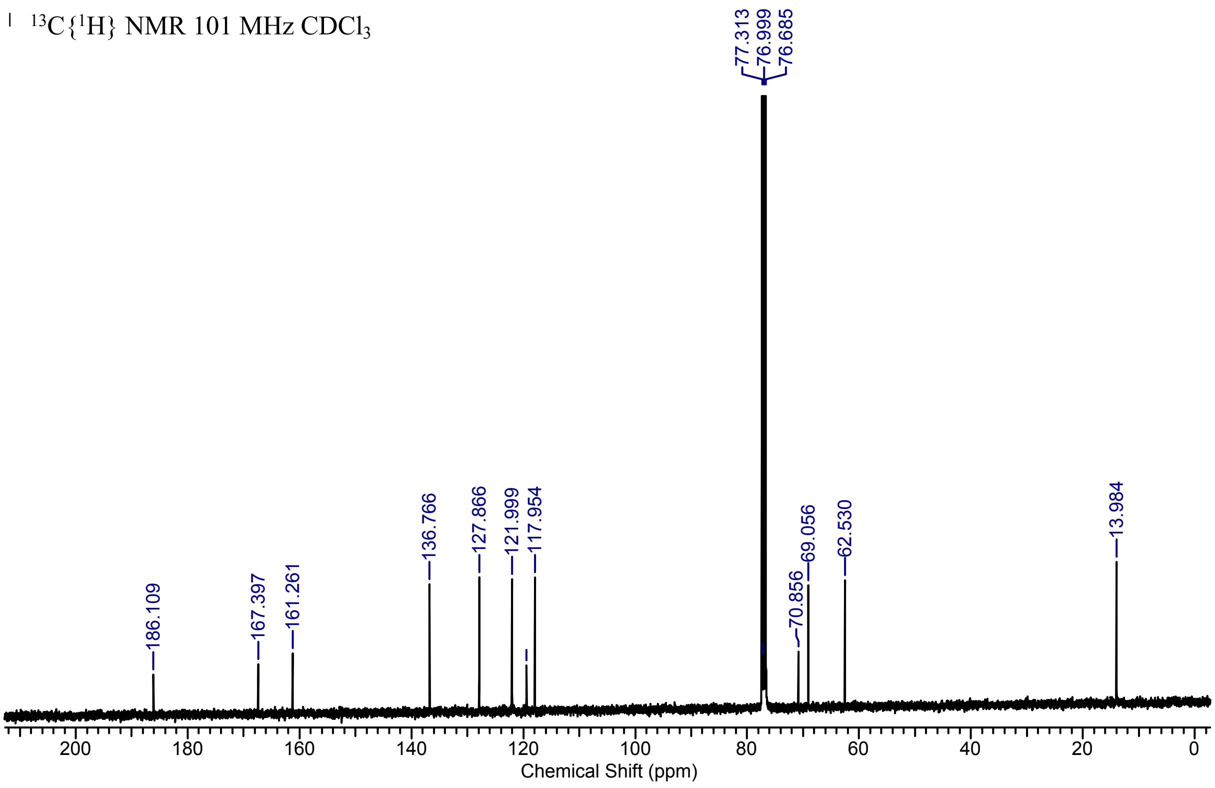


**Figure S141.** $^{13}$C spectrum of compound **4p**.

## 6. HPLC Chromatograms

```
Data File:  C:\CHEM32\1\DATA\IB\RAC010225.D
Sample Name:  IBD-380-RAC
Sample Info:  Phenomenex Lux Amylose-1, 3 um, 90:10, 1.0 mL/min, CHIR
```

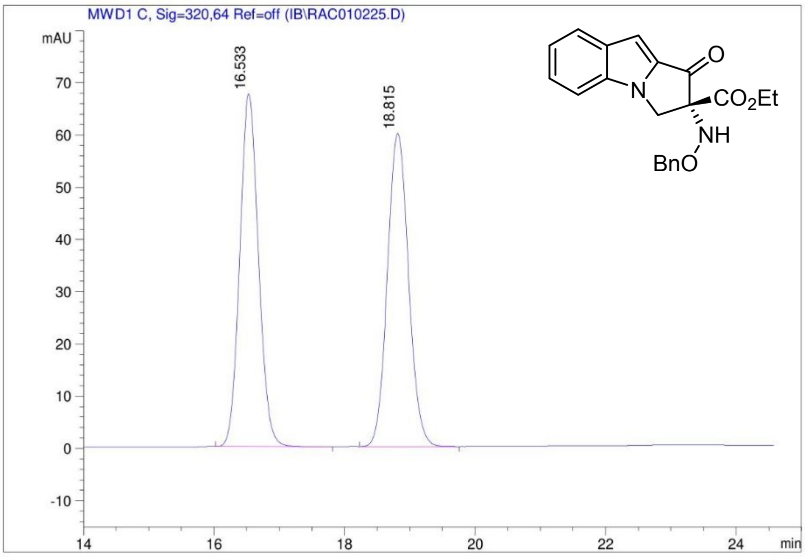


| # | Meas. R | Area | Area % |
|---|---|---|---|
| 1 | 16.533 | 1.348e3 | 49.966 |
| 2 | 18.815 | 1.349e3 | 50.034 |

```
Data File:  C:\CHEM32\1\DATA\IB\RAC010224.D
Sample Name:  IBD-379
Sample Info:  Phenomenex Lux Amylose-1, 3 um, 90:10, 1.0 mL/min, CHIR
```

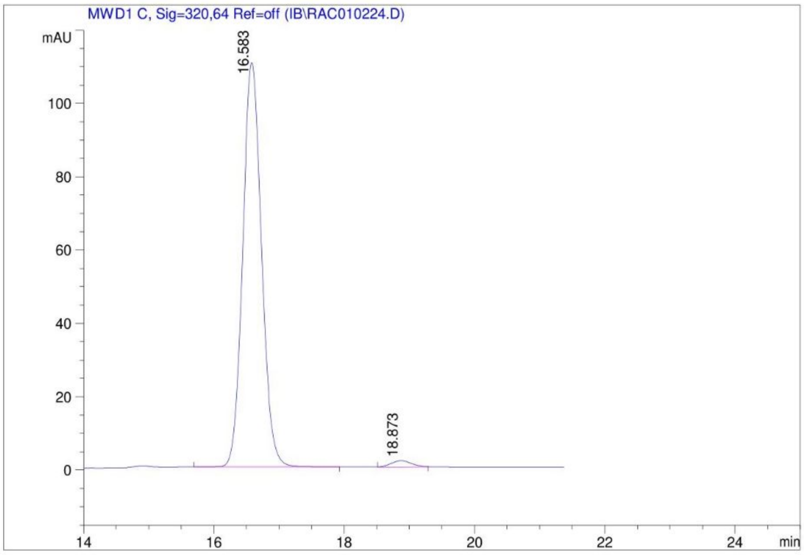


| # | Meas. R | Area | Area % |
|---|---|---|---|
| 1 | 16.583 | 2.211e3 | 98.368 |
| 2 | 18.873 | 36.683 | 1.632 |

**Figure S142.** HPLC chromatograms of **2a** (racemic – top, enantioenriched – bottom).

```
Data File:  C:\CHEM32\1\DATA\IB\RAC000223.D
Sample Name:  IBD-434-rac
Sample Info:  Phenomenex Lux Amylose-1, 3 um, 90:10, 1.0 mL/min, rac,
               p=93 bar; T=25st.C
```

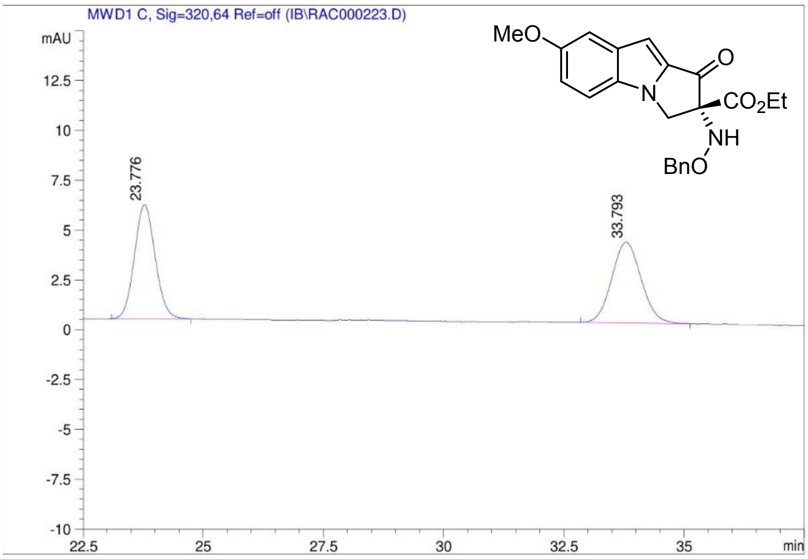


| # | Meas. R | Area | Area % |
|---|---|---|---|
| 1 | 23.776 | 171.771 | 49.906 |
| 2 | 33.793 | 172.417 | 50.094 |

```
Data File:  C:\CHEM32\1\DATA\IB\CHIR000224.D
Sample Name:  IBD-434-ch
Sample Info:  Phenomenex Lux Amylose-1, 3 um, 90:10, 1.0 mL/min, rac,
               p=93 bar; T=25st.C
```

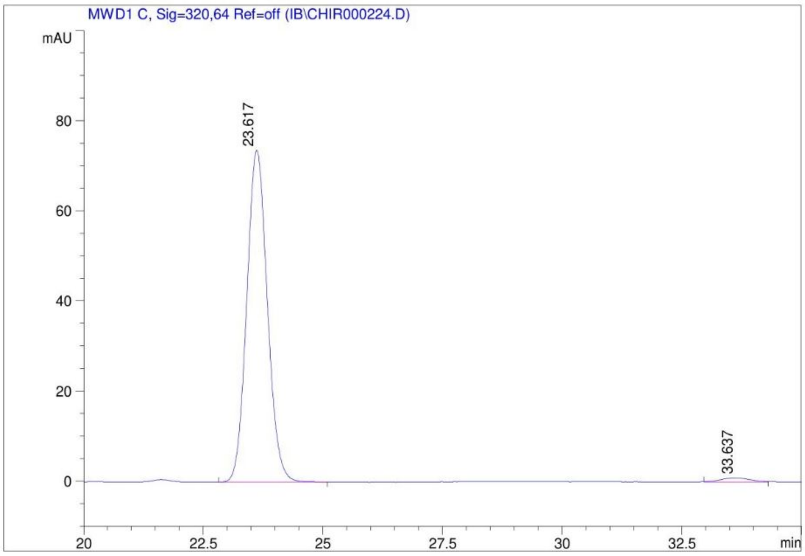


| # | Meas. R | Area | Area % |
|---|---|---|---|
| 1 | 23.617 | 2.208e3 | 98.127 |
| 2 | 33.637 | 42.160 | 1.873 |

**Figure S143**. HPLC chromatograms of **2b** (racemic – top, enantioenriched – bottom).

```
Data File:  C:\CHEM32\1\DATA\IB\RAC001020.D
Sample Name:  IBD-444-RAC
Sample Info:  Phenomenex Lux Amylose-1, 3 um, 90:10, 1.0 mL/min, RAC,
               p=93 bar; T=25st.C
```

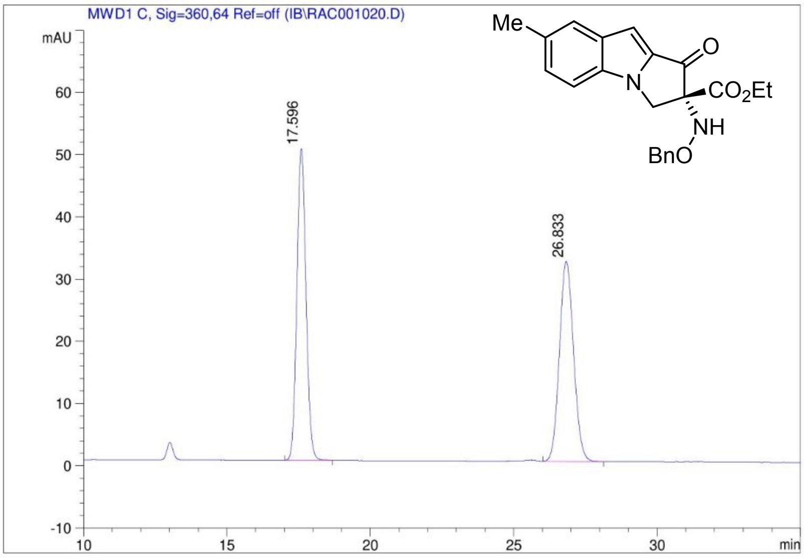


| # | Meas. R | Area | Area % |
|---|---|---|---|
| 1 | 17.596 | 1.106e3 | 50.070 |
| 2 | 26.833 | 1.103e3 | 49.930 |

```
Data File:  C:\CHEM32\1\DATA\IB\CHIR001021.D
Sample Name:  IBD-444-CH
Sample Info:  Phenomenex Lux Amylose-1, 3 um, 90:10, 1.0 mL/min, p=93
               bar; T=25st.C
```

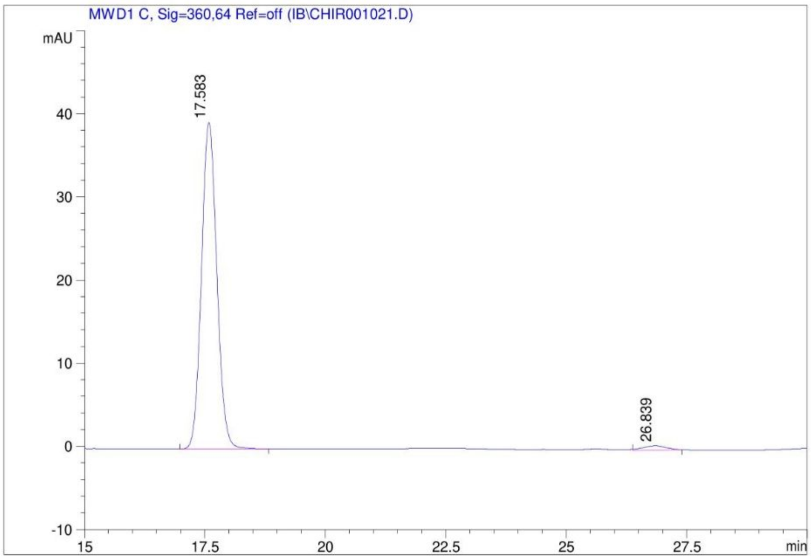


| # | Meas. R | Area | Area % |
|---|---|---|---|
| 1 | 17.583 | 868.268 | 97.974 |
| 2 | 26.839 | 17.958 | 2.026 |

**Figure S144.** HPLC chromatograms of **2c** (racemic – top, enantioenriched – bottom).

```
Data File:   C:\CHEM32\1\DATA\IB\RAC000012.D
Sample Name:   IBD-461-rac
Sample Info:   Phenomenex Lux Amylose-1, 3 um, 90:10, 1.00 mL/min, p=9
               5bar; T=25st.C
```

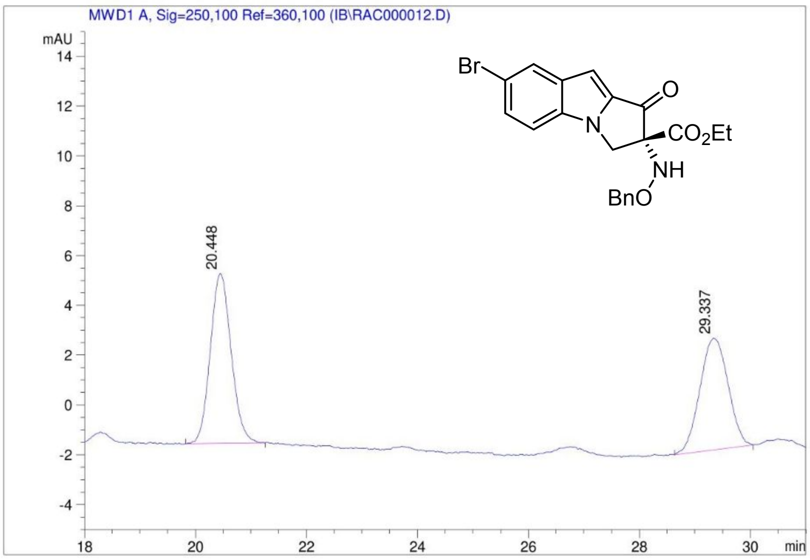


| # | Meas. R | Area | Area % |
|---|---|---|---|
| 1 | 20.448 | 177.676 | 52.909 |
| 2 | 29.337 | 158.138 | 47.091 |

```
Data File:   C:\CHEM32\1\DATA\IB\CHIR020133.D
Sample Name:   IBD-466
Sample Info:   Phenomenex Lux Amylose-1, 3 um, 90:10, 1.00 mL/min, chi
               r, p=92bar; T=25st.C
```

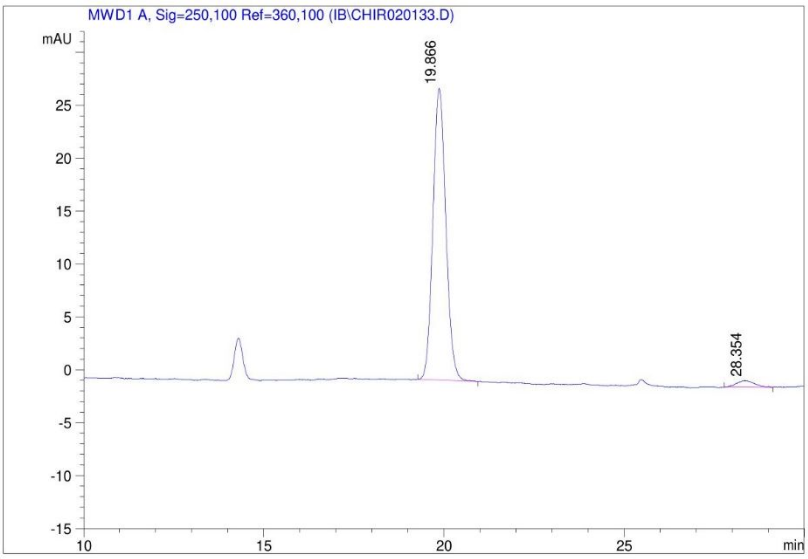


| # | Meas. R | Area | Area % |
|---|---|---|---|
| 1 | 19.866 | 686.308 | 97.047 |
| 2 | 28.354 | 20.882 | 2.953 |

**Figure S145**. HPLC chromatograms of **2d** (racemic – top, enantioenriched – bottom).

```
Data File:  C:\CHEM32\1\DATA\IB\RAC020195.D
Sample Name:  IBD-470-rac
Sample Info:  Phenomenex Lux Amylose-1, 3 um, 91:10, 1.00 mL/min, chi
              r, p=92bar; T=25st.C
```

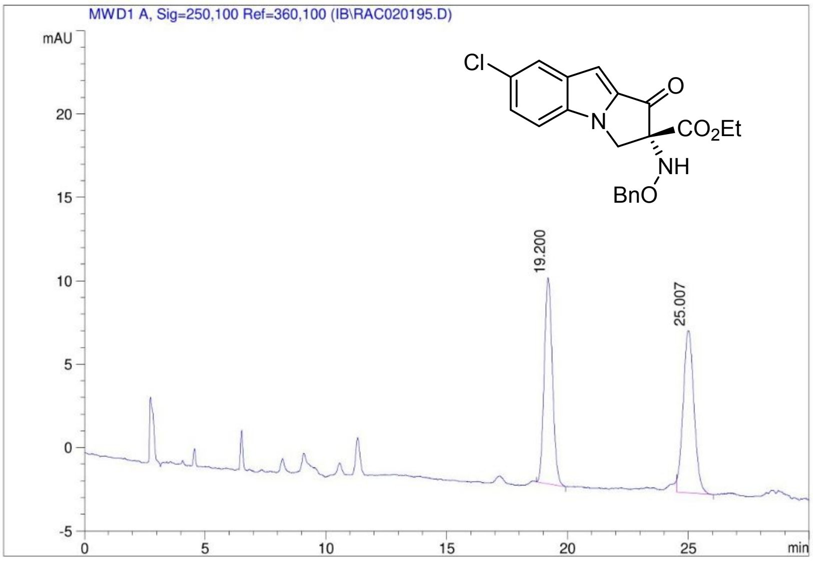


| # | Meas. R | Area | Area % |
|---|---|---|---|
| 1 | 19.200 | 291.119 | 48.897 |
| 2 | 25.007 | 304.257 | 51.103 |

```
Data File:  C:\CHEM32\1\DATA\IB\RAC020205.D
Sample Name:  IBD-470-ch
Sample Info:  Phenomenex Lux Amylose-1, 3 um, 91:10, 1.00 mL/min, chi
              r, p=92bar; T=25st.C
```

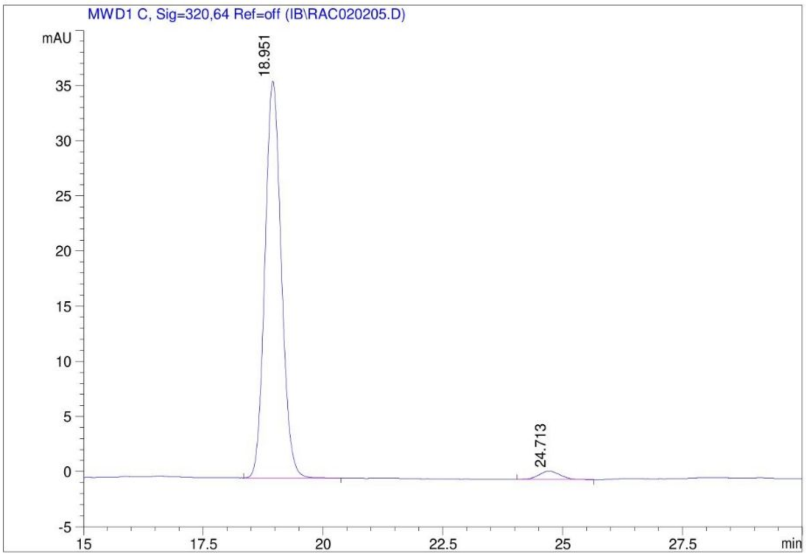


| # | Meas. R | Area | Area % |
|---|---|---|---|
| 1 | 18.951 | 853.895 | 97.258 |
| 2 | 24.713 | 24.070 | 2.742 |

**Figure S146**. HPLC chromatograms of **2e** (racemic – top, enantioenriched – bottom).

```
Data File:  C:\CHEM32\1\DATA\IB\RAC001067.D
Sample Name:  IBD-447-rac
Sample Info:  Phenomenex Lux cellulose-1, 3 um, 90:10, 1.0 mL/min, ra
              c, p=118 bar; T=25st.C
```

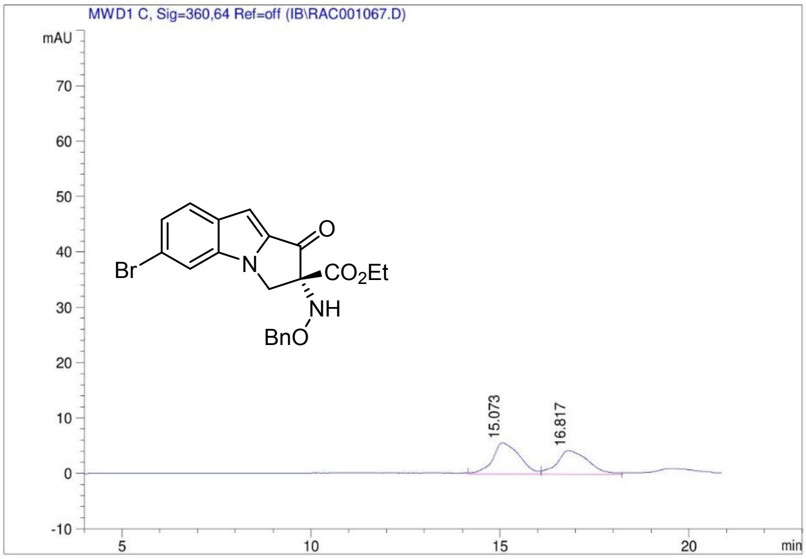


| # | Meas. R | Area | Area % |
|---|---|---|---|
| 1 | 15.073 | 282.624 | 53.352 |
| 2 | 16.817 | 247.113 | 46.648 |

```
Data File:  C:\CHEM32\1\DATA\IB\CHIR020145.D
Sample Name:  IBD-469
Sample Info:  Phenomenex Lux Cellulose-1, 3 um, 95:5, 0.80 mL/min, ch
              ir, p=87bar; T=25st.C
```

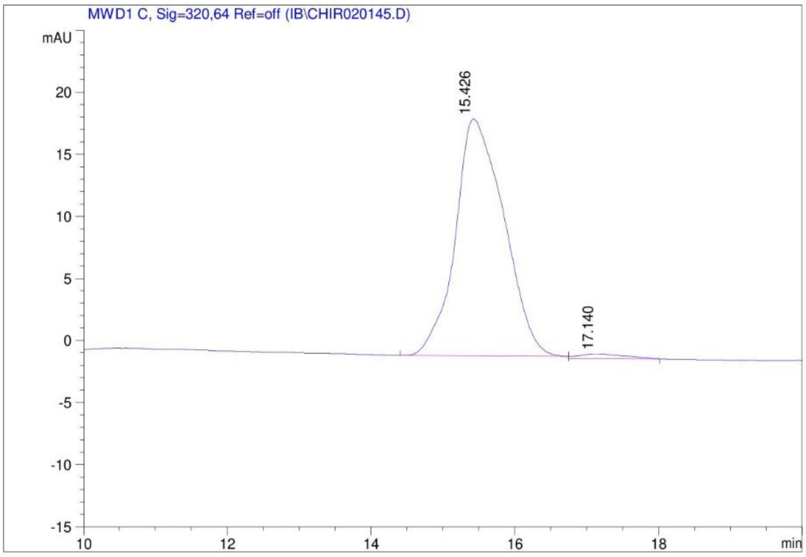


| # | Meas. R | Area | Area % |
|---|---|---|---|
| 1 | 15.426 | 898.901 | 98.097 |
| 2 | 17.140 | 17.441 | 1.903 |

**Figure S147.** HPLC chromatograms of **2f** (racemic – top, enantioenriched – bottom).

```
Data File:  C:\CHEM32\1\DATA\IB\RAC000163.D
Sample Name:  IBD-412-rac
Sample Info:  Phenomenex Lux Amylose-1, 3 um, 90:10, 1,0 mL/min, race
              mic, p=93 bar; T=25st.C
```

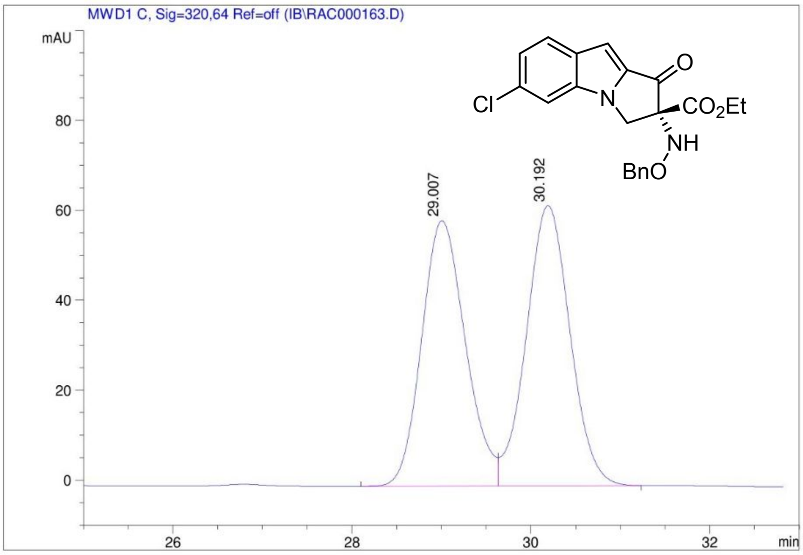


| # | Meas. R | Area | Area % |
|---|---|---|---|
| 1 | 29.007 | 2.008e3 | 48.654 |
| 2 | 30.192 | 2.119e3 | 51.346 |

```
Data File:  C:\CHEM32\1\DATA\IB\RAC000164.D
Sample Name:  IBD-412-chir
Sample Info:  Phenomenex Lux Amylose-1, 3 um, 95:5, 0,8 mL/min, chir,
               p=70 bar; T=25st.C
```

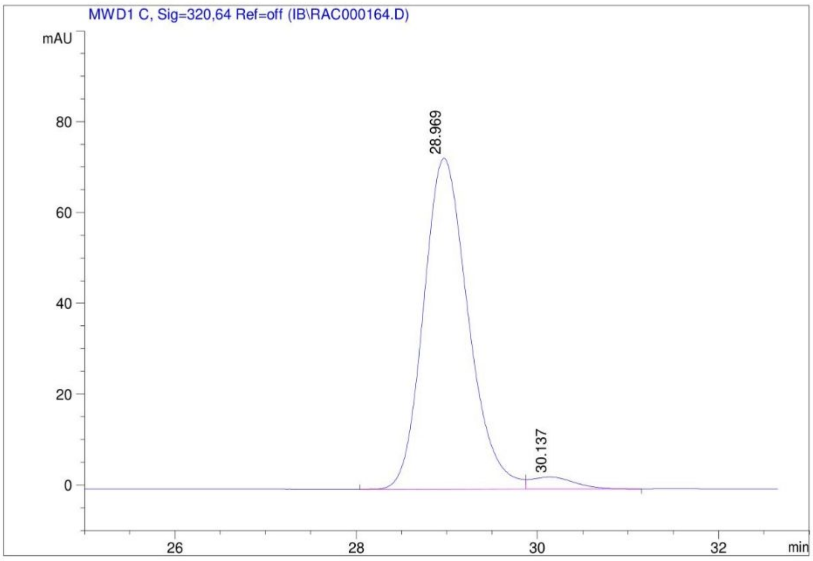


| # | Meas. R | Area | Area % |
|---|---|---|---|
| 1 | 28.969 | 2.499e3 | 96.612 |
| 2 | 30.137 | 87.646 | 3.388 |

**Figure S148.** HPLC chromatograms of **2g** (racemic – top, enantioenriched – bottom).

```
Data File:  C:\CHEM32\1\DATA\IB\RAC020209.D
Sample Name:  IBD-486-rac
Sample Info:  Phenomenex Lux Amylose-1, 3 um, 90:10, 1.00 mL/min, chi
              r, p=92bar; T=25st.C
```

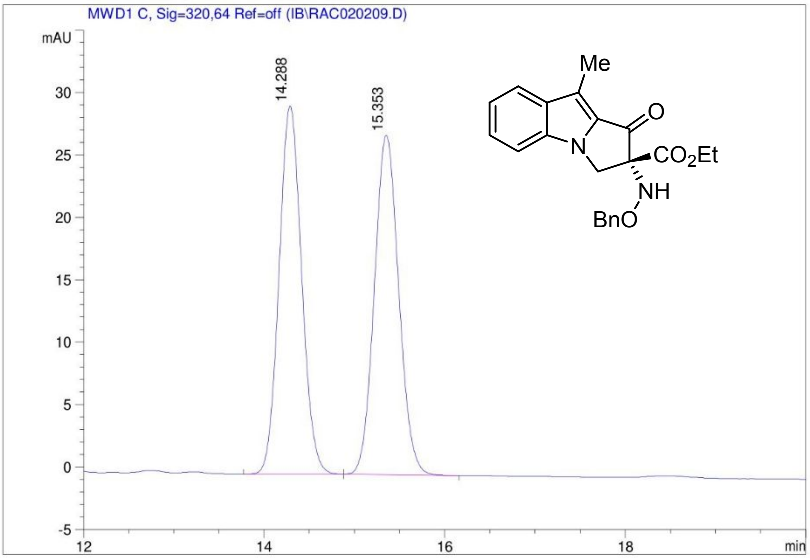


| # | Meas. R | Area | Area % |
|---|---|---|---|
| 1 | 14.288 | 513.166 | 50.120 |
| 2 | 15.353 | 510.708 | 49.880 |

```
Data File:  C:\CHEM32\1\DATA\IB\CHIR020210.D
Sample Name:  IBD-486-ch
Sample Info:  Phenomenex Lux Amylose-1, 3 um, 90:10, 1.00 mL/min, chi
              r, p=92bar; T=25st.C
```

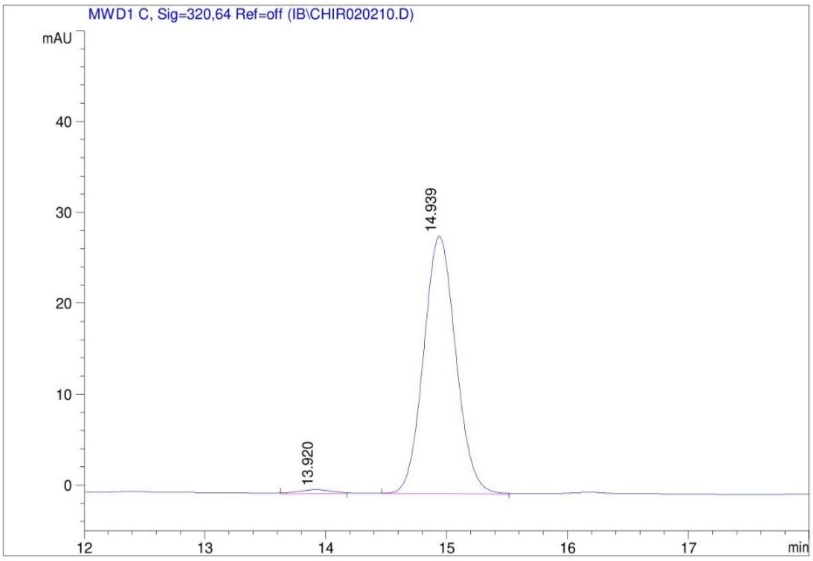


| # | Meas. R | Area | Area % |
|---|---|---|---|
| 1 | 13.920 | 7.912 | 1.490 |
| 2 | 14.939 | 522.977 | 98.510 |

**Figure S149**. HPLC chromatograms of **2h** (racemic – top, enantioenriched – bottom).

Data File: C:\CHEM32\1\DATA\IB\RAC000170.D
Sample Name: IBD-416-rac
Sample Info: Phenomenex Lux Amylose-1, 3 um, 90:10, 1.0 mL/min, rac,
p=95 bar; T=25st.C

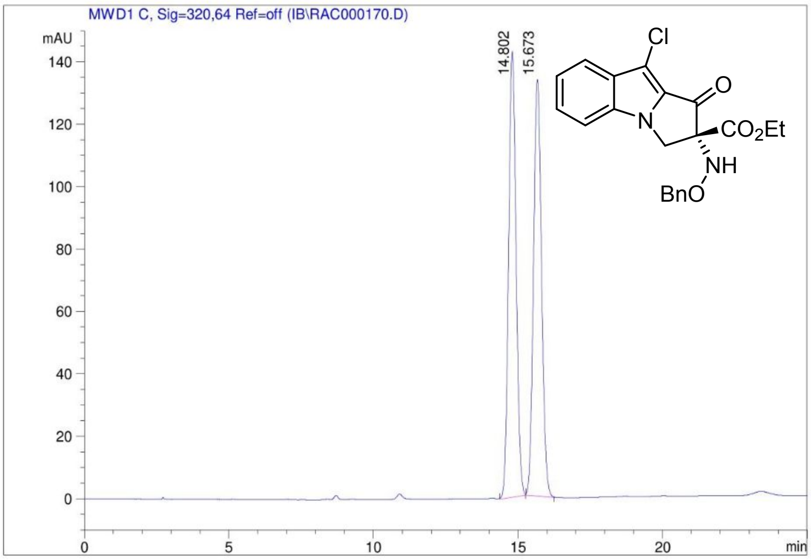


| # | Meas. R | Area | Area % |
|---|---|---|---|
| 1 | 14.802 | 2.586e3 | 50.081 |
| 2 | 15.673 | 2.578e3 | 49.919 |

Data File: C:\CHEM32\1\DATA\IB\CHIR000175.D
Sample Name: IBD-416-ch
Sample Info: Phenomenex Lux Amylose-1, 3 um, 90:10, 1.0 mL/min, rac,
p=95 bar; T=25st.C

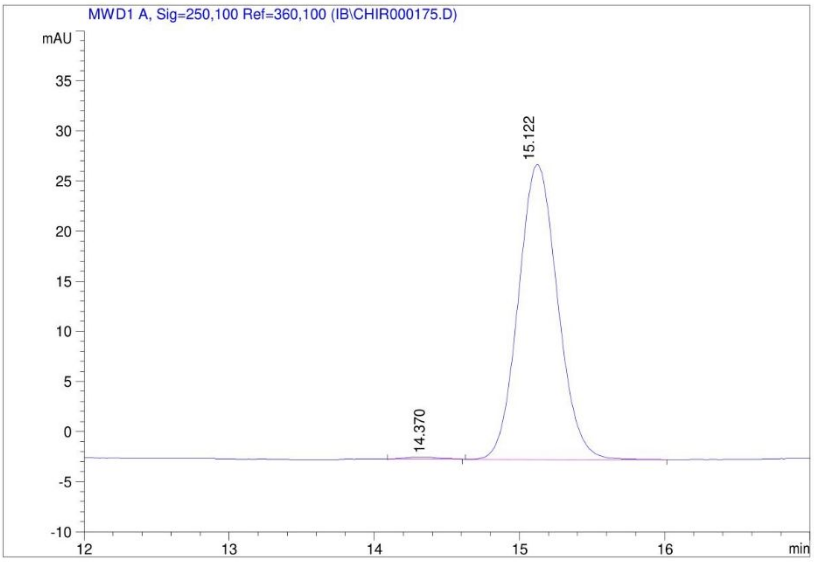


| # | Meas. R | Area | Area % |
|---|---|---|---|
| 1 | 14.370 | 3.161 | 0.568 |
| 2 | 15.122 | 553.723 | 99.432 |

**Figure S150.** HPLC chromatograms of **2i** (racemic – top, enantioenriched – bottom).

```
Data File:  C:\CHEM32\1\DATA\IB\RAC020194.D
Sample Name:  IBD-482-Rac
Sample Info:  Phenomenex Lux Amylose-1, 3 um, 91:10, 1.00 mL/min, chi
              r, p=92bar; T=25st.C
```

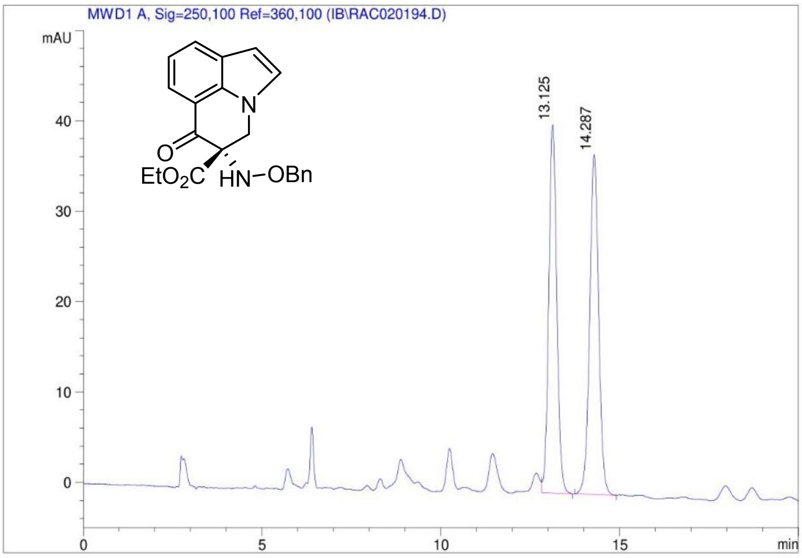


| # | Meas. R | Area | Area % |
|---|---|---|---|
| 1 | 13.125 | 633.983 | 49.029 |
| 2 | 14.287 | 659.083 | 50.971 |

```
Data File:  C:\CHEM32\1\DATA\IB\CHIR020175.D
Sample Name:  IBD-479
Sample Info:  Phenomenex Lux Amylose-1, 3 um, 90:10, 1.00 mL/min, chi
              r, p=93bar; T=25st.C
```

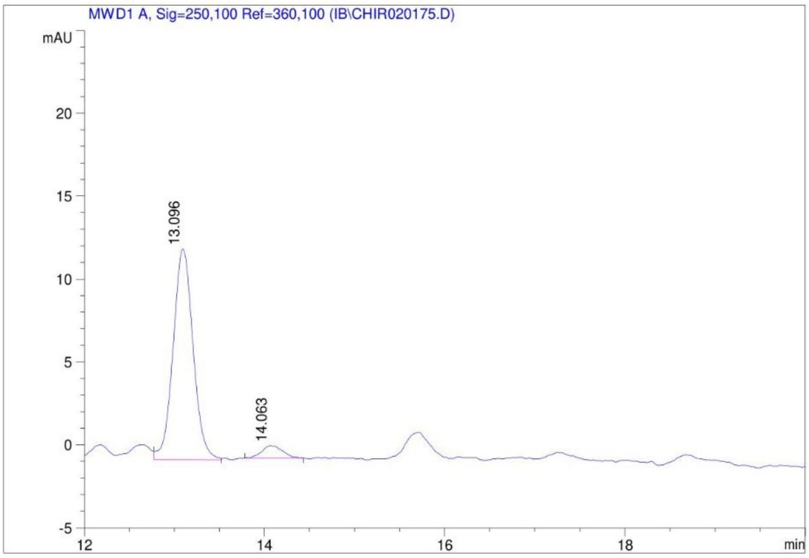


| # | Meas. R | Area | Area % |
|---|---|---|---|
| 1 | 13.096 | 195.074 | 94.342 |
| 2 | 14.063 | 11.699 | 5.658 |

**Figure S151.** HPLC chromatograms of **2j** (racemic – top, enantioenriched – bottom).

Data File: C:\CHEM32\1\DATA\IB\RAC001087.D
Sample Name: IBD-451-RAC
Sample Info: Phenomenex Lux Amylose-1, 3 um, 90:10, 1.00 mL/min, rac
, p=93 bar; T=25st.C

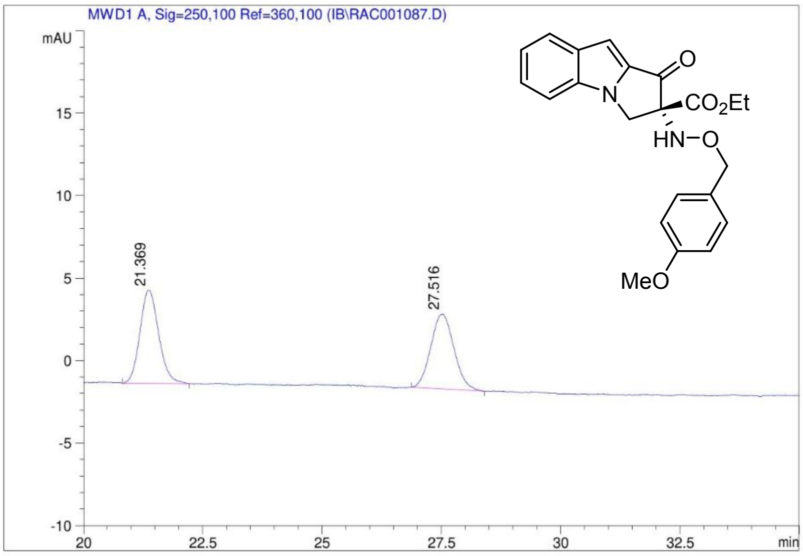


| # | Meas. R | Area | Area % |
|---|---|---|---|
| 1 | 21.369 | 154.728 | 50.204 |
| 2 | 27.516 | 153.469 | 49.796 |

Data File: C:\CHEM32\1\DATA\IB\RAC001088.D
Sample Name: IBD-419-CH
Sample Info: Phenomenex Lux Amylose-1, 3 um, 90:10, 1.00 mL/min, rac
, p=93 bar; T=25st.C

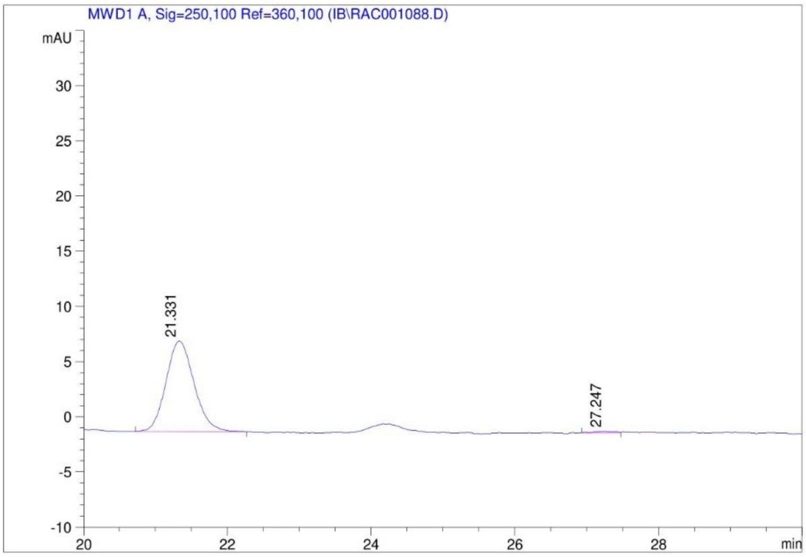


| # | Meas. R | Area | Area % |
|---|---|---|---|
| 1 | 21.331 | 223.887 | 98.888 |
| 2 | 27.247 | 2.519 | 1.112 |

**Figure S152.** HPLC chromatograms of **2n** (racemic – top, enantioenriched – bottom).

```
Data File:  C:\CHEM32\1\DATA\IB\RAC020408.D
Sample Name:  IBD-519-rac
Sample Info:  Phenomenex Lux Amylose-1, 3 um, 70:30, 1.00 mL/min, p=1
              26bar; T=25st.C
=====================================================================
```

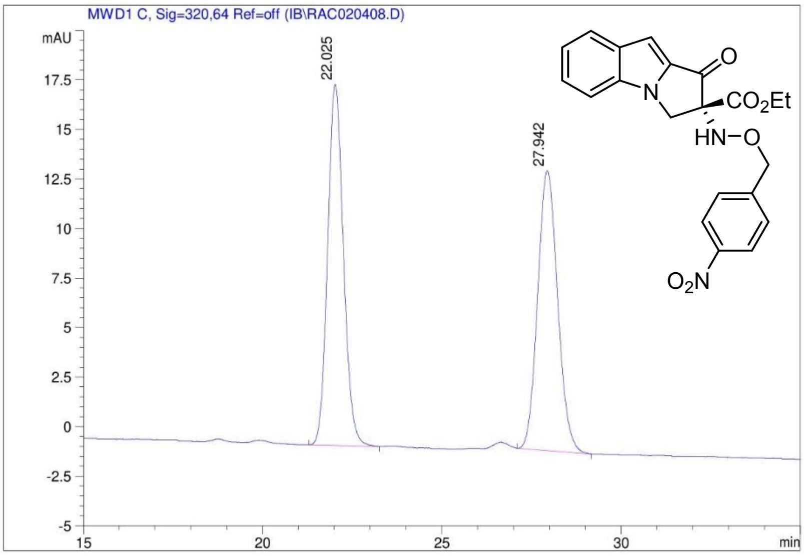


| # | Meas. R | Area | Area % |
|---|---|---|---|
| 1 | 22.025 | 558.698 | 50.502 |
| 2 | 27.942 | 547.595 | 49.498 |

```
Data File:  C:\CHEM32\1\DATA\IB\CHIR020416.D
Sample Name:  IBD-519-CH
Sample Info:  Phenomenex Lux Amylose-1, 3 um, 70:30, 1.00 mL/min, p=1
              26bar; T=25st.C
=====================================================================
```

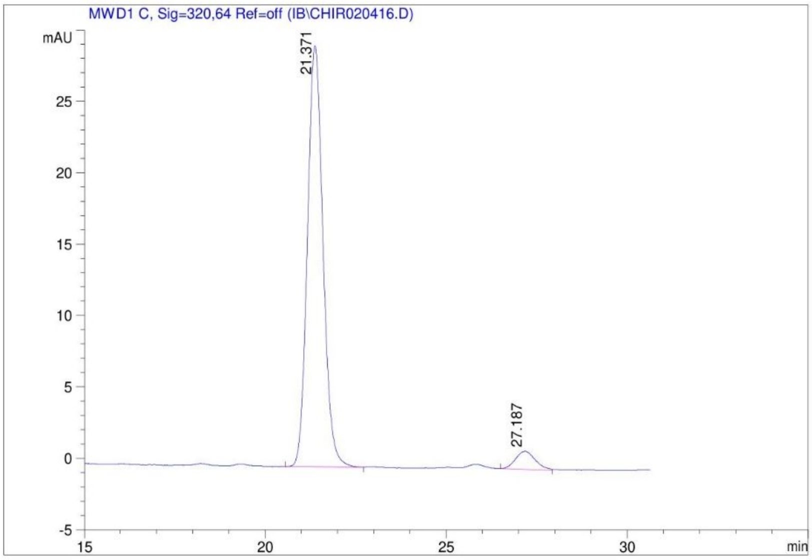


| # | Meas. R | Area | Area % |
|---|---|---|---|
| 1 | 21.371 | 881.224 | 94.685 |
| 2 | 27.187 | 49.463 | 5.315 |

**Figure S153.** HPLC chromatograms of **2o** (racemic – top, enantioenriched – bottom).

Data File: C:\CHEM32\1\DATA\IB\RAC020269.D
Sample Name: IBD-493-rac
Sample Info: Phenomenex Lux Amylose-1, 3 um, 90:10, 1.00 mL/min, rac
, p=91bar; T=25st.C

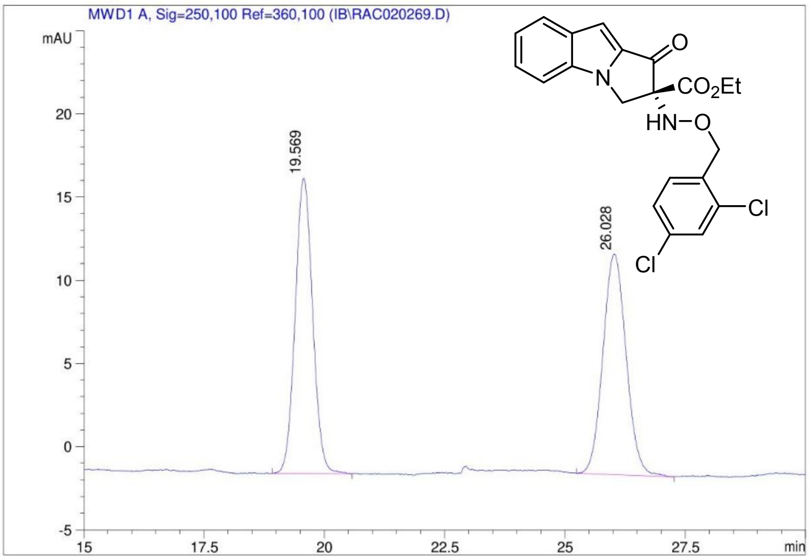


| # | Meas. R | Area | Area % |
|---|---|---|---|
| 1 | 19.569 | 448.641 | 50.194 |
| 2 | 26.028 | 445.168 | 49.806 |

Data File: C:\CHEM32\1\DATA\IB\RAC020271.D
Sample Name: IBD-493-chir
Sample Info: Phenomenex Lux Amylose-1, 3 um, 90:10, 1.00 mL/min, rac
, p=91bar; T=25st.C

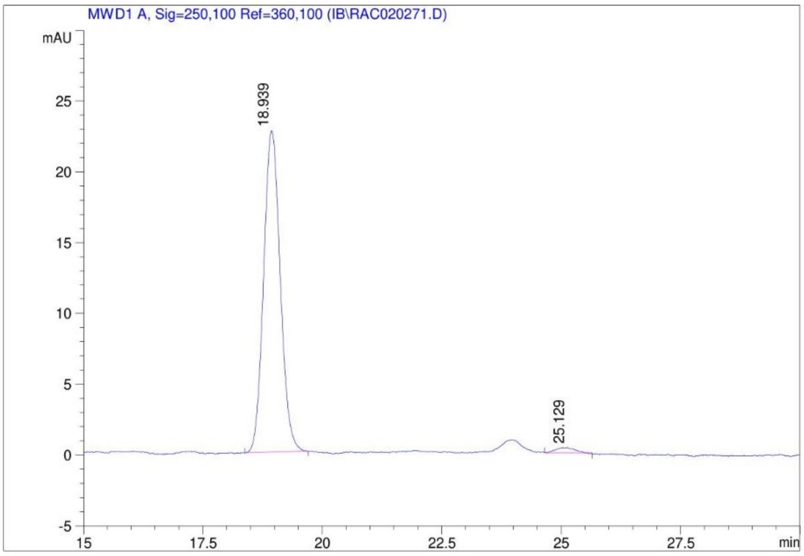


| # | Meas. R | Area | Area % |
|---|---|---|---|
| 1 | 18.939 | 549.142 | 98.134 |
| 2 | 25.129 | 10.440 | 1.866 |

**Figure S154.** HPLC chromatograms of **2p** (racemic – top, enantioenriched – bottom).

```
Data File:  C:\CHEM32\1\DATA\IB\RAC020361.D
Sample Name:  IBD-502-rac-cz
Sample Info:  Phenomenex Lux Amylose-1, 3 um, 80:20, 1.00 mL/min, p=1
              07bar; T=25st.C
=====================================================================
```

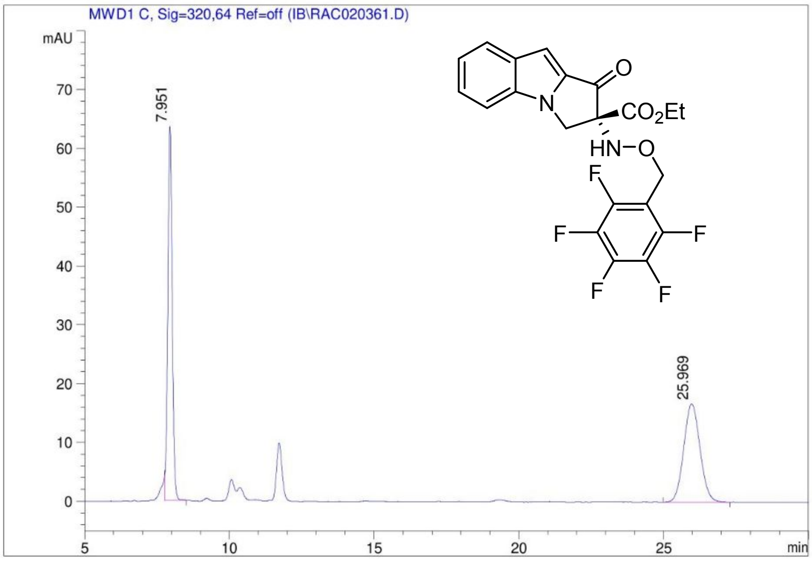


| # | Meas. R | Area | Area % |
|---|---|---|---|
| 1 | 7.951 | 668.807 | 50.624 |
| 2 | 25.969 | 652.308 | 49.376 |

```
Data File:  C:\CHEM32\1\DATA\IB\CHIR020373.D
Sample Name:  IBD-502-CH
Sample Info:  Phenomenex Lux Amylose-1, 3 um, 70:30, 1.00 mL/min, p=1
              25bar; T=25st.C
=====================================================================
```

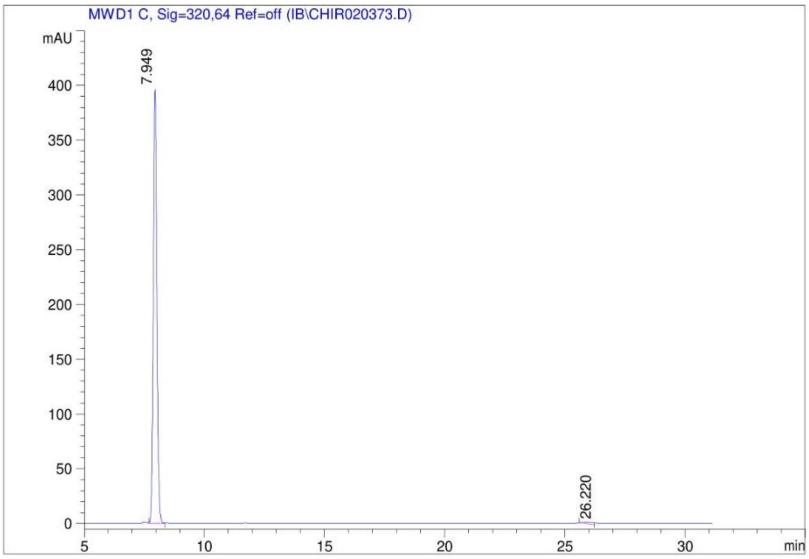


| # | Meas. R | Area | Area % |
|---|---|---|---|
| 1 | 7.949 | 4.084e3 | 98.974 |
| 2 | 26.220 | 42.334 | 1.026 |

**Figure S155.** HPLC chromatograms of **2q** (racemic – top, enantioenriched – bottom).

```
Data File:  C:\CHEM32\1\DATA\IB\RAC020304.D
Sample Name:  IBD-501-RAC
Sample Info:  Phenomenex Lux Amylose-1, 3 um, 90:10, 1.00 mL/min, rac
              , p=92bar; T=25st.C
===========================================================================
```

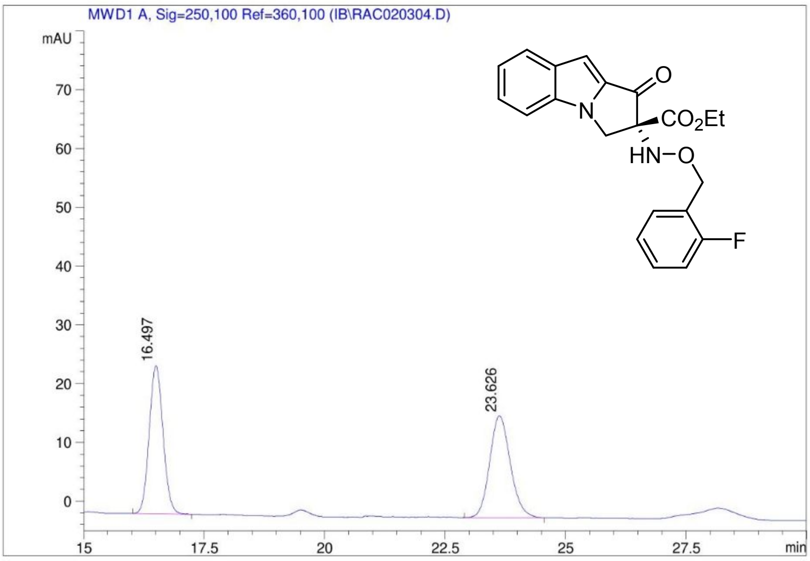


| # | Meas. R | Area | Area % |
|---|---|---|---|
| 1 | 16.497 | 501.273 | 49.189 |
| 2 | 23.626 | 517.811 | 50.811 |

```
Data File:  C:\CHEM32\1\DATA\IB\RAC020305.D
Sample Name:  IBD-501-CH
Sample Info:  Phenomenex Lux Amylose-1, 3 um, 90:10, 1.00 mL/min, rac
              , p=92bar; T=25st.C
===========================================================================
```

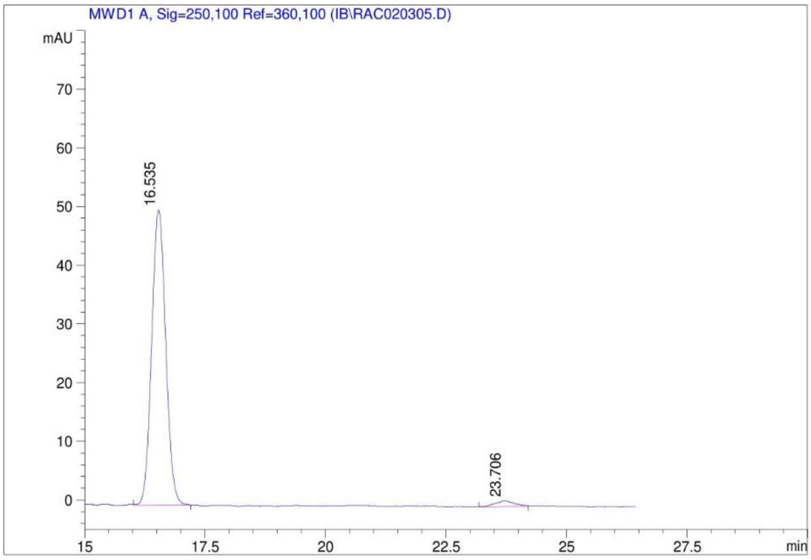


| # | Meas. R | Area | Area % |
|---|---|---|---|
| 1 | 16.535 | 1.020e3 | 97.131 |
| 2 | 23.706 | 30.133 | 2.869 |

**Figure S156.** HPLC chromatograms of **2r** (racemic – top, enantioenriched – bottom).

```
Data File:  C:\CHEM32\1\DATA\IB\RAC020701.D
Sample Name:  IBD-580-rac
Sample Info:  Phenomenex Lux Cellulose-1, 3 um, 95:5, 0.5 mL/min, p=5
              4bar; T=25st.C
=======================================================================
```

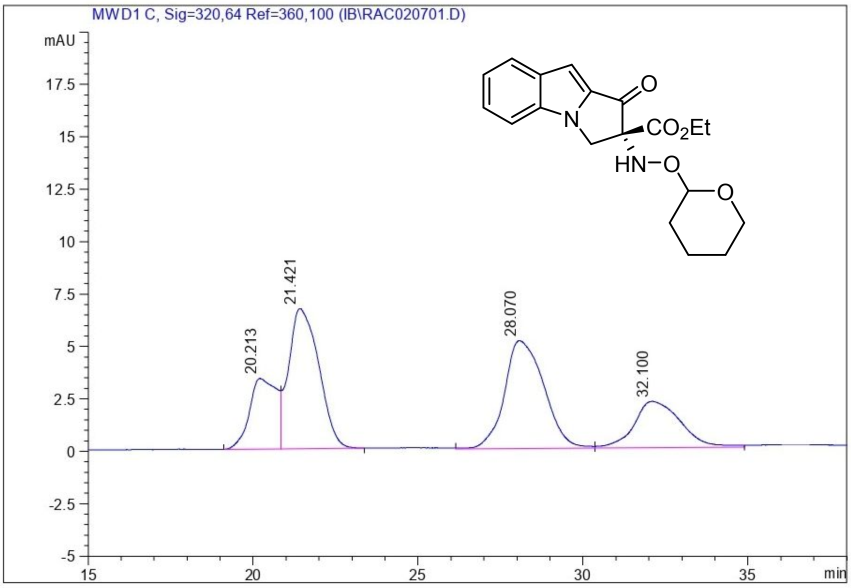


| # | Meas. R | Area | Area % |
|---|---|---|---|
| 1 | 20.213 | 192.941 | 15.231 |
| 2 | 21.421 | 426.822 | 33.694 |
| 3 | 28.070 | 430.111 | 33.954 |
| 4 | 32.100 | 216.882 | 17.121 |

**Figure S157**. HPLC chromatogram of **2s** (racemic).

```
Data File:  C:\CHEM32\1\DATA\IB\CHIR020703.D
Sample Name:  IBD-580-ch-1
Sample Info:  Phenomenex Lux Cellulose-1, 3 um, 95:5, 0.5 mL/min, p=5
              4bar; T=25st.C
```

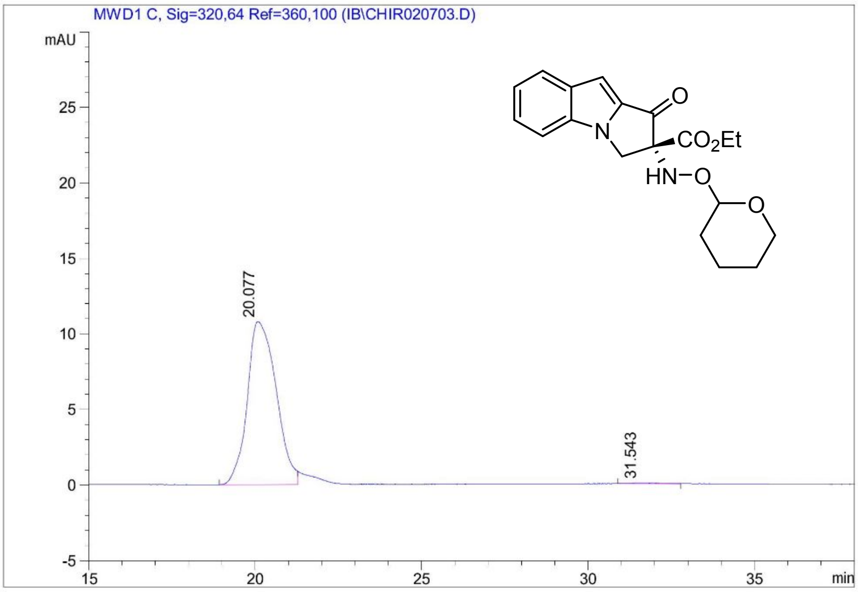


| # | Meas. R | Area | Area % |
|---|---|---|---|
| 1 | 20.077 | 650.624 | 99.590 |
| 2 | 31.543 | 2.680 | 0.410 |

```
Data File:  C:\CHEM32\1\DATA\IB\RAC020702.D
Sample Name:  IBD-580-ch-3
Sample Info:  Phenomenex Lux Cellulose-1, 3 um, 95:5, 0.5 mL/min, p=5
              4bar; T=25st.C
```

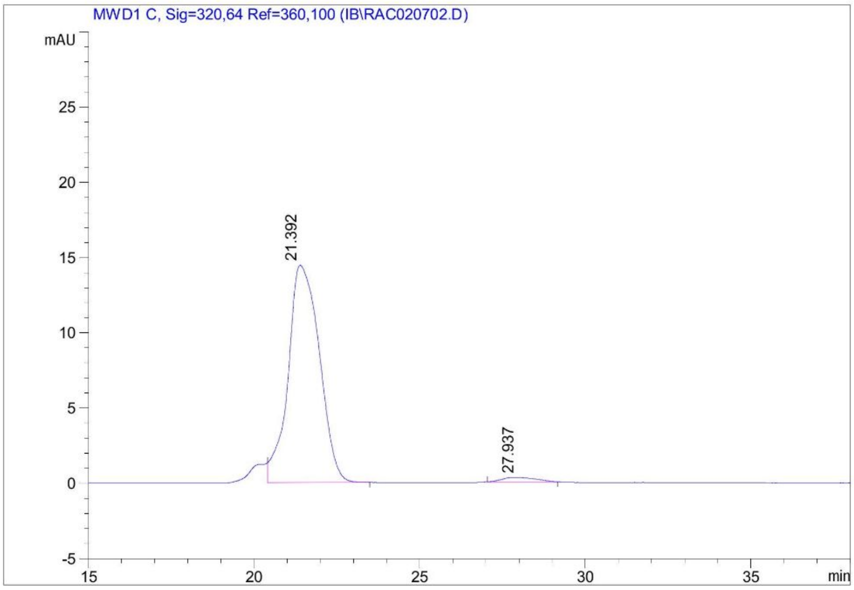


| # | Meas. R | Area | Area % |
|---|---|---|---|
| 1 | 21.392 | 950.851 | 97.647 |
| 2 | 27.937 | 22.913 | 2.353 |

**Figure S158.** HPLC chromatograms of **2s** (diastereoisomer 1 – top, diastereoisomer 2– bottom).

```
Data File:  C:\CHEM32\1\DATA\IB\RAC020542.D
Sample Name:  IBD-572-rac
Sample Info:  Phenomenex Lux Amylose-1, 3 um, 90:10, 1.00 mL/min, p=9
              5bar; T=25st.C
==========================================================================
```

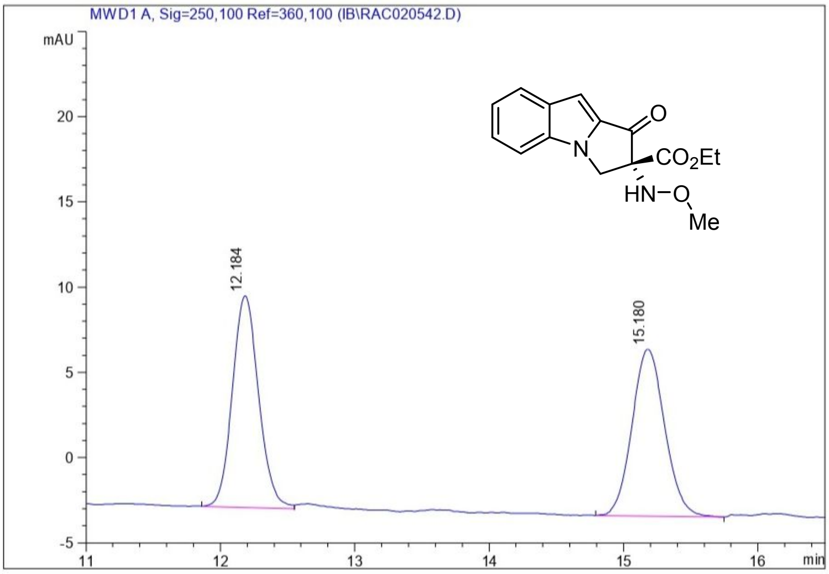


| # | Meas. R | Area | Area % |
|---|---|---|---|
| 1 | 12.184 | 168.672 | 50.413 |
| 2 | 15.180 | 165.908 | 49.587 |

```
Data File:  C:\CHEM32\1\DATA\IB\CHIR020543.D
Sample Name:  IBD-572-chir
Sample Info:  Phenomenex Lux Amylose-1, 3 um, 90:10, 1.00 mL/min, p=9
              7bar; T=25st.C
==========================================================================
```

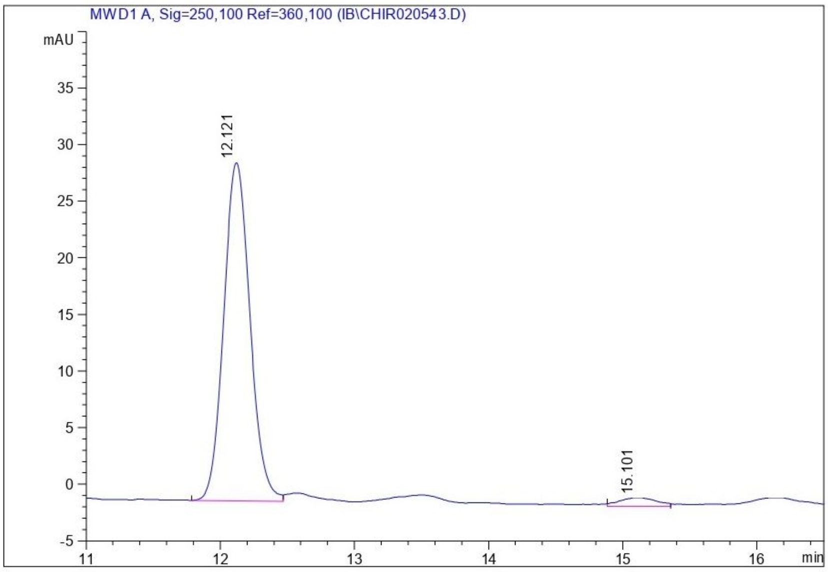


| # | Meas. R | Area | Area % |
|---|---|---|---|
| 1 | 12.121 | 405.905 | 96.398 |
| 2 | 15.101 | 15.169 | 3.602 |

**Figure S159.** HPLC chromatograms of **2t** (top-racemic, botom- chiral).

```
Data File:  C:\CHEM32\1\DATA\IB\RAC020393.D
Sample Name:  IBD-514-rac
Sample Info:  Phenomenex Lux Amylose-1, 3 um, 90:10, 1.00 mL/min, p=9
              1bar; T=25st.C
```

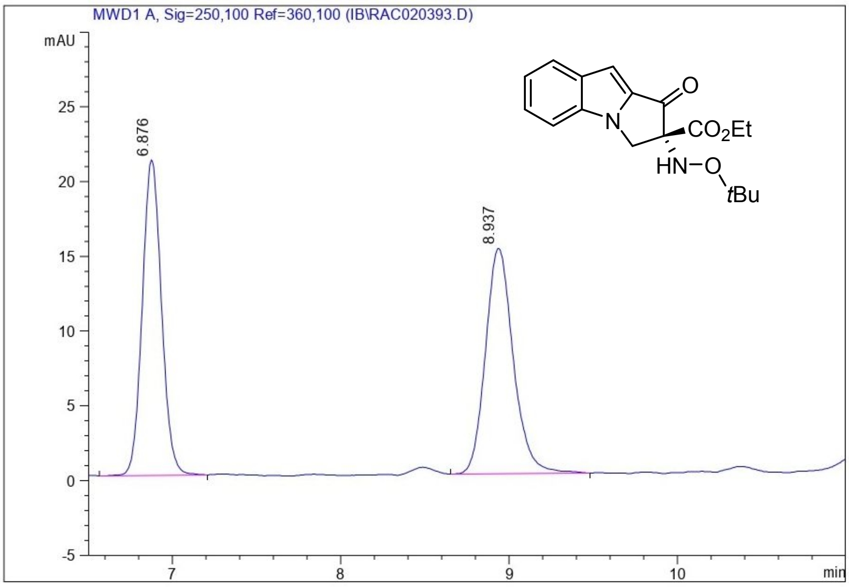


| # | Meas. R | Area | Area % |
|---|---|---|---|
| 1 | 6.876 | 167.268 | 49.076 |
| 2 | 8.937 | 173.570 | 50.924 |

```
Data File:  C:\CHEM32\1\DATA\IB\RAC020396.D
Sample Name:  IBD-514-CH
Sample Info:  Phenomenex Lux Amylose-1, 3 um, 90:10, 1.00 mL/min, p=9
              1bar; T=25st.C
```

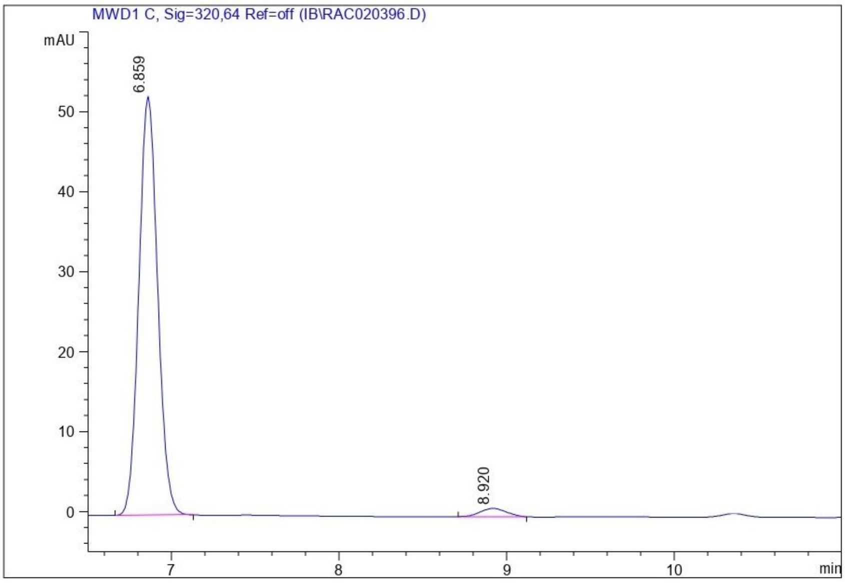


| # | Meas. R | Area | Area % |
|---|---|---|---|
| 1 | 6.859 | 406.999 | 97.370 |
| 2 | 8.920 | 10.995 | 2.630 |

**Figure S160.** HPLC chromatograms of **2u** (top-racemic, botom- chiral).

Data File: C:\CHEM32\1\DATA\IB\RAC020790.D
Sample Name: IBD-594-rac-czysty
Sample Info: Phenomenex Lux Amylose-1, 3 um, 90:10, 1.0 mL/min, p=95 bar; T=25st.C

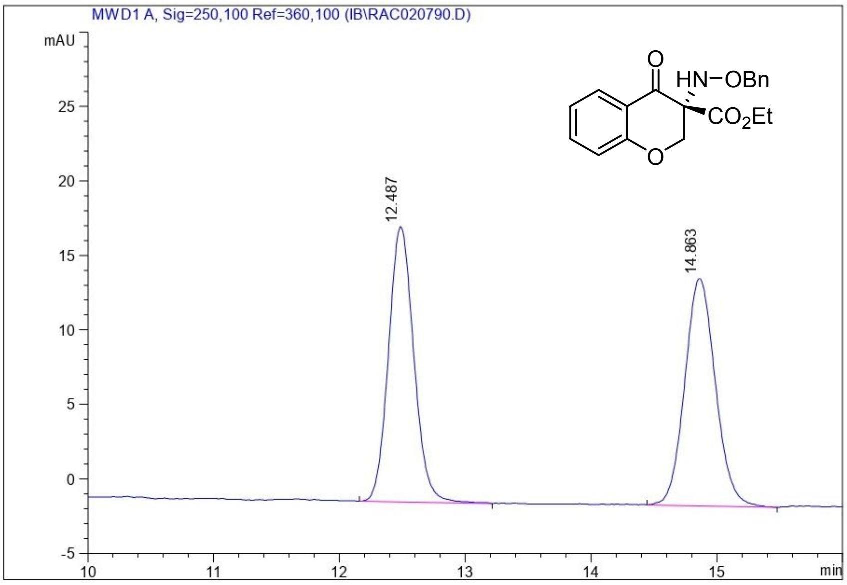


| # | Meas. R | Area | Area % |
|---|---|---|---|
| 1 | 12.487 | 259.105 | 50.019 |
| 2 | 14.863 | 258.904 | 49.981 |

Data File: C:\CHEM32\1\DATA\IB\CHIR020778.D
Sample Name: IBD-594-ch
Sample Info: Phenomenex Lux Amylose-1, 3 um, 90:10, 1.0 mL/min, p=96 bar; T=25st.C

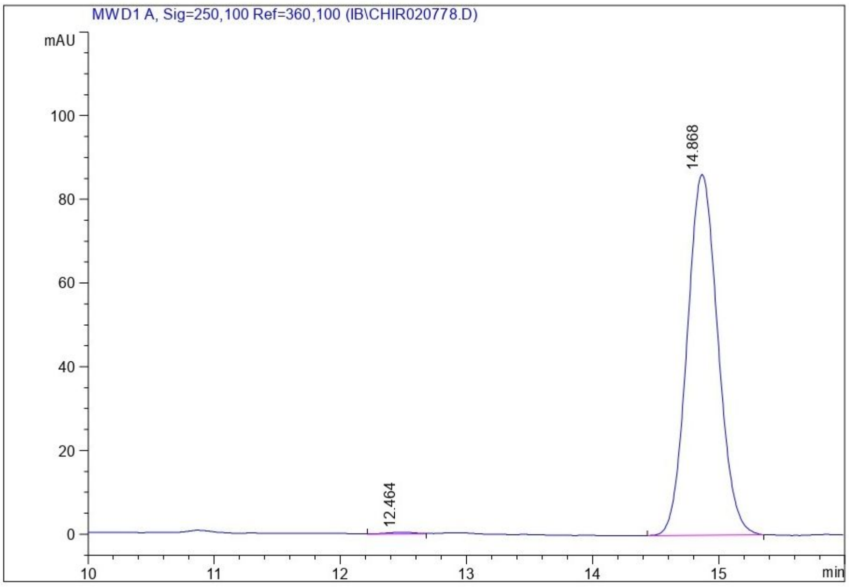


| # | Meas. R | Area | Area % |
|---|---|---|---|
| 1 | 12.464 | 5.775 | 0.395 |
| 2 | 14.868 | 1.457e3 | 99.605 |

**Figure S161**. HPLC chromatograms of **4a** (top-racemic, botom- chiral).

```
Data File:  C:\CHEM32\1\DATA\IB\RAC001575.D
Sample Name:  IBD-623-rac-docz
Sample Info:  Phenomenex Lux Amylose-1, 3 um, 90:10, 1.0 mL/min, p=98
              bar; T=25st.C
==========================================================================
```

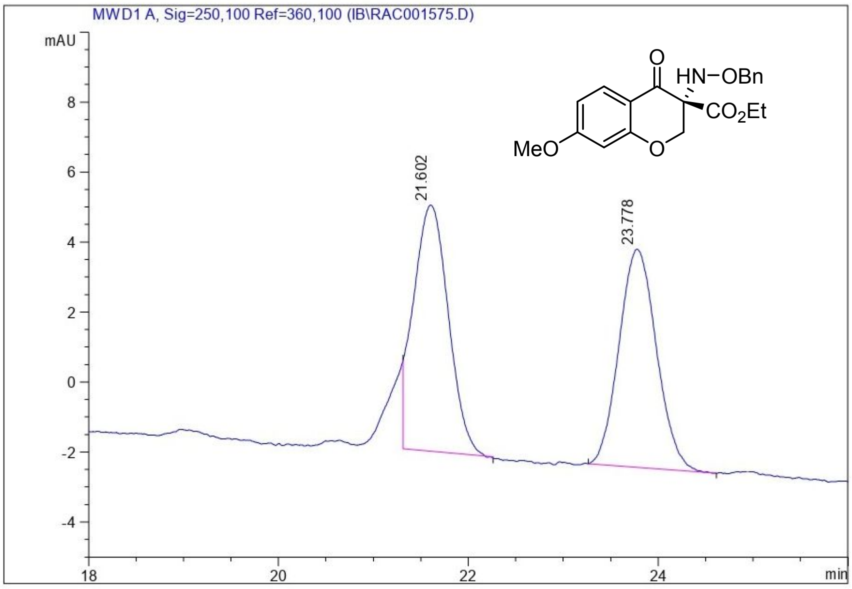


| # | Meas. R | Area | Area % |
|---|---|---|---|
| 1 | 21.602 | 178.715 | 51.050 |
| 2 | 23.778 | 171.367 | 48.950 |

```
Data File:  C:\CHEM32\1\DATA\IB\CHIR000997.D
Sample Name:  IBD-623-chir
Sample Info:  Phenomenex Lux Amylose-1, 3 um, 90:10, 1.0 mL/min, p=94
              bar; T=25st.C
==========================================================================
```

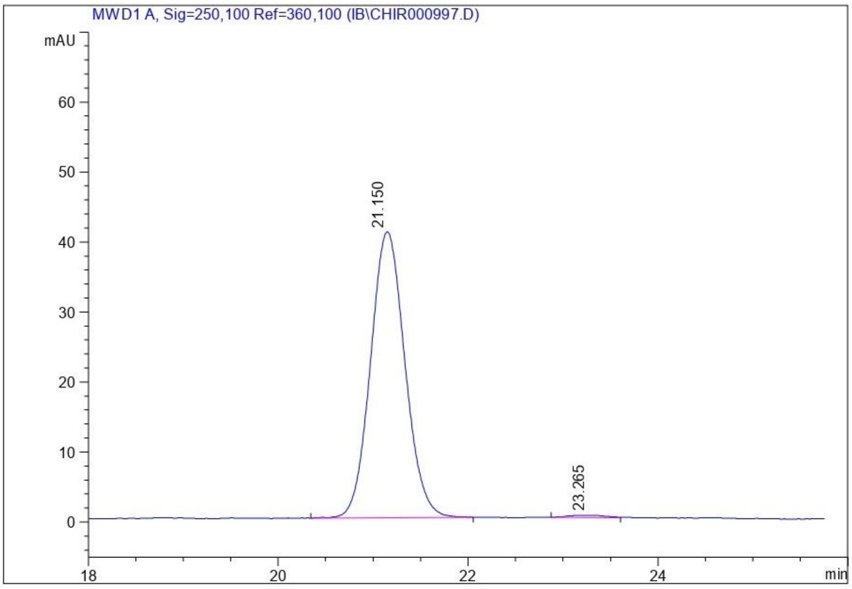


| # | Meas. R | Area | Area % |
|---|---|---|---|
| 1 | 21.150 | 1.029e3 | 99.099 |
| 2 | 23.265 | 9.363 | 0.901 |

**Figure S162.** HPLC chromatograms of **4b** (top-racemic, botom- chiral).

```
Data File:  C:\CHEM32\1\DATA\IB\RAC000921.D
Sample Name:  IBD-609-rac
Sample Info:  Phenomenex Lux Amylose-1, 3 um, 90:10, 1.0 mL/min, p=96
              bar; T=25st.C
```

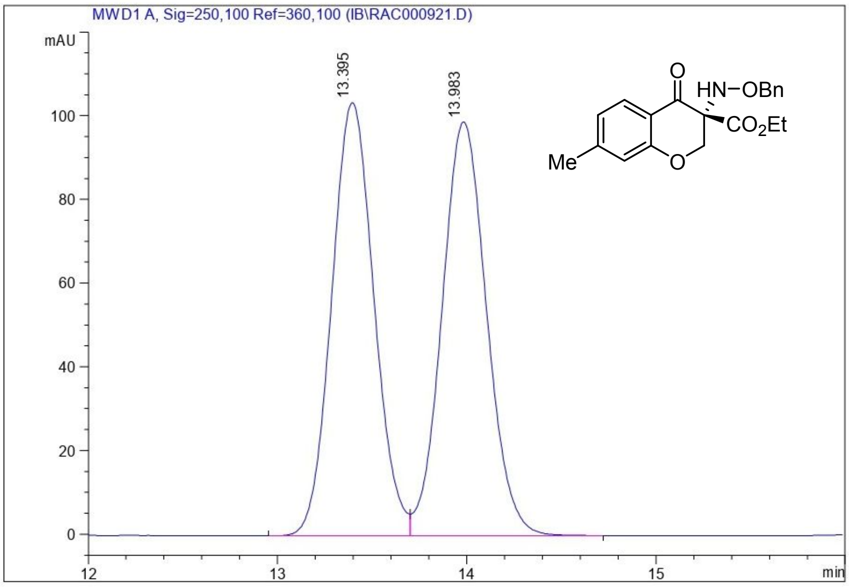


| # | Meas. R | Area | Area % |
|---|---|---|---|
| 1 | 13.395 | 1.606e3 | 49.734 |
| 2 | 13.983 | 1.623e3 | 50.266 |

```
Data File:  C:\CHEM32\1\DATA\IB\CHIR000924.D
Sample Name:  IBD-609-ch
Sample Info:  Phenomenex Lux Amylose-1, 3 um, 90:10, 1.0 mL/min, p=96
              bar; T=25st.C
```

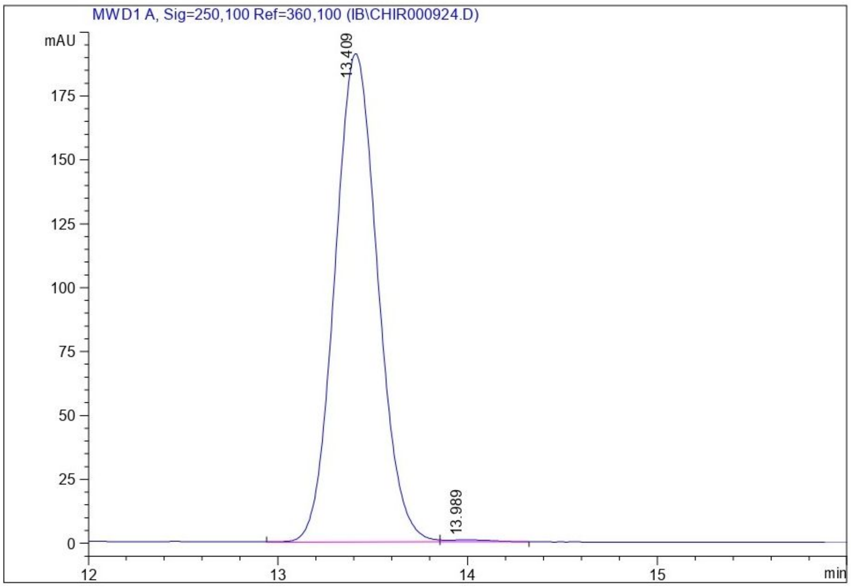


| # | Meas. R | Area | Area % |
|---|---|---|---|
| 1 | 13.409 | 3.008e3 | 99.610 |
| 2 | 13.989 | 11.786 | 0.390 |

**Figure S163.** HPLC chromatograms of **4c** (top-racemic, botom- chiral).

```
Data File:  C:\CHEM32\1\DATA\IB\CHIR001560.D
Sample Name:  IBD-637-rac
Sample Info:  Phenomenex Lux Amylose-1, 3 um, 90:10, 1.0 mL/min, p=95
              bar; T=25st.C
=====================================================================
```

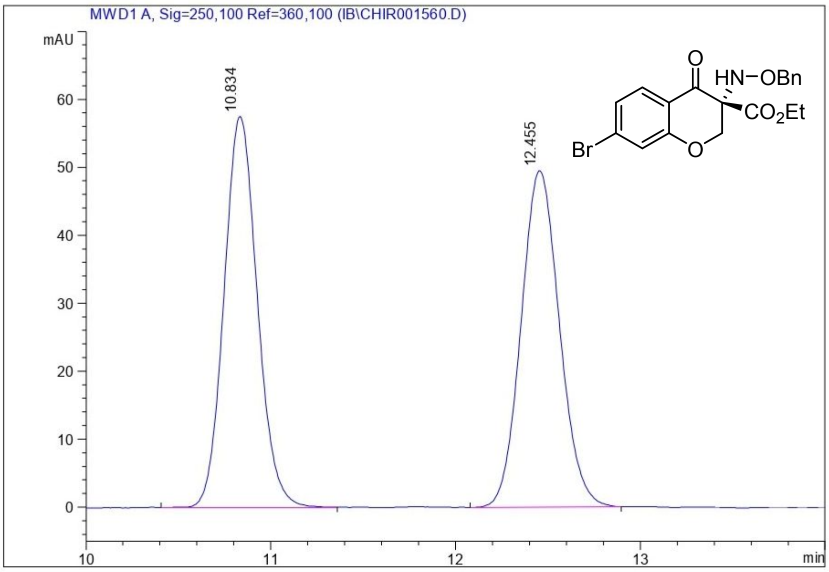


| # | Meas. R | Area | Area % |
|---|---|---|---|
| 1 | 10.834 | 719.592 | 50.196 |
| 2 | 12.455 | 713.964 | 49.804 |

```
Data File:  C:\CHEM32\1\DATA\IB\CHIR001502.D
Sample Name:  IBD-642-ch
Sample Info:  Phenomenex Lux Amylose-1, 3 um, 90:10, 1.0 mL/min, p=95
              bar; T=25st.C
=====================================================================
```

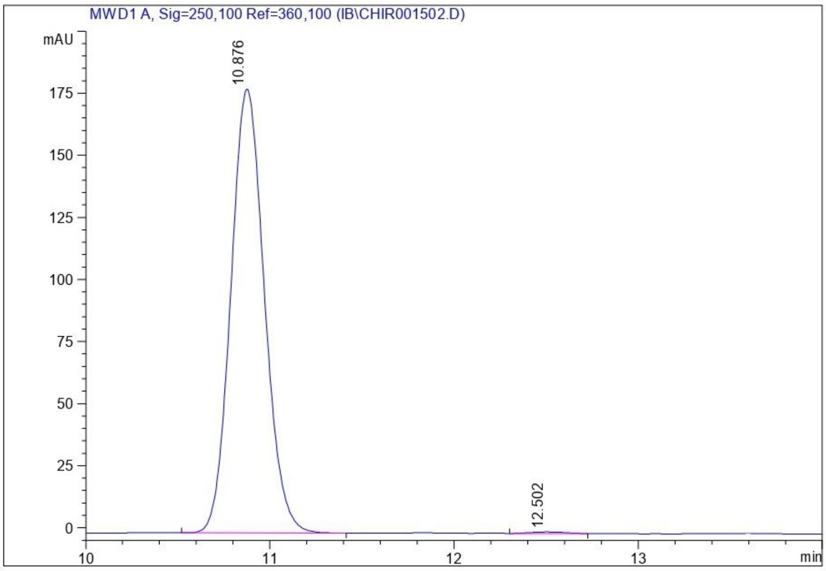


| # | Meas. R | Area | Area % |
|---|---|---|---|
| 1 | 10.876 | 2.260e3 | 99.633 |
| 2 | 12.502 | 8.333 | 0.367 |

**Figure S164**. HPLC chromatograms of **4d** (top-racemic, botom- chiral).

```
Data File:  C:\CHEM32\1\DATA\IB\RAC000918.D
Sample Name:  IBD-608-rac
Sample Info:  Phenomenex Lux Amylose-1, 3 um, 90:10, 1.0 mL/min, p=96
              bar; T=25st.C
```

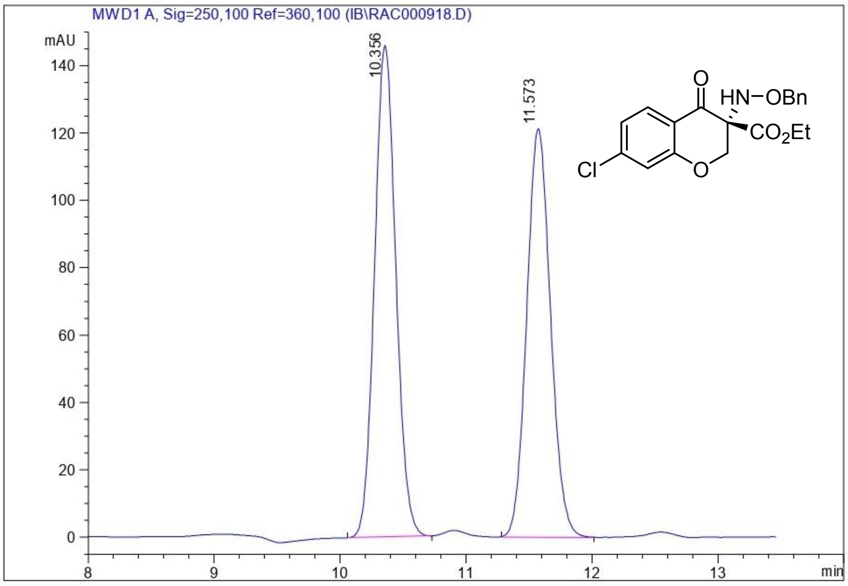


| # | Meas. R | Area | Area % |
|---|---|---|---|
| 1 | 10.356 | 1.713e3 | 51.749 |
| 2 | 11.573 | 1.597e3 | 48.251 |

```
Data File:  C:\CHEM32\1\DATA\IB\CHIR000920.D
Sample Name:  IBD-608-ch
Sample Info:  Phenomenex Lux Amylose-1, 3 um, 90:10, 1.0 mL/min, p=98
              bar; T=25st.C
```

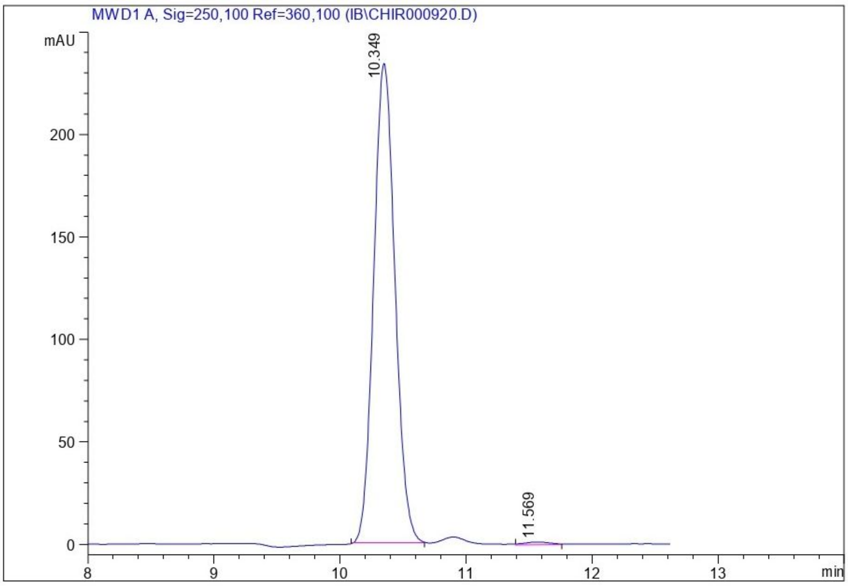


| # | Meas. R | Area | Area % |
|---|---|---|---|
| 1 | 10.349 | 2.747e3 | 99.360 |
| 2 | 11.569 | 17.693 | 0.640 |

**Figure S165**. HPLC chromatograms of **4e** (top-racemic, botom- chiral).

Data File: C:\CHEM32\1\DATA\IB\RAC000986.D
Sample Name: IBD-618-rac
Sample Info: Phenomenex Lux Amylose-1, 3 um, 90:10, 1.0 mL/min, p=94 bar; T=25st.C

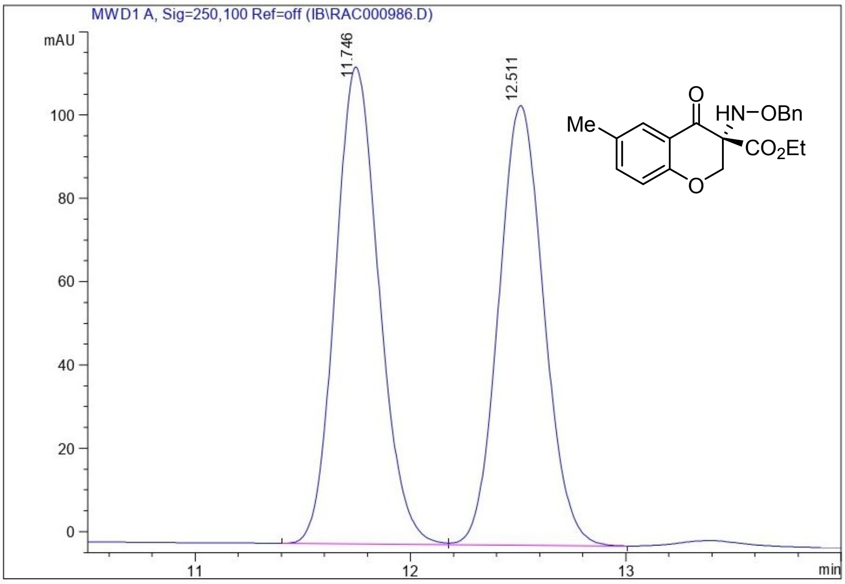


| # | Meas. R | Area | Area % |
|---|---|---|---|
| 1 | 11.746 | 1.614e3 | 51.176 |
| 2 | 12.511 | 1.540e3 | 48.824 |

Data File: C:\CHEM32\1\DATA\IB\RAC000987.D
Sample Name: IBD-618-ch
Sample Info: Phenomenex Lux Amylose-1, 3 um, 90:10, 1.0 mL/min, p=94 bar; T=25st.C

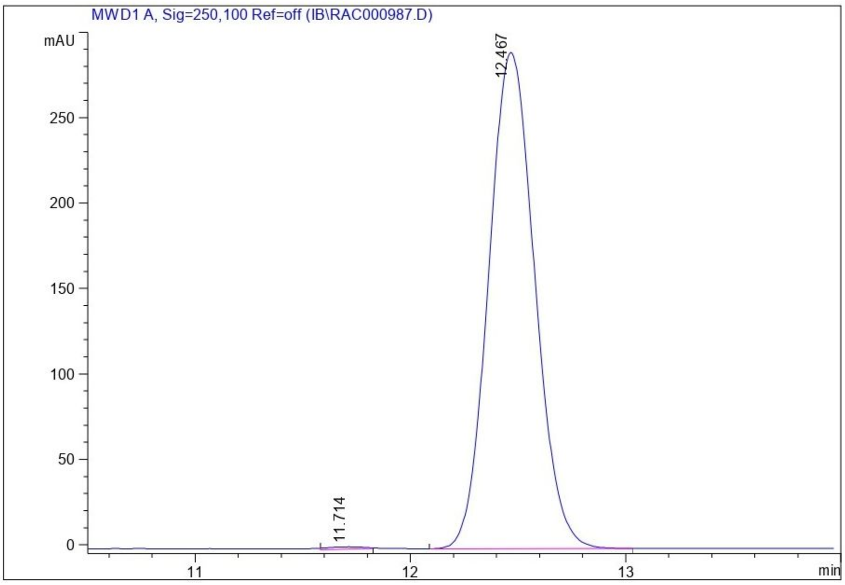


| # | Meas. R | Area | Area % |
|---|---|---|---|
| 1 | 11.714 | 16.513 | 0.381 |
| 2 | 12.467 | 4.318e3 | 99.619 |

**Figure S166**. HPLC chromatograms of **4g** (top-racemic, botom- chiral).

```
Data File:  C:\CHEM32\1\DATA\IB\RAC001483.D
Sample Name:  IBD-633-rac
Sample Info:  Phenomenex Lux Amylose-1, 3 um, 90:10, 1.0 mL/min, p=95
              bar; T=25st.C
========================================================================
```

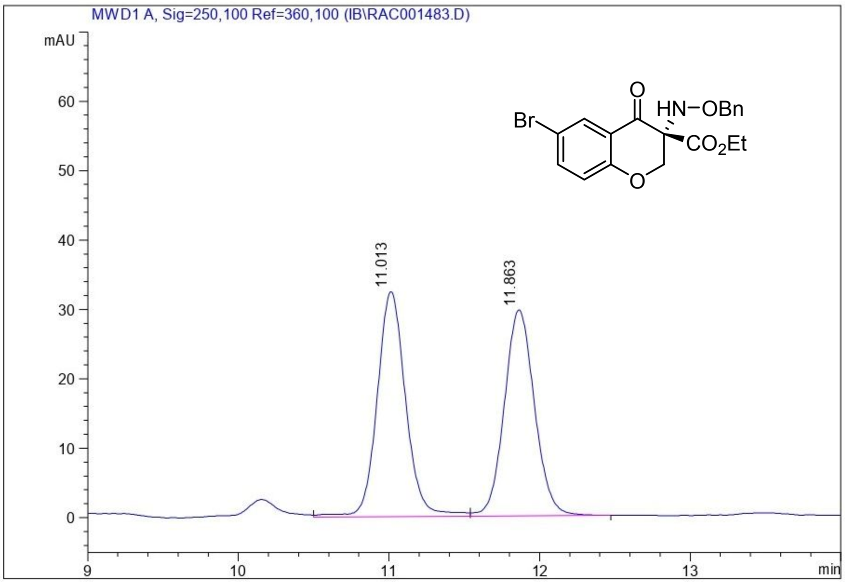


| # | Meas. R | Area | Area % |
|---|---|---|---|
| 1 | 11.013 | 430.157 | 50.911 |
| 2 | 11.863 | 414.767 | 49.089 |

```
Data File:  C:\CHEM32\1\DATA\IB\RAC001485.D
Sample Name:  IBD-633-CHIR
Sample Info:  Phenomenex Lux Amylose-1, 3 um, 90:10, 1.0 mL/min, p=95
              bar; T=25st.C
========================================================================
```

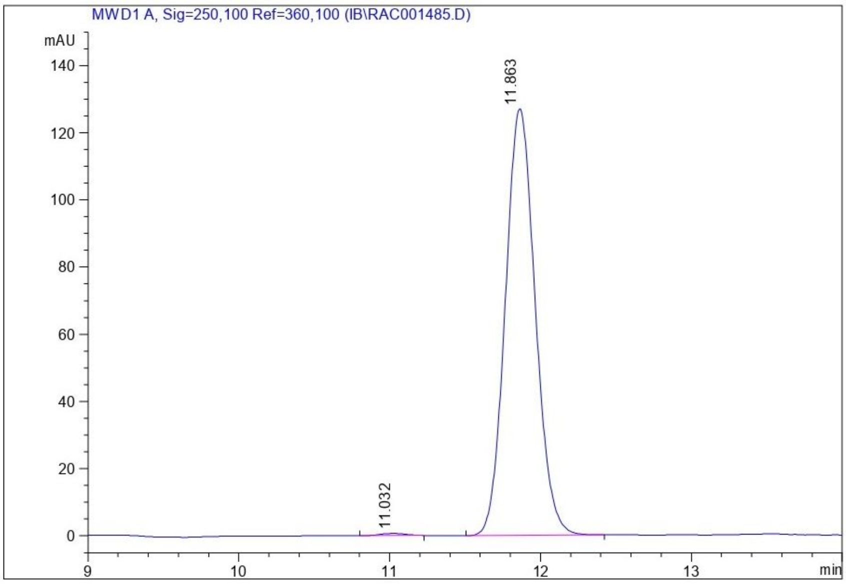


| # | Meas. R | Area | Area % |
|---|---|---|---|
| 1 | 11.032 | 7.193 | 0.410 |
| 2 | 11.863 | 1.748e3 | 99.590 |

**Figure S167.** HPLC chromatograms of **4h** (top-racemic, botom- chiral).

```
Data File:  C:\CHEM32\1\DATA\IB\RAC001492.D
Sample Name:  IBD-638-rac
Sample Info:  Phenomenex Lux Amylose-1, 3 um, 90:10, 1.0 mL/min, p=95
              bar; T=25st.C
```

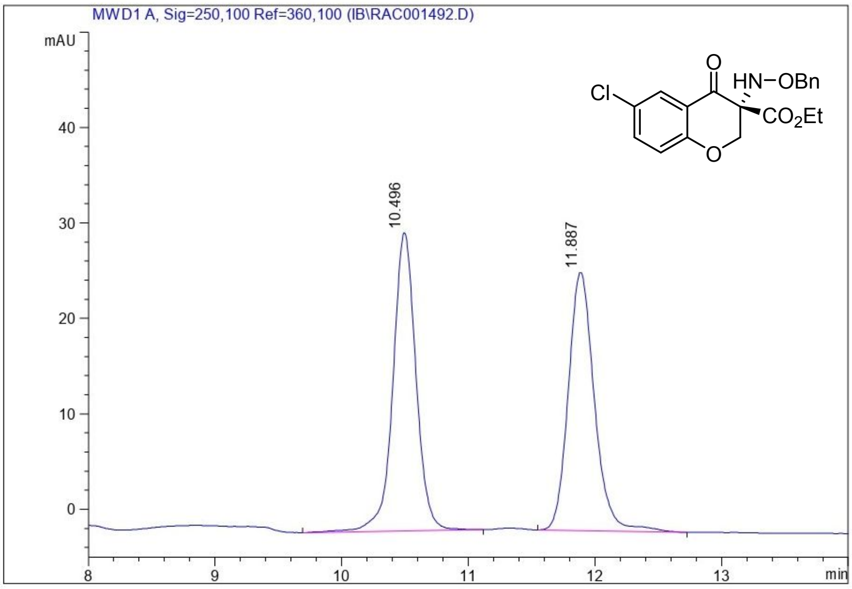


| # | Meas. R | Area | Area % |
|---|---|---|---|
| 1 | 10.496 | 397.983 | 50.611 |
| 2 | 11.887 | 388.372 | 49.389 |

```
Data File:  C:\CHEM32\1\DATA\IB\CHIR001501.D
Sample Name:  IBD-644-ch
Sample Info:  Phenomenex Lux Amylose-1, 3 um, 90:10, 1.0 mL/min, p=95
              bar; T=25st.C
```

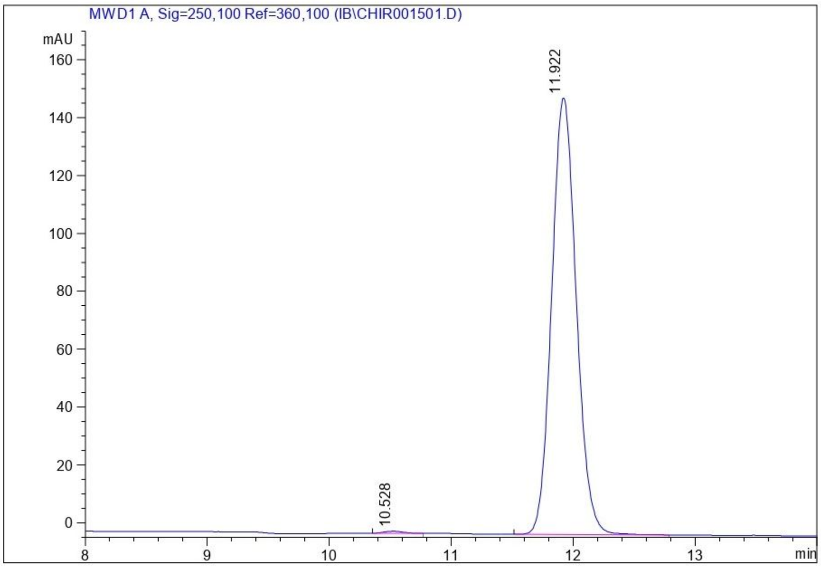


| # | Meas. R | Area | Area % |
|---|---|---|---|
| 1 | 10.528 | 8.559 | 0.404 |
| 2 | 11.922 | 2.112e3 | 99.596 |

**Figure S168**. HPLC chromatograms of **4i** (top-racemic, botom- chiral).

```
Data File:  C:\CHEM32\1\DATA\IB\RAC000994.D
Sample Name:  IBD-619-rac
Sample Info:  Phenomenex Lux Amylose-1, 3 um, 80:20, 1.0 mL/min, p=10
              6bar; T=25st.C
```

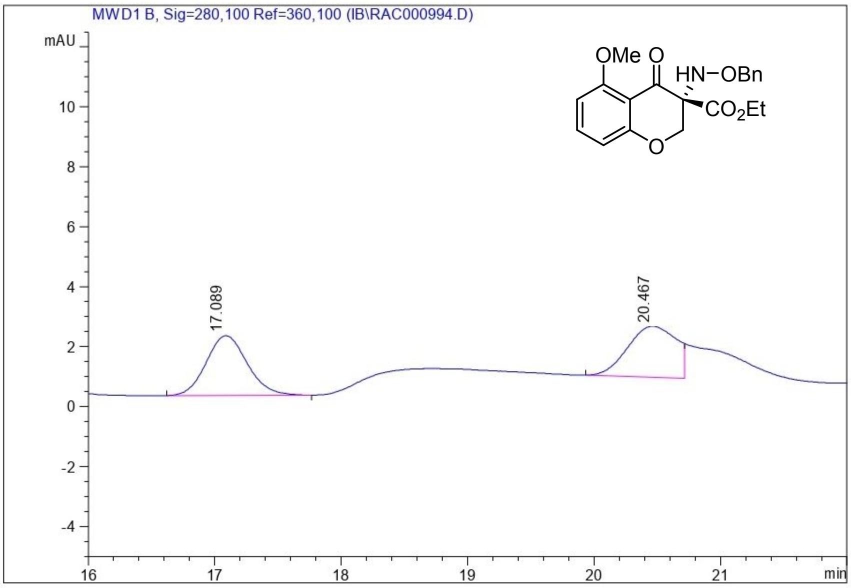


| # | Meas. R | Area | Area % |
|---|---|---|---|
| 1 | 17.089 | 45.151 | 49.844 |
| 2 | 20.467 | 45.433 | 50.156 |

```
Data File:  C:\CHEM32\1\DATA\IB\CHIR001520.D
Sample Name:  IBD-645-ch
Sample Info:  Phenomenex Lux Amylose-1, 3 um, 80:20, 1.0 mL/min, p=11
              0bar; T=25st.C
```

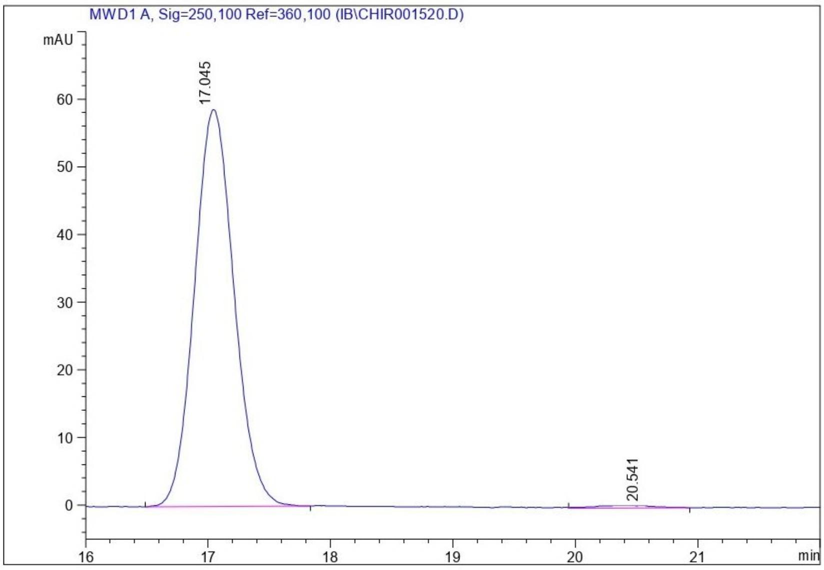


| # | Meas. R | Area | Area % |
|---|---|---|---|
| 1 | 17.045 | 1.320e3 | 99.125 |
| 2 | 20.541 | 11.648 | 0.875 |

**Figure S169**. HPLC chromatograms of **4j** (top-racemic, botom- chiral).

Data File:  C:\CHEM32\1\DATA\IB\RAC001543.D
Sample Name:  IBD-635-rac
Sample Info:  Phenomenex Lux Amylose-1, 3 um, 90:10, 1.0 mL/min, p=95
bar; T=25st.C

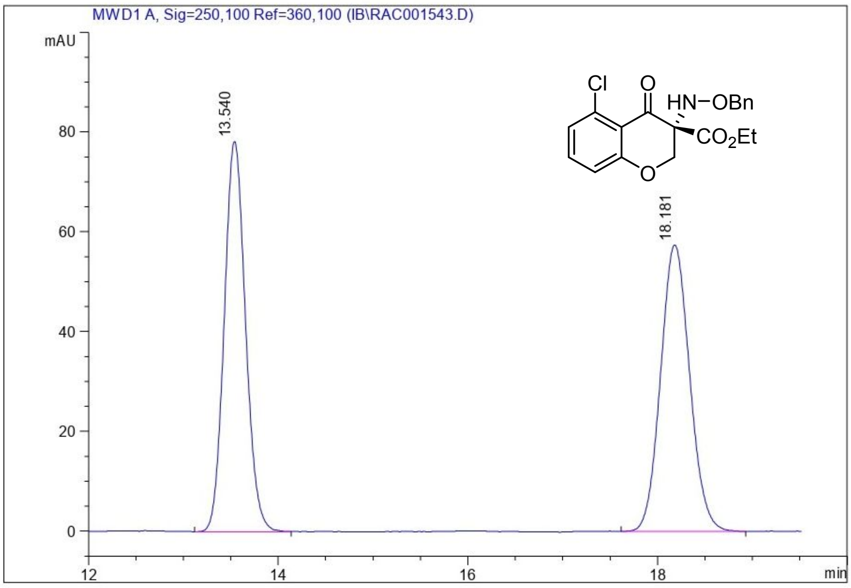


| # | Meas. R | Area | Area % |
|---|---|---|---|
| 1 | 13.540 | 1.220e3 | 49.852 |
| 2 | 18.181 | 1.227e3 | 50.148 |

Data File:  C:\CHEM32\1\DATA\IB\CHIR001545.D
Sample Name:  IBD-640-ch-II
Sample Info:  Phenomenex Lux Amylose-1, 3 um, 90:10, 1.0 mL/min, p=95
bar; T=25st.C

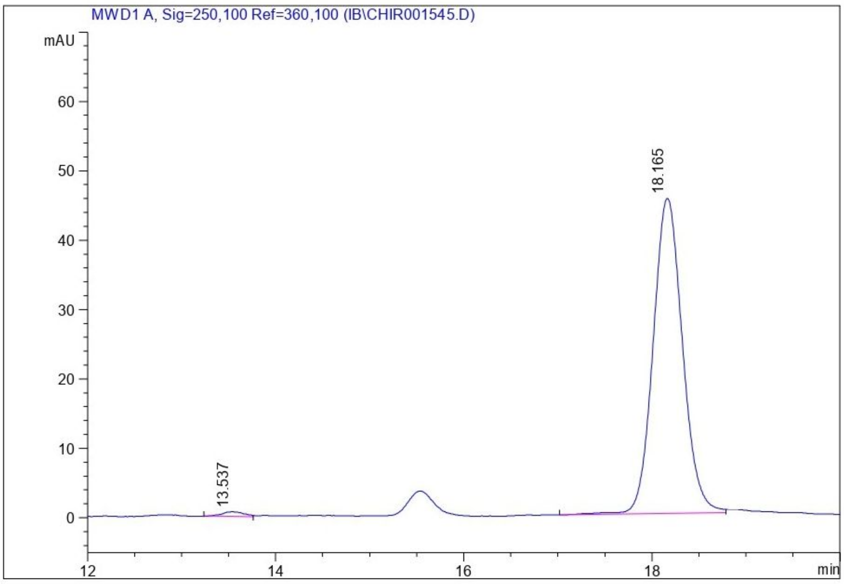


| # | Meas. R | Area | Area % |
|---|---|---|---|
| 1 | 13.537 | 10.643 | 1.068 |
| 2 | 18.165 | 985.588 | 98.932 |

**Figure S170**. HPLC chromatograms of **4k** (top-racemic, botom- chiral).

Data File: C:\CHEM32\1\DATA\IB\RAC001546.D
Sample Name: IBD-636-rac
Sample Info: Phenomenex Lux Amylose-1, 3 um, 90:10, 1.0 mL/min, p=95 bar; T=25st.C

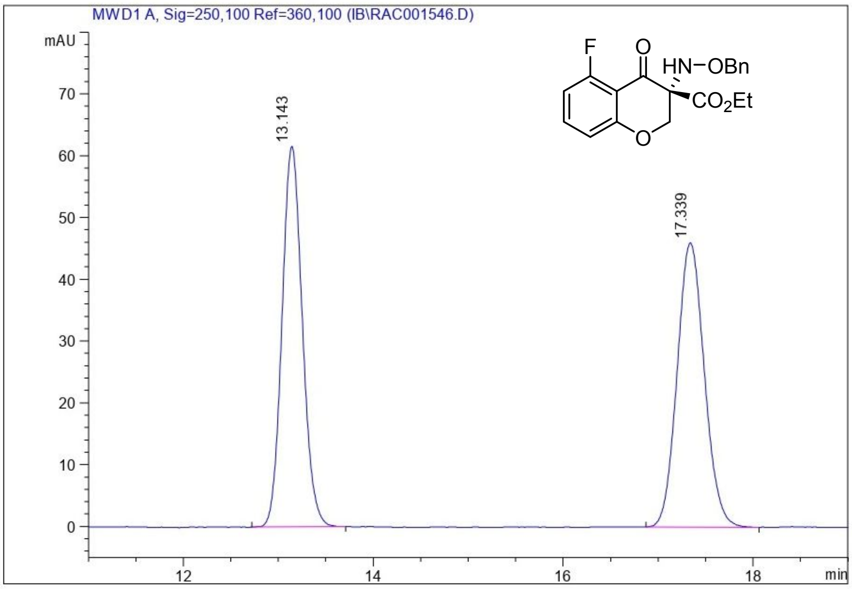


| # | Meas. R | Area | Area % |
|---|---|---|---|
| 1 | 13.143 | 914.928 | 49.939 |
| 2 | 17.339 | 917.164 | 50.061 |

Data File: C:\CHEM32\1\DATA\IB\CHIR001556.D
Sample Name: IBD-641-ch
Sample Info: Phenomenex Lux Amylose-1, 3 um, 90:10, 1.0 mL/min, p=95 bar; T=25st.C

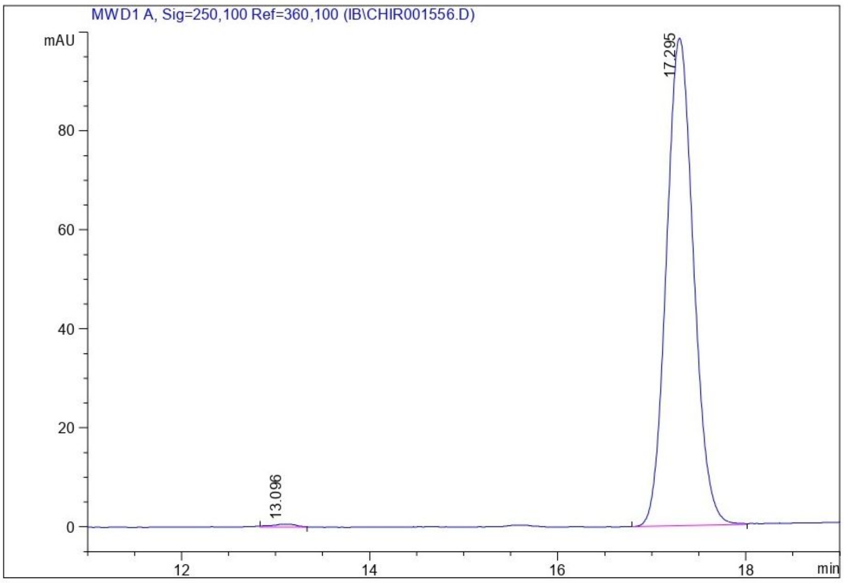


| # | Meas. R | Area | Area % |
|---|---|---|---|
| 1 | 13.096 | 12.180 | 0.612 |
| 2 | 17.295 | 1.980e3 | 99.388 |

**Figure S171**. HPLC chromatograms of **4l** (top-racemic, botom- chiral).

Data File: C:\CHEM32\1\DATA\IB\RAC000999.D
Sample Name: IBD-620-rac
Sample Info: Phenomenex Lux Cellulose-1, 3 um, 95:5, 0.8 mL/min, p=8
6bar; T=25st.C

=====================================================================

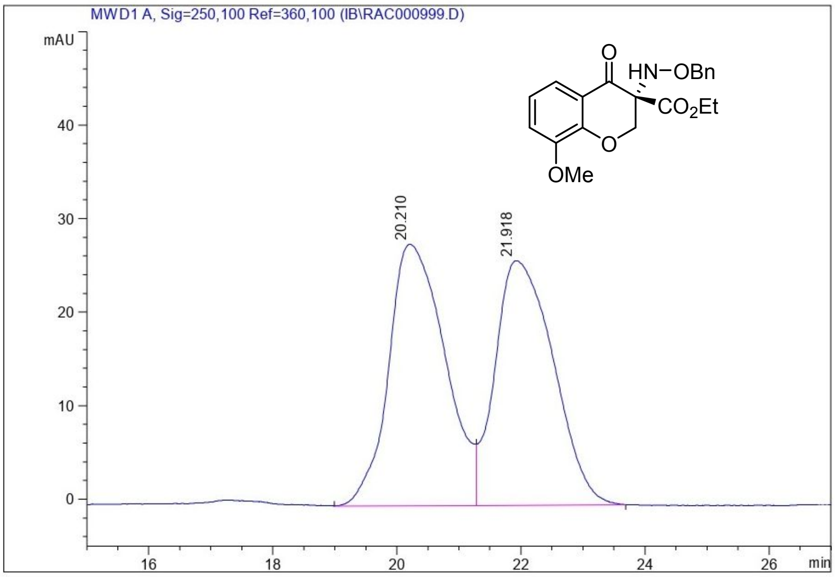


| # | Meas. R | Area | Area % |
|---|---|---|---|
| 1 | 20.210 | 1.768e3 | 50.785 |
| 2 | 21.918 | 1.714e3 | 49.215 |

Data File: C:\CHEM32\1\DATA\IB\CHIR001000.D
Sample Name: IBD-620-ch
Sample Info: Phenomenex Lux Cellulose-1, 3 um, 95:5, 0.8 mL/min, p=8
6bar; T=25st.C

=====================================================================

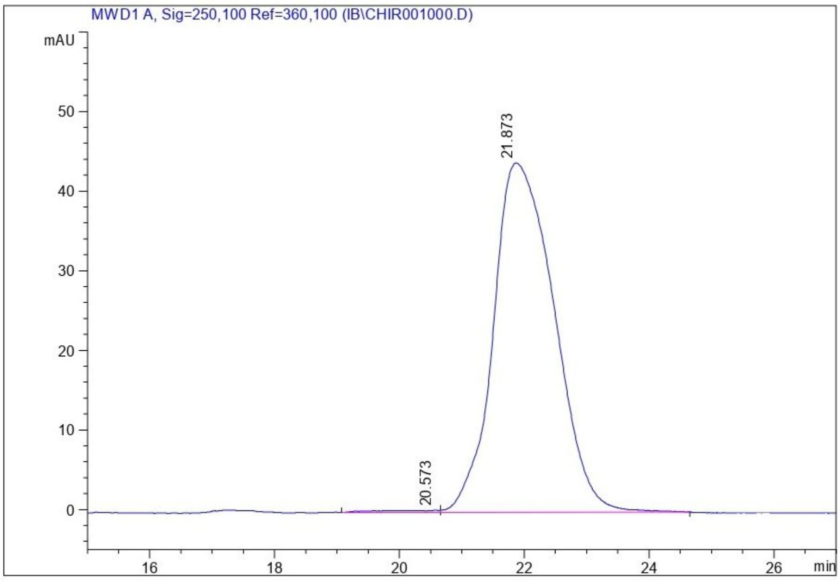


| # | Meas. R | Area | Area % |
|---|---|---|---|
| 1 | 20.573 | 19.657 | 0.657 |
| 2 | 21.873 | 2.973e3 | 99.343 |

**Figure S172**. HPLC chromatograms of **4m** (top-racemic, botom- chiral).

```
Data File:  C:\CHEM32\1\DATA\IB\RAC001430.D
Sample Name:  IBD-628-rac
Sample Info:  Phenomenex Lux Amylose-1, 3 um, 90:10, 1.0 mL/min, p=95
              bar; T=25st.C
=====================================================================
```

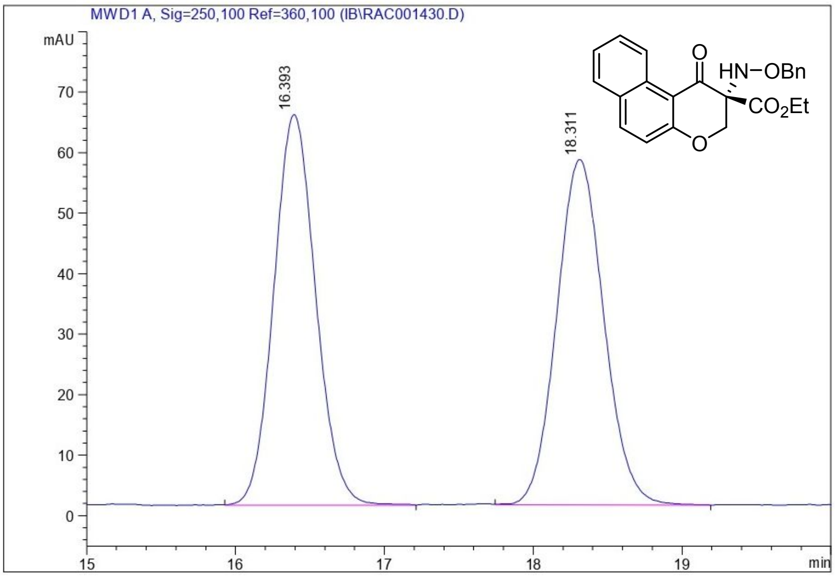


| # | Meas. R | Area | Area % |
|---|---|---|---|
| 1 | 16.393 | 1.261e3 | 50.003 |
| 2 | 18.311 | 1.261e3 | 49.997 |

```
Data File:  C:\CHEM32\1\DATA\IB\CHIR001434.D
Sample Name:  IBD-628-chir
Sample Info:  Phenomenex Lux Amylose-1, 3 um, 90:10, 1.0 mL/min, p=95
              bar; T=25st.C
=====================================================================
```

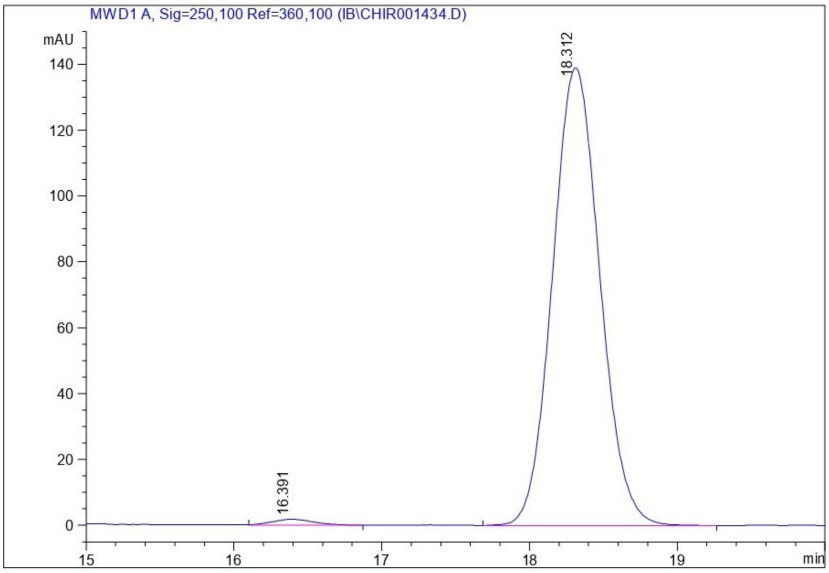


| # | Meas. R | Area | Area % |
|---|---|---|---|
| 1 | 16.391 | 34.225 | 1.094 |
| 2 | 18.312 | 3.093e3 | 98.906 |

**Figure S173**. HPLC chromatograms of **4n** (top-racemic, botom- chiral).

Data File: C:\CHEM32\1\DATA\IB\RAC000992.D
Sample Name: IBD-622-rac
Sample Info: Phenomenex Lux Amylose-1, 3 um, 90:10, 1.0 mL/min, p=96 bar; T=25st.C

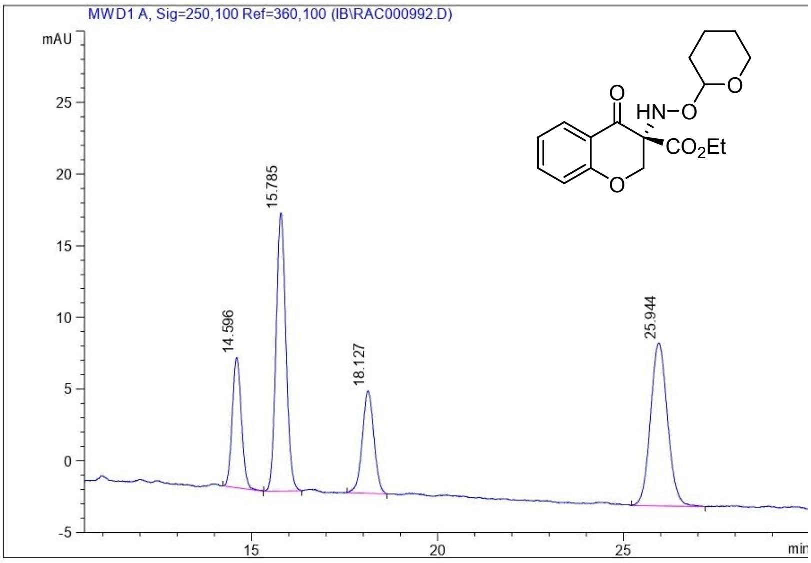


| # | Meas. R | Area | Area % |
|---|---|---|---|
| 1 | 14.596 | 155.616 | 15.087 |
| 2 | 15.785 | 358.047 | 34.714 |
| 3 | 18.127 | 155.182 | 15.045 |
| 4 | 25.944 | 362.589 | 35.154 |

Data File: C:\CHEM32\1\DATA\IB\CHIR000993.D
Sample Name: IBD-622-ch
Sample Info: Phenomenex Lux Amylose-1, 3 um, 90:10, 1.0 mL/min, p=95 bar; T=25st.C

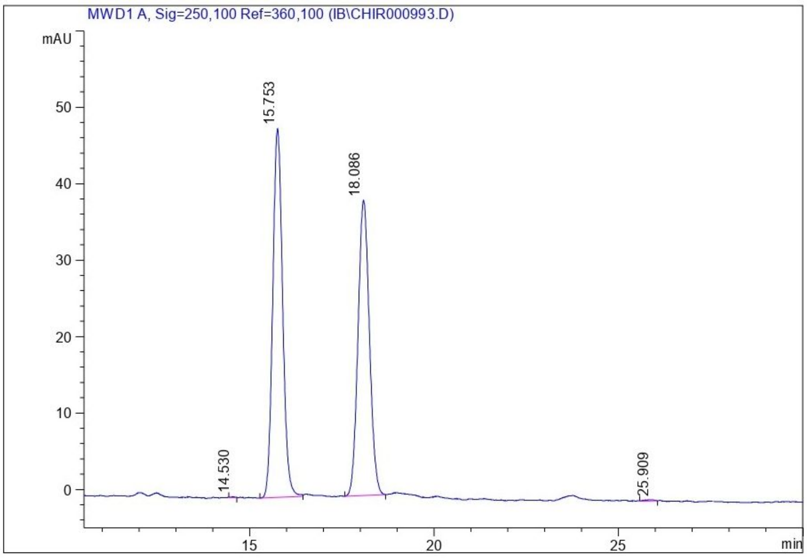


| # | Meas. R | Area | Area % |
|---|---|---|---|
| 1 | 14.530 | 1.041 | 0.060 |
| 2 | 15.753 | 896.448 | 51.797 |
| 3 | 18.086 | 829.807 | 47.946 |
| 4 | 25.909 | 3.408 | 0.197 |

**Figure S174.** HPLC chromatograms of **4o** (top-racemic, botom- chiral).

```
Data File:  C:\CHEM32\1\DATA\IB\RAC001457.D
Sample Name:  IBD-631-rac-cz
Sample Info:  Phenomenex Lux Amylose-1, 3 um, 70:30, 1.2 mL/min, p=15
              5bar; T=25st.C
```

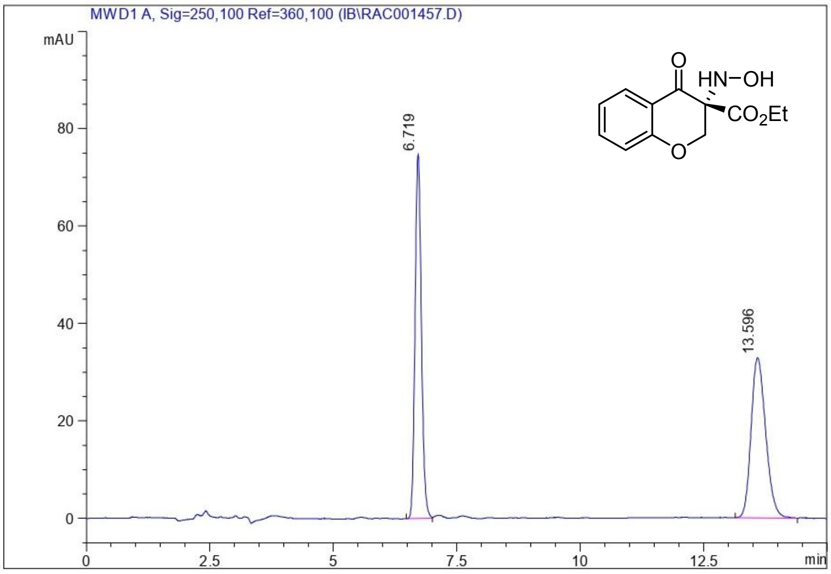


| # | Meas. R | Area | Area % |
|---|---|---|---|
| 1 | 6.719 | 666.579 | 49.680 |
| 2 | 13.596 | 675.164 | 50.320 |

```
Data File:  C:\CHEM32\1\DATA\IB\CHIR001463.D
Sample Name:  IBD-631-chir
Sample Info:  Phenomenex Lux Amylose-1, 3 um, 70:30, 1.2 mL/min, p=15
              5bar; T=25st.C
```

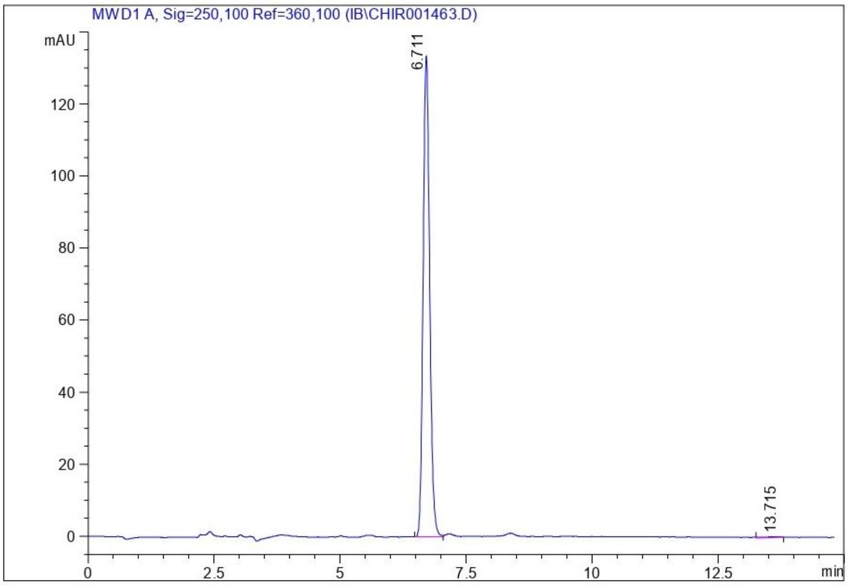


| # | Meas. R | Area | Area % |
|---|---|---|---|
| 1 | 6.711 | 1.196e3 | 99.542 |
| 2 | 13.715 | 5.502 | 0.458 |

**Figure S175**. HPLC chromatograms of **4p** (top-racemic, botom- chiral).

## 7. References


1. Thai, K.; Langdon, S.M.; Bilodeau, F.; Gravel, M. Highly chemo- and enantioselective cross-benzoin reaction of aliphatic aldehydes and α-ketoesters. *Org. Lett.* **2013**, *15*, 2214–2217.
2. Barańska, I.; Ośmiałowski, B.; Rafińska, K.; Rafiński, Z. Construction of Highly Functionalized 2-Styrylfurans by N-Heterocyclic Carbene/Brønsted Acid Catalysis. *Org. Lett.* **2024**, *26*, 3514–3518.
3. Rafiński, Z.; Kozakiewicz, A.; Rafińska, K. (-)-β-pinene-derived N-heterocyclic carbenes: Application to highly enantioselective intramolecular stetter reaction. *ACS Catal.* **2014**, *4*, 1404–1408.
4. Vora, H.U.; Lathrop, S.P.; Reynolds, N.T.; Kerr, M.S.; de Alaniz, J.R.; Rovis, T.; Chennamadhavuni, S.; Davies, H.M.L. Preparation of Chiral and Achiral Triazolium Salts: Carbene Precursors with Demonstrated Synthetic Utility. *Org. Synth.* **2010**, *87*, 350–361.
5. Swaby, C.; Taylor, A.; Greaney, M.F. An NHC-Catalyzed Desulfonylative Smiles Rearrangement of Pyrrole and Indole Carboxaldehydes. *J. Org. Chem.* **2023**, *88*, 12821–12825.
6. Nishiyama, T.; Hatae, N.; Yoshimura, T.; Takaki, S.; Abe, T.; Ishikura, M.; Hibino, S.; Choshi, T. Concise synthesis of carbazole-1,4-quinones and evaluation of their antiproliferative activity against HCT-116 and HL-60 cells. *Eur. J. Med. Chem.* **2016**, *121*, 561–577.
7. Jiang, X.; Yang, J.; Zhang, F.; Yu, P.; Yi, P.; Sun, Y.; Wang, Y. Facile Synthesis of 3-Halobenzo-heterocyclic-2-carbonyl Compounds via in situ Halogenation-Oxidation. *Adv. Synth. Catal.* **2016**, *358*, 2678–2683.
8. Jiang, X.; Zhang, F.; Yang, J.; Yu, P.; Yi, P.; Sun, Y.; Wang, Y. Fluorination-Oxidation of 2-Hydroxymethylindole Using Selectfluor. *Adv. Synth. Catal.* **2017**, *359*, 853–858.
9. Yanping, Z.; Hongjun, W.; Gong, L.; Yuanyuan, J.; Xiang, L.; Liying, Z.; Yanan, L. Rho-associated protein kinase inhibitor, pharmaceutical composition comprising the same, as well as preparation method and use thereof; EP3421465A1; European Patent Office 2019.
10. Hu, Y.E.; Kaur, J.; McFadden, R.; Murry, J.P.; Schultz, B.E.; Truong, H.H.; Yu, H. Protein Kinase C Agonists; WO2020176505A1; WIPO (PCT) 2020.
11. Rigaku Oxford Diffraction. CrysAlisPro Software System, version 1.171.43.120a; Rigaku, Oxford, UK 2024.
12. G. M. Sheldrick, Acta Crystallogr., Sect. A: Found. Adv., 2015, 71, 3–8.
13. G.M. Sheldrick, Acta Crystallogr., 2015, C71, 3–8.
14. C. F. Macrae, I. J. Bruno, J. A. Chisholm, P. R. Edgington, P. McCabe, E. Pidcock, L. Rodriguez-Monge, R. Taylor, J. Van De Streek, P. A. Wood, J. Appl. Cryst., 2008, 41, 466–470.
15. http://www.povray.org/.
